# The Japanese Clinical Practice Guidelines for Management of Sepsis and Septic Shock 2024

**DOI:** 10.1186/s40560-025-00776-0

**Published:** 2025-03-14

**Authors:** Nobuaki Shime, Taka-aki Nakada, Tomoaki Yatabe, Kazuma Yamakawa, Yoshitaka Aoki, Shigeaki Inoue, Toshiaki Iba, Hiroshi Ogura, Yusuke Kawai, Atsushi Kawaguchi, Tatsuya Kawasaki, Yutaka Kondo, Masaaki Sakuraya, Shunsuke Taito, Kent Doi, Hideki Hashimoto, Yoshitaka Hara, Tatsuma Fukuda, Asako Matsushima, Moritoki Egi, Shigeki Kushimoto, Takehiko Oami, Kazuya Kikutani, Yuki Kotani, Gen Aikawa, Makoto Aoki, Masayuki Akatsuka, Hideki Asai, Toshikazu Abe, Yu Amemiya, Ryo Ishizawa, Tadashi Ishihara, Tadayoshi Ishimaru, Yusuke Itosu, Hiroyasu Inoue, Hisashi Imahase, Haruki Imura, Naoya Iwasaki, Noritaka Ushio, Masatoshi Uchida, Michiko Uchi, Takeshi Umegaki, Yutaka Umemura, Akira Endo, Marina Oi, Akira Ouchi, Itsuki Osawa, Yoshiyasu Oshima, Kohei Ota, Takanori Ohno, Yohei Okada, Hiromu Okano, Yoshihito Ogawa, Masahiro Kashiura, Daisuke Kasugai, Ken-ichi Kano, Ryo Kamidani, Akira Kawauchi, Sadatoshi Kawakami, Daisuke Kawakami, Yusuke Kawamura, Kenji Kandori, Yuki Kishihara, Sho Kimura, Kenji Kubo, Tomoki Kuribara, Hiroyuki Koami, Shigeru Koba, Takehito Sato, Ren Sato, Yusuke Sawada, Haruka Shida, Tadanaga Shimada, Motohiro Shimizu, Kazushige Shimizu, Takuto Shiraishi, Toru Shinkai, Akihito Tampo, Gaku Sugiura, Kensuke Sugimoto, Hiroshi Sugimoto, Tomohiro Suhara, Motohiro Sekino, Kenji Sonota, Mahoko Taito, Nozomi Takahashi, Jun Takeshita, Chikashi Takeda, Junko Tatsuno, Aiko Tanaka, Masanori Tani, Atsushi Tanikawa, Hao Chen, Takumi Tsuchida, Yusuke Tsutsumi, Takefumi Tsunemitsu, Ryo Deguchi, Kenichi Tetsuhara, Takero Terayama, Yuki Togami, Takaaki Totoki, Yoshinori Tomoda, Shunichiro Nakao, Hiroki Nagasawa, Yasuhisa Nakatani, Nobuto Nakanishi, Norihiro Nishioka, Mitsuaki Nishikimi, Satoko Noguchi, Suguru Nonami, Osamu Nomura, Katsuhiko Hashimoto, Junji Hatakeyama, Yasutaka Hamai, Mayu Hikone, Ryo Hisamune, Tomoya Hirose, Ryota Fuke, Ryo Fujii, Naoki Fujie, Jun Fujinaga, Yoshihisa Fujinami, Sho Fujiwara, Hiraku Funakoshi, Koichiro Homma, Yuto Makino, Hiroshi Matsuura, Ayaka Matsuoka, Tadashi Matsuoka, Yosuke Matsumura, Akito Mizuno, Sohma Miyamoto, Yukari Miyoshi, Satoshi Murata, Teppei Murata, Hiromasa Yakushiji, Shunsuke Yasuo, Kohei Yamada, Hiroyuki Yamada, Ryo Yamamoto, Ryohei Yamamoto, Tetsuya Yumoto, Yuji Yoshida, Shodai Yoshihiro, Satoshi Yoshimura, Jumpei Yoshimura, Hiroshi Yonekura, Yuki Wakabayashi, Takeshi Wada, Shinichi Watanabe, Atsuhiro Ijiri, Kei Ugata, Shuji Uda, Ryuta Onodera, Masaki Takahashi, Satoshi Nakajima, Junta Honda, Tsuguhiro Matsumoto

**Affiliations:** 1 Department of Emergency and Critical Care Medicine, Graduate School of Biomedical and Health Sciences, Hiroshima University, 1-2-3 Kasumi, Minami-ku, Hiroshima, 734-8551 Japan; 2 Department of Emergency and Critical Care Medicine, Chiba University Graduate School of Medicine, Chiba, Japan; 3 Emergency Department, Nishichita General Hospital, Tokai, Japan; 4 Department of Emergency and Critical Care Medicine, Osaka Medical and Pharmaceutical University, Takatsuki, Japan; 5 Department of Anesthesiology and Intensive Care Medicine, Hamamatsu University School of Medicine, Hamamatsu, Japan; 6 Department of Emergency and Critical Care Medicine, Wakayama Medical University, Wakayama, Japan; 7 Department of Emergency and Disaster Medicine, Juntendo University, Tokyo, Japan; 8 Department of Traumatology and Acute Critical Medicine, Osaka University Graduate School of Medicine, Suita, Japan; 9 Department of Nursing, Fujita Health University Hospital, Toyoake, Japan; 10 Division of Pediatric Critical Care, Department of Pediatrics, School of Medicine, St. Marianna University, Kawasaki, Japan; 11 Department of Pediatric Critical Care, Shizuoka Children’s Hospital, Shizuoka, Japan; 12 Department of Emergency and Critical Care Medicine, Juntendo University, Urayasu Hospital, Urayasu, Japan; 13 Department of Emergency and Intensive Care Medicine, JA Hiroshima General Hospital, Hatsukaichi, Japan; 14 Division of Rehabilitation, Department of Clinical Practice and Support, Hiroshima University Hospital, Hiroshima, Japan; 15 Department of Emergency and Critical Care Medicine, The University of Tokyo, Tokyo, Japan; 16 Department of Infectious Diseases, Hitachi Medical Education and Research Center University of Tsukuba Hospital, Hitachi, Japan; 17 Department of Anesthesiology and Critical Care Medicine, Fujita Health University School of Medicine, Toyoake, Japan; 18 Department of Emergency and Critical Care Medicine, Toranomon Hospital, Tokyo, Japan; 19 Department of Emergency and Critical Care, Nagoya City University Graduate School of Medical Sciences, Nagoya, Japan; 20 Department of Anesthesia and Intensive Care, Kyoto University Hospital, Kyoto, Japan; 21 Division of Emergency and Critical Care Medicine, Tohoku University Graduate School of Medicine, Sendai, Japan; 22 Department of Intensive Care Medicine Kameda Medical Center, Kamogawa, Japan; 23 Department of Adult Health Nursing, College of Nursing, Ibaraki Christian University, Hitachi, Japan; 24 Division of Traumatology, National Defense Medical College Research Institute, Tokorozawa, Japan; 25 Department of Intensive Care Medicine, Sapporo Medical University School of Medicine, Sapporo, Japan; 26 Department of Emergency and Critical Care Medicine, Nara Medical University, Nara, Japan; 27 Department of Emergency and Critical Care Medicine, Tsukuba Memorial Hospital, Tsukuba, Japan; 28 Department of Critical Care and Emergency Medicine, Tokyo Metropolitan Tama Medical Center, Tokyo, Japan; 29 Department of Emergency Medicine, Chiba Kaihin Municipal Hospital, Chiba, Japan; 30 Department of Anesthesiology, Hokkaido University Hospital, Sapporo, Japan; 31 Division of Physical Therapy, Department of Rehabilitation, Showa University School of Nursing and Rehabilitation Sciences, Yokohama, Japan; 32 Division of Intensive Care, Department of Anesthesiology and Intensive Care Medicine, Jichi Medical University School of Medicine, Shimotsuke, Japan; 33 Department of Infectious Diseases, Rakuwakai Otowa Hospital, Kyoto, Japan; 34 Department of Anesthesiology and Intensive Care Medicine, Nagasaki University Graduate School of Biomedical Sciences, Nagasaki, Japan; 35 Department of Emergency and Critical Care Medicine, Dokkyo Medical University, Tochigi, Japan; 36 National Hospital Organization Ibarakihigashi National Hospital, Naka-Gun, Japan; 37 Department of Anesthesiology, Kansai Medical University, Hirakata, Japan; 38 Division of Trauma and Surgical Critical Care, Osaka General Medical Center, Osaka, Japan; 39 Department of Acute Critical Care Medicine, Tsuchiura Kyodo General Hospital, Tsuchiura, Japan; 40 Department of Emergency and Critical Care Medicine, Kitasato University School of Medicine, Sagamihara, Japan; 41 Department of Pharmacy, Kobe Tokushukai Hospital, Kobe, Japan; 42 Department of Emergency and Crical Care Medicine, Shin-Yurigaoka General Hospital, Kawasaki, Japan; 43 Department of Preventive Services, Kyoto University, Kyoto, Japan; 44 Department of Critical Care Medicine, St. Luke’s International Hospital, Tokyo, Japan; 45 Department of Emergency and Critical Care Medicine, Jichi Medical University Saitama Medical Center, Saitama, Japan; 46 Department of Emergency and Critical Care Medicine, Nagoya University Graduate School of Medicine, Nagoya, Japan; 47 Department of Emergency Medicine, Fukui Prefectural Hospital, Fukui, Japan; 48 Department of Emergency and Disaster Medicine, Gifu University Graduate School of Medicine, Gifu, Japan; 49 Department of Critical Care and Emergency Medicine, Japanese Red Cross Maebashi Hospital, Maebashi, Japan; 50 Department of Anesthesiology, Cancer Institute Hospital of Japanese Foundation for Cancer Research, Tokyo, Japan; 51 Department of Intensive Care Medicine, Aso Iizuka Hospital, Iizuka, Japan; 52 Department of Rehabilitation, Showa General Hospital, Tokyo, Japan; 53 Department of Emergency and Critical Care Medicine, Japanese Red Cross Society Kyoto Daini Hospital , Kyoto, Japan; 54 Department of Pediatric Critical Care Medicine, Tokyo Women’s Medical University Yachiyo Medical Center, Yachiyo, Japan; 55 Department of Emergency Medicine, Japanese Red Cross Wakayama Medical Center, Wakayama, Japan; 56 Department of Infectious Diseases, Japanese Red Cross Wakayama Medical Center, Wakayama, Japan; 57 Department of Acute and Critical Care Nursing, School of Nursing, Sapporo City University, Sapporo, Japan; 58 Department of Emergency and Critical Care Medicine, Saga University, Saga, Japan; 59 Department of Critical Care Medicine, Nerima Hikarigaoka Hospital, Nerima, Japan; 60 Department of Anesthesiology, Nagoya University Hospital, Nagoya, Japan; 61 Department of Nursing, Tokyo Medical University Hospital, Shinjuku, Japan; 62 Department of Emergency Medicine, Gunma University Graduate School of Medicine, Maebashi, Japan; 63 Data Science, Medical Division, AstraZeneca K.K, Osaka, Japan; 64 Department of Intensive Care Medicine, Ryokusen-Kai Yonemori Hospital, Kagoshima, Japan; 65 Department of Clinical Engineer, Tenri Hospital, Tenri, Japan; 66 Department of Cardiology, Aizawa Hospital, Matsumoto, Japan; 67 The Advanced Emergency and Critical Care Center, Mie University Hospital, Tsu, Japan; 68 Department of Emergency Medicine, Asahiakwa Medical University, Asahikawa, Japan; 69 Department of Anesthesiology and Intensive Care, Gunma University, Maebashi, Japan; 70 Department of Internal Medicine, National Hospital Organization Kinki-Chuo Chest Medical Center, Osaka, Japan; 71 Department of Anesthesiology, Keio University School of Medicine, Shinjuku, Japan; 72 Department of Intensive Care Medicine, Miyagi Children’s Hospital, Sendai, Japan; 73 Department of Nursing, Hiroshima University Hospital, Hiroshima, Japan; 74 Centre for Heart Lung Innovation, University of British Columbia, Vancouver, British Columbia Canada; 75 Department of Anesthesiology, Osaka Women’s and Children’s Hospital, Izumi, Japan; 76 Department of Nursing, Kokura Memorial Hospital, Kitakyushu, Japan; 77 Department of Intensive Care, University of Fukui Hospital, Fukui, Japan; 78 Division of Critical Care Medicine, Saitama Children’s Medical Center, Saitama, Japan; 79 Department of Pulmonary, Yokohama City University Hospital, Yokohama, Japan; 80 Department of Emergency Medicine, National Hospital Organization Mito Medical Center, Ibaragi, Japan; 81 Department of Traumatology and Critical Care Medicine, Osaka Metropolitan University Hospital, Osaka, Japan; 82 Department of Critical Care Medicine, Fukuoka Children’s Hospital, Fukuoka, Japan; 83 Department of Emergency Self-Defense, Forces Central Hospital, Tokyo, Japan; 84 Department of Acute Medicine & Critical Care Medical Center, National Hospital Organization Osaka National Hospital, Osaka, Japan; 85 Department of Anesthesiology, Kyushu University Beppu Hospital, Beppu, Japan; 86 Laboratory of Clinical Pharmacokinetics, Research and Education Center for Clinical Pharmacy, Kitasato University School of Pharmacy, Tokyo, Japan; 87 Department of Acute Critical Care Medicine, Shizuoka Hospital Juntendo University, Shizuoka, Japan; 88 Department of Nursing, Osaka University Hospital, Osaka, Japan; 89 Department of Disaster and Emergency Medicine, Kobe University, Kobe, Japan; 90 Department of Anesthesiology, Hirosaki University Graduate School of Medicine, Hirosaki, Japan; 91 Department of Emergency and Critical Care Medicine, Kyoto Katsura Hospital, Kyoto, Japan; 92 Medical Education Development Center, Gifu University, Gifu, Japan; 93 Department of Emergency and Intensive Care Medicine, Fukushima Medical University, Fukushima, Japan; 94 Department of Emergency Medicine, Tokyo Metropolitan Bokutoh Hospital, Tokyo, Japan; 95 Department of Internal Medicine, IMS Meirikai Sendai General Hospital, Sendai, Japan; 96 Emergency Department, Ageo Central General Hospital, Ageo, Japan; 97 Department of Pharmacy, Osaka Psychiatric Medical Center, Hirakata, Japan; 98 Emergency and Critical Care Center, Kurashiki Central Hospital, Kurashiki, Japan; 99 Department of Emergency Medicine, Kakogawa Central City Hospital, Kakogawa, Japan; 100 Department of Emergency Medicine, Tokyo Hikifune Hospital, Tokyo, Japan; 101 Department of Infectious Diseases, Tokyo Hikifune Hospital, Tokyo, Japan; 102 Department of Emergency and Critical Care Medicine, Tokyobay Urayasu Ichikawa Medical Center, Urayasu, Japan; 103 Department of Emergency and Critical Care Medicine, Keio University School of Medicine, Shinjuku, Japan; 104 Osaka Prefectural Nakakawachi Emergency and Critical Care Center, Higashiosaka, Japan; 105 Department of Intensive Care, Chiba Emergency and Psychiatric Medical Center, Chiba, Japan; 106 Department of Emergency and Critical Care Medicine, St. Luke’s International Hospital, Chuo-Ku, Japan; 107 Division of Emergency Medicine, Hyogo Prefectural Kobe Children’s Hospital, Kobe, Japan; 108 Department of Cardiology Miyazaki Prefectural, Nobeoka Hospital, Nobeoka, Japan; 109 Department of Emergency Medicine, Yakushiji Jikei Hospital, Soja, Japan; 110 Department of Radiology, Tenri Hospital, Tenri, Japan; 111 Department of Traumatology and Critical Care Medicine, National Defense Medical College Hospital, Saitama, Japan; 112 Department of Primary Care and Emergency Medicine, Graduate School of Medicine, Kyoto University, Kyoto, Japan; 113 Center for Innovative Research for Communities and Clinical Excellence (CIRC2LE), Fukushima Medical University, Fukushima, Japan; 114 Department of Emergency, Critical Care and Disaster Medicine, Faculty of Medicine, Dentistry and Pharmaceutical Sciences, Okayama University, Okayama, Japan; 115 Department of Pharmaceutical Services, Hiroshima University Hospital, Hiroshima, Japan; 116 Department of Emergency Medicine, Rakuwakai Otowa Hospital, Kyoto, Japan; 117 Department of Anesthesiology and Pain Medicine, Fujita Health University Bantane Hospital, Nagoya, Japan; 118 Department of Nursing, Kobe City Medical Center General Hospital, Kobe, Japan; 119 Division of Acute and Critical Care Medicine, Department of Anesthesiology and Critical Care Medicine, Faculty of Medicine, Hokkaido University, Sapporo, Japan; 120 Department of Physical Therapy, Faculty of Rehabilitation Gifu, University of Health Science, Gifu, Japan; 121 Department of Intensive Care Medicine, Matsue Red Cross Hospital, Matsue, Japan; 122 Department of Emergency Medicine, Kyoto Prefectural University of Medicine, Kyoto, Japan

**Keywords:** Evidence-based medicine, GRADE, Infection, Intensive care, Organ failure, Systematic review

## Abstract

The 2024 revised edition of the Japanese Clinical Practice Guidelines for Management of Sepsis and Septic Shock (J-SSCG 2024) is published by the Japanese Society of Intensive Care Medicine and the Japanese Association for Acute Medicine. This is the fourth revision since the first edition was published in 2012. The purpose of the guidelines is to assist healthcare providers in making appropriate decisions in the treatment of sepsis and septic shock, leading to improved patient outcomes. We aimed to create guidelines that are easy to understand and use for physicians who recognize sepsis and provide initial management, specialized physicians who take over the treatment, and multidisciplinary healthcare providers, including nurses, physical therapists, clinical engineers, and pharmacists. The J-SSCG 2024 covers the following nine areas: diagnosis of sepsis and source control, antimicrobial therapy, initial resuscitation, blood purification, disseminated intravascular coagulation, adjunctive therapy, post-intensive care syndrome, patient and family care, and pediatrics. In these areas, we extracted 78 important clinical issues. The GRADE (Grading of Recommendations Assessment, Development and Evaluation) method was adopted for making recommendations, and the modified Delphi method was used to determine recommendations by voting from all committee members. As a result, 42 GRADE-based recommendations, 7 good practice statements, and 22 information-to-background questions were created as responses to clinical questions. We also described 12 future research questions.

**Supplementary Information:**

The online version contains supplementary material available at 10.1186/s40560-025-00776-0.

## Background

Sepsis is a serious condition leading to deaths, and the World Health Organization designated it as an issue to be addressed worldwide in 2017. The Japanese Clinical Practice Guidelines for Management of Sepsis and Septic Shock 2024 (J-SSCG 2024) provides information on diagnosis, treatment, and patient and family care to patients with sepsis and all related healthcare providers, aiming to improve the quality of medical treatment and mortality rate. The first edition of the J-SSCG was published in 2012, with the current revision being the fourth edition. Upon creating the J-SSCG 2024, we carefully selected critical clinical issues (clinical questions, CQs) that are mainly related to sepsis and reduced the number of CQs from 118 in the J-SSCG 2020 to 78. Utilizing our accumulated expertise in creating guidelines, we comprehensively collected the latest evidence, which was then analyzed using standard methods and evaluated using objective methods in accordance with the GRADE (Grading of Recommendations Assessment, Development and Evaluation) system. Additionally, we aimed to create “user-friendly guidelines” that provide useful information to a wide range of healthcare providers from beginners to experts. The current guidelines are filled with the expertise of the working group members, committee members, and directors of the Japanese Society of Intensive Care Medicine (JSICM) and the Japanese Association for Acute Medicine (JAAM). We hope that the guidelines will be used and evaluated by many relevant parties, ultimately leading to improved outcomes for as many patients with sepsis as possible.

## Basic principles and overview of the guidelines

### Name

The name of the guidelines is the “Japanese Clinical Practice Guidelines for Management of Sepsis and Septic Shock 2024,” with the abbreviated designation “J-SSCG 2024” in consideration of the comparison made with the international version.

### Objective

Sepsis is a serious disease that affects individuals of all ages, and the present clinical practice guidelines aim to assist healthcare providers in making decisions to improve outcomes in patients with sepsis. The guidelines are mainly intended to be used in medical institutions in Japan, and caution is required when they are used in different medical environments.

### Target patients

The guidelines target patients, including children and adults, who have or are suspected of having sepsis or septic shock. These include patients who receive diagnosis and treatment not only in an intensive care unit (ICU) but also in general wards and emergency room (ER). However, because patients with sepsis require high-intensity medical care, the guidelines mainly focus on patients receiving intensive care or its equivalent.

### Reflection of patients’ values

In order to reflect the values of patients with sepsis and their families, healthcare providers whose family members had sepsis were included in the committee as patient representatives.

### Funding for creating the guidelines

The present guidelines were prepared with financial support from the JSICM and the JAAM. None of the members received any compensation for creating the guidelines.

### Revision schedule

The present guidelines are scheduled to undergo revision every 4 years, with the next revision scheduled for 2028. Should important findings warranting revision be obtained beforehand, partial revision will be considered.

## Methods for creating the guidelines and interpretation of recommendations

For the definition and diagnosis of sepsis, we adopted the definition of sepsis-3, which is used worldwide. [1]

### Important clinical issues

The current revision focused on clinical issues that were considered important in sepsis treatment, and we excluded clinical issues that have already been included in current practice or had too uncertain evidence to create recommendations. Clinical issues were classified into CQ and future research question (FRQ). Additionally, we created recommendations for CQs, according to the GRADE systems or good practice statement (GPS), and provided the information as background questions. We also summarized the background for FRQs.

### Searching, collecting, and integrating evidence through systematic reviews

We conducted a comprehensive literature search for each CQ and extracted randomized controlled trials (RCTs), as well as observational studies as necessary. In principle, evidence was integrated based on the GRADE methodology. The literature search was conducted based on multiple databases, including CENTRAL, PubMed, and Igaku Chuo Zasshi, and we added EMBASE, CINAHL, PsycINFO, and other databases as necessary. When adopting the CQs included in the J-SSCG 2020, we conducted a systematic review of the literature published after the last search. The risk of bias was evaluated according to the method of RoB 2.0 [2] for RCTs and that of ROBINS-I for observational studies [3]. Meta-analyses were conducted using RevMan 5. An Evidence to Decision table was created, and recommendations were formulated through discussions at the committee meetings. The modified Delphi method was used for consensus building among the committee members. Each committee member anonymously voted online in an independent manner using a point system between 1 and 9 (1: disagree, 9: agree). The median and disagreement index (DI) of the obtained scores were calculated. Consensus was established when the median was ≥7 and DI was <0.3. For GPS, the median of ≥8 and a DI of <0.20 were set as thresholds for consensus building.

The strength of recommendations based on the GRADE system was classified into the following four categories: “Recommended,” “Weakly recommended,” “Weakly not recommended,” and “Not recommended” (Table 1). The interpretation of certainty of evidence is described in Table 2.

**Table 1:** Interpretation of strong and weak recommendations

Strength of recommendation	Notation	Example
Strong recommendation for the intervention	1	We recommend –
Weak recommendation for the intervention	2	We suggest –
Weak recommendation against the intervention	2	We suggest against –
Strong recommendation against the intervention	1	We recommend against –

**Table 2:** Interpretation of certainty of evidence

Certainty of evidence	Notation	Explanation
High	A	High confidence in the estimated value of effects
Moderate	B	Moderate confidence in the estimated value of effects
Low	C	Limited confidence in the estimated value of effects
Very low	D	Little confidence in the estimated value of effects

## Quick reference list of CQs & answers

### CQ1 Diagnosis and source control.

#### CQ1-1: Definition of sepsis

*Answer*: Sepsis is defined as a “life-threatening organ dysfunction caused by a dysregulated host response to infection” (*Provision of information for background question*).

#### CQ1-2: Diagnosis and severity classification of sepsis

*Answer*: Sepsis is diagnosed when there is an acute increase in the Sequential Organ Failure Assessment (SOFA) score of ≥ 2 points in the presence of a confirmed or suspected infection. Additionally, septic shock is diagnosed in patients with sepsis when a patient requires vasopressors to maintain a mean arterial pressure of ≥ 65 mmHg and has a blood lactate level > 2 mmol/L (18 mg/dL) despite adequate fluid resuscitation (*Provision of information for background question*).

#### CQ1-3: What methods are there for early detection of sepsis in general wards and emergency room (ER)?

*Answer*: Methods for early detection of sepsis in general wards and ER include screening tools, such as quick SOFA (qSOFA) and early warning scores (*Provision of information for background question*).

#### CQ1-4: When and how are blood culture samples collected for patients suspected with sepsis?

*Answer*: At least two sets of blood culture samples are collected before antimicrobial administration for patients suspected with sepsis (*Good Practice Statement*).

#### CQ1-5: When and how are culture specimens other than blood culture samples collected for patients suspected with sepsis?

*Answer*: Culture specimens are collected from the site of suspected infection before antimicrobial administration for patients suspected with sepsis (*Good Practice Statement*).

#### CQ1-6: What are the roles of C-reactive protein (CRP), procalcitonin (PCT), presepsin (P-SEP), and interleukin 6 (IL-6) as biomarkers for sepsis diagnosis?

*Answer*: CRP, PCT, P-SEP, or IL-6 alone has not been shown to have high diagnostic accuracy for sepsis in general wards, ER, or ICU. Therefore, the diagnosis of sepsis using any specific biomarker is generally considered difficult. The biomarkers are used as supplementary indicators in addition to observation of general conditions (*Provision of information for background question*).

#### CQ1-7: Are imaging tests performed to identify the source of infection in patients suspected of having sepsis?

*Answer*: Appropriate imaging tests are conducted according to the suspected disease in patients suspected with sepsis (*Good Practice Statement*).

#### CQ1-8: When is the source of infection controlled in patients with sepsis?

*Answer*: The source of infection is controlled as soon as possible after recognition of sepsis (*Good Practice Statement*).

#### CQ1-9: Which facility is appropriate for managing patients with sepsis who are unresponsive to initial fluid resuscitation?

*Answer*: Patients with sepsis who are unresponsive to initial fluid resuscitation are managed in a facility capable of providing intensive care (*Good Practice Statement*).

### CQ2 Antimicrobial therapy

#### CQ2-1: Is Gram stain testing useful for selecting empiric antimicrobials for sepsis?

*Answer*: We suggest using Gram stain testing for selecting empiric antimicrobials for sepsis (GRADE 2C).

#### CQ2-2: Is the administration of empiric antimicrobials for sepsis started within 1 h after diagnosing sepsis?

*Answer*: Although antimicrobials should be started as soon as possible after sepsis or septic shock is diagnosed, we suggest against the use of < 1 h target time (GRADE 2C).

#### CQ2-3: How are empiric antimicrobials selected for sepsis?

*Answer*: Empiric antimicrobials for sepsis are selected for each suspected source of infection by estimating the causative microorganism based on patient background and epidemiology. Rapid microbial diagnostic tests, tissue penetration, and the possibility of resistant bacteria are also assessed (*Provision of information for background question*). (See Additional file 1 and 2).

#### CQ2-4. Under what circumstances is carbapenem included in empiric antimicrobials for sepsis?

*Answer*: Carbapenem is included in empiric antimicrobials for sepsis when an infection is expected to be caused by a microorganism with susceptibility limited to carbapenems, such as extended-spectrum beta-lactamase (ESBL)-producing Enterobacterales, antibiotic-resistant *Pseudomonas aeruginosa*, or *Acinetobacter* spp. (*Provision of information for background question*).

#### CQ2-5: Under what circumstances are empiric antimicrobials against MRSA or atypical pathogens (such as Candida, viruses, Legionella, Rickettsia, and *Clostridioides difficile*) selected for sepsis?

*Answer*: Empiric antimicrobials against MRSA or atypical pathogens are selected when an infection is suspected to be caused by each of these microorganisms based on the infection focus, patient background, or microbiological findings for sepsis (*Provision of information for background question*).

#### CQ2-6: What is used as a reference for adjusting the doses of renally-excreted antimicrobials for sepsis?

*Answer*: Renal function tests measured at multiple time points, changes in body fluids, as well as the presence of renal replacement therapy and other extracorporeal circulation, are used as references for adjusting the doses of renally-excreted antimicrobials for sepsis (*Provision of information for background question*).

#### CQ2-7: Is continuous or extended infusion of antimicrobials used for sepsis?

*Answers*: We suggest using continuous or extended infusion of β-lactam antimicrobials for sepsis (GRADE 2B).

We suggest against using continuous or extended infusion of glycopeptide antimicrobials for sepsis (GRADE 2C).

#### CQ2-8: Is antimicrobial dosage adjusted using therapeutic drug monitoring (TDM) for sepsis?

*Answer*: We suggest antimicrobial administration using TDM for sepsis (GRADE 2D).

#### CQ 2–9: Is de-escalation based on culture and susceptibility results performed in antimicrobial therapy for sepsis?

*Answer*: We suggest applying de-escalation based on culture and susceptibility results performed in antimicrobial therapy for sepsis (GRADE 2C).

#### CQ2-10: In patients with sepsis receiving empiric antifungal drugs, are antifungal drugs discontinued using β-D glucan as an indicator?

*Answer*: We suggest the use of β-D glucan as an indicator for the discontinuation of antifungal drugs in patients with sepsis who have been administered empiric antifungal drugs (GRADE 2C).

#### CQ2-11: Is PCT used as an indicator for discontinuing antimicrobial therapy for sepsis?

*Answer*: We suggest the use of PCT as an indicator for discontinuing antimicrobial therapy for sepsis (GRADE 2A).

#### CQ2-12: Is short-term (≤ 7 days) antimicrobial therapy used for sepsis?

*Answer*: We suggest applying short-term (≤ 7 days) antimicrobial therapy for sepsis (GRADE 2C).

### CQ3 Initial resuscitation

#### CQ3-1: What parameters are used to assess tissue hypoperfusion in initial resuscitation for sepsis?

*Answer*: The measurement of blood lactate level is commonly performed, and the usefulness of capillary refill time (CRT) has also been reported to assess tissue hypoperfusion during initial resuscitation for sepsis (*Provision of information for background question*).

#### CQ3-2: Are cardiac function and preload evaluated using echocardiography in initial resuscitation for sepsis?

*Answer*: Cardiac function and preload are evaluated using echocardiography while performing initial resuscitation for sepsis (*Good Practice Statement*).

#### CQ3-3: What is the target mean arterial pressure (MAP) during initial resuscitation for sepsis?

*Answer*: We suggest 65 mmHg as the target MAP during initial resuscitation for sepsis (GRADE 2C).

#### CQ3-4: Which fluid is used for initial resuscitation of sepsis?

*Answer*: During initial resuscitation for sepsis, we suggest the administration of balanced crystalloid over normal saline (GRADE 2C).

We suggest the administration of isotonic albumin preparations (4–5%) when a patient with sepsis does not respond to standard treatment using crystalloids and requires a large volume of crystalloids (GRADE 2B).

During initial resuscitation for sepsis, we recommend against the administration of synthetic colloids (GRADE 1B).

#### CQ3-5: How is initial fluid therapy given for patients with sepsis?

*Answer*: Initial fluids for septic patients with reduced intravascular volume are aimed at optimizing circulating blood volume, and some patients require the administration of at least 30 mL/kg of crystalloid solutions within 3 h. However, there has been caution for harm caused by excessive fluid administration (*Provision of information for background question*).

#### CQ3-6: Is early administration of vasopressor performed during initial resuscitation for sepsis?

*Answer*: During initial resuscitation for sepsis with hypotension, we suggest early administration of vasopressor combined with resuscitative fluid therapy (GRADE 2C).

#### CQ3-7: Which vasopressor is used as the first-line and second-line drugs in patients with septic shock?

*Answer*: We suggest using noradrenaline as the first-line vasopressor for septic shock (GRADE 2D), and vasopressin as the second-line vasopressor for septic shock (GRADE 2A).

#### CQ3-8: Are steroids administered for septic shock?

*Answer*: We suggest administering low-dose hydrocortisone (200–300 mg/day) to patients with septic shock unresponsive to initial fluid resuscitation and vasopressors for the purpose of recovering from shock (GRADE 2C).

#### CQ3-9: What is the threshold of hemoglobin level for transfusion in initial resuscitation for septic shock?

*Answer*: We suggest a hemoglobin level of 7 g/dL as a threshold for transfusion in initial resuscitation for septic shock (GRADE 2C).

#### CQ3-10: Are β1-adrenoceptor antagonists used for septic patients with persistent tachycardia after initial resuscitation?

*Answer*: We suggest administering β1-adrenoceptor antagonists for patients with sepsis to manage persistent tachycardia after initial resuscitation (GRADE 2C).

#### CQ3-11: Is sodium bicarbonate intravenously administered for septic patients with severe metabolic acidosis (pH ≤ 7.2)?

*Answer*: We suggest the intravenous administration of sodium bicarbonate for septic patients with severe metabolic acidosis (pH ≤ 7.2) (GRADE 2C).

#### CQ3-12: What is the indication for mechanical circulatory support for septic shock?

*Answer*: There has been insufficient evidence for the effects of mechanical circulatory supports, such as veno-arterial extracorporeal membrane oxygenation (V-A ECMO), intra-aortic balloon pumping, and intracardiac pump catheter (Impella®, Abiomed) for cardiac dysfunction in septic shock, and their indications have not been established (*Provision of information for background question*).

#### CQ3-13: Is restrictive fluid management provided in septic patients with stable hemodynamics?

*Answer*: We suggest providing restrictive fluid management in septic patients with stable hemodynamics with monitoring for ischemic organ dysfunction due to hypoperfusion (GRADE 2C).

*Remarks*: Hypoperfusion can be comprehensively evaluated using skin findings (such as mottling and peripheral cyanosis), vital signs, capillary refill time, lactate levels, or urinary output.

### CQ4 Blood purification

#### CQ4-1: Is polymyxin B-immobilized fiber column (PMX-DHP) used for patients with septic shock?

*Answer*: We suggest against using PMX-DHP for patients with septic shock (GRADE 2D).

#### CQ4-2: Is early renal replacement therapy (RRT) performed for septic acute kidney injury (AKI)?

*Answer*: We suggest against performing early RRT for patients with septic AKI (GRADE 2C).

#### CQ4-3: Is continuous RRT provided for septic AKI?

*Answer*: Either continuous or intermittent RRT can be selected as an RRT modality for septic AKI (GRADE 2D).

However, continuous RRT is used for hemodynamically unstable patients (*Good Practice Statement*).

#### CQ4-4: Is treatment dose increased in RRT for septic AKI?

*Answer*: We recommend against increasing the RRT dose beyond the international standard dose (20–25 mL/kg/h) for patients with septic AKI (GRADE 1A).

### CQ5 Disseminated intravascular coagulation

#### CQ5-1: What is the diagnostic method for sepsis-induced disseminated intravascular coagulation (DIC)?

*Answer*: Several diagnostic criteria for DIC in patients with sepsis have been proposed. The Japanese Association for Acute Medicine DIC (JAAM-DIC) and the sepsis-induced coagulopathy (SIC) diagnostic criteria are used to diagnose early DIC and to determine treatment initiation. The International Society on Thrombosis and Hemostasis (ISTH) overt DIC diagnostic criteria are used to diagnose progressed DIC and predict mortality (*Provision of information for background question*).

#### CQ5-2: What are the differential diagnoses for patients with suspected sepsis-induced DIC?

*Answer*: DIC-like clinical conditions include thrombotic microangiopathy (TMA) and heparin-induced thrombocytopenia (HIT), which require differential diagnosis (*Provision of information for background question*).

#### CQ5-3: Is antithrombin administered for sepsis-induced DIC?

*Answer*: We suggest the administration of antithrombin for sepsis-induced DIC (GRADE 2B).

#### CQ5-4: Is recombinant thrombomodulin administered for sepsis-induced DIC?

*Answer*: We suggest the administration of recombinant thrombomodulin for sepsis-induced DIC (GRADE 2B).

### CQ6 Adjuvant therapy

#### CQ6-1: Is intravenous immunoglobulin (IVIG) administered for sepsis?

*Answer*: We suggest against the administration of IVIG for sepsis (GRADE 2C).

#### CQ6-2: Is high-dose vitamin C therapy used for sepsis?

*Answer*: We suggest against the use of high-dose vitamin C therapy for sepsis (GRADE 2B).

#### CQ6-3: What is the target blood glucose level for sepsis?

*Answer*: We suggest 144–180 mg/dL as a target blood glucose level for sepsis (GRADE 2C).

#### CQ6-4: Is antipyretic therapy provided to febrile patients with sepsis?

*Answer*: We suggest against antipyretic therapy for febrile patients with sepsis (GRADE 2C).

#### CQ6-5: Is stress ulcer prophylaxis performed for patients with sepsis to prevent gastrointestinal hemorrhage?

*Answer*: We suggest performing stress ulcer prophylaxis for patients with sepsis to prevent gastrointestinal bleeding (GRADE 2D).

#### CQ6-6: How is the body temperature managed in septic patients with hypothermia?

*Answer*: Rewarming therapy might be rational when hypothermia-associated circulatory disorders or coagulation abnormalities are observed in septic patients with hypothermia (core body temperature of < 35 °C). However, caution should be taken as rewarming therapy may cause peripheral vasodilation, resulting in adverse events, such as hypotension (*Provision of information for background question*).

#### CQ6-7: How is tracheal intubation performed for patients with sepsis?

*Answer*: Pathophysiological conditions for which tracheal intubation is indicated in patients with sepsis include shock and imbalance between oxygen demand and supply, in addition to airway obstruction and hypoxemia. Because sedatives and analgesics used during tracheal intubation may cause hemodynamic fluctuations, it is important to perform appropriate hemodynamic management, such as preparation of vasopressors (*Provision of information for background question*).

### CQ7 Post-intensive care syndrome

#### CQ7-1: Is early rehabilitation implemented to prevent post-intensive care syndrome (PICS)?

*Answer*: We suggest conducting early rehabilitation to prevent PICS (GRADE 2D).

#### CQ 7–2: Is neuromuscular electrical stimulation used to prevent ICU-acquired weakness (ICU-AW)?

*Answer*: We suggest using neuromuscular electrical stimulation to prevent ICU-AW (GRADE 2C).

#### CQ7-3: Is follow-up after ICU discharge be implemented to improve physical, cognitive, and mental functions?

*Answer*: We suggest conducting follow-up after ICU discharge to improve physical, cognitive, and mental functions (GRADE 2D).

#### CQ7-4: Is rehabilitation after hospital discharge implemented to improve physical, cognitive, and mental functions?

*Answer*: We suggest performing rehabilitation after hospital discharge to improve physical, cognitive, and mental functions (GRADE 2C).

### CQ8 Patient and family care

#### CQ 8–1: Is written information provided to the families of critically ill patients?

*Answer*: We suggest providing information related to intensive care to the families of critically ill patients in written or other forms (GRADE 2C).

#### CQ 8–2: What is the relaxation of visitation restrictions for families of critically ill patients?

*Answer*: Relaxation of visitation restrictions for families of critically ill patients include unrestricted visiting hours or numbers of visitors and online visitation. There is an opinion that it may be effective in preventing post-intensive care syndrome family (PICS-F). Its necessity should be considered depending on the situation at one's own facility and individual cases (*Provision of information for background question*).

#### CQ 8–3: What are the methods for supporting decision-making that respect the value systems and ways of thinking in a patient?

*Answer*: There are methods of supporting decision-making that respect the values systems and ways of thinking of a patient through repeated discussions at multidisciplinary conferences involving patients and their families. One of the methods proposed is careful estimation through surrogate decision makers (e.g., family members) when the intentions of a patients are unclear. While respecting the intentions of patients, appropriate medical information is provided to patients and their families (*Provision of information for background question*).

#### CQ 8–4: Is an ICU diary kept for critically ill patients?

*Answer*: We suggest keeping an ICU diary for critically ill patients (GRADE 2C).

#### CQ 8–5: Is follow-up after ICU discharge provided to families of critically ill patients to improve their mental health?

*Answer*: In facilities with well-established systems, we suggest providing follow-ups, such as face-to-face, phone, and online interviews after ICU discharge, to families of critically ill patients to improve their mental health (GRADE 2C).

### CQ9 Pediatrics

#### CQ 9–1: How are empiric antimicrobials selected for pediatric septic shock?

*Answer*: Antimicrobials for all possible microorganisms are selected, taking into account the organ of infection, setting (community, hospital, or ICU), and patient background (e.g., immune status and antimicrobial prescription history) (*Provision of information for background question*).

#### CQ 9–2: How is initial fluid therapy administered for pediatric sepsis?

*Answer*: Methods of administering initial fluid therapy to pediatric sepsis include repeated administration of balanced crystalloid solutions, as a 10–20 mL/kg bolus, while evaluating response to therapy. Clinical findings suggestive of fluid overload or poor response to fluid administration can serve as discontinuing fluid therapy. In particular, attention is paid to the amount and rate of bolus administration in patients complicated by heart failure. We cannot provide information regarding the speed of fluid administration or upper limit of total fluid volume (*Provision of information for background question*).

#### CQ 9–3: How are vasopressors selected for pediatric patients with septic shock?

*Answer*: Adrenaline or noradrenaline is used as vasopressors in pediatric patients with septic shock, according to physical findings, hemodynamic parameters, and echocardiographic findings (*Provision of information for background question*).

#### CQ 9–4: What is the route of administering vasopressors for pediatric sepsis?

*Answer*: Vasopressors are generally administered via the central venous line, as they may cause tissue injury when extravasation occurs. However, vasopressors are administered via a peripheral venous line or intraosseous access at appropriate concentrations for short periods to avoid delays in initiating the administration (*Provision of information for background question*).

#### CQ 9–5: Are steroids administered to pediatric patients with septic shock who are unresponsive to initial fluid therapy and vasopressors?

*Answer*: We suggest against routine administration of steroids for pediatric patients with septic shock who are unresponsive to initial fluid therapy and vasopressors (GRADE 2D).

#### CQ 9–6: What is the optimal hemoglobin level for blood transfusion in pediatric patients with sepsis who have stable hemodynamics?

*Answer*: We suggest transfusing at a hemoglobin level of 7.0 g/dL in hemodynamically stable pediatric patients with sepsis (GRADE 2C).

#### CQ 9–7: Is strict blood glucose control performed for pediatric sepsis?

*Answer*: We suggest against strict blood glucose control for pediatric sepsis (GRADE 2C).

#### CQ 9–8: What are treatment and support policies centered on critically ill pediatric patients?

*Answer*: It is necessary to support the decision-making that prioritizes the benefits of affected children and respects the values and wishes of the affected children and their families.

A multidisciplinary team has a role in providing appropriate medical information. Actively creating an environment that allows family members to participate in care and support the decision-making process is essential, especially in pediatric patients (*Provision of information for background question*).

## Quick reference list of FRQs

FRQ1-1: Do artificial intelligence (AI)-based detection systems for sepsis in the ER and ICU improve prognosis compared to conventional detection systems?

FRQ1-2: Is a tele-ICU system useful for managing patients with sepsis?

FRQ3-1: Is hypertonic albumin solutions (20–25%) used as an initial fluid for septic shock?

FRQ3-2: Is adrenaline added when patients with septic shock have difficulty in maintaining hemodynamics with concomitant use of noradrenaline and vasopressin?

FRQ3-3: Are inotropes used for septic shock patients with decreased cardiac function and tissue hypoperfusion?

FRQ3-4: Is the serum albumin level maintained at 3.0 g/dL using hypertonic albumin solutions (20–25%) after initial resuscitation for septic shock?

FRQ3-5: What is the threshold of hemoglobin levels for transfusion in patients with sepsis who have stable hemodynamics?

FRQ5-1: Are antithrombin and thrombomodulin concomitantly administered for sepsis-induced DIC?

FRQ5-2: Is heparin or heparin analogs administered for sepsis-induced DIC?

FRQ6-1. Is IVIG administered for patients with streptococcal toxic shock syndrome (STSS)?

FRQ7-1: Is the ABCDEFGH bundle implemented to prevent PICS?

FRQ9-1: Is IVIG administered for pediatric sepsis?

## CQ1 Diagnosis and source control

See Fig. 1.

**Fig. 1:**
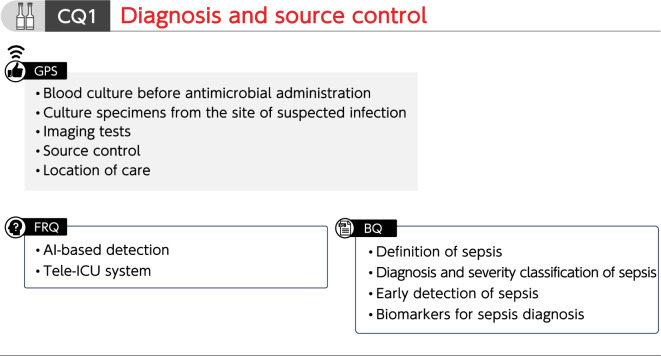
Summary of recommendations (CQ1 Diagnosis and source control). *BQ* background question, *CQ* clinical question, *FRQ* future research question, *GPS* good practice statement, *ICU* intensive care unit

### CQ1-1: Definition of sepsis

*Answer*: Sepsis is defined as a “life-threatening organ dysfunction caused by a dysregulated host response to infection” (*Provision of information for background question*).

#### Rationale

The concept of systemic inflammatory response syndrome (SIRS) was proposed in 1992, in which sepsis was defined as SIRS due to infection (sepsis-1) [4]. Such a definition was revised with the aim of creating a definition that better reflects the pathophysiology of sepsis (sepsis-2) [5]. However, sepsis-2 had no difference in sensitivity or specificity in sepsis diagnosis compared to sepsis-1, and it did not replace the simple, easy-to-use sepsis-1 definition [6].

A limitation of the sepsis-1 definition was its low specificity in predicting the progression of organ dysfunction and mortality, despite its high sensitivity [7]. Furthermore, the pathophysiology of sepsis has come to be understood not only as systemic inflammation but also as a complex host response to infection and associated organ dysfunction. From this perspective, the definition of sepsis was revised in the “Third International Consensus Definitions for Sepsis and Septic Shock (sepsis-3)” in 2016 [1]. The sepsis-3 was a “life-threatening organ dysfunction caused by a dysregulated host response to infection.” Additionally, septic shock was defined as a subset of sepsis in which the underlying circulatory and cellular/metabolic abnormalities profoundly increase the risk of mortality. In the present guidelines, sepsis is defined according to the sepsis-3 definition, as in the J-SSCG 2020 [8, 9].

### CQ1-2: Diagnosis and severity classification of sepsis

*Answer*: Sepsis is diagnosed when there is an acute increase in the SOFA score of ≥ 2 points in the presence of a confirmed or suspected infection. Additionally, septic shock is diagnosed in patients with sepsis when a patient requires vasopressors to maintain a mean arterial pressure of ≥ 65 mmHg and has a blood lactate level > 2 mmol/L (18 mg/dL) despite adequate fluid resuscitation (*Provision of information for background question*).

#### Rationale

In sepsis-3, the progression of infection-induced organ dysfunction is positioned as an important treatment target [1], and sepsis diagnostic criteria using the SOFA score [10] has been proposed. In the present guidelines, we adopted the sepsis-3 definition for sepsis and septic shock.

In an ICU, changes in SOFA score are evaluated in patients with confirmed or suspected infections. An acute increase in a SOFA score of ≥ 2 points is considered the progression of serious organ dysfunction, resulting in a definitive diagnosis of sepsis.

In contrast, SOFA scores may not be easily evaluated outside the ICU. Thus, sepsis-3 proposed sepsis screening using qSOFA [1]. However, due to its low sensitivity for sepsis and hospital mortality, the usefulness of qSOFA as a screening tool is questionable [11–13]. Furthermore, when sepsis is suspected using qSOFA, SOFA score is evaluated to determine sepsis.

Septic shock is the most severe form of sepsis. Sepsis-3 defines septic shock as a condition in which a patient cannot maintain blood pressure with fluid resuscitation alone, requiring vasopressors, such as noradrenaline, and has a blood lactate level of > 2 mmol/L (18 mg/dL).

Several issues have been pointed out regarding the sepsis-3 diagnostic criteria for sepsis and septic shock, including the following: (1) due to the low sensitivity of qSOFA for sepsis, there are concerns about screening using qSOFA alone; (2) revision of the SOFA score (revision to SOFA 2.0) is desired worldwide due to its non-uniformness, lack of reproducibility, and inability to be used for evaluating new treatments [14]; (3) the criteria for suspecting infection are unclear [15]; (4) there is a problem of routine measurement of lactate levels; and (5) prompt diagnosis and initiation of treatment are not always integrated.

### CQ1-3: What methods are there for early detection of sepsis in general wards and ER?

*Answer*: Methods for early detection of sepsis in general wards and ER include screening tools, such as qSOFA and early warning scores (*Provision of information for background question*).

#### Rationale

Early detection of sepsis is important. However, it is challenging to distinguish patients with sepsis from those with other infectious diseases because the pathophysiology is not significantly different. Therefore, screening criteria have been developed focusing on the detection of patients with infectious diseases who have a high risk of mortality and require advanced medical care. Scoring systems, such as SIRS [4, 16], qSOFA [17], and National Early Warning Score (NEWS) [18] have been evaluated in adult patients. Those results suggest that there should be caution when using them independently, and the characteristics and limitations should be well understood. A meta-analysis of 26 studies comparing the mortality prediction ability of SIRS, qSOFA, and NEWS in patients with sepsis showed that SIRS had a high sensitivity (82%) and low specificity (24%), qSOFA had a low sensitivity (46%) and high specificity (82%), and NEWS had a moderate sensitivity (73%) and moderate specificity (52%) [19]. For pediatric patients, pediatric early warning score (PEWS) was evaluated as a tool for early detection of status deterioration. A multicenter cluster RCT reported that the use of PEWS reduced the incidence of clinical deterioration events [20]. Additionally, qSOFA has been evaluated in an observational study of pediatric patients suspected of bacterial infections who visited the ER, which reported that an age-adjusted qSOFA had a moderate predictive performance for pediatric ICU admission and mortality (area under the receiver operating curve [AUROC] 0.72). [21]

### CQ1-4: When and how are blood culture samples collected for patients suspected with sepsis?

*Answer*: At least two sets of blood culture samples are collected before antimicrobial administration for patients suspected with sepsis (*Good Practice Statement*).

#### Rationale

In the treatment of sepsis, identifying the causative pathogen is crucial for appropriate antimicrobial therapy. It is reported that 38–69% of patients with sepsis develop bacteremia [22, 23]. Therefore, blood cultures should be collected before antimicrobial administration while paying attention not to delay the start of antimicrobial therapy. This is important because the rate of detecting pathogens decreases after antimicrobial administration, increasing the possibility of not identifying pathogens. Even if antimicrobials have already been administered for conditions like postoperative infection in hospitalized patients, or other reasons, samples for blood culture should be collected before the administration of new antimicrobials. A study reported that microorganisms are detected in approximately 20% of blood culture samples collected after antimicrobial administration [24].

Regarding the volume of blood for cultures, a sampling volume of 20 mL per set is recommended. Collecting only one set of blood culture results in a low detection rate and difficulty in evaluating contamination. Hence, it is desirable to collect at least two sets of blood cultures, or three sets if possible [25, 26].

Appropriate skin disinfection before the collection of blood culture samples is also important. It is unclear which disinfectant is optimal among 1% chlorhexidine gluconate, povidone-iodine, and 70% alcohol; however, it has been reported that the use of alcohol-containing disinfectants reduces contamination more effectively compared to non-alcohol-containing preparations [27]. Adherence to accurate aseptic techniques to minimize contamination is important.

### CQ1-5: When and how are culture specimens other than blood culture samples collected for patients suspected with sepsis?

*Answer*: Culture specimens are collected from the site of suspected infection before antimicrobial administration for patients suspected with sepsis (*Good Practice Statement*).

#### Rationale

Blood cultures are the standard method for identifying pathogens in sepsis. However, blood cultures do not have a high positive rate, depending on the situation and source of infection [22, 23]. Therefore, we recommend collecting culture specimens other than bloods from the site of suspected infection, based on clinical findings, preferably before the start of antimicrobials.

If pneumonia is suspected, cultures of lower respiratory tract specimens can aid its diagnosis. This is particularly considered for patients with severe pneumonia or those at risk of Methicillin-resistant *Staphylococcus aureus* or *Pseudomonas aeruginosa* infections [28]. For ventilator-associated pneumonia, there is no consensus on whether to use endotracheal aspirate (via blind tracheal suctioning) or bronchoalveolar lavage fluid as a culture specimen. Respiratory symptoms and parameters of patients and the availability of microbiology laboratory at each facility are considered before sampling [29, 30].

When a urinary tract infection is suspected, a urine culture should be obtained before antimicrobial administration to identify the causative bacteria and determine its drug susceptibility. Asymptomatic bacteriuria may occur in older adults and patients with an indwelling urinary catheter. Therefore, antimicrobial therapy should be performed considering physical findings, as well as the results of urinary sediment or blood culture tests.

When bacterial meningitis is suspected, cerebrospinal fluid should be collected before antimicrobial administration if the patient is not contraindicated for a lumbar puncture and has no suspicion of cerebral hernia based on brain computed tomography (CT) or clinical findings. Because delay in antimicrobial administration increases mortality, antimicrobial administration should be prioritized if cerebrospinal fluid collection requires time [31]. Cerebrospinal fluid cultures have a positive rate of 70–80% in untreated patients and ≤ 50% in patients who have received antimicrobial treatment [32]. Thus, collecting blood cultures before administering antimicrobials can aid in microbial diagnosis when antimicrobials are administered prior to cerebrospinal fluid testing. The positivity of blood cultures was reported to be 75% in patients with community-acquired pneumococcal meningitis [33].

### CQ1-6: What are the roles of CRP, PCT, P-SEP, and IL-6 as biomarkers for sepsis diagnosis?

*Answer*: CRP, PCT, P-SEP, or IL-6 alone has not been shown to have high diagnostic accuracy for sepsis in general wards, ER, or ICU. Therefore, the diagnosis of sepsis using any specific biomarker is generally considered difficult. The biomarkers are used as supplementary indicators in addition to observation of general conditions (*Provision of information for background question*).

#### Rationale

Clinical diagnosis of sepsis can often be challenging, and a variety of biomarkers are referenced for this purpose. There are four commonly referenced sepsis biomarkers (CRP, PCT, P-SEP, and IL-6), on which many observational studies have been reported. According to the results from meta-analyses, CRP had a sensitivity of 0.75–0.80, specificity of 0.61–0.67, and AUROC of 0.73–0.77 [34, 35], PCT had a sensitivity of 0.79–0.80, specificity of 0.77–0.78, and AUROC of 0.85 [34, 35], P-SEP had a sensitivity of 0.84, specificity of 0.73–0.76, and AUROC of 0.87–0.88 [36, 37], and IL-6 had a sensitivity 0.68–0.72, specificity of 0.73–0.73, and AUROC of 0.79–0.80 [35, 38].

Although the reported diagnostic accuracies vary among the biomarkers, none has demonstrated sufficient accuracy to make a diagnosis when used alone. Sepsis is a highly heterogeneous clinical condition depending on the infected organ or underlying disease. In general wards, ER, and ICU, the diagnosis of sepsis using any specific biomarker is generally considered difficult. The biomarkers are used as supplementary indicators in addition to observation of general conditions.

### CQ1-7: Are imaging tests performed to identify the source of infection in patients suspected of having sepsis?

*Answer*: Appropriate imaging tests are conducted according to the suspected disease in patients suspected with sepsis (*Good Practice Statement*).

#### Rationale

In patients suspected of having sepsis, it is important to evaluate whether there is a source of infection that needs to be controlled. For this purpose, imaging tests, such as ultrasonography, X-ray, CT, and magnetic resonance imaging (MRI) tests are utilized. The most prioritized test should be selected, depending on the suspected infection site. The risk of radiation exposure, as well as the risks associated with the use of a contrast agent, needs to be considered. If a patient has unstable hemodynamics, attention also needs to be paid to any sudden changes in their condition during transportation to an imaging facility.

Table 3 shows common imaging tests according to the source of infection. Contrast-enhanced CT and MRI are used for brain abscess [39]. Ultrasonography and contrast-enhanced CT are used for cervical abscess [40]. A contrast-enhanced CT, chest X-ray, and ultrasonography are used for empyema [41, 42]. Ultrasonography is the first choice for infectious endocarditis [43]; however, cardiac CT and positron emission computed tomography with [18] F-fluorodeoxyglucose are also used at facilities where the testing is available. Ultrasonography is used for acute abdomen [44], cholangitis/cholecystitis [45], and obstructive urinary tract infection [46], and CT is used in patients whose diagnosis is difficult using ultrasonography. Magnetic resonance imaging and magnetic resonance cholangiopancreatography are applied when a diagnosis cannot be made using CT, despite suspected cholangitis or cholecystitis [45]. For necrotizing soft tissue infection, CT and MRI are applied [47]; however, direct observation of the subcutaneous tissue and fascia through surgical procedures is the most important.

**Table 3:** Common imaging tests according to the source of infection

Region	Suspected source of infection	Primary imaging tests			
		Ultrasonography	X-ray	CT	MRI
Head and neck	Brain abscess			〇 (Contrast-enhanced imaging)	〇
	Cervical abscess	〇		〇 (Contrast-enhanced imaging)	
Chest	Empyema	〇	〇	〇 (Contrast-enhanced imaging)	
	Infective endocarditis	〇^a^		CT (Cardiac CT/^18^F-FDG PET/CT)	
Abdomen	Peritonitis	〇		〇^b^	
	Cholecystitis/cholangitis	〇		〇 (Contrast-enhanced imaging)	〇
	Obstructive urinary tract infection	〇		〇	
Other	Necrotic soft tissue infections			〇	〇

Circles indicate appropriate primary imaging tests

^*18*^*F-FDG PET/CT* positron emission computed tomography with ^18^F-fluorodeoxyglucose, *CT* computed tomography, *MRI* magnetic resonance imaging

^a^Transesophageal echocardiography is indicated if clinically suspected or in patients with prosthetic valves or other implanted devices. [43]

^b^Contrast-enhanced imaging is recommended for the evaluation of organ ischemia, vascular lesions, and acute pancreatitis. [44]

### CQ1-8: When is the source of infection controlled in patients with sepsis?

*Answer*: The source of infection is controlled as soon as possible after recognition of sepsis (*Good Practice Statement*).

#### Rationale

Appropriate control of infection source is important in the treatment of sepsis and septic shock. As the source of infection is identified, it is promptly controlled after assessing its benefits and complications [48, 49], especially when the infection is unlikely to improve with conventional antimicrobial therapy alone. Even when a patient has a poor general condition due to sepsis or septic shock, control of the infection source is considered if its benefits are judged to outweigh the disadvantages [50]. Exceptionally, for patients with infected pancreatic necrosis, endoscopic or percutaneous drainage is applied when encapsulation is expected (usually after 4 weeks of onset), and if their general condition is maintained with conservative treatment [51].

In patients with acute pyelonephritis due to urinary tract obstruction, the source of infection is promptly controlled using transurethral stent placement or percutaneous nephrostomy [52]. Timely surgical debridement procedures are important to manage patients with necrotizing soft tissue infection. A meta-analysis of observational studies showed that an early debridement (within 12 h of hospital admission) was associated with reduced mortality [53]. In patients with sepsis suspected of having a catheter-related bloodstream infection, prompt catheter removal is a protective factor of hospital mortality [54]. Empyema is another clinical condition that requires control of the infection source, for which open or percutaneous thoracic drainage is performed. [55, 56]

### CQ1-9: Which facility is appropriate for managing patients with sepsis who are unresponsive to initial fluid resuscitation?

*Answer*: Patients with sepsis who are unresponsive to initial fluid resuscitation are managed in a facility capable of providing intensive care (*Good Practice Statement*).

#### Rationale

Sepsis is a very common clinical condition that can be encountered in any clinical department or medical facility, and its treatment involves a variety of healthcare providers. Patients with sepsis, or those suspected to have sepsis, are occasionally treated in general wards. However, it should be noted that patient outcomes may deteriorate in situations where sufficient medical resources cannot be provided. Therefore, it is critical to evaluate the severity of each patient and select an appropriate setting for care.

The criterion of “sepsis that is unresponsive to initial fluid resuscitation” includes not only septic shock but also persistent hypotension, prolonged disturbance of consciousness, deteriorated respiratory conditions, and poor lactate clearance. The place of treatment should be decided, considering not only the severity but also the required medical resources, prospects for recovery, and patient’s preferences.

Japanese nationwide database studies have suggested that ICU admission may be associated with a decreased mortality rate of patients with sepsis [57, 58]. An observational study has suggested that treating patients with sepsis in a closed ICU is associated with a decreased hospital mortality rate compared to an open ICU [59]. In pediatric sepsis management, various algorithms have indicated that mechanical ventilation and vasopressors should be started when a patient is determined to be unresponsive to initial fluid resuscitation [60, 61]. Therefore, it would be appropriate to make a decision to transition to intensive care management if the patient is “unresponsive to initial fluid resuscitation,” and to transfer the patient to a hospital bed capable of providing intensive care or to a nearby facility skilled in pediatric critical care.

### FRQ1-1: Do AI-based detection systems for sepsis in the ER and ICU improve prognosis compared to conventional detection systems?

#### Rationale

Management of sepsis is time-sensitive, and early prediction of sepsis is highly important to reduce mortality. In recent years, AI algorithms have been developed to enable early detection of sepsis with high accuracy, and their usefulness has been investigated.

A systematic review and meta-analysis of diagnostic performance using the Quality Assessment of Diagnostic Accuracy Studies checklist reported that the accuracy of AI-based sepsis diagnosis had an AUROC of 0.68–0.99 for ICU, 0.96–0.98 for in-hospital, and 0.87–0.97 for ER [62]. We performed a systematic review and found only one RCT that assessed the efficacy of AI algorithms. This RCT was conducted at an ICU using a machine learning workflow called “InSight” [63]. The mean length of hospital stay was shorter in an intervention group that used InSight (10.3 days) than that in a control group that did not use InSight (13.0 days). Additionally, hospital mortality, which was a secondary endpoint, was lower in the intervention group (9.0%) than that in the control group (21.3%). However, in Japan, InSight has not received the Software as a Medical Device certification as a programmable medical device or undergone any pilot studies. Additionally, early prediction of sepsis using AI may lead to increased use of unnecessary antimicrobials [64] or the occurrence of unknown adverse events. Further studies are needed to evaluate AI-based sepsis detection systems in the future.

### FRQ1-2: Is a tele-ICU system useful for managing patients with sepsis?

#### Rationale

Appropriate and prompt treatment is necessary to improve the prognosis of sepsis. However, due to the limited number of specialist physicians, such as intensive care physicians, not all facilities have specialist physicians with enough experiences and knowledge to treat sepsis. “Tele-ICU,” which is a medical support system using video/voice calls and computer system networks, is expected to cover the shortage of specialist physicians and ensure standardization of the quality of care.

A systematic review published in 2023 showed that the use of tele-ICU supports may be beneficial in sepsis treatment, particularly in settings where a control group has a low survival rate, and that its effectiveness depends on various hospital-level factors, such as the quality of medical care provided at baseline [65]. However, to date, there have been no high-quality studies evaluating the effectiveness of tele-ICU in the prognosis of patients with sepsis. Future studies are needed to accumulate evidence on the effectiveness of tele-ICU supports in the treatment of patients with sepsis.

## CQ2 Antimicrobial therapy

See Fig. 2.

**Fig. 2:**
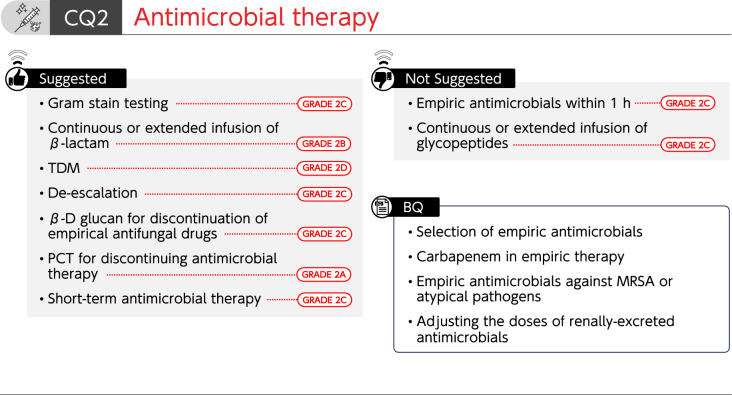
Summary of recommendations (CQ2 Antimicrobial therapy). *BQ* background question, *CQ* clinical question, *MRSA* methicillin-resistant *Staphylococcus aureus,* *TDM*, therapeutic drug monitoring

### CQ2-1: Is Gram stain testing useful for selecting empiric antimicrobials for sepsis?

*Answer*: We suggest using Gram stain testing for selecting empiric antimicrobials for sepsis (GRADE 2C).

#### Rationale

Although drug-resistant bacteria are spreading and becoming more prevalent worldwide, the development of new antimicrobials is on the decline [66, 67]. In 2015, the World Health Organization adopted the Global Action Plan, which emphasized the need for appropriate use of broad-spectrum antimicrobials [68]. However, no method of safely limiting the use of broad-spectrum antimicrobials has been established. In recent years, there have also been reports of an association between excessive exposure to broad-spectrum antimicrobials and increased mortality rate [69, 70]. Gram stain testing classifies the morphological characteristics of bacteria within minutes, and its results may serve as indicators for the appropriate selection of empiric antimicrobials.

We identified a multicenter RCT (206 patients) [71]. As a result of Gram staining-based antimicrobial therapy, a 28-day mortality yielded a risk difference (RD) of 38 fewer per 1000 (95% confidence interval [CI] 103 fewer to 84 more); clinical response rate yielded an RD of 50 more per 1000 (95% CI 65 fewer to 180 more); the use of anti-methicillin-resistant *Staphylococcus aureus* (MRSA) drugs yielded an RD of 390 fewer per 1000 (95% CI 470 fewer to 280 fewer); and the use of antimicrobials having anti-*Pseudomonas aeruginosa* activity yielded an RD of 300 fewer per 1000 (95% CI 380 fewer to 200 fewer). However, the selection of antimicrobials having antibacterial activity against causative bacteria yielded an RD of 55 fewer per 1000 (95% CI 138 fewer to 28 more). Based on these findings, we concluded that the balance of effects was probably better for the intervention (Additional file 3).

Selection of antimicrobials based on Gram staining results requires healthcare providers with the capability of classification by morphological characteristics of bacteria, as well as knowledge of the antimicrobial spectrum. Therefore, it should be noted that its feasibility varies from hospital to hospital.

### CQ2-2: Is the administration of empiric antimicrobials for sepsis started within 1 h after diagnosing sepsis?

*Answer*: Although antimicrobials should be started as soon as possible after sepsis or septic shock is diagnosed, we suggest against the use of < 1 h target time (GRADE 2C).

#### Rationale

The Surviving Sepsis Campaign Guidelines 2021 (SSCG 2021) recommended administering antimicrobials immediately, ideally within 1 h of recognition [72]. However, adhering to the time frame of antimicrobial-administration target of within 1 h may lead to an increase in unnecessary and excessive administration of broad-spectrum and multiple antimicrobials [73]. The J-SSCG 2020 [8, 9] suggested that antibacterial drugs are administered as soon as possible upon identification of sepsis or septic shock, but against using the target time of < 1 h (GRADE 2C: certainty of evidence = “low”). Although immediate administration of antimicrobials is recommended, mandating a 1 h timeframe is controversial.

We conducted a meta-analysis of 11 published observational studies [74–84]. Administering antimicrobials within 1 h, hospital mortality yielded an RD of 22 fewer per 1000 (95% CI 57 fewer to 16 more). The studies included in the meta-analysis did not evaluate the undesirable effects of the intervention. The desirable effects of antimicrobial administration within 1 h were small, and the undesirable effects of the intervention could not be evaluated. These suggest that the balance of effects was neither intervention nor comparator was superior (Additional file 3).

Although we suggest against using the target time of < 1 h for sepsis, the suggestion does not contradict the idea of promptly administering appropriate antimicrobials that cover expected causative pathogens.

### CQ2-3: How are empiric antimicrobials selected for sepsis?

*Answer*: Empiric antimicrobials for sepsis are selected for each suspected source of infection by estimating the causative microorganism based on patient background and epidemiology. Rapid microbial diagnostic tests, tissue penetration, and the possibility of resistant bacteria are also assessed (*Provision of information for background question*). (See Additional file 1 and 2).

#### Rationale

Selection of appropriate empiric antimicrobials, along with surgical intervention for the source of infection, is a definitive treatment for sepsis, and is important in improving patient outcomes [85, 86].

According to epidemiological studies in Japan, respiratory tract, intra-abdominal, urinary tract, and soft tissue infections account for 70–90% of sepsis whose source of infection was identified [87, 88]. In addition to these sources, catheter-related bloodstream infection is considered [85–93]. In contrast, 28–49% of sepsis patients have unidentified infection foci [89–94].

External factors, such as healthcare exposure or travel history, and internal factors, such as age, sex, and underlying diseases, can also be considered for estimating causative microorganism. Community-acquired infections are often caused by microorganisms different from those causing healthcare-associated infections, and *Pseudomonas aeruginosa* does not need to be routinely covered. Exposures that serve as risk factors for healthcare-associated infections include invasive procedures, indwelling devices, and prior antimicrobial exposure.

Because the susceptibility of antimicrobials varies, depending on the location, it is important to understand local data, including antibiograms for each region and facility. Additional file 1 and 2 show a list of empirical and definitive antimicrobials that are likely to be encountered in sepsis treatment, categorized by susceptibility pattern.

### CQ2-4: Under what circumstances is carbapenem included in empiric antimicrobials for sepsis?

*Answer*: Carbapenem is included in empiric antimicrobials for sepsis when an infection is expected to be caused by a microorganism with susceptibility limited to carbapenems, such as ESBL-producing Enterobacterales, antibiotic-resistant *Pseudomonas aeruginosa*, or *Acinetobacter* spp. (*Provision of information for background question*).

#### Rationale

Carbapenems are broad-spectrum antimicrobials and often used in empiric therapy, for sepsis and septic shock. However, excessive use of carbapenems carries the risk of increasing carbapenem-resistant bacteria and elevating antimicrobial-related side effects and costs. Selective use of carbapenems in appropriate cases, rather than routine use, is desirable from the perspective of antimicrobial stewardship.

Several studies on sepsis and severe infections have shown that carbapenems and other broad-spectrum β-lactams are equally effective, suggesting a lack of superiority of routine carbapenem use in this setting [95–101]. Although a recent systematic review of 20 RCTs on hospital-acquired pneumonia, including ventilator-associated pneumonia, reported that carbapenems were superior in improving mortality (risk ratio, 0.84; 95% CI 0.74–0.96) [102], this review showed comparable clinical response rates and an increase in the incidence of resistant bacteria with the use of carbapenems. Excessive use of carbapenems may carry the risk of increasing resistant bacteria. The potential survival benefits of carbapenem use in specific situations should be balanced against the increased risk of antibiotic resistance.

Bacteria for which carbapenems have been shown to have treatment superiority include ESBL-producing Gram-negative bacilli of the Enterobacteriaceae family, and carbapenems may serve as the first-line therapy for these bacteria [103, 104]. Additionally, it is reasonable to select carbapenems for cases where the infection is expected to be caused by *Pseudomonas aeruginosa* or *Acinetobacter species* with susceptibility limited to carbapenems. However, such resistant strains are rarely found in Japan.

### CQ2-5: Under what circumstances are empiric antimicrobials against MRSA or atypical pathogens (such as Candida, viruses, Legionella, Rickettsia, and *Clostridioides difficile*) selected for sepsis?

*Answer*: Empiric antimicrobials against MRSA or atypical pathogens are selected when an infection is suspected to be caused by each of these microorganisms based on the infection focus, patient background, or microbiological findings for sepsis (*Provision of information for background question*).

#### Rationale

The use of appropriate antimicrobials is required. Antimicrobials should be carefully selected when specific bacteria (MRSA and *Clostridioides difficile*, *Legionella pneumophila*, Rickettsia), fungi, and viruses are suspected.

MRSA bacteremia is a high risk for mortality [105]. Empiric therapy with glycopeptides is reasonable when MRSA infection is strongly suspected based on the background, especially in critically ill patients. Infection with *Legionella pneumophila* can be considered in patients with pneumonia who have been exposed to contaminated water and have risk factors. If rickettsiosis is suspected based on the patient's background or clinical findings, specimens are collected, and tetracycline or quinolone are started without waiting for the test results. Risk factors for developing *Clostridioides difficile* infection include antimicrobial exposure, antacids use [106], and advanced age [107]. A study has reported that early and appropriate administration of antifungal drugs for Candida infections reduces the mortality rate [108]. Concomitant use of antifungal drugs with antibacterial agents is acceptable in patients with risk factors for Candida infection.

During the influenza epidemic/pandemic, the administration of anti-influenza drugs is considered if the patient is suspected of having respiratory failure, myocarditis, or encephalitis/encephalopathy [109]. Herpes simplex virus (HSV) type 1 is the most common pathogen of viral encephalitis, and it is an indication for empiric antiviral therapy when encephalitis is suspected [110]. In pregnant women, primary infection with HSV type 2 has a risk of leading to disseminated infection [111]. Cytomegalovirus (CMV) infection can be fatal in immunosuppressed patients. Thus, the amount of CMV in the blood is measured regularly and used as a reference for starting therapy [112]. Additionally, severe acute respiratory syndrome coronavirus 2 infection (COVID-19) should be suspected based on the epidemic/pandemic status and patient's physical findings, followed by testing.

### CQ2-6: What is used as a reference for adjusting the doses of renally-excreted antimicrobials for sepsis?

*Answer*: Renal function tests measured at multiple time points, changes in body fluids, as well as the presence of renal replacement therapy and other extracorporeal circulation, are used as references for adjusting the doses of renally-excreted antimicrobials for sepsis (*Provision of information for background question*).

#### Rationale

Approximately half of AKI in the ICU are caused by sepsis [113–117]. Dosage reduction of renally-excreted antimicrobials is particularly considered for patients with impaired renal function. Additionally, changes in body fluids and volume of distribution are observed in the early stages of sepsis.

When a patient with sepsis-induced AKI is administered renally-excreted water-soluble antimicrobial drugs or renally-excreted lipid-soluble new quinolones, dosage adjustment is performed according to renal function [118–123] (Table 4). Serum creatinine levels estimated glomerular filtration rate, and estimated creatinine clearance are commonly used as indicators of renal function. However, serum creatinine levels do not accurately reflect true renal function during the acute stage of diseases. Renal function is predicted with reference to fluctuations in serum creatinine levels measured at multiple time points [124, 125].

**Table 4:** Types of renally-excreted antimicrobials that require dose adjustment with renal dysfunction

Type of antimicrobials	Exceptions
β-Lactams	Cefoperazone, Ceftriaxone, Biapenem
Aminoglycosides	
Glycopeptides	
Polypeptides	
New quinolones	Moxifloxacin (oral administration)
Sulfamethoxazole-Trimethoprim	
Fluoropyrimidines	
Triazoles	Itraconazole, Voriconazole, Posaconazole

In contrast, dose adjustment in the early stages of sepsis is considered after understanding the following changes in body fluids [126–132]:

1. Dilution of antimicrobials in plasma and extracellular fluids due to increased Vd. Vd is increased in edema due to capillary leakage, fluid therapy, pleural effusion, body fluid drainage, and decreased protein binding rate due to hypoalbuminemia.
2. Increased cardiac output, increased renal blood flow, and increased renal clearance due to vasodilation (augmented renal clearance)

Concentrations of antimicrobials fluctuate when extracorporeal membrane oxygenation or renal replacement therapy is introduced [133–145]. In renal replacement therapy, ultrafiltration rate and concentrations measured in waste fluids can be used as references for dose adjustment [146, 147]. The doses of drugs may be adjusted based on the measured concentrations where possible. [148]

### CQ2-7: Is continuous or extended infusion of antimicrobials used for sepsis?

*Answers*: We suggest using continuous or extended infusion of β-lactam antimicrobials for sepsis (GRADE 2B).

We suggest against using continuous or extended infusion of glycopeptide antimicrobials for sepsis (GRADE 2C).

#### Rationale

##### β-lactams

Beta-lactam antimicrobials are widely used in sepsis treatment. Because β-lactams exhibit a time-dependent antibacterial effect, their continuous administration or extension of infusion time may be beneficial from the perspective of pharmacokinetics/pharmacodynamics (PK/PD). Continuous administration of β-lactam drugs and extended infusion time was suggested in the J-SSCG2020 [8, 9].

We conducted a meta-analysis of 17 RCTs [149–165]. As a result of continuous administration or extended infusion time of β-lactam drugs, the mortality yielded an RD of 53 fewer per 1000 (95% CI 96 fewer to 0), and the clinical response rate yielded an RD of 109 more per 1000 (95% CI 18 more to 214 more). Furthermore, side effects yielded an RD of 1 fewer per 1000 (95% CI 23 fewer to 31 more), and the detection of drug-resistant bacteria yielded an RD of 14 fewer per 1000 (95% CI 58 fewer to 45 more). Thus, we concluded that the balance of effects was probably better for the intervention (Data S3).

No special procedure is required for the continuous administration of antimicrobial agents or the extension of their time of administration. Although a syringe pump is required, this can be relatively performed easily in an ICU and will be well tolerated by healthcare providers. Few facilities routinely perform continuous administration of antimicrobial agents or extended their times of administration, and there may be a need to educate nurses, obtain the cooperation and monitoring of pharmacists, and in-hospital consensus prior to implementation. Furthermore, the time of usage of medical resources needed for continuous administration (e.g., infusion pumps and syringe pumps) will also likely increase.

##### Glycopeptides

Glycopeptides, such as vancomycin, are widely used for MRSA infection. Because glycopeptides, as with β-lactams, exhibit a time-dependent antibacterial effect, their continuous administration or extended infusion time is considered effective from the perspective of PK/PD. Their blood concentrations need to be kept within a safe range since the side effect of renal damage increases in proportion to the increase in blood concentrations, and there is a possibility of using continuous administration instead of intermittent administration.

We conducted a meta-analysis of three RCTs [166–168]. As a result of continuous administration of glycopeptide drugs or extended infusion time, mortality yielded an RD of 16 more per 1000 (95% CI 121 fewer to 242 more), and clinical cure yielded an RD of 24 fewer per 1000 (95% CI 154 fewer to 130 more). However, side effects yielded an RD of 49 fewer per 1000 (95% CI 107 fewer to 68 more). Considering the relative value of each outcome, we concluded that the balance of effects was probably better for the comparator (Additional file 3).

### CQ2-8: Is antimicrobial dosage adjusted using TDM for sepsis?

*Answer*: We suggest antimicrobial administration using TDM for sepsis (GRADE 2D).

#### Rationale

Since the blood concentrations of antimicrobials in patients with sepsis fluctuate due to vascular hyperpermeability or changes in renal blood flow, antimicrobial administration requires dose adjustment, and there have been studies on appropriate designing for the administration of antimicrobials through the measurement of their blood concentrations (i.e., TDM) [118, 122, 169]. Because inappropriate antimicrobial blood concentrations cause treatment failure or organ dysfunction, the clinical question of whether TDM-based treatment strategies improve sepsis outcomes is an important issue. [170–172]

We conducted a meta-analysis of five RCTs that evaluated TDM-based antimicrobial administration, focusing on mortality (five RCTs, 1011 patients) [173–177] and clinical cure (three RCTs, 250 patients) [173, 174, 176, 178]. Considering the relative value of each outcome, the net desirable effect yielded an RD of 124 more per 1000 (95% CI 57 fewer to 304 more). In contrast, no harm was basically expected from performing TDM. Based on these, we concluded that the balance of effects was probably better for the intervention (Additional file 3).

To measure blood concentrations of drugs, new measurement systems need to be set up with high-performance liquid chromatography or liquid chromatograph mass spectrometer (liquid chromatography with tandem mass spectrometry), making it difficult to introduce TDM. Implementation of TDM is considered especially for patients in whom blood concentrations of antimicrobials are expected to fluctuate.

### CQ 2–9: Is de-escalation based on culture and susceptibility results performed in antimicrobial therapy for sepsis?

*Answer*: We suggest applying de-escalation based on culture and susceptibility results performed in antimicrobial therapy for sepsis (GRADE 2C).

#### Rationale

The use of broad-spectrum antimicrobials promotes drug resistance (antimicrobial resistance, AMR), which is a worldwide problem, contributing to rising healthcare costs. De-escalation can be implemented from the perspectives of measures for AMR, infection management, and medical economics if it can be performed safely.

We conducted meta-analyses of one RCT and 17 observational studies. In these analyses, a decrease in overall mortality was considered a desirable effect, although the occurrence of superinfection was considered an undesirable effect. The results from one RCT (116 patients) [179] showed that the mortality yielded an RD of 78 more per 1000 (95% CI 64 fewer to 335 more; the certainty of evidence: very low), but that with 17 observational studies (4374 patients) [180–196] showed that mortality yielded an RD of 92 fewer per 1000 (95% CI 121 fewer to 58 fewer; the certainty of evidence: low). The small sample size in the RCT may have led to inconsistency in the results compared with that in the observational studies. Based on these, the desirable effect was assessed to be small. The meta-analysis with one RCT [179] demonstrated that the occurrence of superinfection yielded an RD of 166 more per 1000 (95% CI 8 more to 539 more). However, we could not perform a meta-analysis with the observational studies [180–196], as none of the studies evaluated the outcome, based on which the undesirable effect was assessed as unknown. Therefore, we concluded that the balance of effects was probably better for the intervention (Additional file 3).

The only intervention is a change in antimicrobials, which can be easily implemented in many medical facilities. De-escalation may extend the total duration of antimicrobial therapy [179], and care should be taken to avoid unnecessary extension of the administration period. [197]

### CQ2-10: In patients with sepsis receiving empiric antifungal drugs, are antifungal drugs discontinued using β-D glucan as an indicator?

*Answer*: We suggest the use of β-D glucan as an indicator for the discontinuation of antifungal drugs in patients with sepsis who have been administered empiric antifungal drugs (GRADE 2C).

#### Rationale

Because fungal infections, especially candidemia, have a high mortality rate [198, 199], the administration of empiric antifungal drugs is considered for patients with sepsis strongly suspected of having fungal infection. It takes time to make definitive diagnoses of fungal infections, and there are risks of drug-induced adverse events and selection of resistance strains. Therefore, whether antifungal drugs can be safely discontinued once the administration of empiric antifungal drugs has initiated is an important clinical issue.

We conducted a meta-analysis of two RCTs. As a result of β-D glucan-guided antifungal therapy, the duration of antifungal administration yielded a mean difference (MD) of 7.64 days shorter (95% CI 8.74 shorter to 6.54 shorter), [200, 201] and a 28–30-day mortality yielded an RD of 3 more per 1000 (95% CI 91 fewer to 146 more). The detection of antifungal-resistant candida yielded an RD of 20 more per 1000 (95% CI 47 fewer to 254 more). Considering the small effect size and wide 95% CI, we observed that there was a high degree of uncertainty and that the undesirable effect was small. Based on these, we concluded that the balance of effects was probably better for the intervention (Additional file 3).

This CQ examined the discontinuation of empiric antifungal drugs in patients with sepsis using β-D glucan. When a patient is definitively diagnosed with invasive candida infection, antifungal drugs should not be discontinued using only β-D glucan as an indicator. The effectiveness of starting empiric antifungal drugs in patients suspected of having infection with fungi other than Candida is unknown.

### CQ2-11: Is PCT used as an indicator for discontinuing antimicrobial therapy for sepsis?

*Answer*: We suggest the use of PCT as an indicator for discontinuing antimicrobial therapy for sepsis (GRADE 2A).

#### Rationale

A history of antimicrobial exposure is associated with the emergence of drug-resistant bacteria, and it may increase the risk for secondary sepsis [202, 203]. Currently, recommended durations of antimicrobials for each infection have become shorter, but whether they are applicable to sepsis is controversial. In patients with sepsis, decreases in PCT and CRP are associated with decreased mortality risk [204–206]. When making the decision to discontinue antibacterial drugs during sepsis treatment, whether the use of PCT or CRP can shorten the duration of antibacterial drugs without worsening outcomes is an important question.

In the present CQ, we conducted a network meta-analysis (NMA) in the following three groups in order to improve the accuracy of effect estimate; the PCT- and CRP-guided strategies and standard treatment.

The NMA was performed using 16 RCTs [207–222]. Regarding PCT-guided strategy, mortality yielded an RD of 32 fewer per 1000 (95% CI 53 fewer to 9 fewer), duration of antimicrobial therapy yielded a MD of 2.15 days shorter (95% CI 2.80 shorter to 1.50 shorter), and recurrence yielded an RD of 7 more per 1000 (95% CI 14 fewer to 32 more). We concluded that the balance of effects was probably better for the intervention (Additional file 3).

We did not create a recommendation for the CRP-guided strategy. Regarding the CRP-guided strategy, the duration of antimicrobial therapy yielded a MD of 2.69 days shorter (95% CI 4.70 shorter to 0.67 shorter). The CRP-guided strategy may slightly increase the mortality and recurrence, although it had a wide 95% CI.

### CQ2-12: Is short-term (≤ 7 days) antimicrobial therapy used for sepsis?

*Answer*: We suggest applying short-term (≤ 7 days) antimicrobial therapy for sepsis (GRADE 2C).

#### Rationale

The duration of antimicrobial therapy has been determined for each target organ and causative microorganism, but there is a lack of sufficient scientific basis. Regarding the duration of antimicrobial therapy for various infections, such as pneumonia, there are an increasing number of studies suggesting no difference in mortality rate or clinical cure rate between short- and long-term therapies [223, 224]. However, the duration of treatment for sepsis remains unclear. The risk of colonization and proliferation of antimicrobial-resistant bacteria, *Clostridioides difficile*, and fungi increases as the duration of antimicrobial administration becomes longer, which may yield a risk of superinfection. The clinical question of whether the duration of antimicrobial administration can be shortened without worsening patient outcomes is important.

We conducted a meta-analysis of six RCTs [225–230]. Short-term antimicrobial therapy was set as an intervention. A decrease in the detection of drug-resistant bacteria was set as a desirable effect, while a decrease in clinical cure, increase in mortality, and increase in new infection events were set as undesirable effects. The detection of drug-resistant bacteria yielded an RD of 132 fewer per 1000 (95% CI 166 fewer to 292 more), clinical cure yielded an RD of 24 fewer per 1000 (95% CI 96 fewer to 63 more), mortality yielded an RD of 5 more per 1000 (95% CI 23 fewer to 39 more), and new infection events yielded an RD of 26 more per 1000 (95% CI 20 fewer to 96 more). Considering the relative value of each outcome, we concluded that the balance of effects was probably better for the intervention (Additional file 3).

Few studies evaluated the short-term antimicrobial therapy for pneumonia, intra-abdominal infections, and bacteremia, and no studies evaluated that of urinary tract infections and cholangitis in critically ill patients. Therefore, this recommendation can serve as a reference for short-term antimicrobial treatment for sepsis caused by pneumonia, intra-abdominal infections, or bacteremia. When short-term therapy is applied, attention should be paid to the risks for recurrence and exacerbation.

## CQ3 Initial resuscitation

See Fig. 3.

**Fig. 3:**
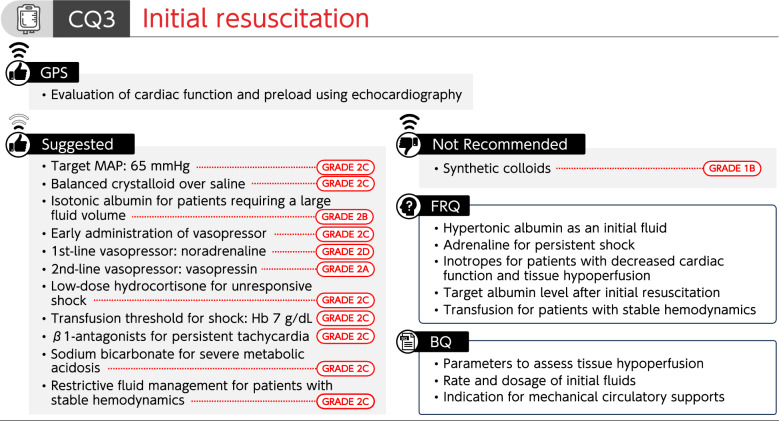
Summary of recommendations (CQ3 Initial resuscitation). *BQ* background question, *CQ* clinical question, *FRQ* future research question, *GPS* good practice statement, *Hb* haemoglobin, *MAP* mean arterial pressure

### CQ3-1: What parameters are used to assess tissue hypoperfusion in initial resuscitation for sepsis?

*Answer*: The measurement of blood lactate level is commonly performed, and the usefulness of CRT has also been reported to assess tissue hypoperfusion during initial resuscitation for sepsis (*Provision of information for background question*).

#### Rationale

Studies on parameters for timely evaluation of the effectiveness of initial resuscitation for sepsis exist. The J-SSCG 2020 and SSCG 2021 have proposed the lactate level and CRT as such parameters [8, 9, 72]. Lactate level is included in the criteria for diagnosing septic shock, and it is widely used as an indicator of tissue hypoperfusion. A multicenter RCT comparing the use of CRT with lactate level in initial resuscitation for sepsis reported a significant decrease in the SOFA score after 72 h and a trend toward reduced 28-day mortality in the CRT-guided group [231]. Early goal-directed therapy using central venous oxygen saturation (ScvO_2_) as a parameter did not improve the mortality rate or duration of wearing from mechanical ventilation compared to standard care [232]. Additionally, few studies investigated whether veno-arterial difference in partial pressure of carbon dioxide is useful for initial resuscitation in sepsis [233]. A meta-analysis of 17 RCTs (7729 patients) examining these parameters showed that both lactate-guided and CRT-guided therapy decreased the 90-day mortality compared to management without specific parameters [234]. In contrast, management using ScvO_2_ may increase the mortality rate compared to those using lactate level [234]. Thus, blood lactate levels and CRT are used as parameters of tissue hypoperfusion in initial resuscitation.

### CQ3-2: Are cardiac function and preload evaluated using echocardiography in initial resuscitation for sepsis?

*Answer*: Cardiac function and preload are evaluated using echocardiography while performing initial resuscitation for sepsis (*Good Practice Statement*).

#### Rationale

Patients with septic shock can present not only distributive shock associated with peripheral vasodilatation but also hypovolemic or cardiogenic shock. Evaluation using echocardiography, including cardiac function, preload, and fluid responsiveness, may optimize infusion fluid volume during initial resuscitation for sepsis, thereby improving prognosis. However, few RCTs have investigated the assessment using echocardiography in initial resuscitation for sepsis. Previous RCTs had a small sample size, and they did not show the efficacy of echocardiography for decreasing mortality [235, 236]. These results were possibly because the experience of ultrasound practitioners was varied, and the optimal thresholds and interventions have not been established. Despite these limitations, we consider that echocardiography is necessary in initial resuscitation for septic shock to examine differential causes of shock other than distributive shock.

### CQ3-3: What is the target mean arterial pressure (MAP) during initial resuscitation for sepsis?

*Answer*: We suggest 65 mmHg as the target MAP during initial resuscitation for sepsis (GRADE 2C).

#### Rationale

Vasopressors are commonly used in septic patients with hypotension. The risk of hypotension must be balanced against the potential adverse events caused by vasopressors. The SSCG 2021 recommended, with moderate certainty, a MAP of ≥ 65 mmHg as the initial target blood pressure in adults with septic shock who require vasopressors [72]. However, it is unclear whether maintaining a higher MAP improves outcomes, and we consider this as an important clinical issue.

We conducted a meta-analysis of three RCTs [237–239]. As the effect of setting a higher target MAP of 70–85 mmHg rather than 65 mmHg (60–70 mmHg), the short-term mortality yielded an RD of 12 fewer per 1000 (95% CI 43 fewer to 24 more), and RRT yielded an RD of 5 fewer per 1000 (95% CI 33 fewer to 27 more). Serious adverse events (arrhythmia, myocardial injury, extremity necrosis, and mesenteric ischemia) yielded an RD of 16 more per 1000 (95% CI 6 fewer to 44 more). The desirable effects were trivial, and the undesirable effects were small. Considering the relative value of each outcome and event rates, the benefit of targeting higher MAP was limited, and we concluded that the balance of effects was neither intervention nor comparator was superior (Additional file 3).

### CQ3-4: Which fluid is used for initial resuscitation of sepsis?

*Answer*: During initial resuscitation for sepsis, we suggest the administration of balanced crystalloid over normal saline (GRADE 2C).

We suggest the administration of isotonic albumin preparations (4–5%) when a patient with sepsis does not respond to standard treatment using crystalloids and requires a large volume of crystalloids (GRADE 2B).

During initial resuscitation for sepsis, we recommend against the administration of synthetic colloids (GRADE 1B).

#### Rationale

##### Balanced crystalloids

Large-volume administration of 0.9% sodium chloride (normal saline solution) may cause hyperchloremic metabolic acidosis and increase the risk of AKI [240]. With low-certainty evidence, the SSCG 2021 suggested the use of balanced crystalloids (crystalloids with chloride concentrations similar to that of plasma), rather than normal saline, for adult patients with sepsis or septic shock [72]. We included whether or not to use balanced crystalloids as an important clinical issue.

We analyzed eight publications (seven RCTs, including four cluster RCTs, and a secondary analysis study of one of the RCT) [241–248]. Since there was only one study focusing solely on sepsis, [248] the analysis included studies that partially targeted sepsis.

As the effect of using balanced crystalloids, short-term mortality yielded an RD of 8 fewer per 1000 (95% CI 18 fewer to 4 more), RRT yielded an RD of 4 fewer per 1000 (95% CI 12 fewer to 3 more), and hyperkalemia yielded an RD of 1 fewer per 1000 (95% CI 3 fewer to 4 more). However, the mechanical ventilator yielded an RD of 7 more per 1000 (95% CI 61 fewer to 88 more). The desirable effects were small, and the undesirable effects were trivial. Therefore, we concluded that the balance of effects was probably better for the intervention (Additional file 3).

##### Isotonic albumin solutions (4–5%)

We conducted a meta-analysis of four published RCTs [249–252]. As the effect of administering isotonic albumin preparations (4–5%), short-term mortality yielded an RD of 11 fewer per 1000 (95% CI 94 fewer to 97 more) (four RCTs) [249–252], and serious adverse events (pulmonary edema) yielded an RD of 583 fewer per 1000 (95% CI 723 fewer to 86 fewer) (one RCT). [249]

Therefore, we observed that the desirable effect was large. Since the studies did not examine any outcomes corresponding to undesirable effects, we found that the undesirable effect was unknown. We concluded that the balance of effects was better for the intervention (Additional file 3).

Among the RCTs in the meta-analysis, only the SAFE study described the administration of crystalloids prior to allocation [251], and the dosage of crystalloids prior to the start of albumin solutions remains unclear. However, the administration of crystalloids as an initial fluid is considered to be widely used in daily clinical practice, and we suggest it for patients who are unresponsive to standard treatment using crystalloids and require a large volume of crystalloids.

##### Synthetic colloids

Synthetic colloids (hydroxyethyl starches) are expected to increase intravascular volume by maintaining colloid osmotic pressure. A recommendation against the administration of synthetic colloids as an initial fluid was made by the J-SSCG 2020 [8, 9] and SSCG 2021 [72]. Whether or not the use of synthetic colloids as an initial fluid for sepsis remains an important issue.

We conducted a meta-analysis of four published RCTs [253–256]. Since there were no studies reporting outcomes that were expected to correspond to desirable effects of the use of synthetic colloids as an initial fluid, we found that the desirable effect was unknown. As for an undesirable effect, the short-term mortality yielded an RD of 9 more per 1000 (95% CI 25 fewer to 46 more), RRT yielded an RD of 55 more per 1000 (95% CI 5 more to 118 more), and serious bleeding events yielded an RD of 49 more per 1000 (95% CI 9 more to 104 more). Considering the relative value of each outcome, the net harm yielded an RD of 131 more per 1000 (95% CI 1 more to 261 more). We concluded that the balance of effects was probably better for the comparator (Additional file 3).

### CQ3-5: How is initial fluid therapy given for patients with sepsis?

*Answer*: Initial fluids for septic patients with reduced intravascular volume are aimed at optimizing circulating blood volume, and some patients require the administration of at least 30 mL/kg of crystalloid solutions within 3 h. However, there has been caution for harm caused by excessive fluid administration (*Provision of information for background question*).

#### Rationale

The J-SSCG 2020 described the necessity of administering at least 30 mL/kg of crystalloid solutions within 3 h during initial fluid therapy for patients with sepsis-induced tissue hypoperfusion and reduced intravascular volume, as well as the importance of avoiding excessive fluid administration with reference to various indicators [8, 9] As a strategy of initial fluid therapy, infusion fluids equivalent to 30 mL/kg are becoming widely used in daily clinical practice [257–259]. The harmful effects of excessive fluid administration have also been reported in fluid strategy after the completion of initial fluid therapy. [260]

Kuttab et al. reported that failure to reach an initial fluid resuscitation of 30 mL/kg within 3 h of sepsis onset was significantly associated with an increase in hospital mortality [261]. In large-scale RCTs, such as the ProCESS [262], ARISE [263], and ProMISe [264] trials, the volume of initial fluid prior to randomization was approximately 30 mL/kg, which is the volume widely used in daily clinical practice [257]. Subsequent large-scale RCTs on restricted fluid strategies, such as the CLASSIC [258] and CLOVERS [259] trials, also administered 30 mL/kg of resuscitation fluids prior to randomization. A meta-analysis of 15 studies on septic shock reported that excessive fluid balance increased mortality risk by 70%, but focusing on within 3 h of sepsis onset, high-dose administration of fluids led to a decrease hospital mortality [265]. The CLASSIC [258] and CLOVERS [259] trials conducted in recent years showed no difference in the 90-day mortality rate between restricted and unrestricted fluid administration groups.

Based on these, the current standard treatment is the administration of at least 30 mL/kg of crystalloids within 3 h, as an initial resuscitation fluid for septic shock patients with decreased intravascular volume. However, the volume of subsequent fluids continues to be debated.

### CQ3-6: Is early administration of vasopressor performed during initial resuscitation for sepsis?

*Answer*: During initial resuscitation for sepsis with hypotension, we suggest early administration of vasopressor combined with resuscitative fluid therapy (GRADE 2C).

#### Rationale

Early administration of vasopressors may avoid excessive fluid administration, thereby improving patient outcomes. In contrast, it may increase adverse events, including ischemic organ dysfunction. The J-SSCG 2020 suggested administering vasopressors simultaneously or in the early stages (within 3 h) of initial fluid resuscitation in patients with sepsis/septic shock who have difficulty in maintaining hemodynamics [8, 9]. Since then, several RCTs have been reported, and we considered this as an important clinical issue.

We conducted a meta-analysis of four RCTs [259, 266–268]. As the effect of early administration of vasopressor, mortality yielded an RD of 41 fewer per 1000 (95% CI 80 fewer to 17 more), pulmonary edema yielded an RD of 23 fewer per 1000 (95%CI 32 fewer to 10 fewer), and AKI yielded an RD of 10 fewer per 1000 (95% CI 28 fewer to 12 more). In contrast, ischemic organ dysfunction yielded an RD of 4 more per 1000 (95% CI 2 fewer to 21 more). Considering these results, we concluded that the balance of effects was probably better for early administration of vasopressors (Additional file 3).

In the CLOVERS study, approximately 30% of enrolled patients were administered vasopressors via peripheral venous lines, and 0.6% of the patients developed extravasation [259]. The occurrence of extravasation was reported in 3.4% (95% CI 2.5–4.7%) of patients administered vasopressor via peripheral venous lines, but tissue necrosis or limb ischemia were not reported [269]. The administration of vasopressor via peripheral venous lines may be acceptable to avoid delays, but the development of extravasation should be carefully monitored. The most common vasopressor used in those RCTs was noradrenaline [259, 266–268].

### CQ3-7: Which vasopressor is used as the first-line and second-line drugs in patients with septic shock?

*Answer*: We suggest using noradrenaline as the first-line vasopressor for septic shock (GRADE 2D), and vasopressin as the second-line vasopressor for septic shock (GRADE 2A).

#### Rationale

##### Noradrenaline

Patients with sepsis often develop hypotension due to venous vasodilation and decreased systemic vascular resistance. Thus, vasopressors are usually administered in initial resuscitation. The J-SSCG 2020 [8, 9] and the SSCG 2021 [72] recommended noradrenaline as the first-line vasopressor. Treatment for hypotension is an important clinical issue during initial resuscitation.

We evaluated four RCTs [270–273]. As the effect of noradrenaline administration, short-term mortality yielded an RD of 21 fewer per 1000 (95% CI 101 fewer to 69 more), arrhythmia yielded an RD of 124 fewer per 1000 (95% CI 176 fewer to 11 fewer), RRT yielded an RD of 1 more per 1000 (95% CI 21 fewer to 31 more); and organ ischemia (limb and intestinal tract) yielded an RD of 2 more per 1000 (95% CI 13 fewer to 17 more).

Considering the relative value of each outcome, the net benefit yielded an RD of 312 more per 1000 (95% CI 7 more to 617 more), and we concluded that the balance of effects was better for the intervention (Additional file 3).

##### Noradrenaline + vasopressin

A combination vasopressor therapy is considered in some patients, whose blood pressure cannot be maintained even with the use of noradrenaline. The present guidelines included this as an important clinical issue and evaluated the evidence for combination therapy with vasopressin, which is frequently used as the second-line vasopressor.

We conducted a meta-analyses of five RCTs [274–278]. As the effect of using vasopressin in addition to the noradrenaline, short-term mortality yielded an RD of 21 fewer per 1000 (95% CI 65 fewer to 31 more), mesenteric ischemia yielded an RD of 7 fewer per 1000 (95% CI 19 fewer to 16 more), and RRT yielded an RD of 115 fewer per 1000 (95% CI 191 fewer to 0). Meanwhile, acute coronary syndrome yielded an RD of 8 more per 1000 (95% CI 101 fewer to 69 more). Considering the relative value of each outcome, the net benefit yielded an RD of 178 more per 1000 (95% CI 3 more to 353 more). Therefore, we concluded that the balance of effects was probably better for the intervention (Additional file 3).

No analyses were conducted in a subgroup that was more likely to obtain the beneficial effects of vasopressin, as well as a subgroup that was more likely to obtain its harmful effects. The effectiveness of using vasopressin for septic shock with reduced cardiac function has not been investigated.

### CQ3-8: Are steroids administered for septic shock?

*Answer*: We suggest administering low-dose hydrocortisone (200–300 mg/day) to patients with septic shock unresponsive to initial fluid resuscitation and vasopressors for the purpose of recovering from shock (GRADE 2C).

#### Rationale

In patients with septic shock unresponsive to initial fluid resuscitation and vasopressors, relative adrenal insufficiency should be considered as the cause of persistent shock. Steroids are expected to lead to recovery from shock because they restore relative adrenal function, suppress inflammatory responses, exert vasoconstrictive effects, and improve responsiveness to vasopressors. In contrast, steroids may suppress immune function and increase the risk of infections, gastrointestinal hemorrhage, and hyperglycemia.

We conducted a meta-analysis of 11 RCTs [275, 279–288]. The steroid used in all of the RCTs was low-dose hydrocortisone (200–300 mg/day). As a result of the administration of low-dose hydrocortisone, short-term mortality yielded an RD of 12 fewer per 1000 (95% CI 40 fewer to 18 more), recovery from shock yielded an RD of 60 more per 1000 (95% CI 30 fewer to 164 more), and the duration of recovery from shock yielded an MD of 1.6 days shorter (95% CI 2.8 days shorter to 0.4 days shorter). In contrast, serious adverse events yielded an RD of 9 more per 1000 (95% CI 26 fewer to 54 more), secondary infections yielded an RD of 10 more per 1000 (95% CI 10 fewer to 31 more), and gastrointestinal hemorrhage yielded an RD of 12 more per 1000 (95% CI 16 fewer to 55 more). Considering these results, we concluded that the balance of effects was probably better for the administration of low-dose hydrocortisone (Additional file 3).

Among the included 11 RCTs [275, 279–288], hydrocortisone was administered intermittently in eight RCTs and continuously in three RCTs. Regarding blood glucose management, continuous administration reduced the workload needed to maintain tight blood glucose control [289], although it was reported to prolong the duration of hyperglycemia [290]. Regarding the method of hydrocortisone dose reduction, some RCTs gradually decreased its dose, while others interrupted the dose. The duration of hydrocortisone administration was 5–12 days.

### CQ3-9: What is the threshold of hemoglobin level for transfusion in initial resuscitation for septic shock?

*Answer*: We suggest a hemoglobin level of 7 g/dL as a threshold for transfusion in initial resuscitation for septic shock (GRADE 2C).

#### Rationale

The J-SSCG 2020 and SSCG 2021 suggested starting blood transfusion at a hemoglobin level of < 7 g/dL during initial resuscitation for patients with septic shock [8, 9, 72]. However, maintaining relatively high hemoglobin levels for shock may improve tissue hypoxia and reduce ischemic organ dysfunction. We compared management using higher and lower hemoglobin levels as a threshold of blood transfusion for septic shock.

We performed a meta-analysis of three RCTs [291–293]. All of the RCTs adopted a hemoglobin of 9 and 7 g/dL as higher and lower thresholds for transfusion, respectively. As a result of using a higher threshold, the mortality yielded an RD of 20 fewer per 1000 (95% CI 99 fewer to 69 more). In contrast, serious adverse events yielded an RD of 3 more per 1000 (95% CI 1 fewer to 113 more), and ischemic organ dysfunction yielded an RD of 1 more per 1000 (95% CI 23 fewer to 38 more) (Additional file 3).

The balance of effects was probably better for a higher threshold of hemoglobin level for blood transfusion. However, a meta-analysis similar to this CQ (although there was no improvement in patient-centered outcomes) highlighted that opportunities for blood transfusion increased by 32.8% and that the use of blood transfusion increased by 2.45 units in a liberal transfusion threshold (9 g/dL) group [294]. Considering these results, the threshold of hemoglobin for transfusion for septic shock can be set at 7 g/dL. However, higher hemoglobin levels may be preferred in patients with a history of hyperhemoglobinemia due to chronic hypoxemia, concomitant hemorrhagic shock, concomitant organ ischemia (such as myocardial infarction), and expected hemorrhage due to surgical procedures.

### CQ3-10: Are β1-adrenoceptor antagonists used for septic patients with persistent tachycardia after initial resuscitation?

*Answer*: We suggest administering β1-adrenoceptor antagonists for patients with sepsis to manage persistent tachycardia after initial resuscitation (GRADE 2C).

#### Rationale

Tachycardia and catecholamine administration are factors associated with poor prognosis in sepsis [295, 296]. In patients with septic shock, the use of β1-adrenoceptor antagonists is considered to manage tachycardia. However, β1-adrenoceptor antagonists may worsen hemodynamics, and their effectiveness has not been established.

We performed a meta-analysis of four RCTs [297–300]. As a result of administering β1-adrenoceptor antagonists, the short-term mortality yielded an RD of 206 fewer per 1000 (95% CI 271 fewer to 130 fewer), and arrhythmia yielded an RD of 160 fewer per 1000 (95% CI 213 fewer to 46 fewer). Meanwhile, serious adverse events yielded an RD of 3 more per 1000 (95% CI 62 fewer to 184 more). Considering these results, we concluded that the balance of effects was probably better for administering β1-adrenoceptor antagonists.

Recently, an RCT was published and demonstrated that the administration of landiolol might increase the 28-day mortality rate (37.1% vs. 25.4%, *p* = 0.16), resulting in early termination of the trial [301]. In patients with septic shock who were administered landiolol, noradrenaline was administered at a higher dose of approximately 0.1 μg/kg/min and with a longer duration of approximately 1 day compared to those without landiolol. When we performed a meta-analysis of five RCTs, including this new RCT [297–301], the balance of effects was still probably better for administering β1-adrenoceptor antagonists (Additional file 3). However, β1-adrenoceptor antagonists should be administered under careful monitoring of hemodynamics, as they may decrease cardiac output, lower blood pressure, and worsen tissue hypoperfusion.

### CQ3-11: Is sodium bicarbonate intravenously administered for septic patients with severe metabolic acidosis (pH ≤ 7.2)?

*Answer*: We suggest the intravenous administration of sodium bicarbonate for septic patients with severe metabolic acidosis (pH ≤ 7.2) (GRADE2C).

#### Rationale

Patients with sepsis often develop acute metabolic acidosis, and sodium bicarbonate is used for its correction. However, whether the administration of sodium bicarbonate for severe metabolic acidosis leads to improved outcomes is unclear and controversial. With low-certainty evidence, the SSCG 2021 stated that “For adults with septic shock and hypoperfusion-induced lactic acidemia, we suggest against using sodium bicarbonate therapy to improve hemodynamics or to reduce vasopressor requirements” [72].

Our analysis included four published studies [302–305] (three RCTs [302, 304, 305]), as well as a secondary analysis study [303] of one of the RCTs. Outcomes were extracted from only one study [302]. Short-term mortality yielded an RD of 91 fewer per 1000 (95% CI 172 fewer to 11 more), new-onset organ failure yielded an RD of 69 fewer per 1000 (95% CI 152 fewer to 28 more), and RRT yielded an RD of 165 fewer per 1000 (95% CI 242 fewer to 72 fewer). In contrast, severe metabolic adverse events requiring treatment intervention yielded an RD of 15 more per 1000 (95% CI 57 fewer to 118 more). Considering the relative value of each outcome, we concluded that the balance of effects was probably better for the intervention (Additional file 3).

### CQ3-12: What is the indication for mechanical circulatory support for septic shock?

*Answer*: There has been insufficient evidence for the effects of mechanical circulatory supports, such as V-A ECMO, intra-aortic balloon pumping, and intracardiac pump catheter (Impella^®^, Abiomed) for cardiac dysfunction in septic shock, and their indications have not been established (*Provision of information for background question*).

#### Rationale

Patients with septic shock may present not only with distributive shock but also with cardiogenic shock due to sepsis-induced myocardial dysfunction (SIMD) [306, 307]. In those patients, the incidences of left ventricular systolic, left ventricular diastolic, and right ventricular dysfunctions were reported to be 23–63% [308–311], 37–68% [309, 310], and 35–48% [310, 312], respectively, all of which may be associated with mortality. [309–312]

Few clinical trials have been reported on the effects of mechanical circulatory support in septic shock patients with SIMD. Twenty-eight-day survival rate in septic shock patients receiving intra-aortic balloon pumping was reported as approximately 30% [313]. In some case series and observational studies using V-A ECMO, survival rates varied widely among studies between 15 and 90% [314–319]. A meta-analysis of the prognosis of patients with septic shock who received V-A ECMO reported an hospital survival rate of 36% [320]. The effect of Impella^®^ in patients with SIMD has also been evaluated insufficiently, with only a few case reports reported [321, 322]. Therefore, there has been insufficient evidence on the effectiveness of mechanical circulatory supports in septic shock patients, and their indications have not been established.

Sepsis-induced myocardial dysfunction is a reversible clinical condition, and mechanical circulatory support may be used in septic patients with poor cardiac dysfunction if their hemodynamics cannot be maintained with inotropes. An appropriate device can be selected based on the assessment of the severity of shock-induced organ dysfunction, degree of cardiac dysfunction, and risk of complications. It is desirable that mechanical circulatory supports are provided at an experienced facility.

### CQ3-13: Is restrictive fluid management provided in septic patients with stable hemodynamics?

*Answer*: We suggest providing restrictive fluid management in septic patients with stable hemodynamics with monitoring for ischemic organ dysfunction due to hypoperfusion (GRADE 2C).

*Remarks*: Hypoperfusion can be comprehensively evaluated using skin findings (such as mottling and peripheral cyanosis), vital signs, capillary refill time, lactate levels, or urinary output.

#### Rationale

Both fluid overload and underload are associated with increased mortality in patients with sepsis [323]. Restrictive fluid management may improve prognosis by reducing organ congestion, but increase adverse events, including ischemic organ dysfunction.

We performed a meta-analysis of eight RCTs [258, 259, 267, 324–327]. As a result of restrictive fluid management, 90-day mortality yielded an RD of 6 fewer per 1000 (95% CI 34 fewer to 23 more), AKI or use of RRT yielded an RD of 19 fewer per 1000 (95% CI 37 fewer to 5 more), and serious adverse events yielded an RD of 8 fewer per 1000 (95% CI 28 fewer to 16 more). Based on these findings, we concluded that the balance of effects was probably better for restrictive fluid management (Additional file 3). Additionally, another systematic review and meta-analysis similar to our analysis showed no significant difference in any outcomes between higher and lower fluid volume managements [327]. Although sensitivity analyses were performed regarding the risk of bias, severity of illness, protocol, timing of intervention, and definition of sepsis, no significant difference was observed in any subgroups.

Restrictive fluid management is expected to reduce organ congestion associated with excessive fluids. However, in most of the RCTs included in our analysis, fluids of at least 20–30 mL/kg had already been administered before the study enrollment [258, 324, 326–329]. That means fluid volume administered during initial resuscitation was not restricted. If there is a concern about ischemic organ dysfunction due to hypoperfusion, resuscitation fluid should not be hesitated. Evaluation of fluid responsiveness is required to avoid excessive fluids.

### FRQ3-1: Is hypertonic albumin solutions (20–25%) used as an initial fluid for septic shock?

#### Rationale

The optimal albumin concentrations, isotonic (4–5%) or hypertonic (20–25%), for initial resuscitation in septic shock remain controversial. The clinical benefit of using hypertonic albumin as an initial resuscitation fluid in septic shock is uncertain.

There are two types of albumin solutions: isotonic (with a concentration close to that in human plasma: 4–5%) and hypertonic (with a high concentration: 20–25%). Experimental data and observational studies have suggested that hypertonic albumin solutions may be more effective than isotonic solutions in increasing intravascular volume and may enable resuscitation with smaller fluid volumes. In contrast, there is a possibility that they may not achieve the theoretical effect of increasing intravascular volume in patients with significant capillary leakage, such as septic shock. Additionally, rapid administration of hypertonic albumin solutions may induce a hyperosmolar state, leading to a decreased glomerular filtration rate.

The RCTs investigating the use of hypertonic albumin solutions for initial fluids for septic shock include the ERASS and ALPS trials. The ERASS trial (*n* = 792) showed no significant difference in the 28-day mortality between 20% albumin and 0.9% saline (24.1% vs. 26.3%), with comparable incidence rates of kidney failure [330]. In the ALPS trial (*n* = 100), there was no significant difference in the 28-day mortality between 20% albumin and crystalloids (58% vs. 56%) [331].

Further RCTs are needed to investigate whether the use of hypertonic albumin solutions reduces the volume of initial fluids for septic shock or whether it improves septic shock outcomes.

### FRQ3-2: Is adrenaline added when patients with septic shock have difficulty in maintaining hemodynamics with concomitant use of noradrenaline and vasopressin?

#### Rationale

The SSCG 2021 recommended the addition of adrenaline to achieve the target mean blood pressure during the initial resuscitation for sepsis when concomitant use of noradrenaline and vasopressin does not achieve a sufficient pressor effect after sufficient infusion fluid therapy [72], although this has not been investigated in any RCT. Under the use of high-dose noradrenaline, α1 receptors may already be saturated and downregulated. The administration of adrenaline is expected to exert effects as an inotrope in patients with decreased cardiac function, rather than the effects on α1 receptor. However, it may increase adverse events, such as organ ischemia associated with α stimulation effect and arrhythmia associated with β stimulation effect [332].

Future RCTs will investigate the usefulness of adrenaline, including the timing and dosage.

### FRQ3-3: Are inotropes used for septic shock patients with decreased cardiac function and tissue hypoperfusion?

#### Rationale

Approximately 40% of patients with septic shock are complicated by a cardiac dysfunction called SIMD, and its association with aggravation of the disease has been suggested [333, 334]. In order to maintain tissue perfusion in patients with SIMD-complicated septic shock, inotropes, dobutamine, and adrenaline, in addition to vasopressors, have been used. The SSCG 2021 recommended the concomitant use of noradrenaline and dobutamine, or the administration of adrenaline alone for septic shock patients with decreased cardiac function, who exhibit persistent tissue hypoperfusion despite maintaining appropriate fluid resuscitation and maintained arterial pressure, although it is not based on sufficient evidence [72].

Inotropes include dobutamine, adrenaline, and calcium (Ca) sensitizers. The SSCG 2021 suggested against the use of Ca sensitizers and did not mention PDE III inhibitors [72]. At the time of publication of the SSCG 2021, there were no RCTs comparing dobutamine, adrenaline, and PDE III inhibitors. Regarding the use of Ca sensitizers, three RCTs have been conducted, and no association with mortality was found in a group using Ca sensitizers compared to a placebo group. However, a multicenter RCT study suggested that the use of Ca sensitizers hindered successful weaning from invasive mechanical ventilation and increased supraventricular arrhythmia [335].

Since the publication of the SSCG 2021, there have been no new RCTs on the use of inotropes in patients with septic shock complicated by decreased cardiac function.

### FRQ3-4: Is the serum albumin level maintained at 3.0 g/dL using hypertonic albumin solutions (20–25%) after initial resuscitation for septic shock?

#### Rationale

Albumin has various properties, including increasing intravascular volume, regulating colloid osmotic pressure, binding and transporting different molecules, exerting anti-inflammatory and antioxidant effects, and regulating nitric oxide metabolism. Hypoalbuminemia is associated with poor prognosis in critically ill patients, and the aforementioned effects of albumin may be lost in patients with sepsis.

Hypertonic albumin solutions may correct hypoalbuminemia, maintain colloid osmotic pressure, reduce edema, and improve outcomes in patients with septic shock. However, the clinical benefit of maintaining serum albumin levels in patients with septic shock using hypertonic albumin solutions remains uncertain.

In the ALBIOS trial, patients administered 20% albumin solutions and crystalloids to maintain serum albumin levels ≥ 3.0 g/dL were compared with those administered crystalloids alone. The study showed no significant difference in the 28- and 90-day mortality [336]. However, a subgroup analysis suggested that the maintenance of serum albumin levels may reduce the 90-day mortality in patients with septic shock (*n* = 1121) (risk ratio: 0.87 [95% CI 0.77–0.99]). Two ongoing RCTs, the ARISS [337] and ALBIOSS-BAL [338] trials, tested this hypothesis after initial resuscitation for septic shock.

### FRQ3-5: What is the threshold of hemoglobin levels for transfusion in patients with sepsis who have stable hemodynamics?

#### Rationale

Tissue hypoxia accompanying anemia is a clinically important issue. Blood transfusion is performed to treat and prevent tissue hypoxia, but increasing blood transfusion is associated with the risk of allergies, infection, blood transfusion, transfusion-associated circulatory overload, and transfusion-related acute lung injury. The threshold of hemoglobin level for transfusion in patients with sepsis who have stable hemodynamics has not been established.

In a RCT conducted by Hebert et al. [339], 838 critically ill patients with a hemoglobin of < 9.0 g/dL were randomly allocated into the following two groups; (1) those that maintained hemoglobin of 7.0–9.0 g/dL (restricted transfusion group; 418 patients); and (2) those that maintained hemoglobin of 10.0–12.0 g/dL with a blood transfusion threshold of hemoglobin 10.0 g/dL (unrestricted transfusion group; 420 patients). No significant difference was observed in the 30-day mortality, which was the primary endpoint (restricted transfusion group, 18.7% vs. unrestricted transfusion group, 23.3%; *p* = 0.11), but hospital mortality was significantly lower in the restricted transfusion group (22.3% vs. 28.1%, *p* = 0.05). A subgroup analysis in patients with septic shock showed no significant difference in the 30-day mortality (restricted transfusion group 22.8% vs. unrestricted transfusion group 29.7%, *p* = 0.36).

No clinical trials have been conducted to investigate the threshold of hemoglobin levels for the initiation of blood transfusion in patients with sepsis who have no signs of shock or have recovered from shock. Clinical trials investigating this question are warranted.

## CQ4 Blood purification

See Fig. 4.

**Fig. 4:**
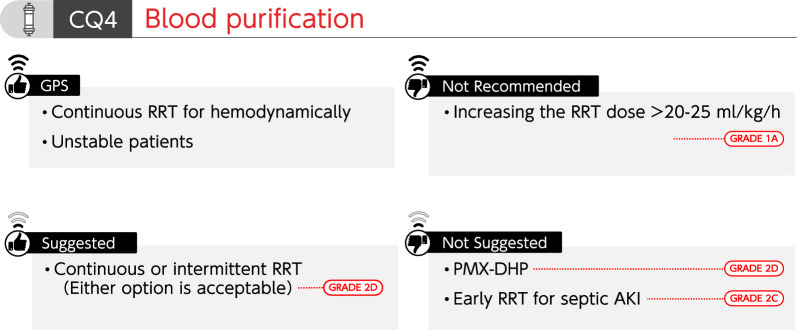
Summary of recommendations (CQ4 Blood purification). *AKI* acute kidney injury, *CQ* clinical question, *GPS* good practice statement, *PMX-DHP* polymyxin B-immobilized fiber column, *RRT* renal replacement therapy

### CQ4-1: Is PMX-DHP used for patients with septic shock?

*Answer*: We suggest against using PMX-DHP for patients with septic shock (GRADE 2D).

#### Rationale

Direct hemoperfusion with PMX-DHP is used for endotoxin adsorption. The J-SSCG 2020 suggested against PMX-DHP for septic shock [8, 9], but the effectiveness of PMX-DHP remains controversial.

We conducted a meta-analysis of four RCTs [340–343]. With regard to the desirable effects of PMX-DHP, mortality yielded a RD of 37 fewer per 1000 (95% CI 134 fewer to 110 more), and organ dysfunction score yielded a standardized mean difference (SMD) of 0.49 lower (95% CI 1.2 lower to 0.21 higher). With regard to the undesirable effects of PMX-DHP, complications, such as hemorrhage and in-circuit coagulation, yielded an RD of 216 more per 1000 (95% CI 91 fewer to 1000 more), vasopressor-free days yielded a MD of 1.8 days shorter (95% CI 4.1 days shorter to 0.5 days longer). The desirable effects were trivial, and the undesirable effects were large. Thus, we concluded that the balance of effects was probably better for the comparator (Additional file 3).

Further evidence will be accumulated in future studies, including the currently ongoing RCT (the TIGRIS trial) [344].

### CQ4-2: Is early RRT performed for septic AKI?

*Answer*: We suggest against performing early RRT for patients with septic AKI (GRADE 2C).

#### Rationale

Patients with septic AKI have higher severity of AKI, requirement of RRT, and mortality rates than those with non-septic AKI [345]. However, there are no clear standards on the timing of starting RRT in septic patients with AKI. In 2020, a large-scale RCT (the STARRT-AKI trial) was reported [346]. In the present guidelines, “early” was defined as AKI stage 2/3 or within 12 h fulfilling inclusion criteria.

We conducted a meta-analysis of four RCTs [346–349]. With regard to the desirable effects of early RRT, dialysis dependence yielded an RD of 12 fewer per 1000 (95% CI 40 fewer to 70 more), and hemorrhagic complications yielded an RD of 5 fewer per 1000 (95% CI 12 fewer to 8 more). With regard to the undesirable effects, mortality yielded an RD of 8 more per 1000 (95% CI 23 fewer to 38 more). Thus, we concluded that the balance of effects was unknown (Additional file 3).

The actual timing of starting RRT may vary, depending not only on medical aspects but also on the availability of RRT equipment and human resources of each facility. When performing RRT, clinical situation, such as medical resources and the will of patients are considered.

### CQ4-3: Is continuous RRT provided for septic AKI?

*Answer*: Either continuous or intermittent RRT can be selected as an RRT modality for septic AKI (GRADE 2D).

However, continuous RRT is used for hemodynamically unstable patients (*Good Practice Statement*).

#### Rationale

Renal replacement therapy is an essential life support for patients with advanced septic AKI. RRT is classified into continuous RRT (CRRT) and intermittent RRT (IRRT). The J-SSCG 2020 [8, 9] stated that CRRT should be selected for hemodynamically unstable patients and that either CRRT or IRRT can be selected for patients with stable hemodynamics. Whether to use CRRT or IRRT depends not only on the clinical condition but also on the experience and healthcare-providing system of each facility.

We conducted a meta-analysis of five published RCTs [350–354] With regard to the desirable effect of CRRT, hemorrhagic complications yielded an RD of 3 fewer per 1000 (95% CI 29 fewer to 46 more). With regard to the undesirable effects, mortality yielded an RD of 38 more per 1000 (95% CI 49 fewer to 136 more), and dialysis dependence yielded an RD of 4 more per 1000 (95% CI 38 fewer to 106 more). We concluded that the balance of effects was better for IRRT (Additional file 3). However, we suggest that either continuous or intermittent RRT can be selected, considering both are widely used in clinical settings. An observational study stated that CRRT is generally selected for patients with unstable hemodinamics [355].

### CQ4-4: Is treatment dose increased in RRT for septic AKI?

*Answer*: We recommend against increasing the RRT dose beyond the international standard dose (20–25 mL/kg/h) for patients with septic AKI (GRADE 1A).

#### Rationale

In the provision of RRT for patients with septic AKI, increasing the dialysis and filtration dose has been expected to improve prognosis. Approximately 25 mL/kg/h is considered the standard prescribed dose internationally. Setting the prescribed dose with the highest treatment effect for septic AKI is an important issue for improving prognosis.

We performed a meta-analysis of three published RCTs [356–358]. Since the desirable effect of increasing the treatment dose (35–40 mL/kg/h) was not found in the meta-analysis, we assessed that the desirable effect was unknown. With regard to the undesirable effects, mortality yielded an RD of 26 more per 1000 (95% CI 9 fewer to 64 more), dialysis dependence yielded an RD of 68 more per 1000 (95% CI 51 fewer to 226 more), and complications (hypophosphatemia) yielded an RD of 124 more per 1000 (95% CI 4 more to 286 more). Thus, we concluded that the balance of effects was better for the comparator (Additional file 3).

The RRT dose used in the RCTs of this meta-analysis was 20–25 mL/kg/h [356–358]. The dose approved by Japanese health insurance is 15 mL/kg/h. The efficacy of RRT with the lower dose is unclear.

## CQ5 Disseminated intravascular coagulation

See Fig. 5.

**Fig. 5:**
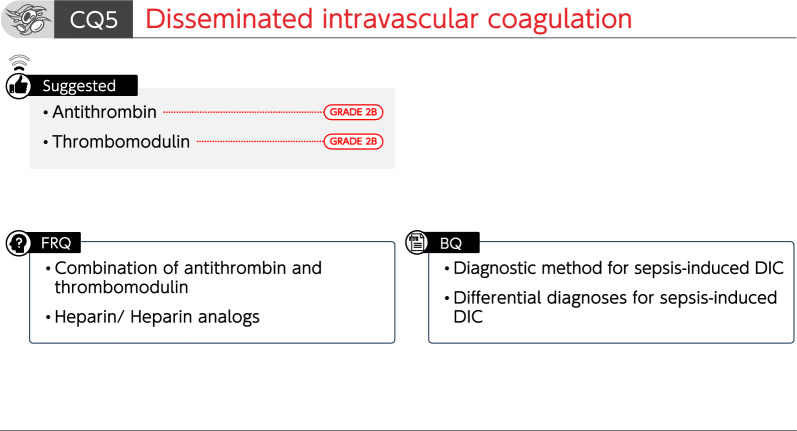
Summary of recommendations (CQ5 Disseminated intravascular coagulation). *BQ* background question, *CQ* clinical question, *DIC* disseminated intravascular coagulation, *FRQ* future research question

### CQ5-1: What is the diagnostic method for sepsis-induced DIC?

*Answer*: Several diagnostic criteria for DIC in patients with sepsis have been proposed. The JAAM-DIC and the SIC diagnostic criteria are used to diagnose early DIC and to determine treatment initiation. The ISTH overt DIC diagnostic criteria are used to diagnose progressed DIC and predict mortality (*Provision of information for background question*).

#### Rationale

The first diagnostic criteria for DIC, the Japanese Ministry of Health and Welfare DIC diagnostic criteria, were established in 1979, followed by the ISTH overt DIC, JAAM DIC, and SIC. The JAAM DIC diagnostic criteria, which are widely used in clinical practice in Japan, included the SIRS score and considered the rate of decrease in platelet count over time in addition to the platelet count at a certain cut-off as diagnostic items, aiming to sensitively detect inflammation-associated coagulation abnormalities [359]. The SIC diagnostic criteria included the SOFA score as a diagnostic item, in addition to the routine test of prothrombin time (PT) and platelet count, which is consistent with the change in sepsis diagnostic criteria from the SIRS score to the SOFA score [360]. The overt DIC diagnostic criteria, which are commonly used internationally, are stricter than the JAAM-DIC or SIC diagnostic criteria, and they are used to avoid overdiagnosis and identify severe DIC cases [361].

These diagnostic criteria can be used according to the purpose, as there is no gold standard for DIC diagnosis, and it is difficult to assess which criteria are superior to others. The JAAM-DIC and SIC diagnostic criteria are used to diagnose early DIC and determine treatment initiation, whereas the ISTH overt DIC diagnostic criteria are used to diagnose progressed DIC and predict mortality.

### CQ5-2: What are the differential diagnoses for patients with suspected sepsis-induced DIC?

*Answer*: DIC-like clinical conditions include TMA and HIT, which require differential diagnosis (*Provision of information for background question*).

#### Rationale

In ICUs, 9–19% of thrombocytopenia cases are caused by DIC [362], and sepsis-induced DIC accounts for the majority of these cases. However, some clinical conditions that require different treatment strategy also cause blood test abnormalities similar to those of DIC. Although anticoagulat therapy is considered effective for DIC, it is not only ineffective for such clinical conditions, but may also be harmful. Therefore, it is important to differentiate between sepsis-induced DIC and its similar clinical conditions. In patients with TMA prompt specific treatments can improve mortality or prevent serious sequelae. Thrombotic microangiopathy has three main symptoms as follows; microangiopathic hemolytic anemia, consumptive thrombocytopenia, and organ damage due to microvascular platelet thrombosis. Prolonged PT and elevated fibrin degradation product (FDP) observed in DIC are usually absent or mild [363].

Sepsis-induced DIC should be diagnosed promptly. However, even if a patient is diagnosed with DIC, the actual diagnosis may be TMA instead of DIC or TMA concurrently with DIC. If the patient exhibits poor response to DIC treatment or atypical clinical signs, clinicians should consider the possibility of TMA, and prompt diagnosis and conversion to specific treatment (such as plasma exchange and molecular target drug) are required.

Other diseases that need to be differentiated from sepsis-induced DIC except TMA include HIT, hemolysis, elevated liver enzymes, and low platelets (HELLP) syndrome, and severe hepatic dysfunction [364–366]. For any of these diseases, thrombocytopenia can be the trigger for diagnosis.

### CQ5-3: Is antithrombin administered for sepsis-induced DIC?

*Answer*: We suggest the administration of antithrombin for sepsis-induced DIC (GRADE 2B).

#### Rationale

Antithrombin has anticoagulant effects, mainly by inhibiting thrombin and activated factor X. Additionally, it has an anti-inflammatory effect that may help control sepsis-induced DIC [367]. Previous meta-analyses have conflicting results regarding its contribution to improving the prognosis of sepsis-induced DIC, and no clear evidence has been established [368, 369]. The J-SSCG2020 suggested the administration of antithrombin (GRADE 2C) [8, 9].

We conducted a meta-analysis of five RCTs using a decrease in mortality and recovery from DIC as desirable effects [370–374]. Concerning the desirable effects of antithrombin, the analysis of the five RCTs [370–374] showed that mortality yielded a RD of 147 fewer per 1000 (95% CI 214 fewer to 67 fewer), and analysis of three RCTs [370, 371, 374] showed that recovery from DIC yielded an RD of 448 more per 1000 (95% CI 161 more to 999 more). Concerning the undesirable effect of antithrombin, analysis of the three RCTs [370, 373, 374] showed that bleeding complications yielded an RD of 8 more per 1000 (95% CI 24 fewer to 89 more). Considering the relative value of each outcome, the desirable effects were large, and the undesirable effects were trivial. Thus, we concluded that the balance of effects was better for the intervention (Additional file 3).

Future studies would clarify issues, such as the optimal dosage, target activity levels, and criteria for starting and discontinuing administration. In clinical practice, individual decision must be made, depending on the general conditions of patients.

### CQ5-4: Is recombinant thrombomodulin administered for sepsis-induced DIC?

*Answer*: We suggest the administration of recombinant thrombomodulin for sepsis-induced DIC (GRADE 2B).

#### Rationale

Recombinant thrombomodulin has an anticoagulant effect by binding to thrombin and activating protein C. In addition, it exerts an anti-inflammatory effect through its lectin-like domain [375]. However, there has been no sufficient evidence for recombinant thrombomodulin in patients with sepsis, and no definitive conclusion has been reached on its efficacy [376–378]. Therefore, we evaluated recombinant thrombomodulin for sepsis-induced DIC.

We conducted a meta-analysis of four RCTs [379–382], using a decrease in mortality as a desirable effect. The results showed that mortality yielded an RD of 39 fewer per 1000 (95% CI 75 fewer to 3 more). Additionally, a meta-analysis of three RCTs [380–382] showed that recovery from DIC yielded an RD of 120 more per 1000 (95% CI 4 more to 274 more), also considered a desirable effect.

For adverse effects, another meta-analysis of four RCTs [379–382] showed that bleeding complications yielded an RD of 12 more per 1000 (95% CI 6 fewer to 41 more). Considering the relative value of each outcome, we found that the beneficial effects were substantial, while the adverse effects were minimal. Thus, we concluded that the balance of effects was probably better for the intervention (Additional file 3).

The frequency and risk of hemorrhagic complications in sepsis-induced DIC vary, depending on the pathophysiology and presence or absence of invasive treatment. Thus, clinicians should exercise caution regarding bleeding complications when administering recombinant thrombomodulin.

### FRQ5-1: Are antithrombin and thrombomodulin concomitantly administered for sepsis-induced DIC?

#### Rationale

The J-SSCG 2020 recommended the administration of antithrombin or recombinant thrombomodulin for sepsis-induced DIC [8, 9]. In Japan, some facilities use combination therapy with antithrombin and recombinant thrombomodulin, but there is currently no consistent opinion on its effectiveness. Thus, the present guidelines covered the concomitant administration of antithrombin and recombinant thrombomodulin for sepsis-induced DIC as an FRQ. We conducted a meta-analysis of seven observational studies [383–390] and examined the usefulness of the combination therapy for sepsis-induced DIC using a random-effects model. As a result of the combination therapy, there was a decreasing tendency in mortality rate (odds ratio: 0.89, 95% CI 0.74–1.07, heterogeneity: 72%), although there was no significant difference. Additionally, the incidence of hemorrhagic complications in the combination therapy was comparable to that in monotherapy. This meta-analysis has several limitations, including that all of the studies were conducted in Japan, that they were observational studies rather than RCTs, and that there was a statistically high heterogeneity. Therefore, we currently present the CQ as an FRQ without providing any recommendations.

### FRQ5-2: Is heparin or heparin analogs administered for sepsis-induced DIC?

#### Rationale

The J-SSCG2020 suggested against the administration of heparin or heparin analogs for sepsis-induced DIC [8, 9]. However, heparin administration in the pathophysiology of sepsis is attracting renewed attention based on the usefulness of heparin for coagulation abnormalities in patients with COVID-19, as well as several reports suggested the effect of heparin in improving the prognosis of sepsis and sepsis-induced DIC [391–393]. Therefore, in order to reconsider the possibility of heparin and heparin analogs, the present guidelines mentioned them as an FRQ.

A systematic review by Fu et al. has suggested that the administration of heparin for patients with sepsis may improve prognosis [394]. Additionally, a study using the US Medical Information Mart for Intensive Care-IV database for SIC reported that the early administration of heparin improved ICU mortality rate [395]. Heparin may be useful, depending on the timing of administration and selected target. A systematic review by Li et al. reported that the administration of low-molecular-weight heparin for patients with sepsis may improve prognosis and reduce hemorrhagic risk [396]. However, we set the CQ as an FRQ without providing any recommendations because we found that there was insufficient evidence to provide a recommendation from the perspective of the risk of bias. Large-scale RCTs and high-quality observational studies are needed to clarify the effectiveness of heparin and heparin analogs for sepsis.

## CQ6 Adjuvant therapy

See Fig. 6.

**Fig. 6:**
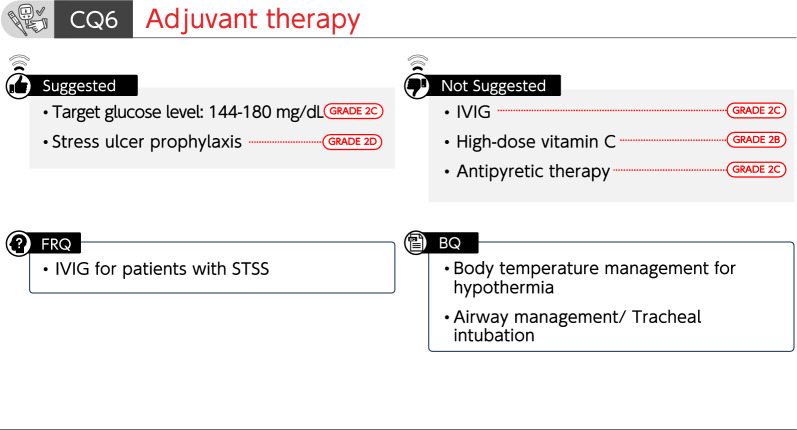
Summary of recommendations (CQ6 Adjuvant therapy). *BQ* background question, *CQ* clinical question, *FRQ* future research question, *IVIG* intravenous immunoglobulin, *STSS* streptococcal toxic shock syndrome

### CQ6-1: Is IVIG administered for sepsis?

*Answer*: We suggest against the administration of IVIG for sepsis (GRADE 2C).

#### Rationale

IVIG includes specific antibodies against various bacteria, toxins, and viruses. Immunoglobulin neutralizes pathogenic microorganisms and toxins, promote phagocytosis and bacteriolysis through complement activation, has opsonic, antibody-dependent cellular cytotoxic, non-specific anti-inflammatory effects, and suppresses inflammatory cytokine production. Patients with sepsis have decreased serum immunoglobulin G (IgG) levels from the early stage of onset due to decreased production, capillary leakage, and wasting consumption. The incidence of shock and mortality rate increase significantly if serum IgG levels are severely decreased [397, 398]. Based on the background of the aforementioned studies, the administration of IVIG along with appropriate systemic management and early administration of antimicrobials may improve prognosis.

We performed a meta-analysis of nine published RCT [399–407]. As a result of IVIG administration, the adverse events yielded an RD of 1 fewer per 1000 (95%CI 23 fewer to 46 more; two RCTs, 724 patients) [400, 401]. In contrast, the short-term mortality yielded an RD of 14 more per 1000 (95% CI 51 fewer to 88 more; three RCTs, 745 patients) [399–401]. Considering these results, we concluded that the balance of effects was probably better for the comparator (Additional file 3).

The use of IVIG as a standard treatment is undesirable. However, we do not exclude the indications in special pathophysiological conditions, such as STSS [408, 409]. This point has been summarized in FRQ6-1.

### CQ6-2: Is high-dose vitamin C therapy used for sepsis?

*Answer*: We suggest against the use of high-dose vitamin C therapy for sepsis (GRADE 2B).

#### Rationale

Vitamin C, a water-soluble vitamin, cannot be synthesized within the body. It has been reported in recent years that high-dose administration of vitamin C may improve survival rate in patients with sepsis [410]. Many RCTs have been conducted [411–428], and there is a concern that administration of high-dose vitamin C may cause kidney injury.

We conducted a meta-analysis of 18 published RCTs [411–428]. With regards to mortality, long-term mortality (≥ 60 days) was adopted as an outcome, as we decided to adopt the outcome with the highest certainty of evidence. As a result of high-dose vitamin C administration, the long-term mortality yielded an RD of 23 more per 1000 (95% CI 15 fewer to 69 more; six RCTs, 2148 patients) [411–416], and AKI yielded an RD of 26 more per 1000 (95% CI 34 fewer to 104 more; six RCTs, 1846 patients) [412, 413, 416–419]. The balance of effects was probably better for the comparator (Additional file 3).

This recommendation concerns the administration of high-dose vitamin C to patients with sepsis, and it does not discourage the administration of standard-dose vitamin C as a nutritional therapy.

### CQ6-3: What is the target blood glucose level for sepsis?

*Answer*: We suggest 144–180 mg/dL as a target blood glucose level for sepsis (GRADE 2C).

#### Rationale

It has been recommended that the glucose control in an ICU should avoid both low (< 110 mg/dL) [429] and high (≥ 180 mg/dL) ranges. However, there is a question as to whether there is a difference in the incidence of hypoglycemia between blood glucose levels near 110 mg/dL and 180 mg/dL. To clarify this question, we divided the range of blood glucose levels (110–180 mg/dL) into two based on the NICE-SUGAR study [430]: 110–144 and 144–180 mg/dL. We then conducted a network meta-analysis (NMA) using the four range of blood glucose levels as follows: < 110 mg/dL, 110–144, 144–180, and ≥ 180 mg/dL.

An NMA was performed using 36 RCTs [8, 9, 429–464]. As a result, the balance of effects among the groups was summarized as follows. A range of < 110 mg/dL was inferior to all the other ranges. Second, a range of 144–180 mg/dL was superior to 110–144, and ≥ 180 mg/dL was not superior to 110–144 mg/dL. Finally, values ≥ 180 mg/dL was not superior to 144–180 mg/dL. Therefore, we observed that 144–180 mg/dL was the most optimal target level (Additional file 3).

To prevent insulin-induced hypoglycemia, it is important to measure blood glucose at appropriate intervals during continuous administration of insulin. However, we did not examine appropriate intervals of blood glucose measurement. The European Society for Clinical Nutrition and Metabolism guideline recommended measuring blood glucose at least every 4 h for 48 h after ICU admission as good practice points, and stated that more frequent measurements may be needed, depending on patients’ conditions. [465]

Methods for measuring blood glucose levels in acute-phase conditions include measurements using blood biochemical testing in a laboratory, blood gas analyzer, or a simple blood glucose meter with arterial/venous and capillary blood. Measurement using glucometer with capillary blood can result in significant errors and have a risk of overlooking hypoglycemia [8, 9].

### CQ6-4: Is antipyretic therapy provided to febrile patients with sepsis?

*Answer*: We suggest against antipyretic therapy for febrile patients with sepsis (GRADE 2C).

#### Rationale

Patients with sepsis frequently develop fever, which causes patient discomfort, increased oxygen demand, and central nervous system disorders. On the other hand, fever serves as a defense reaction that activates the immune system and is associated with the promotion of the elimination of pathogenic microorganisms. Antipyretic therapy is frequently administered to reduce discomfort and oxygen demand and prevent central nervous system disorders. However, because it may also suppress defense reaction, the balance of its benefits and harms needs to be clarified.

We conducted a meta-analysis of seven published RCTs that examined antipyretic therapy comprising acetaminophen, extracorporeal cooling, or a combination of both, compared with a non-intervention group [466–472]. Six RCTs examined drug therapy [466, 467, 469–472] (one of the RCTs examined the concomitant use of antipyretics with body-surface cooling [467]), while one RCT examined intervention using body-surface cooling [468]. As a result of the antipyretic therapy, 28- or 30-day mortality yielded an RD of 43 more per 1000 (95% CI 48 fewer to 174 more; four RCTs, 1236 patients) [466–469]. Additionally, all serious adverse events yielded an RD of 1 more per 1000 (39 fewer to 74 more; four RCTs, 1312 patients) [466, 469–471], and infectious complications yielded an RD of 28 fewer per 1000 (70 fewer to 54 more; three RCTs, 510 patients) [466, 467, 472]. The effects of antipyretic therapy were limited. Thus, we concluded that the balance of effects was probably better for the comparator (Additional file 3). However, this suggestion may not be applied in remarkable hyperthermia or in cases where alleviating fever-associated symptoms is prioritized.

### CQ6-5: Is stress ulcer prophylaxis performed for patients with sepsis to prevent gastrointestinal hemorrhage?

*Answer*: We suggest performing stress ulcer prophylaxis for patients with sepsis to prevent gastrointestinal bleeding (GRADE 2D).

#### Rationale

Since stress ulcer may cause gastrointestinal bleeding in intensive care patients, pharmacological ulcer prophylaxis is indicated. However, there are concerns about the side effects of antacids, such as pneumonia and *Clostridioides difficile* infection. Therefore, it is necessary to clarify the balance of benefits and harms of the prophylactic use of antacids.

We conducted a meta-analysis of 32 published RCTs and one additional outcome report evaluating the effects of stress ulcer prophylaxis in non-specific intensive care patients [473–505]. The following five outcomes were assessed: gastrointestinal bleeding (30 RCTs, 6866 patients) [473–502], mortality (14 RCTs, 5065 patients) [473, 475, 477, 480, 482, 487, 488, 490, 491, 496, 498, 500, 503, 504], pneumonia (15 RCTs, 5146 patients) [473, 475, 477, 479, 485, 487, 488, 490–494, 500, 503, 505], serious adverse events (seven RCTs, 4143 patients) [477, 487, 488, 495, 497, 500, 503] and *Clostridioide*s infection (three RCTs, 3607 patients) [480, 500, 503]. Regarding desirable effects, gastrointestinal bleeding yielded an RD of 66 fewer per 1000 (95% CI 84 fewer to 43 fewer), and *Clostridioides* infection yielded an RD of 4 fewer per 1000 (9 fewer to 5 more). In contrast, mortality rate yielded an RD of 10 more per 1000 (13 fewer to 36 more), and pneumonia yielded an RD of 8 more per 1000 (12 fewer to 29 more). Serious adverse events also yielded an RD of 5 more per 1000 (6 fewer to 20 more). Considering the relative value of each outcome, we concluded that the balance of effects was probably better for the intervention (Additional file 3). It should be noted that this recommendation was drawn from data of patients receiving intensive care, but not specific for patients with sepsis.

### CQ6-6: How is the body temperature managed in septic patients with hypothermia?

*Answer*: Rewarming therapy might be rational when hypothermia-associated circulatory disorders or coagulation abnormalities are observed in septic patients with hypothermia (core body temperature of < 35 °C). However, caution should be taken as rewarming therapy may cause peripheral vasodilation, resulting in adverse events, such as hypotension (*Provision of information for background question*).

#### Rationale

Hypothermia is one of the body temperature abnormalities that occur in patients with sepsis. Septic patients with hypothermia have poor prognosis, and hypothermia affects the defense mechanism against microbial infection and causes complications, such as decreased cardiac function, arrhythmia, electrolyte abnormalities, and coagulopathy.

Hypothermia is independently associated with poor prognosis in patients with sepsis [506]. A multicenter observational study in Japan reported that 11.1% of patients with sepsis had hypothermia of < 36 °C at the time of ICU admission [507]. Compared to patients with body temperature > 38 °C at the time of ICU admission, unadjusted odds ratio of hospital mortality for patients with hypothermia (< 36 °C) was 1.76 (95% CI 1.13–2.73), indicating a poor prognosis for septic patients with hypothermia [507].

Hypothermia (core body temperature of < 35 °C) leads to decreased immune function due to dysregulation of inflammatory cytokines, such as interleukin 6 and tumor necrosis factor-α, and lymphopenia [508]. Hypothermia causes decreased cardiac function, arrhythmia, cold diuresis, electrolyte abnormalities, and coagulation abnormalities, and severe hypothermia develops unstable hemodynamics and hemorrhagic tendency [509–511]. Based on these serious complications, rewarming might be rational in septic patients with hypothermia [512]. A questionnaire survey described that 96% of respondents reported that there was no protocol for the management of hypothermic sepsis, although 62% of the respondents actively rewarmed patients with hypothermic sepsis [513]. When providing rewarming therapy, attention should be paid to the occurrence of adverse events, such as hypotension due to peripheral vasodilatation, altered the balance between oxygen demand and supply, and electrolyte abnormalities. [514, 515]

No RCTs have been conducted on rewarming therapy for septic patients with hypothermia. The balance of benefits and harms of rewarming therapy may differ for each patient. Therefore, physicians need to assess whether or not rewarming therapy is necessary, considering the severity of hypothermia and rewarming-associated complications.

### CQ6-7: How is tracheal intubation performed for patients with sepsis?

*Answer*: Pathophysiological conditions for which tracheal intubation is indicated in patients with sepsis include shock and imbalance between oxygen demand and supply, in addition to airway obstruction and hypoxemia. Because sedatives and analgesics used during tracheal intubation may cause hemodynamic fluctuations, it is important to perform appropriate hemodynamic management, such as preparation of vasopressors (*Provision of information for background question*).

#### Rationale

Tracheal intubation and mechanical ventilation are required in 40–85% of patients with septic shock for a variety of reasons [516]. A previous review described that complications occurred in 45% of critically ill patients receiving tracheal intubation outside an operating room [517].

Indications for tracheal intubation is divided into problems in airway and gas exchange. Furthermore, insufficient oxygen supply relative to its demand, such as in patients with shock or circulatory failure, is also indicated because it has been suggested that oxygen supply to vital organs can be maintained by mechanical ventilation in patients with shock [518].

Physiological abnormalities, such as metabolic acidosis, are often present in sepsis. In these patinets, positive pressure ventilation itself can trigger circulatory collapse [517, 519]. Therefore, physiological abnormalities should also be considered in addition to anatomical factors during tracheal intubation for patients with sepsis [519]. Evaluation of the airway, adequate preoxygenation before tracheal intubation, and preparation of drugs and tracheal intubation devices are important in order to reduce complications associated with tracheal intubation [517].

Requiements of analgesics and sedatives during tracheal intubation is reduced in critically ill patients [520]. Clinicians should pay attention to hemodynamic and respiratory failure immediately after tracheal intubation [520]. A recent review shows that fluid loading and early introduction of vasopressors together may decrease the occurrence of intubation-related hemodynamic complications [521].

First-attempt failure was reported to be a contributing factor to periprocedural complications and death in tracheal intubation [521]. Methods for improving the successful intubation rates and reducing the incidence of difficult intubations include the use of stylet [522] and video laryngoscopes. To obtain the cooperation of physicians who are skilled in tracheal intubation is also important in order to safely and reliably perform tracheal intubation in patients with sepsis.

### FRQ6-1: Is IVIG administered for patients with STSS?

#### Rationale

Streptococcal toxic shock syndrome or severe invasive streptococcal infection can progress rapidly to hypotension and multiple organ failure. It has a high mortality rate of approximately 40% [523, 524], with the majority of deaths occurring within a few days after onset [525]. However, STSS is caused by the exotoxin produced by group A *Streptococcus*. IVIG, which has the effect of neutralizing toxins and suppressing cytokine production, may improve the clinical conditions of STSS. [526]

A systematic review analyzing one published RCT [399] and several observational studies showed that the administration of IVIG was associated with improved prognosis [408, 409]. In the J-SSCG 2020, we performed an analysis limited to adult patients with STSS and obtained similar results [8, 9]. However, there are some negative opinions about the administration of IVIG for the low certainty of evidence in the aforementioned systematic review and different titer of neutralizing antibodies for each IVIG formulation [524]. Additionally, there are no clear administration protocols regarding IVIG dosage. In one RCT, 1 g/kg was administered on the 1st day of treatment, followed by 0.5 g/kg on the second and third days [399]. Recently, the administration of 25 g IVIG per dose has been reported to be effective in neutralizing toxins, and a protocol of administering 0.5 g/kg on the 1st day of treatment, followed by 25 g on the second and third days, has been proposed [527].

## CQ7 Post-intensive care syndrome

See Fig. 7.

**Fig. 7:**
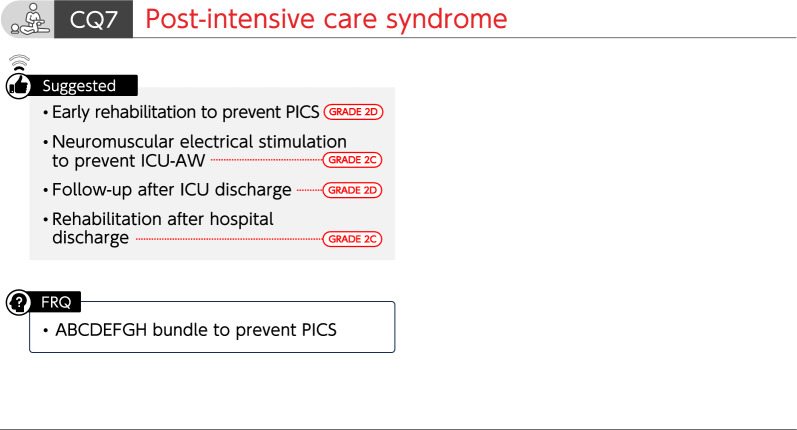
Summary of recommendations (CQ7 Post-intensive care syndrome). *CQ* clinical question, *FRQ* future research question, *ICU-AW* intensive care unit- acquired weakness, *PICS* post-intensive care syndrome

### CQ7-1: Is early rehabilitation implemented to prevent PICS?

*Answer*: We suggest conducting early rehabilitation to prevent PICS (GRADE 2D).

#### Rationale

Early rehabilitation can prevent PICS in patients admitted into an ICU. However, the benefit and harm of early rehabilitation for patients with sepsis have not been established. Additionally, there is no consensus regarding its definition and details for intervention. The J-SSCG 2020 suggested early rehabilitation for the prevention of PICS in patients with sepsis and those that are critically ill [8, 9]. Based on subsequent findings, the present CQ examined the efficacy of early rehabilitation in preventing PICS.

We conducted a meta-analysis of five RCTs evaluating the effect of early rehabilitation [528–532]. The SMD in muscle strength after discharge was 0.16 higher (95% CI 0.08 lower to 0.40 higher), and the MD in cognitive function after discharge was 0.6 higher (95% CI 0.25 lower to 1.45 higher). Additionally, the MD in mental function after discharge was 0.3 high (95% CI 4.92 lower to 5.52 higher), and the SMD in activity of daily living after discharge was 0.57 high (95% CI 0.1 higher to 1.05 higher). Any adverse events yielded an RD of 7 fewer per 1000 (95% CI 58 fewer to 124 more), but short-term mortality yielded an RD of 11 more per 1000 (95%CI 36 fewer to 77 more), which was considered an undesirable effect. Considering the importance of short-term mortality, we concluded that the balance of effects was probably better for the intervention (Additional file 3).

### CQ 7–2: Is neuromuscular electrical stimulation used to prevent ICU-AW?

*Answer*: We suggest using neuromuscular electrical stimulation to prevent ICU-AW (GRADE 2C).

#### Rationale

Sepsis itself and the use of vasopressors are risk factors for developing ICU-acquired weakness (ICU-AW). Neuromuscular electrical stimulation is expected to be effective in preventing muscle weakness in critically ill patients. However, it is difficult to achieve effective muscle contraction through neuromuscular electrical stimulation in sepsis patients, patients requiring vasopressor drugs, and patients with edema [533], and its efficacy remains unclear. J-SSCG 2020 suggested against performing neuromuscular electrical stimulation to prevent ICU-AW in sepsis patients and critically ill patients [8, 9]. The present CQ examined the efficacy of neuromuscular electrical stimulation in preventing the development of ICU-AW and its effect on quality of life (QOL).

We conducted a meta-analysis of 15 RCTs evaluating the effect of neuromuscular electrical stimulation [534–548]. The incidence rate of ICU-AW at ICU discharge yielded an RD of 218 fewer per 1000 (95% CI 285 fewer to 117 fewer), and the MD in health-related QOL after ICU discharge was 0.2 higher (95% CI 0.03 lower to 0.43 higher). On the other hand, short-term mortality yielded an RD of 18 more per 1000 (95% CI 33 fewer to 79 more), and any adverse events yielded an RD of 10 more per 1000 (95% CI 20 fewer to 40 more). Considering the relative value of each outcome, we concluded that the balance of effects was probably better for the intervention (Additional file 3).

### CQ7-3: Is follow-up after ICU discharge be implemented to improve physical, cognitive, and mental functions?

*Answer*: We suggest conducting follow-up after ICU discharge to improve physical, cognitive, and mental functions (GRADE 2D).

#### Rationale

Many survivors who have been admitted to the ICU develop PICS, and they experience difficulties in returning to daily life and work due to physical, cognitive, and mental dysfunctions developed during ICU stay and after ICU discharge. Follow-up rounds for PICS after ICU discharge (PICS rounds) and follow-up outpatient visits after hospital discharge are aimed at improving physical, cognitive, and mental functions. However, evaluation of the benefit and harm of follow-up for patients with sepsis after ICU discharge has not been established.

We conducted a meta-analysis of three RCTs evaluating the effect of follow-up after ICU discharge [549–551]. The MD in physical function after hospital discharge was 15 lower (95% CI 25.41 lower to 4.59 lower), and the SMD in mental function (depression) after hospital discharge was 0 lower (95% CI 0.19 lower to 0.19 higher). There were no studies reporting any adverse events. On the other hand, the MD in cognitive function after hospital discharge was 0.3 lower (95% CI 1.35 lower to 0.75 higher), and the SMD in mental function (posttraumatic stress disorder, PTSD) after hospital discharge was 0.1 higher (95%CI 0.42 lower to 0.62 higher), which were considered undesirable effects. Considering the results of cognitive and mental functions after discharge, we concluded that the balance of effects was probably better for the intervention (Additional file 3). Follow-up methods after ICU discharge depending on the circumstances of each facility, as well as the establishment of selection criteria for target patients, are considered.

### CQ7-4: Is rehabilitation after hospital discharge implemented to improve physical, cognitive, and mental functions?

*Answer*: We suggest performing rehabilitation after hospital discharge to improve physical, cognitive, and mental functions (GRADE 2C).

#### Rationale

Many survivors who have been admitted to the ICU develop PICS, and they experience decreased QOL and poor long-term prognosis due to physical, cognitive, and mental dysfunctions developed during hospitalization and after hospital discharge. Enhanced rehabilitation after hospital discharge aims at improving physical, cognitive, and mental functions. However, benefit and harm of post-hospital discharge rehabilitation for sepsis patients have not been established.

We conducted a meta-analysis of nine RCTs evaluating the effect of performing rehabilitation after ICU discharge [552–560]. The SMD in physical function after hospital discharge was 0.17 higher (95% CI 0.17 lower to 0.52 higher), and the MD in cognitive function after hospital discharge was 3.5 higher (95% CI 1.56 higher to 5.44 higher). Also, the MD in mental impairment (depression) after hospital discharge was 0.24 lower (95% CI 3.53 lower to 3.05 higher). On the other hand, any adverse events yielded an RD of 29 more per 1000 (95% CI 2 more to 107 more). Based on these results, we concluded that the balance of effects was probably better for the intervention (Additional file 3).

Each facility needs to establish the selection criteria for target patients for post-hospital discharge intensive rehabilitation depending on the circumstances. Upon implementation, the timing, duration, intensity, duration, and frequency are stipulated by healthcare providers, depending on the circumstances of the patients.

### FRQ7-1: Is the ABCDEFGH bundle implemented to prevent PICS?

#### Rationale

It is often difficult to achieve a complete cure of PICS in its natural course, and thus, its prevention and early intervention are crucial. Experts proposed the ABCDEFGH bundle for the prevention of PICS (Table 5). The ABCDEFGH bundle is a concept in which “FGH” to reduce PICS or PICS-F has been added to the ABCDE bundle proposed in 2010 to comprehensively improve the management of mechanically ventilated patients [561–563]. Large-scale multicenter observational studies of critically ill adult patients have reported that a high rate of adherence to the ABCDEF bundle is associated with decreases in hospital mortality and delirium incidence [564, 565]. At present, no clinical studies have evaluated the effectiveness of PICS/PICS-F prevention in the entire ABCDEFGH bundle as outcomes. Studies on the effectiveness of the ABCDEFGH bundle, which incorporates comprehensive prevention of PICS including post-ICU discharge, are needed.

**Table 5:** ABCDEFGH bundle

A: Assess, prevent, and manage pain	To understand pain and use tools for its assessment, treatment, and prevention
B: Both SAT and SBT	To use spontaneous awakening trial and spontaneous breathing trial in combination
C: Choice of analgesia and sedation	To understand the importance of depth of sedation and select appropriate drugs
D: Delirium: Assess, prevent, and manage	To understand risk factors for delirium and use tools for their assessment, treatment, and prevention
E: Early mobility and exercise	Early mobilization and rehabilitation in the ICU do not just change the body position of patients
F: Family engagement and empowerment, Follow-up referrals, Functional reconciliation	Patients' recovery can be facilitated by engaging family members in their care To make follow-up referrals and functional reconciliation
G: Good handoff communication	To establish good handoff communication
H: Hand the patients and family written information about PICS or PICS-F	To provide patients and their families with written information regarding PICS and PICS-F

Created from references. [561–563, 566]

*ICU* intensive care unit, *PICS* post-intensive care syndrome, *PICS-F* post-intensive care syndrome family, *SAT* spontaneous awakening trial, *SBT* spontaneous breathing trial

## CQ8 Patient and family care

See Fig. 8.

**Fig. 8:**
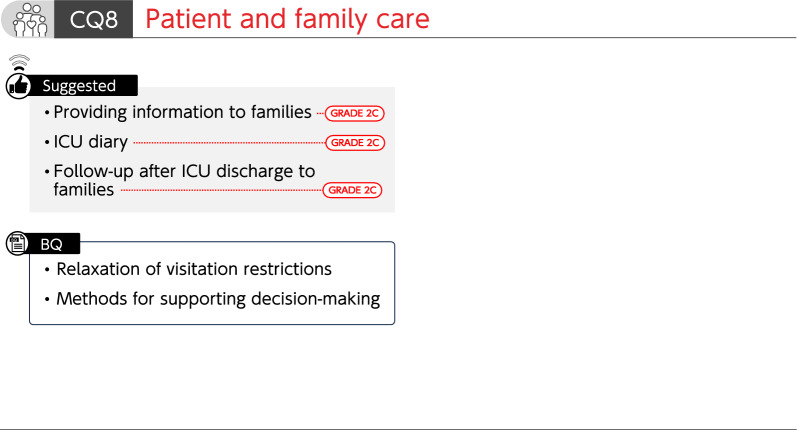
Summary of recommendations (CQ8 Patient and family care). *BQ* background question, *CQ* clinical question, *ICU* intensive care unit

### CQ 8–1: Is written information provided to the families of critically ill patients?

*Answer*: We suggest providing information related to intensive care to the families of critically ill patients in written or other forms (GRADE 2C).

#### Rationale

Many families of critically ill patients treated in an ICU have trouble in understanding their patients’ conditions due to unfamiliar medical information and inadequate communication with healthcare providers. Several studies have suggested that written information provision to the families of critically ill patients, in addition to verbal explanations by healthcare providers, is associated with a reduced psychological symptom of the families and improvement of their satisfaction and understanding [567, 568]. However, its effects have not yet been validated.

We conducted a meta-analysis of six RCTs [567–572]. As a result of written information provision related to intensive care to families of critically ill patients, the SMD in families' anxiety was 0.27 lower (95% CI 0.68 lower to 1.13 higher), the SMD in families' depression was 0.23 lower (95% CI 0.54 lower to 0.08 higher), and the MD in families’ stress disorder was 9.39 lower (95% CI 13.47 lower to 5.3 lower). The MD in families’ satisfaction (a lower value indicates a higher satisfaction) was 1.26 lower (95% CI 2.35 lower to 0.17 lower). The families' understanding yielded a RD of 295 more per 1000 (95% CI 142 more to 479 more). There were no reports of adverse events resulting from the information provision. Based on these results, we concluded that the balance of effects was better for the intervention (Additional file 3).

In the RCTs included in this meta-analysis, patients’ severity and the methods of information provision varied. Therefore, it is necessary to confirm the patients' and their families’ values and consider the compliance of families and the methods of information provision before implementing the intervention.

### CQ 8–2: What is the relaxation of visitation restrictions for families of critically ill patients?

*Answer*: Relaxation of visitation restrictions for families of critically ill patients include unrestricted visiting hours or numbers of visitors and online visitation. There is an opinion that it may be effective in preventing PICS-F. Its necessity should be considered depending on the situation at one’s own facility and individual cases (*Provision of information for background question*).

#### Rationale

Visitation restriction is necessary in preventing the spread of infection, ensuring the rest and safety of patients, improving the work efficiency of healthcare providers, and protecting privacy [573, 574]. In contrast, visitation restrictions may make it difficult for family members to obtain information about patients and pose a hindrance on patient and family members-centered care, resulting in increased risk of developing PICS-F. To solve this problem, relaxation of visitation restrictions has been proposed. Large-scale RCTs of adult ICU patients have shown that relaxation of visitation restrictions based on the provision of appropriate information can be implemented without increasing the incidence of infection or burnout rate of healthcare providers, and that it might reduce the anxiety of family members and increase their satisfaction level [575, 576]. A recent meta-analysis showed that relaxation of visitation restrictions was associated with decreased incidence of delirium in patients and shorter length of ICU stay without an increasing risk of infection [577]. A large-scale retrospective observational study reported that the incidence of mental disorders in patients during the first year after hospital discharge decreased by 21% with in-person family visits. [578]

Since the COVID-19 pandemic, strict restrictions, including prohibitions for visiting, have been implemented. Relaxation of visitation restrictions is fraught with more complex issues than ever. Assessment regarding whether or not to allow visitation is carefully made from various perspectives, such as the risk of epidemiology of infectious diseases, burden on healthcare providers, and protection of patient privacy. It is important to decide how to not to restrict visitation based on the social situation, policies and circumstances of their own facility, and patients' conditions.

### CQ 8–3: What are the methods for supporting decision-making that respect the value systems and ways of thinking in a patient?

*Answer*: There are methods of supporting decision-making that respect the values systems and ways of thinking of a patient through repeated discussions at multidisciplinary conferences involving patients and their families. One of the methods proposed is careful estimation through surrogate decision makers (e.g., family members) when the intentions of a patients are unclear. While respecting the intentions of patients, appropriate medical information is provided to patients and their families (*Provision of information for background question*).

#### Rationale

Decision-making support is becoming increasingly important with the increasing complexity of medical care and diversification of values, views, and lifestyles of patients. Emphasis has been placed on respecting patients' right to know, right of self-determination, and principles of autonomy, and shared decision making and advance care planning (ACP) have been proposed. Shared decision making is a concept in which patients, their families, and acquaintances and friends whom the patient trusts in making decisions proceed with decision-making together with healthcare providers. Decisions are made through a continuous two-way process; healthcare providers present accurate information that serves as evidence for the patient's condition and treatment options/methods, while the patient and patient’s family provide information about the patient's own values and wishes. They organize medical facts, have discussions at multidisciplinary conferences based on decisions made by the patient him/herself, and decide on the best policy for the patient [579]. ACP is important in this decision-making process; in order to provide information, including the values/wishes of the patient and medical care that the patient desires, it is necessary for a patient and his/her family to have an advance discussion in anticipation of emergencies, that is, ACP. When the intentions of a patient cannot be confirmed, surrogate intention-estimating individuals, such as family members, are carefully identified, and the best policy for the patient is taken while respecting the estimated intentions of the patient based on ACP. Shared decision making using ACP may reduce stress, depression, and anxiety in families after bereavement [567, 580]. These methods are not perfect even when a decision is made, and they are repeated over time, depending on changes in clinical course including patient's physical and mental conditions, and prognostication. The contents of each discussion during this process are summarized and recorded in medical records. [579]

### CQ 8–4: Is an ICU diary kept for critically ill patients?

*Answer*: We suggest keeping an ICU diary for critically ill patients (GRADE 2C).

#### Rationale

Critically ill patients treated in the ICU often have consciousness disorders or are under sedation due to their severe conditions. In the ICU, critically ill patients develop memory loss or delusional memories, in which events that did not actually occur are recalled as vivid memories. An ICU diary is an intervention that assists in the correct organization and reconstruction of memories by having healthcare providers, family members, and other individuals write a diary about the patient's daily situations in the ICU and hand over the diary to the patient after achieving recovery. Multiple studies have shown that ICU diary is associated with reductions in stress disorders, anxiety, and depression symptoms in critically ill patients and their families. However, its effectiveness and adverse events have not yet been validated.

We conducted a meta-analysis of six RCTs [581–586]. As a result of keeping an ICU diary for critically ill patients, the SMD in the level of stress disorder in patients was 0.13 lower (95% CI 0.32 lower to 0.06 higher), and the MD in the level of anxiety was 1.15 lower (95% CI 2.59 lower to 0.28 higher). Additionally, the MD in the level of depression was 0.39 lower (95% CI 1.06 lower to 0.28 higher) (a lower value in each indicates a milder symptom). Although anxiety in family members yielded an RD of 58 more per 1000 (95% CI 43 fewer to 191 more), depression yielded an RD of 19 fewer per 1000 (95% CI 104 fewer to 80 more), and an SMD in the level of stress disorder was 0.09 lower (95% CI 0.29 lower to 0.11 higher). There were no reports of adverse events resulting from applying an ICU diary. Based on these, we concluded that the balance of effects was probably better for the intervention (Additional file 3).

The RCTs included in the analysis had diverse target patients and families, as well as varying methods of ICU diary entries, such as the person making entries, entry method, timing, and duration. It is important to confirm the wishes of patients and their families prior to intervention and consider whether and how to provide the intervention.

### CQ 8–5: Is follow-up after ICU discharge provided to families of critically ill patients to improve their mental health?

*Answer*: In facilities with well-established systems, we suggest providing follow-ups, such as face-to-face, phone, and online interviews after ICU discharge, to families of critically ill patients to improve their mental health (GRADE 2C).

#### Rationale

The PICS-F is a mental disorder that occurs in family members of critically ill patients when the patient is staying in the ICU, has been discharged from the ICU, or has passed away. Multiple studies have suggested that providing follow-up visits to family members of critically ill patient after the patient's discharge from the ICU is associated with a reduction in the psychological symptoms of family members and improvement in their QOL. However, its effectiveness and adverse events have not yet been validated.

We conducted a meta-analysis of eight RCTs [587–594]. As a result of providing follow-ups to the families of critically ill patients, such as face-to-face, phone, and online interviews after ICU discharge, the SMD in the level of family's depression was 0.03 higher (95% CI 0.09 lower to 0.15 higher). However, the SMD in the level of anxiety was 0.03 lower (95% CI 0.15 lower to 0.09 higher), and the SMD in the level of stress disorder was 0.01 lower (95% CI 0.14 lower to 0.11 higher). The SMD in family's mental-related QOL was 0.06 lower (95% CI 0.3 lower to 0.18 higher), and the SMD in overall health-related QOL was 0.11 lower (95% CI 0.35 lower to 0.13 higher). There were no reports of adverse events resulting from the provision of follow-ups. As a result of examination with a focus on anxiety, depression, and stress disorders of families, we concluded that the balance of effects was probably better for the intervention (Additional file 3).

The RCTs included in the analysis had diverse targets and intervention methods, and interventions are expected to increase the workload of healthcare providers. Some interventions require families to pay their own medical expenses. Upon implementation, it is important to take into account the systems of one's own facility, confirm the wishes of a family in advance, and consider the content and implementation period of the follow-up.

## CQ9 Pediatrics

See Fig. 9.

**Fig. 9:**
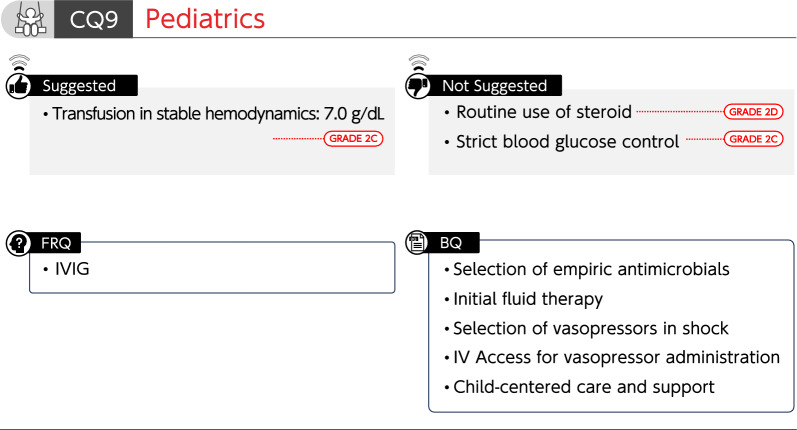
Summary of recommendations (CQ9 Pediatrics). *BQ* background question, *CQ* clinical question, *FRQ* future research question, *IV* intravenous, *IVIG* intravenous immunoglobulin

### Emergence of new diagnostic criteria for pediatric sepsis and septic shock: the Phoenix Sepsis Score

In 2016, the definition of sepsis in adult patients was revised to “sepsis-3,” which focuses on infection-associated organ dysfunction. The problems encountered upon creating the “sepsis-3” criteria were; (1) internal validity was tested based only on reports from high- and middle-income countries; and (2) no decision was made on which indicators should be used to evaluate organ dysfunction in children. For these reasons, it has been considered inappropriate to directly apply “sepsis-3” to pediatric sepsis patients [1].

New sepsis diagnostic criteria for pediatric patients, called the Phoenix Sepsis Score (Table 6), was published in January 2024 [595, 596]. The Phoenix Sepsis Score assigns a score, ranging from 0 to 3, to each of four organ functions (respiratory, cardiovascular, coagulation, and neurological) in pediatric patients suspected of having an infection within 24 h of hospitalization. Among patients suspected of having an infection, those with the Phoenix Sepsis Score of ≥ 2 points are defined as having sepsis. Among pediatric patients with sepsis, those having cardiovascular dysfunction with the Phoenix Sepsis Score of ≥ 1 cardiovascular point are defined as having septic shock. The J-SSCG 2024 did not use the Phoenix Sepsis Score as a definition of sepsis, as it was published during the preparation of the J-SSCG2024. However, the Phoenix Sepsis Score is expected to be widely used as a new definition of pediatric sepsis in the future.

**Table 6:** Phoenix Sepsis Score

Variables	0 Points	1 Point	2 Points	3 Points
Respiratory (0–3 points)				
	PaO_2_/F_I_O_2_ ratio ≥ 400 or SpO_2_/F_I_O_2_ ≥ 292^a^	PaO_2_/F_I_O_2_ ratio < 400 on any respiratory support or SpO_2_/F_I_O_2_ ratio < 292 on any respiratory support^a,b^	PaO_2_/F_I_O_2_ ratio 100–200 and IMV or SpO_2_/F_I_O_2_ ratio 148–220 and IMV^a^	PaO_2_/F_I_O_2_ ratio < 100 and IMV or SpO_2_/F_I_O_2_ ratio < 148 and IMV^a^
Cardiovascular (0–6 points)				
		1 point each (up to 3) for:	2 points each (up to 6) for:	
	No vasoactive medications^c^	1 vasoactive medications^c^	≥ 2 vasoactive medications^c^	
	Lactate < 5 mmol/L^d^	Lactate 5–10.9 mmol/L^d^	Lactate ≥ 11 mmol/L^d^	
Mean arterial pressure by age, mmHg^e,f^				
< 1 month	> 30	17–30	< 17	
1–11 months	> 38	25–38	< 25	
1 to < 2 years	> 43	31–43	< 31	
2 to < 5 years	> 44	32–44	< 32	
5 to < 12 years	> 48	36–48	< 36	
12 to < 17 years	> 51	38–51	< 38	
Coagulation (0–2 points)^g^				
		1 point each (maximum of 2 points) for:		
	Platelets ≥ 100 × 10^3^/μL	Platelets < 100 × 10^3^/μL		
	International normalized ratio ≤ 1.3	International normalized ratio > 1.3		
	D-dimer ≤ 2 mg/L FEU	D-dimer > 2 mg/L FEU		
	Fibrinogen ≥ 100 mg/dL	Fibrinogen < 100 mg/dL		
Neurological (0–2 points)^h^				
	Glasgow Coma Scale score > 10; pupils reactive^i^	Glasgow Coma Scale score ≤ 10^i^	Fixed pupils bilaterally	
Phoenix sepsis criteria				
Sepsis	Suspected infection and Phoenix Sepsis Score ≥ 2 points			
Septic shock	Sepsis with ≥ 1 cardiovascular point(s)			

The Phoenix Sepsis Score may be calculated in the absence of some variables (e.g., even if lactate level is not measured and vasoactive medications are not used, a cardiovascular score can still be ascertained using blood pressure). It is expected that laboratory tests and other measurements will be obtained at the discretion of the medical team based on clinical assessment. Unmeasured variables contribute no points to the score. Ages are not adjusted for prematurity, and the criteria do not apply to birth hospitalizations, neonates whose postconceptional age is < 37 weeks, or those aged ≥ 18 years

*FEU*, fibrinogen equivalent units, *F*_*I*_*O*_*2*_, fraction of inspired oxygen ratio, *IMV* invasive mechanical ventilation, *INR* international normalized ratio of prothrombin time, *MAP* mean arterial pressure, *PaO*_*2*_, arterial partial pressure of oxygen, *SpO*_*2*_, oxygen saturation measured by pulse oximetry (only SpO_2_ of ≤ 97%)

^a^SpO_2_/F_I_O_2_ is only calculated if SpO_2_ is ≤ 97% or

^b^Respiratory dysfunction of 1 point can be assessed in any patient receiving oxygen, high-flow, non-invasive positive pressure, or IMV respiratory support, and includes a PaO_2_/F_I_O_2_ ratio of < 200 and SpO_2_/F_I_O_2_ ratio of < 220 in children who are not receiving IMV. For children receiving IMV with a PaO_2_/F_I_O_2_ ratio of < 200 and SpO_2_/F_I_O_2_ ratio of < 220, see the criteria for 2 and 3 points

^c^Vasoactive medications include any dose of epinephrine, norepinephrine, dopamine, dobutamine, milrinone, and/or vasopressin (for shock)

^d^Lactate reference range between 0.5 and 2.2 mmol/L. Lactate can be arterial or venous

^e^Age is not adjusted for prematurity, and the criteria do not apply to birth hospitalizations, children whose postconceptional age is < 37 weeks, or those aged ≥ 18 years

^f^Use measured MAP preferentially (invasive arterial if available or non-invasive oscillometry), and if measured MAP is not available, a calculated MAP (1/3 × systolic + 2/3 × diastolic) may be used as an alternative

^g^Coagulation variable reference ranges: platelets, 150–450 × 10^3^/μL; D-dimer, < 0.5 mg/L FEU; fibrinogen, 180–410 mg/dL. The INR reference range is based on the local reference prothrombin time

^h^Neurological dysfunction sub score was pragmatically validated in both sedated and non-sedated patients, and those receiving or not receiving IMV support

^i^The Glasgow Coma Scale score measures the level of consciousness based on verbal, eye, and motor response (range, 3–15, with a higher score indicating better neurological function)

### CQ 9–1: How are empiric antimicrobials selected for pediatric septic shock?

*Answer*: Antimicrobials for all possible microorganisms are selected, taking into account the organ of infection, setting (community, hospital, or ICU), and patient background (e.g., immune status and antimicrobial prescription history) (*Provision of information for background question*).

#### Rationale

Identification of infection focus is important in the treatment of pediatric sepsis, and it allows us to target causative microorganisms based on past epidemiological information. In pediatric patients with sepsis, a causative infection focus is often found in the respiratory or urinary tract system, and other possible locations include the abdominal cavity, skin/soft tissue, and central nervous system [597–599].

If an infection focus can be identified based on medical history, physical findings, and various tests, we can estimate the causative microorganism based on patient's age, settings, and patient background, and select antimicrobials based on tissue penetration and antimicrobial spectrum.

If an infectious focus cannot be identified, empiric antimicrobials can be selected taking into consideration factors, such as age, settings, patient background, and tissue penetration. If the infectious focus is unknown in community-acquired pediatric sepsis patients, it is often found in the respiratory system, urinary tract system, or abdominal cavity [597], and causative microorganism can be *Staphylococcus aureus*, or Enterobacteriaceae (such as *Escherichia coli*) [600]. The causative microorganism in patients with nosocomial sepsis can also be glucose-non-fermentative bacteria, such as *Pseudomonas aeruginosa* and *Acinetobacter species*, in addition to Enterobacteriaceae, among other Gram-negative bacilli. It is noted that these must be affected by regional epidemiology or public health situation [601]. Additionally, patients with an underlying disease have an increasing risk of sepsis caused by methicillin-resistant *Staphylococcus aureus*, *Pseudomonas aeruginosa*, *Clostridioides difficile*, or fungi [602, 603]. Empiric antimicrobials effective to these microorganisms are selected considering the individual patient's background, risk of antibiotic-resistant pathogens, and severity of illness.

### CQ 9–2: How is initial fluid therapy administered for pediatric sepsis?

*Answer*: Methods of administering initial fluid therapy to pediatric sepsis include repeated administration of balanced crystalloid solutions, as a 10–20 mL/kg bolus, while evaluating response to therapy. Clinical findings suggestive of fluid overload or poor response to fluid administration can serve as discontinuing fluid therapy. In particular, attention is paid to the amount and rate of bolus administration in patients complicated by heart failure. We cannot provide information regarding the speed of fluid administration or upper limit of total fluid volume (*Provision of information for background question*).

#### Rationale

In patients with sepsis complicated by tissue hypoperfusion or decreased blood pressure, initial fluid therapy is important for preventing the progression of organ dysfunction. In initial fluid therapy, a 20 mL/kg bolus of a modified crystalloid solution is first administered, which is then increased up to 40–60 mL/kg in the first hour until normal perfusion and blood pressure are achieved while monitoring for signs of fluid overload [61]. Previous high-quality studies that focused on the dosage and administration rate of initial fluids for pediatric patients with sepsis were small-scale. No significant difference in the mortality rate has been observed among different dosages and administration rates of fluid administration. Although there is no evidence that can be used for the recommendation on the superiority of saline or balanced crystalloids, the SSCG in Children 2020 suggested using balanced crystalloids [604]. When administering initial fluid therapy, responsiveness to fluid administration is frequently evaluated, and the speed of fluid administration and vasopressors are titrated. The effectiveness of initial fluid therapy is evaluated as needed using capillary refill time, lactate levels, and echocardiogram. If a patient exhibits insufficient response or signs of fluid overload, fluid loading is discontinued, and the use of vasopressors is considered.

### CQ 9–3: How are vasopressors selected for pediatric patients with septic shock?

*Answer*: Adrenaline or noradrenaline is used as vasopressors in pediatric patients with septic shock, according to physical findings, hemodynamic parameters, and echocardiographic findings (*Provision of information for background question*).

#### Rationale

It is reasonable to select noradrenaline in patients presenting with vasodilatory shock. Dopamine has a weaker α-receptor-stimulating effect than noradrenaline, and there is also a concern about an immunosuppressive effect due to the suppression of prolactin secretion via dopamine receptors. There is insufficient evidence for using dopamine as the first-line vasopressor, compared to adrenaline [605, 606]. Vasopressin exerts a pressor effect through a mechanism different from that of catecholamines [607, 608]. The responsiveness to first-line vasopressor, such as noradrenaline and adrenaline, is evaluated individually, and the additional use of vasopressin is considered. Physical findings, hemodynamic parameters, and echocardiogram should be comprehensively and repeatedly evaluated for each case when considering support with noradrenaline or adrenaline.

### CQ 9–4: What is the route of administering vasopressors for pediatric sepsis?

*Answer*: Vasopressors are generally administered via the central venous line, as they may cause tissue injury when extravasation occurs. However, vasopressors are administered via a peripheral venous line or intraosseous access at appropriate concentrations for short periods to avoid delays in initiating the administration (*Provision of information for background question*).

#### Rationale

In the management of pediatric sepsis, prompt initiation of vasopressor administration is important for those who are unresponsive to initial fluid resuscitation. The administration of vasopressors through the peripheral venous line has the risk of developing extravasation, secondary tissue injury, and local perfusion deficits [609]. For this reason, they are generally administered through the central venous line [610]. However, placement of a central venous line requires time, leading to a delay in starting the administration of vasopressors. Some case series of children have suggested that the administration of vasopressors through a peripheral venous line is safe at appropriate concentrations for short periods [609, 611]. A recent meta-analysis of adults and children found a very low incidence of extravasation or no serious events in children administered vasopressors through the peripheral venous line [612]. The relationship between the concentration of administered vasopressor and the incidence of extravasation is unclear.

### CQ 9–5: Are steroids administered to pediatric patients with septic shock who are unresponsive to initial fluid therapy and vasopressors?

*Answer*: We suggest against routine administration of steroids for pediatric patients with septic shock who are unresponsive to initial fluid therapy and vasopressors (GRADE 2D).

#### Rationale

There has been a debate on the routine use of systemic steroids in pediatric patients with sepsis, and some high-quality studies have been published. We conducted meta-analyses of three RCTs [613–615]. With regard to the desirable effects of steroid administration, mortality yielded an RD of 57 fewer per 1000 (95% CI 161 fewer to 100 more), and duration until recovery from shock yielded an MD of 3.3 days shorter (95% CI 4.0 days shorter to 2.6 days shorter). In contrast, with regard to the undesirable effects of steroid administration, the length of hospital stay yielded an MD of 3.2 days longer (95% CI 0.13 days shorter to 6.5 days longer), and infectious complications yielded an RD of 40 more per 1000 (95% CI 68 fewer to 328 more). The desirable effects were small, and the undesirable effects were also small. Thus, we concluded that the balance of effects was neither intervention nor comparator was superior (Additional file 3).

### CQ 9–6: What is the optimal hemoglobin level for blood transfusion in pediatric patients with sepsis who have stable hemodynamics?

*Answer*: We suggest transfusing at a hemoglobin level of 7.0 g/dL in hemodynamically stable pediatric patients with sepsis (GRADE 2C).

#### Rationale

Children may easily develop anemia due to lower normal hemoglobin levels or greater effects of blood sampling compared to adults. Hemoglobin has an important role in oxygen transport, and RBC transfusion therapy has been one of the most important therapeutic options. In contrast, the choice of whether or not to administer blood transfusion therapy is crucial, considering the detrimental effects of excessive blood transfusion and complications, such as infections and allergic reactions, as well as long-term post-treatment effects. We conducted a meta-analysis of three RCTs [616–618]. With regard to the desirable effects of setting a relatively low hemoglobin level, which determines the implementation of transfusion, hospital mortality yielded an RD of 117 fewer per 1000 (95% CI 170 fewer to 22 fewer), new or progressive multiple organ dysfunction yielded an RD of 5 fewer per 1000 (95% CI 46 fewer to 55 more), length of ICU stay yielded an MD of 1.78 days shorter (95% CI 2.7 days shorter to 0.86 days shorter); and duration of mechanical ventilation yielded an MD of 1.02 days shorter (95% CI 1.77 days shorter to 0.27 days shorter). Concerning the undesirable effects, ICU mortality yielded an RD of 9 more per 1000 (95% CI 11 fewer to 57 more), and transfusion-related complications yielded an RD of 20 more per 1000 (95% CI 48 fewer to 97 more). The desirable effects were small, and the undesirable effects were trivial. Considering the relative value of each outcome, we concluded that the balance of effects was probably better for the intervention (Additional file 3).

### CQ 9–7: Is strict blood glucose control performed for pediatric sepsis?

*Answer*: We suggest against strict blood glucose control for pediatric sepsis (GRADE 2C).

#### Rationale

There are diverse opinions on the appropriateness of strict blood glucose control for pediatric patients with sepsis. The occurrence of hyperglycemia in severe pediatric patients may affect the immune function and exacerbate infections, leading to increased mortality and length of hospital stay. Hypoglycemia is a noteworthy harm of insulin therapy, and the occurrence of hypoglycemia is associated with worsened prognosis in critically ill patients. We conducted a meta-analysis of five RCTs [619–623]. With regard to the desirable effects of strict blood glucose control, the short-term mortality, which was the most important, yielded an RD of 2 more per 1000 (95% CI 10 fewer to 19 more), length of ICU stay yielded an MD of 0.51 days shorter (95% CI 0.53 days shorter to 0.49 days longer), and duration of mechanical ventilation yielded an MD of 0.30 days shorter (95% CI 0.32 days shorter to 0.28 days longer). With regard to the undesirable effect of strict blood glucose control, hypoglycemia yielded an RD of 146 more per 1000 (95% CI 108 more to 192 more). The desirable effects were trivial, and the undesirable effect were moderate. Thus, we concluded that the balance of effects was probably better for the comparator (Additional file 3).

### CQ 9–8: What are treatment and support policies centered on critically ill pediatric patients?

*Answer*: It is necessary to support the decision-making that prioritizes the benefits of affected children and respects the values and wishes of the affected children and their families.

A multidisciplinary team has a role in providing appropriate medical information. Actively creating an environment that allows family members to participate in care and support the decision-making process is essential, especially in pediatric patients (*Provision of information for background question*).

#### Rationale

In order to support the decision-making of critically ill pediatric patients and their families, it is essential for a multidisciplinary team to provide accurate medical information about the potential risks and benefits of treatment. When considering treatment policy, healthcare providers should develop a sufficient care plan for family members of a patient who are entrusted with decisions on medical treatment, while prioritizing the benefits and values of the affected child [567, 624]. There has been insufficient evidence on the optimal method for appropriately formulating care plans for critically ill pediatric patients and their families. It is necessary to formulate guidelines for each medical team and implement a comprehensive care plan centered on affected children. Its examples include having family members participate in medical team rounds, presenting information leaflets about the ICU to family members, introducing an ICU diary, engaging with family members in cooperation with multiple professions, working on noise reduction and environmental hygiene in the ICU, setting flexible or unrestricted family visits, and actively creating an environment that allows family members to spend time together. Healthcare providers aim to improve outcomes for pediatric patients and alleviate psychological burden on their families by prioritizing the improvement of the physiological conditions of affected children, formulating specific guidelines for family support, and supporting the decision-making process, while keeping in mind the particularity of pediatric medical care.

### FRQ9-1: Is IVIG administered for pediatric sepsis?

#### Rationale

Intravenous immunoglobulin is occasionally administered to severe infections despite its effectiveness in improving clinical prognosis remains unclear. Although some studies have attempted to administer it in high doses for the purpose of immunomodulation, the studies have yielded inconsistent results, and there is a lack of high-quality RCTs of pediatric patients excluding neonates [625–627]. There have been weak recommendations against its use in adult patients with sepsis [72] and its routine administration to pediatric patients [8, 9, 604]. The evaluation of the effectiveness and harmfulness of IVIG has not been established. IVIG, which is a plasma fraction preparation, is not inexpensive, and clarifying its clinical efficacy is of great significance. It is worthwhile to summarize the information about IVIG, given the high mortality for pediatric sepsis patients.

Intravenous immunoglobulin is expected to have a pharmacological effect of reducing inflammation by exerting an immunoregulatory action through the enhancement of passive immunity due to neutralization of pathogenic microorganisms and toxins and suppression of inflammatory mediators [8, 9, 604, 628].

However, high-quality studies on IVIG in pediatric patients with sepsis are scarce. One RCT investigated the administration of polyclonal IVIG in 100 pediatric patients with sepsis, and its administration decreased the hospital mortality rate (28% vs. 56%), length of pediatric ICU stay (6.1 days vs. 9.1 days), and complications (8% vs. 32%) [629]. However, it was a single-center, open-label RCT with a small sample size, no specification on the method of randomization, and the target population was limited to relatively young children (1 month to 2 years old). Thus, there were concerns, such as difficulty in extrapolating the study to all pediatric sepsis patients. The J-SSCG 2020 avoided creating a recommendation based only on this evidence [8, 9, 604]. The SSCG in Children 2020 states, “*We suggest against the routine administration of intravenous immunoglobulin to pediatric patients with septic shock or sepsis-related organ dysfunction (weak recommendation, low quality of evidence)*” [604].

The effectiveness of IVIG in adults is unfavorable, and the present guidelines, as well as the SSCG 2021, suggested against the administration of IVIG adult patients with sepsis [72]. Regarding neonates, a high-quality large-scale multicenter RCT conducted mainly on premature infants (the INIS trial) [630], as well as meta-analyses including the one for the RCT [625, 631], have clearly denied the treatment effect of IVIG on severe infections.

High-quality, large-scale multicenter RCTs of IVIG in pediatric patients are desired. When conducting the study, it is ideal that stratification is performed according to the type of infection (such as toxic shock syndrome or necrotizing fasciitis) and the presence or absence of comorbidities (such as hypogammaglobulinemia or immunodeficiency), resolving the uncertainty about the effectiveness of IVIG in each population [401, 408, 632].

## Supplementary Information

Additional file 1

Additional file 2

Additional file 3

Additional file 4

## Data Availability

The datasets used and analyzed for systematic reviews are available from the corresponding author on reasonable request.

## Abbreviations

ACP: Advance care planning
AI: Artificial intelligence
AKI: Acute kidney injury
AMR: Antimicrobial resistance
AUROC: Area under the receiver operating curve
Ca: Calcium
CI: Confidence interval
COVID-19: Severe acute respiratory syndrome coronavirus 2 infection
CRT: Capillary refill time
CQ: Clinical question
CMV: Cytomegalovirus
CRP: C-reactive protein
CRRT: Continuous renal replacement therapy
CRT: Capillary refill time
CT: Computed tomography
DI: Disagreement index
DIC: Disseminated intravascular coagulation
ER: Emergency room
ESBL: Extended-spectrum beta-lactamase
FDP: Fibrin degradation product
FRQ: Future research question
GRADE: Grading of Recommendations Assessment Development and Evaluation
GPS: Good practice statement
HELLP: Hemolysis elevated liver enzymes and low platelets
HSV: Herpes simplex virus
HIT: Heparin-induced thrombocytopenia
ICU: Intensive care unit
ICU-AW: ICU-acquired weakness
IgG: Immunoglobulin G
IL-6: Interleukin 6
IRRT: Intermittent renal replacement therapy
ISTH: International Society on Thrombosis and Hemostasis
IVIG: Intravenous immunoglobulin
JAAM: Japanese Association for Acute Medicine
JSICM: Japanese Society of Intensive Care Medicine
J-SSCG: Japanese Clinical Practice Guidelines for Management of Sepsis and Septic Shock
MAP: Mean arterial pressure
MD: Mean difference
MRI: Magnetic resonance imaging
MRSA: Methicillin-resistant *Staphylococcus aureus*
NEWS: National Early Warning Score
NMA: Network meta-analysis
PCT: Procalcitonin
PEWS: Pediatric patients’ pediatric early warning score
PICS: Post-intensive care syndrome
PICS-F: Post-intensive care syndrome family
PK/PD: Pharmacokinetics/pharmacodynamics
PMX-DHP: Polymyxin B-immobilized fiber column
P-SEP: Presepsin
PT: Prothrombin time
PTSD: Posttraumatic stress disorder
qSOFA: Quick sequential organ failure assessment
TDM: Therapeutic drug monitoring
TMA: Thrombotic microangiopathy
QOL: Quality of life
RCT: Randomized controlled trials
RD: Risk difference
RRT: Replacement therapy
ScvO_2_: Central venous oxygen saturation
SIC: Sepsis-induced coagulopathy
SIMD: Sepsis-induced myocardial dysfunction
SIRS: Systemic inflammatory response syndrome
SMD: Standardized mean difference
SOFA: Sequential organ failure assessment
SSCG: Surviving sepsis campaign guidelines
STSS: Streptococcal toxic shock syndrome
V-A ECMO: Veno-arterial extracorporeal membrane oxygenation

## References

[1] Singer M, Deutschman CS, Seymour CW, Shankar-Hari M, Annane D, Bauer M, et al. The third international consensus definitions for sepsis and septic shock (Sepsis-3). JAMA. 2016;315:801–10. 26903338 10.1001/jama.2016.0287 PMC4968574

[2] Sterne JAC, Savović J, Page MJ, Elbers RG, Blencowe NS, Boutron I, et al. RoB 2: a revised tool for assessing risk of bias in randomised trials. BMJ. 2019;366:l4898. 31462531 10.1136/bmj.l4898

[3] Sterne JA, Hernán MA, Reeves BC, Savović J, Berkman ND, Viswanathan M, et al. ROBINS-I: a tool for assessing risk of bias in non-randomised studies of interventions. BMJ. 2016;355:i4919. 27733354 10.1136/bmj.i4919 PMC5062054

[4] Bone RC, Balk RA, Cerra FB, et al. Definitions for sepsis and organ failure and guidelines for the use of innovative therapies in sepsis. The ACCP/SCCM Consensus Conference Committee. American College of Chest Physicians/Society of Critical Care Medicine. Chest. 1992;101:1644–55. 1303622 10.1378/chest.101.6.1644

[5] Levy MM, Fink MP, Marshall JC, Abraham E, Angus D, Cook D, Cohen J, Opal SM, Vincent JL, Ramsay G. 2001 SCCM/ESICM/ACCP/ATS/SIS international sepsis definitions conference. Intensive Care Med. 2003;29:530–8. 12664219 10.1007/s00134-003-1662-x

[6] Vincent JL, Opal SM, Marshall JC, Tracey KJ. Sepsis definitions: time for change. Lancet. 2013;381:774–5. 23472921 10.1016/S0140-6736(12)61815-7 PMC4535310

[7] Pittet D, Rangel-Frausto S, Li N, Tarara D, Costigan M, Rempe L, Jebson P, Wenzel RP. Systemic inflammatory response syndrome, sepsis, severe sepsis and septic shock: incidence, morbidities and outcomes in surgical ICU patients. Intensive Care Med. 1995;21:302–9. 7650252 10.1007/BF01705408

[8] Egi M, Ogura H, Yatabe T. The Japanese Clinical Practice Guidelines for Management of Sepsis and Septic Shock 2020 (J-SSCG 2020). J Intensive Care. 2021;9:53. 34433491 10.1186/s40560-021-00555-7 PMC8384927

[9] Egi M, Ogura H, Yatabe T. The Japanese Clinical Practice Guidelines for Management of Sepsis and Septic Shock 2020 (J-SSCG 2020). Acute Med Surg. 2021;8: e659. 34484801 10.1002/ams2.659 PMC8390911

[10] Vincent JL, Moreno R, Takala J. The SOFA (sepsis-related organ failure assessment) score to describe organ dysfunction/failure: On behalf of the working group on sepsis-related problems of the European society of intensive care medicine. Intensive Care Med. 1996;22:707–10. 8844239 10.1007/BF01709751

[11] Peake SL, Delaney A, Bailey M. Potential impact of the 2016 consensus definitions of sepsis and septic shock on future sepsis research. Ann Emerg Med. 2017;70:553–61. 28601273 10.1016/j.annemergmed.2017.04.007

[12] Maitra S, Som A, Bhattacharjee S. Accuracy of quick sequential organ failure assessment (qSOFA) score and systemic inflammatory response syndrome (SIRS) criteria for predicting mortality in hospitalized patients with suspected infection: a meta-analysis of observational studies. Clin Microbiol Infect. 2018;24:1123–9. 29605565 10.1016/j.cmi.2018.03.032

[13] Song JU, Sin CK, Park HK. Performance of the quick sequential (sepsis-related) organ failure assessment score as a prognostic tool in infected patients outside the intensive care unit: a systematic review and meta-analysis. Crit Care. 2018;22:28. 29409518 10.1186/s13054-018-1952-x PMC5802050

[14] Moreno R, Rhodes A, Piquilloud L. The sequential organ failure assessment (SOFA) score: has the time come for an update? Crit Care. 2023;27:15. 36639780 10.1186/s13054-022-04290-9 PMC9837980

[15] IDSA Sepsis Task Force. Infectious Diseases Society of America (IDSA) position statement: why IDSA did not endorse the surviving sepsis campaign guidelines. Clin Infect Dis. 2018;66:1631–5. 29182749 10.1093/cid/cix997 PMC6927848

[16] Kaukonen KM, Bailey M, Pilcher D. Systemic inflammatory response syndrome criteria in defining severe sepsis. N Engl J Med. 2015;372:1629–38. 25776936 10.1056/NEJMoa1415236

[17] Seymour CW, Liu VX, Iwashyna TJ. Assessment of clinical criteria for sepsis: for the third international consensus definitions for sepsis and septic shock (Sepsis-3). JAMA. 2016;315:762–74. 26903335 10.1001/jama.2016.0288 PMC5433435

[18] Royal College of Physicians. National Early Warning Score (NEWS) 2. https://www.rcplondon.ac.uk/projects/outputs/national-early-warning-score-news-2. Accessed 13 Feb 2025.

[19] Wang C, Xu R, Zeng Y. A comparison of qSOFA, SIRS and NEWS in predicting the accuracy of mortality in patients with suspected sepsis: a meta-analysis. PLoS ONE. 2022;17: e0266755. 35427367 10.1371/journal.pone.0266755 PMC9012380

[20] Parshuram CS, Dryden-Palmer K, Farrell C, Canadian Critical Care Trials Group, the EPOCH Investigators. Effect of a pediatric early warning system on all-cause mortality in hospitalized pediatric patients: the EPOCH randomized clinical trial. JAMA. 2018;319:1002–12. 29486493 10.1001/jama.2018.0948 PMC5885881

[21] van Nassau SC, van Beek RH, Driessen GJ. Translating sepsis-3 criteria in children: prognostic accuracy of age-adjusted quick SOFA score in children visiting the emergency department with suspected bacterial infection. Front Pediatr. 2018;6:266. 30327759 10.3389/fped.2018.00266 PMC6174358

[22] Fabre V, Sharara SL, Salinas AB. Does this patient need blood cultures? A scoping review of indications for blood cultures in adult nonneutropenic inpatients. Clin Infect Dis. 2020;71:1339–47. 31942949 10.1093/cid/ciaa039

[23] Coburn B, Morris AM, Tomlinson G. Does this adult patient with suspected bacteremia require blood culture? JAMA. 2012;308:502–11. 22851117 10.1001/jama.2012.8262

[24] Cheng MP, Stenstrom R, Paquette K. FABLED Investigators: blood culture results before and after antimicrobial administration in patients with severe manifestations of Sepsis: a diagnostic study. Ann Intern Med. 2019;171:547–54. 31525774 10.7326/M19-1696

[25] Cockerill FR, Wilson JW, Vetter EA. Optimal testing parameters for blood cultures. Clin Infect Dis. 2004;38:1724–30. 15227618 10.1086/421087

[26] Lee A, Mirrett S, Reller LB. Detection of bloodstream infections in adults: how many blood cultures are needed? J Clin Microbiol. 2007;45:3546–8. 17881544 10.1128/JCM.01555-07 PMC2168497

[27] Doern GV, Carroll KC, Diekema DJ. Practical guidance for clinical microbiology laboratories: a comprehensive update on the problem of blood culture contamination and a discussion of methods for addressing the problem. Clin Microbiol Rev. 2019;33:e00009-e00019. 31666280 10.1128/CMR.00009-19 PMC6822992

[28] Metlay JP, Waterer GW, Long AC. Diagnosis and treatment of adults with community-acquired pneumonia. An official clinical practice guideline of the American thoracic society and infectious diseases society of America. Am J Respir Crit Care Med. 2019;200:e45-67. 31573350 10.1164/rccm.201908-1581ST PMC6812437

[29] Kalil AC, Metersky ML, Klompas M. Management of adults with hospital-acquired and ventilator-associated pneumonia: 2016 clinical practice guidelines by the infectious diseases society of America and the American thoracic society. Clin Infect Dis. 2016;63:e61-111. 27418577 10.1093/cid/ciw353 PMC4981759

[30] Torres A, Niederman MS, Chastre J. International ERS/ESICM/ESCMID/ALAT guidelines for the management of hospital-acquired pneumonia and ventilator-associated pneumonia. Eur Respir J. 2017;50:1700582. 28890434 10.1183/13993003.00582-2017

[31] Bodilsen J, Dalager-Pedersen M, Schønheyder HC. Time to antibiotic therapy and outcome in bacterial meningitis: a Danish population-based cohort study. BMC Infect Dis. 2016;16:392. 27507415 10.1186/s12879-016-1711-z PMC4977612

[32] McGill F, Heyderman RS, Panagiotou S. Acute bacterial meningitis in adults. Lancet. 2016;388:3036–47. 27265346 10.1016/S0140-6736(16)30654-7

[33] Bijlsma MW, Brouwer MC, Kasanmoentalib ES. Community-acquired bacterial meningitis in adults in the Netherlands, 2006–14: a prospective cohort study. Lancet Infect Dis. 2016;16:339–47. 26652862 10.1016/S1473-3099(15)00430-2

[34] Tan M, Lu Y, Jiang H, Zhang L. The diagnostic accuracy of procalcitonin and C-reactive protein for sepsis: a systematic review and meta-analysis. J Cell Biochem. 2019;120:5852–9. 30417415 10.1002/jcb.27870

[35] Liu Y, Hou JH, Li Q. Biomarkers for diagnosis of sepsis in patients with systemic inflammatory response syndrome: a systematic review and meta-analysis. Springerplus. 2016;5:2091. 28028489 10.1186/s40064-016-3591-5 PMC5153391

[36] Kondo Y, Umemura Y, Hayashida K. Diagnostic value of procalcitonin and presepsin for sepsis in critically ill adult patients: a systematic review and meta-analysis. J Intensive Care. 2019;7:22. 31016020 10.1186/s40560-019-0374-4 PMC6466719

[37] Wu CC, Lan HM, Han ST. Comparison of diagnostic accuracy in sepsis between presepsin, procalcitonin, and C-reactive protein: a systematic review and meta-analysis. Ann Intensive Care. 2017;7:91. 28875483 10.1186/s13613-017-0316-z PMC5585118

[38] Ma L, Zhang H, Yin YL. Role of interleukin-6 to differentiate sepsis from non-infectious systemic inflammatory response syndrome. Cytokine. 2016;88:126–35. 27599258 10.1016/j.cyto.2016.08.033

[39] Brouwer MC, Tunkel AR, McKhann GM II. Brain abscess. N Engl J Med. 2014;371:447–56. 25075836 10.1056/NEJMra1301635

[40] Maroldi R, Farina D, Ravanelli M. Emergency imaging assessment of deep neck space infections. Semin Ultrasound CT MR. 2012;33:432–42. 22964409 10.1053/j.sult.2012.06.008

[41] Shen KR, Bribriesco A, Crabtree T. The American association for thoracic surgery consensus guidelines for the management of empyema. J Thorac Cardiovasc Surg. 2017;153:E129–46. 28274565 10.1016/j.jtcvs.2017.01.030

[42] Bedawi EO, Ricciardi S, Hassan M. ERS/ESTS statement on the management of pleural infection in adults. Eur Respir J. 2023;61:2201062. 36229045 10.1183/13993003.01062-2022

[43] Fowler VG, Durack DT, Selton-Suty C, Athan E, Bayer AS, Chamis AL, Dahl A, Bernardo L, Durante-Mangoni E, Duval X, Fortes C. The 2023 Duke-ISCVID criteria for infective endocarditis: updating the modified Duke criteria. Clin Infect Dis. 2023;77:518–26. 37138445 10.1093/cid/ciad271 PMC10681650

[44] Mayumi T, Yoshida M, Tazuma S. The practice guidelines for primary care of acute abdomen 2015. Jpn J Radiol. 2016;34:80–115. 26678269 10.1007/s11604-015-0489-z

[45] Kiriyama S, Kozaka K, Takada T. Tokyo Guidelines 2018: diagnostic criteria and severity grading of acute cholangitis (with videos). J Hepatobiliary Pancreat Sci. 2018;25:17–30. 29032610 10.1002/jhbp.512

[46] Tamburrini S, Lugarà M, Iannuzzi M. Pyonephrosis ultrasound and computed tomography features: a pictorial review. Diagnostics. 2021;11:331. 33671431 10.3390/diagnostics11020331 PMC7921924

[47] Stevens DL, Bisno AL, Chambers HF. Practice guidelines for the diagnosis and management of skin and soft tissue infections: 2014 update by the infectious diseases society of America. Clin Infect Dis. 2014;59:147–59. 24947530 10.1093/cid/ciu296

[48] Solomkin JS, Mazuski JE, Bradley JS. Diagnosis and management of complicated intra-abdominal infection in adults and children: guidelines by the surgical infection society and the infectious diseases society of America. Surg Infect. 2010;11:79–109. 10.1089/sur.2009.9930 20163262

[49] Mazuski JE, Tessier JM, May AK. The surgical infection society revised guidelines on the management of intra-abdominal infection. Surg Infect. 2017;18:1–76. 10.1089/sur.2016.261 28085573

[50] Ross JT, Matthay MA, Harris HW. Secondary peritonitis: principles of diagnosis and intervention. BMJ. 2018;361: k1407. 29914871 10.1136/bmj.k1407 PMC6889898

[51] The Revision Committee. JPN practice guidelines for the management of acute pancreatitis 2021. Tokyo: Kanehara & Co., Ltd.; 2021. p. 2021.

[52] Assimos D, Krambeck A, Miller NL. Surgical management of stones: American urological association/endourological society guideline. PART I J Urol. 2016;196:1153–60. 27238616 10.1016/j.juro.2016.05.090

[53] Nawijn F, Smeeing DPJ, Houwert RM. Time is of the essence when treating necrotizing soft tissue infections: a systematic review and meta-analysis. World J Emerg Surg. 2020;15:4. 31921330 10.1186/s13017-019-0286-6 PMC6950871

[54] Garnacho-Montero J, Aldabó-Pallás T, Palomar-Martínez M. Risk factors and prognosis of catheter-related bloodstream infection in critically ill patients: a multicenter study. Intensive Care Med. 2008;34:2185–93. 18622596 10.1007/s00134-008-1204-7

[55] Davies HE, Davies RJO, Davies CWH. Management of pleural infection in adults: British thoracic society pleural disease guideline 2010. Thorax. 2010;65:ii41. 20696693 10.1136/thx.2010.137000

[56] Colice GL, Curtis A, Deslauriers J. Medical and surgical treatment of parapneumonic effusions: an evidence-based guideline. Chest. 2000;118:1158–71. 11035692 10.1378/chest.118.4.1158

[57] Endo K, Mizuno K, Seki T. Intensive care unit versus high-dependency care unit admission on mortality in patients with septic shock: a retrospective cohort study using Japanese claims data. J Intensive Care. 2022;10:35. 35869538 10.1186/s40560-022-00627-2 PMC9306250

[58] Oami T, Imaeda T, Nakada T. Mortality analysis among sepsis patients in and out of intensive care units using the Japanese nationwide medical claims database: a study by the Japan sepsis Alliance study group. J Intensive Care. 2023;11:2. 36611188 10.1186/s40560-023-00650-x PMC9826578

[59] Ogura T, Nakamura Y, Takahashi K, Nishida K, Kobashi D, Matsui S. Treatment of patients with sepsis in a closed intensive care unit is associated with improved survival: a nationwide observational study in Japan. J Intensive Care. 2018;6:57. 30202529 10.1186/s40560-018-0322-8 PMC6122219

[60] Dellinger RP, Levy MM, Rhodes A. Surviving sepsis campaign: international guidelines for management of severe sepsis and septic shock, 2012. Intensive Care Med. 2013;39:165–228. 23361625 10.1007/s00134-012-2769-8 PMC7095153

[61] Davis AL, Carcillo JA, Aneja RK, Deymann AJ, Lin JC, Nguyen TC, et al. American College of Critical care medicine clinical practice parameters for hemodynamic support of pediatric and neonatal septic shock. Crit Care Med. 2017;45:1061–93. 28509730 10.1097/CCM.0000000000002425

[62] Fleuren LM, Klausch TLT, Zwager CL. Machine learning for the prediction of sepsis: a systematic review and meta-analysis of diagnostic test accuracy. Intensive Care Med. 2020;46:383–400. 31965266 10.1007/s00134-019-05872-y PMC7067741

[63] Shimabukuro DW, Barton CW, Feldman MD. Effect of a machine learning-based severe sepsis prediction algorithm on patient survival and hospital length of stay: a randomised clinical trial. BMJ Open Respir Res. 2017;4: e000234. 10.1136/bmjresp-2017-000234 PMC5687546 29435343

[64] Schinkel M, van der Poll T, Wiersinga WJ. Artificial intelligence for early sepsis detection: a word of caution. Am J Respir Crit Care Med. 2023;207:853–4. 36724366 10.1164/rccm.202212-2284VP PMC10111986

[65] Tu KJ, Wymore C, Tchangalova N. The impact of telehealth in sepsis care: a systematic review. J Telemed Telecare. 2023;24:38. 10.1177/1357633X231170038 PMC11187410 37093782

[66] Wenzel RP. The antibiotic pipeline—challenges, costs, and values. N Engl J Med. 2004;351:523–6. 15295041 10.1056/NEJMp048093

[67] Cassell GH, Mekalanos J. Development of antimicrobial agents in the era of new and reemerging infectious diseases and increasing antibiotic resistance. JAMA. 2001;285:601–5. 11176866 10.1001/jama.285.5.601

[68] WHO. Global action plan on antimicrobial resistance. 2015. http://apps.who.int/iris/bitstream/10665/193736/1/9789241509763_eng.pdf. Accessed 16 May 2023.

[69] Jones BE, Ying J, Stevens V, Haroldsen C, He T, Nevers M, et al. Empirical anti-MRSA vs standard antibiotic therapy and risk of 30-day mortality in patients hospitalized for pneumonia. JAMA Intern Med. 2020;180:552–60. 32065604 10.1001/jamainternmed.2019.7495 PMC7042818

[70] Kett DH, Cano E, Quartin AA, Mangino JE, Zervos MJ, Peyrani P, Improving Medicine through Pathway Assessment of Critical Therapy of Hospital-Acquired Pneumonia (IMPACT-HAP) Investigators, et al. Implementation of guidelines for management of possible multidrug-resistant pneumonia in intensive care: an observational, multicentre cohort study. Lancet Infect Dis. 2011;11:181–9. 21256086 10.1016/S1473-3099(10)70314-5

[71] Yoshimura J, Yamakawa K, Ohta Y, Nakamura K, Hashimoto H, Kawada M, et al. Effect of gram stain-guided initial antibiotic therapy on clinical response in patients with ventilator-associated pneumonia: the GRACE-VAP randomized clinical trial. JAMA Netw Open. 2022;5: e226136. 35394515 10.1001/jamanetworkopen.2022.6136 PMC8994124

[72] Evans L, Rhodes A, Alhazzani W. Surviving sepsis campaign: international guidelines for management of sepsis and septic shock 2021. Intensive Care Med. 2021;47:1181–247. 34599691 10.1007/s00134-021-06506-y PMC8486643

[73] Marik PE, Farkas JD, Spiegel R. POINT: should the surviving sepsis campaign guidelines be retired? Yes Chest. 2019;155:12–4. 30616719 10.1016/j.chest.2018.10.008

[74] Abe T, Kushimoto S, Tokuda Y. Implementation of earlier antibiotic administration in patients with severe sepsis and septic shock in Japan: a descriptive analysis of a prospective observational study. Crit Care. 2019;23:360. 31744549 10.1186/s13054-019-2644-x PMC6862854

[75] Althunayyan SM, Aljanoubi MA, Alghadeer SM. The impact of emergency antibiotic administration time on patients with sepsis. Saudi Med J. 2021;42:1002–8. 34470839 10.15537/smj.2021.42.9.20210447 PMC9280501

[76] Ascuntar J, Mendoza D, Jaimes F. Antimicrobials administration time in patients with suspected sepsis: is faster better? An analysis by propensity score. J Intensive Care. 2020;8:28. 32337048 10.1186/s40560-020-00448-1 PMC7178597

[77] Im Y, Kang D, Ko RE. Time-to-antibiotics and clinical outcomes in patients with sepsis and septic shock: a prospective nationwide multicenter cohort study. Crit Care. 2022;26:19. 35027073 10.1186/s13054-021-03883-0 PMC8756674

[78] Li A, Ling L, Qin H. Epidemiology, management, and outcomes of sepsis in ICUs among countries of differing national wealth across Asia. Am J Respir Crit Care Med. 2022;206:1107–16. 35763381 10.1164/rccm.202112-2743OC

[79] Tantarattanapong S, Hemwej T. Door-to-antibiotic time and in-hospital mortality of elder patients presenting to emergency department with sepsis; a cross-sectional study. Arch Acad Emerg Med. 2021;9: e44. 34223189 10.22037/aaem.v9i1.1266 PMC8221551

[80] Ferrer R, Artigas A, Suarez D. Effectiveness of treatments for severe sepsis: a prospective, multicenter, observational study. Am J Respir Crit Care Med. 2009;180:861–6. 19696442 10.1164/rccm.200812-1912OC

[81] Ferrer R, Martin-Loeches I, Phillips G. Empiric antibiotic treatment reduces mortality in severe sepsis and septic shock from the first hour: results from a guideline-based performance improvement program. Crit Care Med. 2014;42:1749–55. 24717459 10.1097/CCM.0000000000000330

[82] Gaieski DF, Mikkelsen ME, Band RA. Impact of time to antibiotics on survival in patients with severe sepsis or septic shock in whom early goal-directed therapy was initiated in the emergency department. Crit Care Med. 2010;38:1045–53. 20048677 10.1097/CCM.0b013e3181cc4824

[83] Puskarich MA, Trzeciak S, Shapiro NI. Association between timing of antibiotic administration and mortality from septic shock in patients treated with a quantitative resuscitation protocol. Crit Care Med. 2011;39(2):066–71. 10.1097/CCM.0b013e31821e87ab PMC3158284 21572327

[84] Yokota PK, Marra AR, Martino MD. Impact of appropriate antimicrobial therapy for patients with severe sepsis and septic shock—a quality improvement study. PLoS ONE. 2014;9: e104475. 25375775 10.1371/journal.pone.0104475 PMC4222820

[85] Paul M, Shani V, Muchtar E. Systematic review and metaanalysis of the efficacy of appropriate empiric antibiotic therapy for sepsis. Antimicrob Agents Chemother. 2010;54:4851–63. 20733044 10.1128/AAC.00627-10 PMC2976147

[86] Niederman MS, Baron RM, Bouadma L, Calandra T, Daneman N, DeWaele J, et al. Initial antimicrobial management of sepsis. Crit Care Med. 2021;25:307. 10.1186/s13054-021-03736-w PMC8390082 34446092

[87] Imaeda T, Nakada TA, Takahashi N. Trends in the incidence and outcome of sepsis using data from a Japanese nationwide medical claims database-the Japan sepsis Alliance (JaSA) study group. Crit Care Med. 2021;25:338. 10.1186/s13054-021-03762-8 PMC8444487 34530884

[88] Abe T, Ogura H, Kushimoto S. Variations in infection sites and mortality rates among patients in intensive care units with severe sepsis and septic shock in Japan. J Intensive Care. 2019;7:28. 31073407 10.1186/s40560-019-0383-3 PMC6500015

[89] Guarino M, Perna B, Cesaro AE. 2023 update on sepsis and septic shock in adult patients: management in the emergency department. J Clin Med. 2023;12:3188. 37176628 10.3390/jcm12093188 PMC10179263

[90] Rannikko J, Syrjänen J, Seiskari T. Sepsis-related mortality in 497 cases with blood culture-positive sepsis in an emergency department. Int J Infect Dis. 2017;58:52–7. 28288925 10.1016/j.ijid.2017.03.005

[91] Vakkalanka JP, Harland KK, Swanson MB. Clinical and epidemiological variability in severe sepsis: an ecological study. J Epidemiol Community Health. 2018;72:741–5. 29636401 10.1136/jech-2018-210501 PMC7027349

[92] van Vught LA, Klein Klouwenberg PMC, Spitoni C. Incidence, risk factors, and attributable mortality of secondary infections in the intensive care unit after admission for sepsis. JAMA. 2016;315:1469–79. 26975785 10.1001/jama.2016.2691

[93] Leligdowicz A, Dodek PM, Norena M. Association between source of infection and hospital mortality in patients who have septic shock. Am J Respir Crit Care Med. 2014;189:1204–13. 24635548 10.1164/rccm.201310-1875OC

[94] Li Y, Guo J, Yang H. Comparison of culture-negative and culture-positive sepsis or septic shock: a systematic review and meta-analysis. Crit Care Med. 2021;25:167. 10.1186/s13054-021-03592-8 PMC8106121 33964934

[95] Bochud P, Bonten M, Marchetti O. Antimicrobial therapy for patients with severe sepsis and septic shock: an evidence-based review. Crit Care Med. 2004;32:495–512. 15542958 10.1097/01.ccm.0000143118.41100.14

[96] Trouillet JL, Vuagnat A, Combes A. *Pseudomonas aeruginosa* ventilator-associated pneumonia: comparison of episodes due to piperacillin-resistant versus piperacillin-susceptible organisms. Clin Infect Dis. 2002;34:1047–54. 11914992 10.1086/339488

[97] Schmitt DV, Leitner E, Welte T. Piperacillin/tazobactam vs imipenem/cilastatin in the treatment of nosocomial pneumonia—a double blind prospective multicentre study. Infection. 2006;34:127–34. 16804655 10.1007/s15010-006-5020-0

[98] Joshi M, Metzler M, McCarthy M. Comparison of piperacillin/tazobactam and imipenem/cilastatin, both in combination with tobramycin, administered every 6 h for treatment of nosocomial pneumonia. Respir Med. 2006;100:1554–65. 16487695 10.1016/j.rmed.2006.01.004

[99] Erasmo AA, Crisostomo AC, Yan LN. Randomized comparison of piperacillin/tazobactam versus imipenem/cilastatin in the treatment of patients with intra-abdominal infection. Asian J Surg. 2004;27:227–35. 15564167 10.1016/S1015-9584(09)60039-7

[100] Klugman KP, Dagan R. Randomized comparison of meropenem with cefotaxime for treatment of bacterial meningitis. Meropenem meningitis study group. Antimicrob Agents Chemother. 1995;39:1140–6. 7625802 10.1128/aac.39.5.1140 PMC162697

[101] Schmutzhard E, Williams KJ, Vukmirovits G. A randomised comparison of meropenem with cefotaxime or ceftriaxone for the treatment of bacterial meningitis in adults. Meropenem meningitis study group. J Antimicrob Chemother. 1995;36:85–97. 8543502 10.1093/jac/36.suppl_a.85

[102] Howatt M, Klompas M, Kalil AC. Carbapenem antibiotics for the empiric treatment of nosocomial pneumonia: a systematic review and meta-analysis. Chest. 2021;159:1041–54. 33393468 10.1016/j.chest.2020.10.039

[103] Tamma PD, Rodriguez-Bano J. The use of noncarbapenem β-lactams for the treatment of extended-spectrum β-lactamase infections. Clin Infect Dis. 2017;64:972–80. 28362938 10.1093/cid/cix034 PMC5848369

[104] Harris PNA, Tambyah PA, Lye DC. Effect of piperacillin-Tazobactam vs Meropenem on 30-day mortality for patients with *E. coli* or *Klebsiella pneumoniae* bloodstream infection and ceftriaxone resistance: a randomized clinical trial. JAMA. 2018;320:984–94. 30208454 10.1001/jama.2018.12163 PMC6143100

[105] Marchaim D, Kaye KS, Fowler VG. Case–control study to identify factors associated with mortality among patients with methicillin-resistant *Staphylococcus aureus* bacteraemia. Clin Microbiol Infect. 2010;16:747–52. 19723135 10.1111/j.1469-0691.2009.02934.x

[106] Janarthanan S, Ditah I, Adler DG. Clostridium difficile-associated diarrhea and proton pump inhibitor therapy: a meta-analysis. Am J Gastroenterol. 2012;107:1001–10. 22710578 10.1038/ajg.2012.179

[107] Loo VG, Bourgault A-M, Poirier L. Host and pathogen factors for Clostridium difficile infection and colonization. N Engl J Med. 2011;365:1693–703. 22047560 10.1056/NEJMoa1012413

[108] Pappas PG, Kauffman CA, Andes DR. Clinical practice guideline for the management of Candidiasis: 2016 update by the Infectious diseases society of America. Clin Infect. 2016;62:e1-50. 10.1093/cid/civ933 PMC4725385 26679628

[109] Uyeki TM, Bernstein HH, Bradley JS. Clinical practice guidelines by the infectious diseases society of America: 2018 update on diagnosis, treatment, chemoprophylaxis, and institutional outbreak management of seasonal influenza. Clin Infect Dis. 2019;68:e1-47. 10.1093/cid/ciy866 PMC6653685 30566567

[110] Beaman MH. Community-acquired acute meningitis and encephalitis: a narrative review. Med J Aust. 2018;209:449–54. 30309300 10.5694/mja17.01073

[111] Schiffer JT, Core L. Herpes simplex virus. In: Mandell, Douglas, and Bennett’s principles practice of infectious diseases. 9th ed. Amsterdam: Elsevier Inc; 2019. p. 1828–48.

[112] Ljungman P, de la Camara R, Robin C. Guidelines for the management of cytomegalovirus infection in patients with haematological malignancies and after stem cell transplantation from the 2017 European conference on infections in leukaemia (ECIL 7). Lancet Infect Dis. 2019;19:e260–72. 31153807 10.1016/S1473-3099(19)30107-0

[113] Bagshaw SM, George C, Bellomo R. Early acute kidney injury and sepsis. A multicentre evaluation. Crit Care. 2008;12:R47. 18402655 10.1186/cc6863 PMC2447598

[114] Uchino S, Kellum JA, Bellomo R, Doig GS, Morimatsu H, Morgera S, et al. Acute renal failure in critically ill patients. A multinational, multicenter study. JAMA. 2005;294:813–8. 16106006 10.1001/jama.294.7.813

[115] Bagshaw SM, Lapinsky S, Dial S, Arabi Y, Dodek P, Wood G, et al. Acute kidney injury in septic shock: clinical outcomes and impact of duration of hypotension prior to initiation of antimicrobial therapy. Intensive Care Med. 2009;35:871–81. 19066848 10.1007/s00134-008-1367-2

[116] Schortgen F, Asfar P. Update in sepsis and acute kidney injury 2014. Am J Respir Crit Care Med. 2015;191:1226–31. 26029837 10.1164/rccm.201502-0307UP

[117] Hoste EA, Bagshaw SM, Bellomo R, Cely CM, Colman R, Cruz DN, et al. Epidemiology of acute kidney injury in critically ill patients: the multinational AKI-EPI study. Intensive Care Med. 2015;41:1411–23. 26162677 10.1007/s00134-015-3934-7

[118] Roberts JA, Abdul-Aziz MH, Lipman J, Mouton JW, Vinks AA, Felton TW, et al. Individualised antibiotic dosing for patients who are critically ill: challenges and potential solutions. Lancet Infect Dis. 2014;14:498–509. 24768475 10.1016/S1473-3099(14)70036-2 PMC4181663

[119] Godin M, Murray P, Mehta RL. Clinical approach to the patient with AKI and sepsis. Semin Nephrol. 2015;35:12–22. 25795496 10.1016/j.semnephrol.2015.01.003 PMC5617729

[120] Sime FB, Roberts MS, Roberts JA. Optimization of dosing regimens and dosing in special populations. Clin Microbiol Infect. 2015;21:886–93. 25980350 10.1016/j.cmi.2015.05.002

[121] Lewis SJ, Mueller BA. Antibiotic dosing in patients with acute kidney injury. “Enough but not too much.” J Intensive Care Med. 2016;31:164–76. 25326429 10.1177/0885066614555490

[122] Póvoa P, Moniz P, Pereira JG, Coelho L. Optimizing antimicrobial drug dosing in critically ill patients. Microorganisms. 2021;9:1401. 34203510 10.3390/microorganisms9071401 PMC8305961

[123] Eyler RF, Mueller BA. Antibiotic dosing in critically ill patients with acute kidney injury. Nat Rev Nephrol. 2011;7:226–35. 21343897 10.1038/nrneph.2011.12

[124] Bernier-Jean A, Beaubien-Souligny W, Goupil R, Madore F, Paquette F, Troyanov S, et al. Diagnosis and outcomes of acute kidney injury using surrogate and imputation methods for missing preadmission creatinine values. BMC Nephrol. 2017;18:141. 28454562 10.1186/s12882-017-0552-3 PMC5410063

[125] Ostermann M, Joannidis M. Diagnosis and diagnostic workup. Crit Care. 2016;2016(20):299. 10.1186/s13054-016-1478-z PMC5037640 27670788

[126] De Waele JJ, Lipman J, Akova M, Bassetti M, Dimopoulos G, Kaukonen M, et al. Risk factors for target non-attainment during empirical treatment with β-lactam antibiotics in critically ill patients. Intensive Care Med. 2014;40:1340–51. 25053248 10.1007/s00134-014-3403-8

[127] Baptista JP, Udy AA, Sousa E, Pimentel J, Wang L, Roberts JA, et al. A comparison of estimates of glomerular filtration in critically ill patients with augmented renal clearance. Crit Care. 2011;15:R139. 21651804 10.1186/cc10262 PMC3219011

[128] Martin JH, Fay MF, Udy A, Roberts J, Kirkpatrick C, Ungerer J, et al. Pitfalls of using estimations of glomerular filtration rate in an intensive care population. Intern Med J. 2011;41:537–43. 21762334 10.1111/j.1445-5994.2009.02160.x

[129] Gonçalves-Pereira J, Póvoa P. Antibiotics in critically ill patients. A systematic review of the pharmacokinetics of β-lactams. Crit Care. 2011;15:R206. 21914174 10.1186/cc10441 PMC3334750

[130] Grootaert V, Willems L, Debaveye Y, Meyfroidt G, Spriet I. Augmented renal clearance in the critically ill. How to assess kidney function. Ann Pharmacother. 2012;46:952–9. 22693271 10.1345/aph.1Q708

[131] Udy AA, Roberts JA, Lipman J. Clinical implications of antibiotic pharmacokinetic principles in the critically ill. Intensive Care Med. 2013;39:2070–82. 24045886 10.1007/s00134-013-3088-4

[132] Udy AA, Baptista JP, Lim NL, Joynt GM, Jarrett P, Wockner L, et al. Augmented renal clearance in the ICU: results of a multicenter observational study of renal function in critically ill patients with normal plasma creatinine concentrations. Crit Care Med. 2014;42:520–7. 24201175 10.1097/CCM.0000000000000029

[133] Shekar K, Roberts JA, McDonald CI, Ghassabian S, Anstey C, Wallis SC, et al. Protein-bound drugs are prone to sequestration in the extracorporeal membrane oxygenation circuit. Results from an ex vivo study. Crit Care. 2015;19:164. 25888449 10.1186/s13054-015-0891-z PMC4407324

[134] Dzierba AL, Abrams D, Brodie D. Medicating patients during extracorporeal membrane oxygenation. The evidence is building. Crit Care. 2017;21:66. 28320466 10.1186/s13054-017-1644-y PMC5359850

[135] Donadello K, Antonucci E, Cristallini S, Roberts JA, Beumier M, Scolletta S, et al. β-Lactam pharmacokinetics during extracorporeal membrane oxygenation therapy. A case-control study. Int J Antimicrob Agents. 2015;45:278–82. 25542059 10.1016/j.ijantimicag.2014.11.005

[136] Kühn D, Metz C, Seiler F, Wehrfritz H, Roth S, Alqudrah M, et al. Antibiotic therapeutic drug monitoring in intensive care patients treated with different modalities of extracorporeal membrane oxygenation (ECMO) and renal replacement therapy. A prospective, observational single-center study. Crit Care. 2020;24:664. 33239110 10.1186/s13054-020-03397-1 PMC7689974

[137] Choi G, Gomersall CD, Tian Q, Joynt GM, Freebairn R, Lipman J. Principles of antibacterial dosing in continuous renal replacement therapy. Crit Care Med. 2009;37:2268–82. 19487930 10.1097/CCM.0b013e3181aab3d0

[138] Scoville BA, Mueller BA. Medication dosing in critically ill patients with acute kidney injury treated with renal replacement therapy. Am J Kidney Dis. 2013;61:490–500. 23127618 10.1053/j.ajkd.2012.08.042

[139] Ulldemolins M, Vaquer S, Llauradó-Serra M, Pontes C, Calvo G, Soy D, et al. Beta-lactam dosing in critically ill patients with septic shock and continuous renal replacement therapy. Crit Care. 2014;18:227. 25042938 10.1186/cc13938 PMC4075152

[140] Veiga RP, Paiva JA. Pharmacokinetics-pharmacodynamics issues relevant for the clinical use of beta-lactam antibiotics in critically ill patients. Crit Care. 2018;22:233. 30244674 10.1186/s13054-018-2155-1 PMC6151903

[141] Roberts DM, Roberts JA, Roberts MS, Liu X, Nair P, Cole L, et al. Variability of antibiotic concentrations in critically ill patients receiving continuous renal replacement therapy. A multicentre pharmacokinetic study. Crit Care Med. 2012;40:1523–8. 22511133 10.1097/CCM.0b013e318241e553

[142] Roberts DM, Liu X, Roberts JA, Nair P, Cole L, Roberts MS, et al. A multicenter study on the effect of continuous hemodiafiltration intensity on antibiotic pharmacokinetics. Crit Care. 2015;19:84. 25881576 10.1186/s13054-015-0818-8 PMC4404619

[143] Seyler L, Cotton F, Taccone FS, De Backer D, Macours P, Vincent JL, et al. Recommended β-lactam regimens are inadequate in septic patients treated with continuous renal replacement therapy. Crit Care. 2011;15:R137. 21649882 10.1186/cc10257 PMC3219006

[144] Roberts JA, Joynt GM, Lee A, Choi G, Bellomo R, Kanji S, et al. The effect of renal replacement therapy and antibiotic dose on antibiotic concentrations in critically ill patients. Data from the multinational sampling antibiotics in renal replacement therapy study. Clin Infect Dis. 2021;72:1369–78. 32150603 10.1093/cid/ciaa224

[145] Werumeus Buning A, Hodiamont CJ, Lechner NM, Schokkin M, Elbers PWG, Juffermans NP, et al. Population pharmacokinetics and probability of target attainment of different dosing regimens of Ceftazidime in critically ill patients with a proven or suspected *Pseudomonas aeruginosa* infection. Antibiotics. 2021;10:612. 34063815 10.3390/antibiotics10060612 PMC8224000

[146] Wahby KA, Cunmuljaj L, Mouabbi K, Almadrahi Z, Wilpula L. Evaluation of dosing strategies and trough concentrations of vancomycin in patients undergoing continuous venovenous hemofiltration. Pharmacotherapy. 2021;41:554–61. 33963536 10.1002/phar.2535

[147] Wang C, Zhang C, Li X, Zhao S, He N, Zhai S, et al. Dose optimization of vancomycin for critically ill patients undergoing CVVH. A prospective population PK/PD analysis. Antibiotics. 2021;10:1392. 34827330 10.3390/antibiotics10111392 PMC8614878

[148] Economou CJP, Wong G, McWhinney B, Ungerer JPJ, Lipman J, Roberts JA. Impact of β-lactam antibiotic therapeutic drug monitoring on dose adjustments in critically ill patients undergoing continuous renal replacement therapy. Int J Antimicrob Agents. 2017;49:589–94. 28341612 10.1016/j.ijantimicag.2017.01.009

[149] Abdul-Aziz MH, Sulaiman H, Mat-Nor MB. Beta-Lactam Infusion in Severe Sepsis (BLISS): a prospective, two-centre, open-labelled randomised controlled trial of continuous versus intermittent beta-lactam infusion in critically ill patients with severe sepsis. Intensive Care Med. 2016;42:1535–45. 26754759 10.1007/s00134-015-4188-0

[150] Angus BJ, Smith MD, Suputtamongkol Y. Pharmacokineticpharmacodynamic evaluation of ceftazidime continuous infusion vs intermittent bolus injection in septicaemic melioidosis. Br J Clin Pharmacol. 2000;49:445–52. 10792202 10.1046/j.1365-2125.2000.00179.x PMC2014958

[151] Chytra I, Stepan M, Benes J. Clinical and microbiological efficacy of continuous versus intermittent application of meropenem in critically ill patients: a randomized open-label controlled trial. Crit Care. 2012;16:R113. 22742765 10.1186/cc11405 PMC3580671

[152] Dulhunty JM, Roberts JA, Davis JS. Continuous infusion of beta-lactam antibiotics in severe sepsis: a multicenter doubleblind, randomized controlled trial. Clin Infect Dis. 2013;5(6):236–44. 10.1093/cid/cis856 23074313

[153] Georges B, Conil JM, Cougot P. Cefepime in critically ill patients: continuous infusion vs. an intermittent dosing regimen. Int J Clin Pharmacol Ther. 2005;43:360–9. 16119511 10.5414/cpp43360

[154] Laterre PF, Wittebole X, Van de Velde S. Temocillin (6 g daily) in critically ill patients: continuous infusion versus three times daily administration. J Antimicrob Chemother. 2015;70:891–8. 25433006 10.1093/jac/dku465

[155] Lau WK, Mercer D, Itani KM. Randomized, open-label, comparative study of piperacillin-tazobactam administered by continuous infusion versus intermittent infusion for treatment of hospitalized patients with complicated intra-abdominal infection. Antimicrob Agents Chemother. 2006;50:3556–61. 16940077 10.1128/AAC.00329-06 PMC1635208

[156] Lipš M, Siller M, Strojil J. Pharmacokinetics of imipenem in critically ill patients during empirical treatment of nosocomial pneumonia: a comparison of 0.5-h and 3-h infusions. Int J Antimicrob Agents. 2014;44:358–62. 25216543 10.1016/j.ijantimicag.2014.05.011

[157] Dulhunty JM, Roberts JA, Davis JS. A Multicenter randomized trial of continuous versus intermittent β-lactam infusion in severe sepsis. Am J Respir Crit Care Med. 2015;192:1298–305. 26200166 10.1164/rccm.201505-0857OC

[158] Rafati MR, Rouini MR, Mojtahedzadeh M. Clinical efficacy of continuous infusion of piperacillin compared with intermittent dosing in septic critically ill patients. Int J Antimicrob Agents. 2006;28:122–7. 16815689 10.1016/j.ijantimicag.2006.02.020

[159] Roberts JA, Boots R, Rickard CM. Is continuous infusion ceftriaxone better than once-a-day dosing in intensive care? A randomized controlled pilot study. J Antimicrob Chemother. 2007;59:285–91. 17135183 10.1093/jac/dkl478

[160] Roberts JA, Kirkpatrick CM, Roberts MS. Meropenem dosing in critically ill patients with sepsis and without renal dysfunction: intermittent bolus versus continuous administration? Monte Carlo dosing simulations and subcutaneous tissue distribution. J Antimicrob Chemother. 2009;64:142–50. 19398460 10.1093/jac/dkp139

[161] Zhao HY, Gu J, Lyu J. Pharmacokinetic and pharmacodynamic efficacies of continuous versus intermittent administration of meropenem in patients with severe sepsis and septic shock: a prospective randomized pilot study. Chin Med J. 2017;130:1139–45. 28485312 10.4103/0366-6999.205859 PMC5443018

[162] Nicolau DP, McNabb J, Lacy MK. Continuous versus intermittent administration of ceftazidime in intensive care unit patients with nosocomial pneumonia. Int J Antimicrob Agents. 2001;17:497–504. 11397621 10.1016/s0924-8579(01)00329-6

[163] Mahtabalsadat M, Farid Z, Iman K. The clinical and paraclinical effectiveness of four-hour infusion vs. half-hour infusion of high-dose ampicillin-sulbactam in treatment of critically ill patients with sepsis or septic shock: an assessor-blinded randomized clinical trial. J Crit Care. 2023;73:154170. 36272277 10.1016/j.jcrc.2022.154170

[164] Christina MN, Elmazar MM, Nagwa AS. Extended infusion of piperacillin–tazobactam versus intermittent infusion in critically ill Egyptian patients: a cost-effectiveness study. Sci Rep. 2020;12:10882. 10.1038/s41598-022-12861-7 PMC9237083 35760971

[165] Monti G, Bradić N, Marzaroli M. Continuous vs intermittent meropenem administration in critically ill patients with sepsis: the MERCY randomized clinical trial. JAMA. 2023;330:141–51. 37326473 10.1001/jama.2023.10598 PMC10276329

[166] Wysocki M, Delatour F, Faurisson F. Continuous versus intermittent infusion of vancomycin in severe staphylococcal infections: prospective multicenter randomized study. Antimicrob Agents Chemother. 2001;45:2460–7. 11502515 10.1128/AAC.45.9.2460-2467.2001 PMC90678

[167] Eldemiry EM, Sabry NA, Abbassi MM. A specially tailored vancomycin continuous infusion regimen for renally impaired critically ill patients. SAGE Open Med. 2013;1:7921. 10.1177/2050312113507921 PMC4687768 26770686

[168] Schmelzer TM, Christmas AB, Norton HJ. Vancomycin intermittent dosing versus continuous infusion for treatment of ventilator-associated pneumonia in trauma patients. Am Surg. 2013;79:1185–90. 24165255 10.1177/000313481307901123

[169] Roberts JA, Paul SK, Akova M, Bassetti M, De Waele JJ, Dimopoulos G, et al. DALI: defining antibiotic levels in intensive care unit patients: are current β-lactam antibiotic doses sufficient for critically ill patients? Clin Infect Dis. 2014;58:1072–83. 24429437 10.1093/cid/ciu027

[170] Kollef MH, Sherman G, Ward S, Fraser VJ. Inadequate antimicrobial treatment of infections: a risk factor for hospital mortality among critically ill patients. Chest. 1999;115:462–74. 10027448 10.1378/chest.115.2.462

[171] Ibrahim EH, Sherman G, Ward S, Fraser VJ, Kollef MH. The influence of inadequate antimicrobial treatment of bloodstream infections on patient outcomes in the ICU setting. Chest. 2000;118:146–55. 10893372 10.1378/chest.118.1.146

[172] Shorr AF, Micek ST, Welch EC, Doherty JA, Reichley RM, Kollef MH. Inappropriate antibiotic therapy in gram-negative sepsis increases hospital length of stay. Crit Care Med. 2011;39:46–51. 20890186 10.1097/CCM.0b013e3181fa41a7

[173] Bartal C, Danon A, Schlaeffer F, Reisenberg K, Alkan M, Smoliakov R, et al. Pharmacokinetic dosing of aminoglycosides: a controlled trial. Am J Med. 2003;114:194–8. 12637133 10.1016/s0002-9343(02)01476-6

[174] De Waele JJ, Carrette S, Carlier M, Stove V, Boelens J, Claeys G, et al. Therapeutic drug monitoring-based dose optimisation of piperacillin and meropenem: a randomised controlled trial. Intensive Care Med. 2014;40:380–7. 24356862 10.1007/s00134-013-3187-2

[175] Ewoldt TMJ, Abdulla A, Rietdijk WJR, Muller AE, de Winter BCM, Hunfeld NGM, et al. Model-informed precision dosing of beta-lactam antibiotics and ciprofloxacin in critically ill patients: a multicentre randomised clinical trial. Intensive Care Med. 2022;48:1760–71. 36350354 10.1007/s00134-022-06921-9 PMC9645317

[176] Hagel S, Bach F, Brenner T, Bracht H, Brinkmann A, Annecke T, TARGET Trial Investigators, et al. Effect of therapeutic drug monitoring-based dose optimization of piperacillin/tazobactam on sepsis-related organ dysfunction in patients with sepsis: a randomized controlled trial. Intensive Care Med. 2022;48:311–21. 35106617 10.1007/s00134-021-06609-6 PMC8866359

[177] Roggeveen LF, Guo T, Fleuren LM, Driessen R, Thoral P, van Hest RM, et al. Right dose, right now: bedside, real-time, data-driven, and personalised antibiotic dosing in critically ill patients with sepsis or septic shock-a two-centre randomised clinical trial. Crit Care. 2022;26:265. 36064438 10.1186/s13054-022-04098-7 PMC9443636

[178] Takahashi N, Kondo Y, Kubo K, Egi M, Kano KI, Ohshima Y, et al. Efficacy of therapeutic drug monitoring-based antibiotic regimen in critically ill patients: a systematic review and meta-analysis of randomized controlled trials. J Intensive Care. 2023;11:48. 37936203 10.1186/s40560-023-00699-8 PMC10631080

[179] Leone M, Bechis C, Baumstarck K. De-escalation versus continuation of empirical antimicrobial treatment in severe sepsis: a multicenter non-blinded randomized noninferiority trial. Intensive Care Med. 2014;40:1399–408. 25091790 10.1007/s00134-014-3411-8

[180] Byoung Soo K, Sang Ho C, Younsuck K, Jin-Won H, Sang-Bum H, Chae-Man L. Safety of antimicrobial de-escalation for culture-negative severe pneumonia. J Crit Care. 2019;54:14–9. 31319347 10.1016/j.jcrc.2019.06.026 PMC7126337

[181] Pedroso JVM, Motter FR, Koba ST. Feasibility of de-escalation implementation for positive blood cultures in patients with sepsis: a prospective cohort study. Front Pharmacol. 2021;11:576849. 33643032 10.3389/fphar.2020.576849 PMC7907639

[182] Routsi C, Gkoufa A, Arvaniti K. De-escalation of antimicrobial therapy in ICU settings with high prevalence of multidrug-resistant bacteria: a multicentre prospective observational cohort study in patients with sepsis or septic shock. J Antimicrob Chemother. 2020;75:3665–74. 32865203 10.1093/jac/dkaa375

[183] Palacios-Baena ZR, Delgado-Valverde M, Valiente Méndez A. Impact of de-escalation on prognosis of patients with bacteremia due to Enterobacteriaceae: a post hoc analysis from a Multicenter prospective cohort. Clin Infect Dis. 2019;69:956–62. 30535051 10.1093/cid/ciy1032

[184] Carugati M, Franzetti F, Wiemken T. De-escalation therapy among bacteraemic patients with community-acquired pneumonia. Clin Microbiol Infect. 2015;21: e87. 10.1016/j.cmi.2015.06.015 26115864

[185] Garnacho-Montero J, Gutiérrez-Pizarraya A. Escoresca-Ortega A De-escalation of empirical therapy is associated with lower mortality in patients with severe sepsis and septic shock. Intensive Care Med. 2014;40:32–40. 24026297 10.1007/s00134-013-3077-7

[186] Gonzalez L, Cravoisy A, Barraud D. Factors influencing the implementation of antibiotic de-escalation and impact of this strategy in critically ill patients. Crit Care. 2013;17:R140. 23849321 10.1186/cc12819 PMC4055984

[187] Heenen S, Jacobs F, Vincent JL. Antibiotic strategies in severe nosocomial sepsis: why do we not de-escalate more often? Crit Care Med. 2012;40:1404–9. 22430235 10.1097/CCM.0b013e3182416ecf

[188] Lee CC, Lee NY, Chen PL. Impact of antimicrobial strategies on clinical outcomes of adults with septic shock and community-onset Enterobacteriaceae bacteremia: de-escalation is beneficial. Diagn Microbiol Infect Dis. 2015;82:158–64. 25796557 10.1016/j.diagmicrobio.2015.03.004

[189] Lee CC, Wang JL, Lee CH. Clinical benefits of antimicrobial de-escalation in adults with community-onset monomicrobial *Escherichia coli*, *Klebsiella* species and *Proteus mirabilis* bacteremia. Int J Antimicrob Agents. 2017;50:371–6. 28694235 10.1016/j.ijantimicag.2017.03.024

[190] Mokart D, Slehofer G, Lambert J. De-escalation of antimicrobial treatment in neutropenic patients with severe sepsis: results from an observational study. Intensive Care Med. 2014;40:41–9. 24231857 10.1007/s00134-013-3148-9

[191] Moraes RB, Guillén JA, Zabaleta WJ, Borges FK. De-escalation, adequacy of antibiotic therapy and culture positivity in septic patients: an observational study. Descalonamento, adequação antimicrobiana e positividade de culturas em pacientes sépticos: estudo observacional. Rev Bras Ter Intensiva. 2016;28:315–22. 27626951 10.5935/0103-507X.20160044 PMC5051191

[192] Morel J, Casoetto J, Jospé R. De-escalation as part of a global strategy of empiric antibiotherapy management. A retrospective study in a medico-surgical intensive care unit. Crit Care. 2010;14:R225. 21167047 10.1186/cc9373 PMC3219998

[193] Niimura T, Zamami Y, Imai T. Evaluation of the benefits of de-escalation for patients with sepsis in the emergency intensive care unit. J Pharm Pharm Sci. 2018;21:54–9. 29455711 10.18433/jpps29737

[194] Oshima T, Kodama Y, Takahashi W. Empiric antibiotic therapy for severe sepsis and septic shock. Surg Infect. 2016;17:210–6. 10.1089/sur.2014.096 26630548

[195] Salahuddin N, Amer L, Joseph M, El Hazmi A, Hawa H, Maghrabi K. Determinants of deescalation failure in critically ill patients with sepsis: a prospective cohort study. Crit Care Res Prac. 2016;2016:6794861. 10.1155/2016/6794861 PMC4963586 27493799

[196] Viasus D, Simonetti AF, Garcia-Vidal C, Niubó J, Dorca J, Carratalà J. Impact of antibiotic de-escalation on clinical outcomes in community-acquired pneumococcal pneumonia. J Antimicrob Chemother. 2017;72:547–53. 27798219 10.1093/jac/dkw441

[197] Campion M, Scully G. Antibiotic use in the intensive care unit: optimization and de-escalation. J Intensive Care Med. 2018;33:647–55. 29534630 10.1177/0885066618762747

[198] Wisplinghoff H, Bischoff T, Tallent SM. Nosocomial bloodstream infections in US hospitals: analysis of 24,179 cases from a prospective nationwide surveillance study. Clin Infect Dis. 2004;39:309–17. 15306996 10.1086/421946

[199] Nagao M. A multicentre analysis of epidemiology of the nosocomial bloodstream infections in Japanese university hospitals. Clin Microbiol Infect. 2013;19:852–8. 23176224 10.1111/1469-0691.12083

[200] Rouzé A, Loridant S, Poissy J. Biomarker-based strategy for early discontinuation of empirical antifungal treatment in critically ill patients: a randomized controlled trial. Intensive Care Med. 2017;43:1668–77. 28936678 10.1007/s00134-017-4932-8

[201] De Pascale G, Posteraro B, D’Arrigo S. (1,3)-β-D-Glucan-based empirical antifungal interruption in suspected invasive candidiasis: a randomized trial. Crit Care. 2020;24:550. 32891170 10.1186/s13054-020-03265-y PMC7487510

[202] Micek S, Johnson MT, Reichley R, Kollef MH. An institutional perspective on the impact of recent antibiotic exposure on length of stay and hospital costs for patients with gram-negative sepsis. BMC Infect Dis. 2012;12:56. 22414209 10.1186/1471-2334-12-56 PMC3325861

[203] Baggs J, Jernigan JA, Halpin AL, Epstein L, Hatfield KM, McDonald LC. Risk of subsequent sepsis within 90 days after a hospital stay by type of antibiotic exposure. Clin Infect Dis. 2018;66:1004–12. 29136126 10.1093/cid/cix947 PMC7909479

[204] Charles PE, Tinel C, Barbar S. Procalcitonin kinetics within the first days of sepsis: relationship with the appropriateness of antibiotic therapy and the outcome. Crit Care. 2009;13:R38. 19291325 10.1186/cc7751 PMC2689475

[205] Karlsson S, Heikkinen M, Pettilä V. Predictive value of procalcitonin decrease in patients with severe sepsis: a prospective observational study. Crit Care. 2010;14:R205. 21078153 10.1186/cc9327 PMC3219988

[206] Póvoa P, Coelho L, Dal-Pizzol F. How to use biomarkers of infection or sepsis at the bedside: guide to clinicians. Intensive Care Med. 2023;49:142–53. 36592205 10.1007/s00134-022-06956-y PMC9807102

[207] Hochreiter M, Köhler T, Schweiger AM. Procalcitonin to guide duration of antibiotic therapy in intensive care patients: a randomized prospective controlled trial. Crit Care. 2009;13:R83. 19493352 10.1186/cc7903 PMC2717450

[208] Ali WA, Bazan NS, Elberry AA, Hussein RRS. A randomized trial to compare procalcitonin and C-reactive protein in assessing severity of sepsis and in guiding antibacterial therapy in Egyptian critically ill patients. Ir J Med Sci. 2021;190:1487–95. 33447966 10.1007/s11845-020-02494-y

[209] Deliberato RO, Marra AR, Sanches PR. Clinical and economic impact of procalcitonin to shorten antimicrobial therapy in septic patients with proven bacterial infection in an intensive care setting. Diagn Microbiol Infect Dis. 2013;76:266–71. 23711530 10.1016/j.diagmicrobio.2013.03.027

[210] Borges I, Borges I, Carneiro R. Duration of antibiotic therapy in critically ill patients: a randomized controlled trial of a clinical and C-reactive protein-based protocol versus an evidence-based best practice strategy without biomarkers. Crit Care. 2020;24:281. 32487263 10.1186/s13054-020-02946-y PMC7266125

[211] Bloos F, Trips E, Nierhaus A. Effect of sodium selenite administration and procalcitonin-guided therapy on mortality in patients with severe sepsis or septic shock: a randomized clinical trial. JAMA Intern Med. 2016;176:1266–76. 27428731 10.1001/jamainternmed.2016.2514

[212] de Jong E, van Oers JA, Beishuizen A. Efficacy and safety of procalcitonin guidance in reducing the duration of antibiotic treatment in critically ill patients: a randomised, controlled, open-label trial. Lancet Infect Dis. 2016;16:819–27. 26947523 10.1016/S1473-3099(16)00053-0

[213] Liu Y, Yang W, Wei J. Guiding effect of serum procalcitonin (PCT) on the antibiotic application to patients with sepsis. Iran J Public Health. 2017;46:1535–9. 29167772 PMC5696693

[214] Schroeder S, Hochreiter M, Koehler T. Procalcitonin (PCT)-guided algorithm reduces length of antibiotic treatment in surgical intensive care patients with severe sepsis: results of a prospective randomized study. Langenbecks Arch Surg. 2009;394:221–6. 19034493 10.1007/s00423-008-0432-1

[215] Shehabi Y, Sterba M, Garrett PM. Procalcitonin algorithm in critically ill adults with undifferentiated infection or suspected sepsis: a randomized controlled trial. Am J Respir Crit Care Med. 2014;190:1102–10. 25295709 10.1164/rccm.201408-1483OC

[216] Annane D, Maxime V, Faller JP. Procalcitonin levels to guide antibiotic therapy in adults with non-microbiologically proven apparent severe sepsis: a randomised controlled trial. BMJ Open. 2013;3: e002186. 10.1136/bmjopen-2012-002186 PMC3586059 23418298

[217] Kyriazopoulou E, Liaskou-Antoniou L, Adamis G. Procalcitonin to reduce long-term infection-associated adverse events in sepsis a randomized trial. Am J Respir Crit Care Med. 2021;203:202–10. 32757963 10.1164/rccm.202004-1201OC PMC7874409

[218] Oliveira CF, Botoni FA, Oliveira CRA. Procalcitonin versus C-reactive protein for guiding antibiotic therapy in sepsis: a randomized trial. Crit Care Med. 2013;41:2336–43. 23921272 10.1097/CCM.0b013e31828e969f

[219] Jeon K, Suh JK, Jang EJ. Procalcitonin-guided treatment on duration of antibiotic therapy and cost in septic patients (PRODA): a multi-center randomized controlled trial. J Korean Med Sci. 2019;34: e10. 30977312 10.3346/jkms.2019.34.e110 PMC6460106

[220] Vishalashi SG, Gupta P, Verma PK. Serum procalcitonin as a biomarker to determine the duration of antibiotic therapy in adult patients with sepsis and septic shock in intensive care units: a prospective study. Ind J Crit Care Med. 2021;25:507–11. 10.5005/jp-journals-10071-23802 PMC8196370 34177168

[221] Bouadma L, Luyt CE, Tubach F. Use of procalcitonin to reduce patients’ exposure to antibiotics in intensive care units (PRORATA trial): a multicentre randomised controlled trial. Lancet. 2010;375:463–74. 20097417 10.1016/S0140-6736(09)61879-1

[222] Nobre V, Harbarth S, Graf JD, Rohner P, Pugin J. Use of procalcitonin to shorten antibiotic treatment duration in septic patients: a randomized trial. Am J Respir Crit Care Med. 2008;177:498–505. 18096708 10.1164/rccm.200708-1238OC

[223] Lee RA, Stripling JT, Spellberg B, Centor RM. Short-course antibiotics for common infections: what do we know and where do we go from here? Clin Microbiol Infect. 2023;29:150–9. 36075498 10.1016/j.cmi.2022.08.024

[224] Israelsen SB, Fally M, Tarp B, Kolte L, Ravn P, Benfield T. Short-course antibiotic therapy for hospitalized patients with early clinical response in community-acquired pneumonia: a multicentre cohort study. Clin Microbiol Infect. 2023;29:54–60. 35988851 10.1016/j.cmi.2022.08.004

[225] Molina J, Montero-Mateos E, Praena-Segovia J, León-Jiménez E, Natera C, López-Cortés LE, et al. Seven-versus 14-day course of antibiotics for the treatment of bloodstream infections by Enterobacterales: a randomized controlled trial. Clin Microbiol Infect. 2022;28:550–7. 34508886 10.1016/j.cmi.2021.09.001

[226] Yahav D, Franceschini E, Koppel F, Turjeman A, Babich T, Bitterman R, et al. Seven versus 14 days of antibiotic therapy for uncomplicated gram-negative Bacteremia: a noninferiority randomized controlled trial. Clin Infect Dis. 2019;69:1091–8. 30535100 10.1093/cid/ciy1054

[227] Montravers P, Tubach F, Lescot T, Veber B, Esposito-Farèse M, Seguin P, et al. Short-course antibiotic therapy for critically ill patients treated for postoperative intra-abdominal infection: the DURAPOP randomised clinical trial. Intensive Care Med. 2018;44:300–10. 29484469 10.1007/s00134-018-5088-x

[228] Kollef MH, Chastre J, Clavel M, Restrepo MI, Michiels B, Kaniga K, et al. A randomized trial of 7-day doripenem versus 10-day imipenem-cilastatin for ventilator-associated pneumonia. Crit Care. 2012;16:R218. 23148736 10.1186/cc11862 PMC3672596

[229] Capellier G, Mockly H, Charpentier C, Annane D, Blasco G, Desmettre T, et al. Early-onset ventilator-associated pneumonia in adults randomized clinical trial: comparison of 8 versus 15 days of antibiotic treatment. PLoS ONE. 2012;7: e41290. 22952580 10.1371/journal.pone.0041290 PMC3432026

[230] Chastre J, Wolff M, Fagon JY, Chevret S, Thomas F, Wermert D, et al. Comparison of 8 vs 15 days of antibiotic therapy for ventilator-associated pneumonia in adults: a randomized trial. JAMA. 2003;290:2588–98. 14625336 10.1001/jama.290.19.2588

[231] Hernández G, Ospina-Tascón GA, Damiani LP, Estenssoro E, Dubin A, Hurtado J, et al. Effect of a resuscitation strategy targeting peripheral perfusion status vs serum lactate levels on 28-day mortality among patients with septic shock: the ANDROMEDA-SHOCK randomized clinical trial. JAMA. 2019;321:654–64. 30772908 10.1001/jama.2019.0071 PMC6439620

[232] Rowan KM, Angus DC, Bailey M, Barnato AE, Bellomo R, Canter RR, et al. Early, goal-directed therapy for septic shock—a patient-level meta-analysis. N Engl J Med. 2017;376:2223–34. 28320242 10.1056/NEJMoa1701380

[233] Ltaief Z, Schneider AG, Liaudet L. Pathophysiology and clinical implications of the veno-arterial PCO(2) gap. Crit Care. 2021;25:318. 34461974 10.1186/s13054-021-03671-w PMC8407023

[234] Yumoto T, Kuribara T, Yamada K, Sato T, Koba S, Tetsuhara K, et al. Clinical parameter-guided initial resuscitation in adult patients with septic shock: a systematic review and network meta-analysis. Acute Med Surg. 2023;10: e914. 38148753 10.1002/ams2.914 PMC10750303

[235] Musikatavorn K, Plitawanon P, Lumlertgul S, Narajeenron K, Rojanasarntikul D, Tarapan T, et al. Randomized controlled trial of ultrasound-guided fluid resuscitation of sepsis-induced hypoperfusion and septic shock. West J Emerg Med. 2021;22:369–78. 33856325 10.5811/westjem.2020.11.48571 PMC7972359

[236] Lanspa MJ, Burk RE, Wilson EL, Hirshberg EL, Grissom CK, Brown SM. Echocardiogram-guided resuscitation versus early goal-directed therapy in the treatment of septic shock: a randomized, controlled, feasibility trial. J Intensive Care. 2018;6:50. 30123511 10.1186/s40560-018-0319-3 PMC6090604

[237] Asfar P, Meziani F, Hamel JF, Grelon F, Megarbane B, Anguel N, et al. High versus low blood-pressure target in patients with septic shock. N Engl J Med. 2014;370:1583–93. 24635770 10.1056/NEJMoa1312173

[238] Lamontagne F, Meade MO, Hebert PC, Asfar P, Lauzier F, Seely AJE, et al. Higher versus lower blood pressure targets for vasopressor therapy in shock: a multicentre pilot randomized controlled trial. Intensive Care Med. 2016;42:542–50. 26891677 10.1007/s00134-016-4237-3

[239] Lamontagne F, Richards-Belle A, Thomas K, Harrison DA, Sadique MZ, Grieve RD, et al. Effect of reduced exposure to vasopressors on 90-day mortality in older critically ill patients with vasodilatory hypotension: a randomized clinical trial. JAMA. 2020;323:938–49. 32049269 10.1001/jama.2020.0930 PMC7064880

[240] Yunos NM, Bellomo R, Hegarty C, Story D, Ho L, Bailey M. Association between a chloride-liberal vs chloride-restrictive intravenous fluid administration strategy and kidney injury in critically ill adults. JAMA. 2012;308:1566–72. 23073953 10.1001/jama.2012.13356

[241] Young P, Bailey M, Beasley R, Henderson S, Mackle D, McArthur C, et al. Effect of a buffered crystalloid solution vs saline on acute kidney injury among patients in the intensive care unit: the SPLIT randomized clinical trial. JAMA. 2015;314:1701–10. 26444692 10.1001/jama.2015.12334

[242] Verma B, Luethi N, Cioccari L, Lloyd-Donald P, Crisman M, Eastwood G, et al. A multicentre randomised controlled pilot study of fluid resuscitation with saline or plasma-Lyte 148 in critically ill patients. Crit Care Resusc. 2016;18:205–12. 27604335

[243] Semler MW, Wanderer JP, Ehrenfeld JM, Stollings JL, Self WH, Siew ED, et al. Balanced crystalloids versus saline in the intensive care unit. The SALT randomized trial. Am J Respir Crit Care Med. 2017;195:1362–72. 27749094 10.1164/rccm.201607-1345OC PMC5443900

[244] Semler MW, Self WH, Wanderer JP, Ehrenfeld JM, Wang L, Byrne DW, et al. Balanced crystalloids versus saline in critically ill adults. N Engl J Med. 2018;378:829–39. 29485925 10.1056/NEJMoa1711584 PMC5846085

[245] Brown RM, Wang L, Coston TD, Krishnan NI, Casey JD, Wanderer JP, et al. Balanced crystalloids versus saline in sepsis. A secondary analysis of the SMART clinical trial. Am J Respir Crit Care Med. 2019;200:1487–95. 31454263 10.1164/rccm.201903-0557OC PMC6909845

[246] Zampieri FG, Machado FR, Biondi RS, Freitas FGR, Veiga VC, Figueiredo RC, et al. Effect of intravenous fluid treatment with a balanced solution vs 09% saline solution on mortality in critically ill patients. JAMA. 2021;326:1–12. 10.1001/jama.2021.11684 PMC8356144 34375394

[247] Finfer S, Micallef S, Hammond N, Navarra L, Bellomo R, Billot L, et al. Balanced multielectrolyte solution versus saline in critically ill adults. N Engl J Med. 2022;386:815–26. 35041780 10.1056/NEJMoa2114464

[248] Golla R, Kumar S, Dhibhar DP, Bhalla A, Sharma N. 0.9% saline V/S Ringer’s lactate for fluid resuscitation in adult sepsis patients in emergency medical services: an open-label randomized controlled trial. Hong Kong J Emerg Med. 2022;29:271–80.

[249] Rackow EC, Falk JL, Siegel FIA, JS, Packman MI, Haupt MT, et al. Fluid resuscitation in circulatory shock: a comparison of the cardiorespiratory effects of albumin, hetastarch, and saline solutions in patients with hypovolemic and septic shock. Crit Care Med. 1983;11:839–50. 6194934

[250] Van der Heijden M, Verheij J, van Nieuw Amerongen GP, Johan Groeneveld AB. Crystalloid or colloid fluid loading and pulmonary permeability, edema, and injury in septic and nonseptic critically ill patients with hypovolemia. Crit Care Med. 2009;37:1275–81. 19242338 10.1097/CCM.0b013e31819cedfd

[251] SAFE Study Investigators, Finfer S, McEvoy S, Bellomo R, McArthur C, Myburgh J, et al. Impact of albumin compared to saline on organ function and mortality of patients with severe sepsis. Intensive Care Med. 2011;37:86–96. 20924555 10.1007/s00134-010-2039-6

[252] Park CHL, de Almeida JP, de Oliveira GQ, Rizk SI, Fukushima JT, Nakamura RE, et al. Lactated Ringer’s versus 4% albumin on lactated Ringer’s in early sepsis therapy in cancer patients: a pilot single-center randomized trial. Crit Care Med. 2019;47:e798-805. 31356475 10.1097/CCM.0000000000003900

[253] Perner A, Haase N, Guttormsen AB, et al. Hydroxyethyl starch 130/0.42 versus Ringer’s acetate in severe sepsis. N Engl J Med. 2012;367:124–34. 22738085 10.1056/NEJMoa1204242

[254] Annane D, Siami S, Jaber S. Effects of fluid resuscitation with colloids vs crystalloids on mortality in critically ill patients presenting with hypovolemic shock: the CRISTAL randomized trial. JAMA. 2013;310:1809–17. 24108515 10.1001/jama.2013.280502

[255] Guidet B, Martinet O, Boulain T. Assessment of hemodynamic efficacy and safety of 6% hydroxyethylstarch 130/0.4 vs. 0.9% NaCl fluid replacement in patients with severe sepsis: the CRYSTMAS study. Crit Care. 2012;16:R94. 22624531 10.1186/cc11358 PMC3580640

[256] McIntyre LA, Fergusson D, Cook DJ, et al. Fluid resuscitation in the management of early septic shock (FINESS): a randomized controlled feasibility trial. Can J Anaesth. 2008;55:819–26. 19050085 10.1007/BF03034053

[257] Rowan KM, Angus DC, PRISM investigators. Early, goal-directed therapy for septic shock—a patient-level meta-analysis. N Engl J Med. 2017;376:2223–34. 28320242 10.1056/NEJMoa1701380

[258] Meyhoff TS, Hjortrup PB, Wetterslev J. Restriction of intravenous fluid in ICU patients with septic shock. N Engl J Med. 2022;386:2459–70. 35709019 10.1056/NEJMoa2202707

[259] Shapiro NI, Douglas IS, Brower RG. Early restrictive or liberal fluid management for sepsis-induced hypotension. N Engl J Med. 2023;388:499–510. 36688507 10.1056/NEJMoa2212663 PMC10685906

[260] Boyd JH, Forbes J, Nakada TA. Fluid resuscitation in septic shock: a positive fluid balance and elevated central venous pressure are associated with increased mortality. Crit Care Med. 2011;39:259–65. 20975548 10.1097/CCM.0b013e3181feeb15

[261] Kuttab H, Lykins JD, Hughes MD. Evaluation and predictors of fluid resuscitation in patients with severe sepsis and septic shock. Crit Care Med. 2019;47:1582–90. 31393324 10.1097/CCM.0000000000003960 PMC8096207

[262] Yealy D, Kellum JA, Huang DT. A randomized trial of protocol-based care for early septic shock. N Engl J Med. 2014;370:1683–93. 24635773 10.1056/NEJMoa1401602 PMC4101700

[263] Peake SL, Delaney A, Bailey M. Goal-directed resuscitation for patients with early septic shock. N Engl J Med. 2014;371:1496–506. 25272316 10.1056/NEJMoa1404380

[264] Mouncey PR, Osborn TM, Power GS. Trial of early, goal-directed resuscitation for septic shock. N Engl J Med. 2015;372:1301–11. 25776532 10.1056/NEJMoa1500896

[265] Tigbu BM, Davari M, Kebriaeezadeh A. Fluid volume, fluid balance and patient outcome in severe sepsis and septic shock: a systematic review. J Crit Care. 2018;48:153–9. 30199843 10.1016/j.jcrc.2018.08.018

[266] Elbouhy MA, Soliman M, Gaber A, Taema KM, Abdel-Aziz A. Early use of norepinephrine improves survival in septic shock: earlier than early. Arch Med Res. 2019;50:325–32. 31677537 10.1016/j.arcmed.2019.10.003

[267] Macdonald SPJ, Keijzers G, Taylor DM, Kinnear F, Arendts G, Fatovich DM, et al. Restricted fluid resuscitation in suspected sepsis associated hypotension (REFRESH): a pilot randomised controlled trial. Intensive Care Med. 2018;44:2070–8. 30382308 10.1007/s00134-018-5433-0

[268] Permpikul C, Tongyoo S, Viarasilpa T, Trainarongsakul T, Chakorn T, Udompanturak S. Early use of norepinephrine in septic shock resuscitation (CENSER). A randomized trial. Am J Respir Crit Care Med. 2019;199:1097–105. 30704260 10.1164/rccm.201806-1034OC

[269] Tian DH, Smyth C, Keijzers G, Macdonald SP, Peake S, Udy A, et al. Safety of peripheral administration of vasopressor medications: a systematic review. Emerg Med Australas. 2020;32:220–7. 31698544 10.1111/1742-6723.13406

[270] De Backer D, Biston P, Devriendt J, Madl C, Chochrad D, Aldecoa C, et al. Comparison of dopamine and norepinephrine in the treatment of shock. N Engl J Med. 2010;362:779–89. 20200382 10.1056/NEJMoa0907118

[271] Martin C, Papazian L, Perrin G, Saux P. Gouin F Norepinephrine or dopamine for the treatment of hyperdynamic septic shock? Chest. 1993;103:1826–31. 8404107 10.1378/chest.103.6.1826

[272] Patel GP, Grahe JS, Sperry M, Singla S, Elpern E, Lateef O, et al. Efficacy and safety of dopamine versus norepinephrine in the management of septic shock. Shock. 2010;33:375–80. 19851126 10.1097/SHK.0b013e3181c6ba6f

[273] Sazgar M, Golikhatir I, Pashaee SM, Tirandaz F, Firouzian A, Miniahidashti H. Norepinephrine with dopamine infusion on the end-tidal carbon dioxide (ETco2) pressure in patients with septic shock. Caspian J Intern Med. 2021;12:580–5. 34820066 10.22088/cjim.12.4.580 PMC8590414

[274] Barzegar E, Ahmadi A, Mousavi S. The therapeutic role of vasopressin on improving lactate clearance during and after vasogenic shock: microcirculation, is it the black box? Acta Med Iran. 2016;54:15–23. 26853286

[275] Gordon AC, Mason AJ, Thirunavukkarasu N. Effect of early vasopressin vs norepinephrine on kidney failure in patients with septic shock: the VANISH randomized clinical trial. JAMA. 2016;316:509–18. 27483065 10.1001/jama.2016.10485

[276] Hussien RM. EI-Gendy HA, Elsaidy MI, Comparison between norepinephrine alone versus norepinephrine/vasopressin combination for resuscitation in septic shock Egypt J. Crit Care Med. 2021;8:58–65.

[277] Lauzier F, Lévy B, Lamarre P. Vasopressin or norepinephrine in early hyperdynamic septic shock: a randomized clinical trial. Intensive Care Med. 2006;32:1782–9. 17019548 10.1007/s00134-006-0378-0

[278] Russell JA, Walley KR, Singer J. Vasopressin versus norepinephrine infusion in patients with septic shock. N Engl J Med. 2008;358:877–87. 18305265 10.1056/NEJMoa067373

[279] Annane D, Sébille V, Charpentier C, Bollaert PE, François B, Korach JM, et al. Effect of treatment with low doses of hydrocortisone and fludrocortisone on mortality in patients with septic shock. JAMA. 2002;288:862–71. 12186604 10.1001/jama.288.7.862

[280] Annane D, Renault A, Brun-Buisson C, Megarbane B, Quenot JP, Siami S, Cariou A, et al. Hydrocortisone plus fludrocortisone for adults with septic shock. N Engl J Med. 2018;378:809–18. 29490185 10.1056/NEJMoa1705716

[281] Arabi YM, Aljumah A, Dabbagh O, Tamim HM, Rishu AH, Al-Abdulkareem A, et al. Low-dose hydrocortisone in patients with cirrhosis and septic shock: a randomized controlled trial. CMAJ. 2010;182:1971–7. 21059778 10.1503/cmaj.090707 PMC3001503

[282] Bollaert PE, Charpentier C, Levy B, Debouverie M, Audibert G, Larcan A. Reversal of late septic shock with supraphysiologic doses of hydrocortisone. Crit Care Med. 1998;26:645–50. 9559600 10.1097/00003246-199804000-00010

[283] Briegel J, Forst H, Haller M, Schelling G, Kilger E, Kuprat G, et al. Stress doses of hydrocortisone reverse hyperdynamic septic shock: a prospective, randomized, double-blind, single-center study. Crit Care Med. 1999;27:723–32. 10321661 10.1097/00003246-199904000-00025

[284] Gordon AC, Mason AJ, Perkins GD, Stotz M, Terblanche M, Ashby D, et al. The interaction of vasopressin and corticosteroids in septic shock: a pilot randomized controlled trial. Crit Care Med. 2014;42:1325–33. 24557425 10.1097/CCM.0000000000000212

[285] Lv QQ, Gu XH, Chen QH, Yu JQ, Zheng RQ. Early initiation of low-dose hydrocortisone treatment for septic shock in adults: a randomized clinical trial. Am J Emerg Med. 2017;35:1810–4. 28615145 10.1016/j.ajem.2017.06.004

[286] Oppert M, Schindler R, Husung C, Offermann K, Gräf KJ, Boenisch O, et al. Low-dose hydrocortisone improves shock reversal and reduces cytokine levels in early hyperdynamic septic shock. Crit Care Med. 2005;33:2457–64. 16276166 10.1097/01.ccm.0000186370.78639.23

[287] Sprung CL, Annane D, Keh D, Moreno R, Singer M, Freivogel K, et al. Hydrocortisone therapy for patients with septic shock. N Engl J Med. 2008;358:111–24. 18184957 10.1056/NEJMoa071366

[288] Venkatesh B, Finfer S, Cohen J, Rajbhandari D, Arabi Y, Bellomo R, et al. Adjunctive glucocorticoid therapy in patients with septic shock. N Engl J Med. 2018;378:797–808. 29347874 10.1056/NEJMoa1705835

[289] Loisa P, Parviainen I, Tenhunen J, Hovilehto S, Ruokonen E. Effect of mode of hydrocortisone administration on glycemic control in patients with septic shock: a prospective randomized trial. Crit Care. 2007;11:R21. 17306016 10.1186/cc5696 PMC2151907

[290] Tilouche N, Jaoued O, Ali HBS, Gharbi R, Fekih Hassen M, Elatrous S. Comparison between continuous and intermittent administration of hydrocortisone during septic shock: a randomized controlled clinical trial. Shock. 2019;52:481–6. 30628950 10.1097/SHK.0000000000001316

[291] Bergamin FS, Almeida JP, Landoni G, Galas F, Fukushima JT, Fominskiy E, et al. Liberal versus restrictive transfusion strategy in critically ill oncologic patients: the transfusion requirements in critically ill oncologic patients randomized controlled trial. Crit Care Med. 2017;45:766–73. 28240687 10.1097/CCM.0000000000002283

[292] Holst LB, Haase N, Wetterslev J, Wernerman J, Guttormsen AB, Karlsson S, et al. Lower versus higher hemoglobin threshold for transfusion in septic shock. N Engl J Med. 2014;371:1381–91. 25270275 10.1056/NEJMoa1406617

[293] Mazza BF, Freitas FG, Barros MM, Azevedo LC, Machado FR. Blood transfusions in septic shock: is 7.0 g/dL really the appropriate threshold? Rev Bras Ter Intensiva. 2015;27:36–43. 25909311 10.5935/0103-507X.20150007 PMC4396895

[294] Vlaar AP, Oczkowski S, de Bruin S, Wijnberge M, Antonelli M, Aubron C, et al. Transfusion strategies in non-bleeding critically ill adults: a clinical practice guideline from the European society of intensive care medicine. Intensive Care Med. 2020;46:673–96. 31912207 10.1007/s00134-019-05884-8 PMC7223433

[295] Xiao W, Liu W, Zhang J, Liu Y, Hua T, Yang M. The association of diastolic arterial pressure and heart rate with mortality in septic shock: a retrospective cohort study. Eur J Med Res. 2022;27:285. 36496399 10.1186/s40001-022-00930-6 PMC9738025

[296] Sacha GL, Lam SW, Wang L, Duggal A, Reddy AJ, Bauer SR. Association of catecholamine dose, lactate, and shock duration at vasopressin initiation with mortality in patients with septic shock. Crit Care Med. 2022;50:614–23. 34582425 10.1097/CCM.0000000000005317

[297] Cocchi MN, Dargin J, Chase M, Patel PV, Grossestreuer A, Balaji L, et al. Esmolol to treat the hemodynamic effects of septic shock: a randomized controlled trial. Shock. 2022;57:508–17. 35066509 10.1097/SHK.0000000000001905 PMC10448435

[298] Kakihana Y, Nishida O, Taniguchi T, Okajima M, Morimatsu H, Ogura H, et al. Efficacy and safety of landiolol, an ultra-short-acting β1-selective antagonist, for treatment of sepsis-related tachyarrhythmia (J-land 3S): a multicentre, open-label, randomised controlled trial. Lancet Respir Med. 2020;8:863–72. 32243865 10.1016/S2213-2600(20)30037-0

[299] Morelli A, Ertmer C, Westphal M, Rehberg S, Kampmeier T, Ligges S, et al. Effect of heart rate control with esmolol on hemodynamic and clinical outcomes in patients with septic shock: a randomized clinical trial. JAMA. 2013;310:1683–91. 24108526 10.1001/jama.2013.278477

[300] Wang Z, Wu Q, Nie X, Guo J, Yang C. Combination therapy with milrinone and esmolol for heart protection in patients with severe sepsis: a prospective, randomized trial. Clin Drug Investig. 2015;35:707–16. 10.1007/s40261-015-0325-3 26387030

[301] Whitehouse T, Hossain A, Perkins GD, Gordon AC, Bion J, Young D, et al. Landiolol and organ failure in patients with septic shock: the STRESS-L randomized clinical trial. JAMA. 2023;330:1641–52. 37877587 10.1001/jama.2023.20134 PMC10600724

[302] Jaber S, Paugam C, Futier E. Sodium bicarbonate therapy for patients with severe metabolic acidaemia in the intensive care unit (BICAR-ICU): a multicentre, open-label, randomised controlled, phase 3 trial. Lancet. 2018;392:31–40. 29910040 10.1016/S0140-6736(18)31080-8

[303] Bendiab E, Garnier F, Soler M. Long-term outcome of severe metabolic acidemia in ICU patients, a BICAR-ICU trial post hoc analysis. Crit Care Med. 2023;51:e1–12. 36351174 10.1097/CCM.0000000000005706

[304] Mathieu D, Neviere R, Billard V. Effects of bicarbonate therapy on hemodynamics and tissue oxygenation in patients with lactic acidosis: a prospective, controlled clinical study. Crit Care Med. 1991;19:1352–6. 1935152 10.1097/00003246-199111000-00008

[305] Cooper DJ, Walley KR, Wiggs BR. Bicarbonate does not improve hemodynamics in critically ill patients who have lactic acidosis. A prospective, controlled clinical study. Ann Intern Med. 1990;112:492–8. 2156475 10.7326/0003-4819-112-7-492

[306] Parker MM, Shelhamer JH, Bacharach SL, Green MV, Natanson C, Frederick TM, et al. Profound but reversible myocardial depression in patients with septic shock. Ann Intern Med. 1984;100:483–90. 6703540 10.7326/0003-4819-100-4-483

[307] Kakihana Y, Ito T, Nakahara M, Yamaguchi K, Yasuda T. Sepsis-induced myocardial dysfunction: pathophysiology and management. J Intensive Care. 2016;4:22. 27011791 10.1186/s40560-016-0148-1 PMC4804632

[308] Charpentier J, Luyt CE, Fulla Y, Vinsonneau C, Cariou A, Grabar S, et al. Brain natriuretic peptide: a marker of myocardial dysfunction and prognosis during severe sepsis. Crit Care Med. 2004;32:660–5. 15090944 10.1097/01.ccm.0000114827.93410.d8

[309] Landesberg G, Gilon D, Meroz Y, Georgieva M, Levin PD, Goodman S, et al. Diastolic dysfunction and mortality in severe sepsis and septic shock. Eur Heart J. 2012;33:895–903. 21911341 10.1093/eurheartj/ehr351 PMC3345552

[310] Lanspa MJ, Cirulis MM, Wiley BM, Olsen TD, Wilson EL, Beesley SJ, et al. Right ventricular dysfunction in early sepsis and septic shock. Chest. 2021;159:1055–63. 33068615 10.1016/j.chest.2020.09.274 PMC7965651

[311] Dugar S, Sato R, Chawla S, You JY, Wang X, Grimm R, et al. Is left ventricular systolic dysfunction associated with increased mortality among patients with sepsis and septic shock? Chest. 2023;163:1437–47. 36646415 10.1016/j.chest.2023.01.010

[312] Vallabhajosyula S, Shankar A, Vojjini R, Cheungpasitporn W, Sundaragiri PR, DuBrock HM, et al. Impact of right ventricular dysfunction on short-term and Long-term mortality in sepsis: a meta-analysis of 1,373 patients. Chest. 2021;159:2254–63. 33359215 10.1016/j.chest.2020.12.016 PMC8579312

[313] Takahashi Y, Sonoo T, Naraba H, Hashimoto H, Nakamura K. Effect of intra-arterial balloon pumping for refractory septic cardiomyopathy: a case series. Ind J Crit Care Med. 2019;23:182–5. 10.5005/jp-journals-10071-23150 PMC6521831 31130790

[314] Huang CT, Tsai YJ, Tsai PR, Ko WJ. Extracorporeal membrane oxygenation resuscitation in adult patients with refractory septic shock. J Thorac Cardiovasc Surg. 2013;146:1041–6. 22959322 10.1016/j.jtcvs.2012.08.022

[315] Cheng A, Sun HY, Tsai MS, Ko WJ, Tsai PR, Hu FC, et al. Predictors of survival in adults undergoing extracorporeal membrane oxygenation with severe infections. J Thorac Cardiovasc Surg. 2016;152:1526–36. 27692951 10.1016/j.jtcvs.2016.08.038

[316] Bréchot N, Luyt CE, Schmidt M, Leprince P, Trouillet JL, Léger P, et al. Venoarterial extracorporeal membrane oxygenation support for refractory cardiovascular dysfunction during severe bacterial septic shock. Crit Care Med. 2013;41:1616–26. 23563585 10.1097/CCM.0b013e31828a2370

[317] Falk L, Hultman J, Broman LM. Extracorporeal membrane oxygenation for septic shock. Crit Care Med. 2019;47:1097–105. 31162206 10.1097/CCM.0000000000003819

[318] Vogel DJ, Murray J, Czapran AZ, Camporota L, Ioannou N, Meadows CIS, et al. Veno-arterio-venous ECMO for septic cardiomyopathy: a single-centre experience. Perfusion. 2018;33:57–64. 29788842 10.1177/0267659118766833

[319] Bréchot N, Hajage D, Kimmoun A, Demiselle J, Agerstrand C, Montero S, et al. Venoarterial extracorporeal membrane oxygenation to rescue sepsis-induced cardiogenic shock: a retrospective, multicentre, international cohort study. Lancet. 2020;396:545–52. 32828186 10.1016/S0140-6736(20)30733-9

[320] Ling RR, Ramanathan K, Poon WH, Tan CS, Brechot N, Brodie D, et al. Venoarterial extracorporeal membrane oxygenation as mechanical circulatory support in adult septic shock: a systematic review and meta-analysis with individual participant data meta-regression analysis. Crit Care. 2021;25:246. 34261492 10.1186/s13054-021-03668-5 PMC8278703

[321] Haidari Z, Ruhparwar A, Weymann A. Mechanical circulatory support with Impella 5.0 in septic shock. Artif Organs. 2021;45:183–4. 32929738 10.1111/aor.13793

[322] Mustafa A, Obholz J, Hitt N, Rattin R. Prolonged use of an Impella assist device in a sepsis-induced cardiomyopathy: a case report. Cureus. 2021;13: e18889. 34804733 10.7759/cureus.18889 PMC8599395

[323] Marik PE, Linde-Zwirble WT, Bittner EA, Sahatjian J, Hansell D. Fluid administration in severe sepsis and septic shock, patterns and outcomes: an analysis of a large national database. Intensive Care Med. 2017;43:625–32. 28130687 10.1007/s00134-016-4675-y

[324] Chen C, Kollef MH. Targeted fluid minimization following initial resuscitation in septic shock: a pilot study. Chest. 2015;148:1462–9. 26291900 10.1378/chest.15-1525

[325] Jessen MK, Andersen LW, Thomsen MH, Kristensen P, Hayeri W, Hassel RE, et al. Restrictive fluids versus standard care in adults with sepsis in the emergency department (REFACED): a multicenter, randomized feasibility trial. Acad Emerg Med. 2022;29:1172–84. 35652491 10.1111/acem.14546 PMC9804491

[326] Semler MW, Janz DR, Casey JD, Self WH, Rice TW. Conservative fluid management after sepsis resuscitation: a pilot randomized trial. J Intensive Care Med. 2020;35:1374–82. 30630380 10.1177/0885066618823183 PMC6620161

[327] Sivapalan P, Ellekjaer KL, Jessen MK, Meyhoff TS, Cronhjort M, Hjortrup PB, et al. Lower vs higher fluid volumes in adult patients with sepsis: an updated systematic review with meta-analysis and trial sequential analysis. Chest. 2023;164:892–912. 37142091 10.1016/j.chest.2023.04.036 PMC10567931

[328] Corl KA, Prodromou M, Merchant RC, Gareen I, Marks S, Banerjee D, et al. The restrictive IV fluid trial in severe sepsis and septic shock (RIFTS): a randomized pilot study. Crit Care Med. 2019;47:951–9. 30985449 10.1097/CCM.0000000000003779 PMC6579683

[329] Hjortrup PB, Haase N, Bundgaard H, Thomsen SL, Winding R, Pettilä V, et al. Restricting volumes of resuscitation fluid in adults with septic shock after initial management: the CLASSIC randomised, parallel-group, multicentre feasibility trial. Intensive Care Med. 2016;42:1695–705. 27686349 10.1007/s00134-016-4500-7

[330] Charpentier J, Mira JP, Group ES. Efficacy and tolerance of hyperoncotic albumin administration in septic shock patients: the EARSS study. Intensive Care Med. 2011;37:S115.

[331] Maiwall R, Kumar A, Pasupuleti SSR, Hidam AK, Tevethia H, Kumar G, et al. A randomized-controlled trial comparing 20% albumin to plasmalyte in patients with cirrhosis and sepsis-induced hypotension [ALPS trial]. J Hepatol. 2022;77:670–82. 35460725 10.1016/j.jhep.2022.03.043

[332] De Backer D, Creteur J, Silva E. Effects of dopamine, norepinephrine, and epinephrine on the splanchnic circulation in septic shock: which is best? Crit Care Med. 2003;31:1659–67. 12794401 10.1097/01.CCM.0000063045.77339.B6

[333] Bouhemad B, Nicolas-Robin A, Arbelot C. Acute left ventricular dilatation and shock-induced myocardial dysfunction. Crit Care Med. 2009;37:441–7. 19114917 10.1097/CCM.0b013e318194ac44

[334] Romero-Bermejo FJ, Ruiz-Bailen M, Gil-Cebrian J. Sepsisinduced cardiomyopathy. Curr Cardiol Rev. 2011;7:163–83. 22758615 10.2174/157340311798220494 PMC3263481

[335] Gordon AC, Perkins GD, Singer M. Levosimendan for the prevention of acute organ dysfunction in sepsis. N Engl J Med. 2016;375:1638–48. 27705084 10.1056/NEJMoa1609409

[336] Caironi P, Tognoni G, Masson S, ALBIOS Study Investigators. Albumin replacement in patients with severe sepsis or septic shock. N Engl J Med. 2014;370:1412–21. 24635772 10.1056/NEJMoa1305727

[337] Sakr Y, Bauer M, Nierhaus A, SepNet—critical care trials group. Randomized controlled multicentre study of albumin replacement therapy in septic shock (ARISS): protocol for a randomized controlled trial. Trials. 2020;21:1002. 33287911 10.1186/s13063-020-04921-y PMC7720035

[338] https://classic.clinicaltrials.gov/ct2/show/NCT03654001. Accessed 7 Jul 2023

[339] Hébert PC, Wells G, Blajchman MA, Marshall J, Martin C, Pagliarello G, et al. A multicenter, randomized, controlled clinical trial of transfusion requirements in critical care. Transfusion requirements in critical care investigators, Canadian critical care trials group. N Engl J Med. 1999;340:409–17. 9971864 10.1056/NEJM199902113400601

[340] Nakamura T, Ebihara I, Shoji H. Treatment with polymyxin B-immobilized fiber reduces platelet activation in septic shock. Inflamm Res. 1999;48:171–5. 10344466 10.1007/s000110050442

[341] Cruz DN, Antonelli M, Fumagalli R. Early use of polymyxin B hemoperfusion in abdominal septic shock: the EUPHAS randomized controlled trial. JAMA. 2009;301:2445–52. 19531784 10.1001/jama.2009.856

[342] Payen DM, Guilhot J, Launey Y. Early use of polymyxin B hemoperfusion in patients with septic shock due to peritonitis: a multicenter randomized control trial. Intensive Care Med. 2015;41:975–84. 25862039 10.1007/s00134-015-3751-z PMC4477725

[343] Dellinger RP, Bagshaw SM, Antonelli M. Effect of targeted polymyxin B hemoperfusion on 28-day mortality in patients with septic shock and elevated endotoxin level: the EUPHRATES randomized clinical trial. JAMA. 2018;320:1455–63. 30304428 10.1001/jama.2018.14618 PMC6233793

[344] Safety and Efficacy of Polymyxin B Hemoperfusion (PMX) for Endotoxemic Septic Shock in Randomized, Open-Label Study (TIGRIS). ClinicalTrials.gov Identifier: NCT03901807, https://clinicaltrials.gov/ct2/show/NCT03901807.

[345] Peters E, Antonelli M, Wittebole X. A worldwide multicentre evaluation of the influence of deterioration or improvement of acute kidney injury on clinical outcome in critically ill patients with or without sepsis at ICU admission: results from the intensive care over nations audit. Crit Care. 2018;22:188. 30075798 10.1186/s13054-018-2112-z PMC6091052

[346] The STARRT-AKI Investigators, Bagshaw SM, Wald R, Adhikari NKJ. Timing of initiation of renal-replacement therapy in acute kidney injury. N Engl J Med. 2020;383:240–51. 32668114 10.1056/NEJMoa2000741

[347] Wald R, Adhikari NK, Smith OM. Comparison of standard and accelerated initiation of renal replacement therapy in acute kidney injury. Kidney Int. 2015;88:897–904. 26154928 10.1038/ki.2015.184

[348] Gaudry S, Hajage D, Schortgen F. Initiation strategies for renal-replacement therapy in the intensive care unit. N Engl J Med. 2016;375:122–33. 27181456 10.1056/NEJMoa1603017

[349] Barbar SD, Clere-Jehl R, Bourredjem A. Timing of renal-replacement therapy in patients with acute kidney injury and sepsis. N Engl J Med. 2018;379:1431–42. 30304656 10.1056/NEJMoa1803213

[350] Mehta RL, McDonald B, Gabbai FB. A randomized clinical trial of continuous versus intermittent dialysis for acute renal failure. Kidney Int. 2001;60:1154–63. 11532112 10.1046/j.1523-1755.2001.0600031154.x

[351] Gasparovic V, Filipovic-Grcic I, Merkler M, Pisl Z. Continuous renal replacement therapy (CRRT) or intermittent hemodialysis (IHD)—what is the procedure of choice in critically ill patients? Ren Fail. 2003;25:855–62. 14575293 10.1081/jdi-120024300

[352] Uehlinger DE, Jakob SM, Ferrari P. Comparison of continuous and intermittent renal replacement therapy for acute renal failure. Nephrol Dial Transplant. 2005;20:1630–7. 15886217 10.1093/ndt/gfh880

[353] Vinsonneau C, Camus C, Combes A. Continuous venovenous haemodiafiltration versus intermittent haemodialysis for acute renal failure in patients with multiple-organ dysfunction syndrome: a multicentre randomised trial. Lancet. 2006;368:379–85. 16876666 10.1016/S0140-6736(06)69111-3

[354] Schefold JC, von Haehling S, Pschowski R. The effect of continuous versus intermittent renal replacement therapy on the outcome of critically ill patients with acute renal failure (CONVINT): a prospective randomized controlled trial. Crit Care. 2014;18:R11. 24405734 10.1186/cc13188 PMC4056033

[355] Iwagami M, Yasunaga H, Noiri E. Choice of renal replacement therapy modality in intensive care units: data from a Japanese Nationwide administrative claim database. J Crit Care. 2015;30:381–5. 25434720 10.1016/j.jcrc.2014.11.003

[356] Tolwani AJ, Campbell RC, Stofan BS. Standard versus high-dose CVVHDF for ICU-related acute renal failure. J Am Soc Nephrol. 2008;19:1233–8. 18337480 10.1681/ASN.2007111173 PMC2396940

[357] Bellomo R, Cass A, Cole L. Intensity of continuous renal-replacement therapy in critically ill patients. N Engl J Med. 2009;361:1627–38. 19846848 10.1056/NEJMoa0902413

[358] Palevsky PM, Zhang JH, O’Connor TZ. Intensity of renal support in critically ill patients with acute kidney injury. N Engl J Med. 2008;359:7–20. 18492867 10.1056/NEJMoa0802639 PMC2574780

[359] Gando S, Iba T, Eguchi Y. A multicenter, prospective validation of disseminated intravascular coagulation diagnostic criteria for critically ill patients: comparing current criteria. Crit Care Med. 2006;34:625–31. 16521260 10.1097/01.ccm.0000202209.42491.38

[360] Iba T, Nisio MD, Levy JH. New criteria for sepsis-induced coagulopathy (SIC) following the revised sepsis definition: a retrospective analysis of a nationwide survey. BMJ Open. 2017;7: e017046. 10.1136/bmjopen-2017-017046 PMC5623518 28963294

[361] Taylor FBJ, Toh CH, Hoots WK. Towards definition, clinical and laboratory criteria, and a scoring system for disseminated intravascular coagulation. Thromb Haemost. 2001;86:1327–30. 11816725

[362] Vincent JL, Castro P, Hunt BJ. Thrombocytopenia in the ICU: disseminated intravascular coagulation and thrombotic microangiopathies-what intensivists need to know. Crit Care. 2018;22:158. 29895296 10.1186/s13054-018-2073-2 PMC5998546

[363] Iba T, Watanabe E, Umemura Y. Sepsis-associated disseminated intravascular coagulation and its differential diagnoses. J Intensive Care. 2019;7:32. 31139417 10.1186/s40560-019-0387-z PMC6528221

[364] Warkentin TE. Clinical picture of heparin-induced thrombocytopenia (HIT) and its differentiation from non-HIT thrombocytopenia. Thromb Haemost. 2016;116:813–22. 27656712 10.1160/TH16-06-0435

[365] Hulstein JJ, van Runnard Heimel PJ, Franx A. Acute activation of the endothelium results in increased levels of active von Willebrand factor in hemolysis, elevated liver enzymes and low platelets (HELLP) syndrome. J Thromb Haemost. 2006;4:2569–75. 16968329 10.1111/j.1538-7836.2006.02205.x

[366] Strnad P, Tacke F, Koch A, Trautwein C. Liver—guardian, modifier and target of sepsis. Nat Rev Gastroenterol Hepatol. 2017;14:55–66. 27924081 10.1038/nrgastro.2016.168

[367] Levy JH, Sniecinski RM, Welsby IJ, Levi M. Antithrombin: anti-inflammatory properties and clinical applications. Thromb Haemost. 2016;115:712–28. 26676884 10.1160/TH15-08-0687

[368] Allingstrup M, Wetterslev J, Ravn FB. Antithrombin III for critically ill patients. Cochrane Database Syst Rev. 2016;2:CD005370. 26858174 10.1002/14651858.CD005370.pub3 PMC6517014

[369] Wiedermann CJ, Kaneider NC. A systematic review of antithrombin concentrate use in patients with disseminated intravascular coagulation of severe sepsis. Blood Coagul Fibrinolysis. 2006;17:521–6. 16988545 10.1097/01.mbc.0000245302.18010.40

[370] Fourrier F, Chopin C, Huart JJ. Double-blind, placebo-controlled trial of antithrombin III concentrates in septic shock with disseminated intravascular coagulation. Chest. 1993;104:882–8. 8365305 10.1378/chest.104.3.882

[371] Inthorn D, Hoffmann JN, Hartl WH. Antithrombin III supplementation in severe sepsis: beneficial effects on organ dysfunction. Shock. 1997;8:328–34. 9361342 10.1097/00024382-199711000-00003

[372] Baudo F, Caimi TM, de Cataldo F. Antithrombin III (ATIII) replacement therapy in patients with sepsis and/or postsurgical complications: a controlled double-blind, randomized, multicenter study. Intensive Care Med. 1998;24:336–42. 9609411 10.1007/s001340050576

[373] Kienast J, Juers M, Wiedermann CJ. Treatment effects of high-dose antithrombin without concomitant heparin in patients with severe sepsis with or without disseminated intravascular coagulation. J Thromb Haemost. 2006;4:90–7. 16409457 10.1111/j.1538-7836.2005.01697.x

[374] Gando S, Saitoh D, Ishikura H. A randomized, controlled, multicenter trial of the effects of antithrombin on disseminated intravascular coagulation in patients with sepsis. Crit Care. 2013;17:R297. 24342495 10.1186/cc13163 PMC4057033

[375] Mohri M, Sugimoto E, Sata M. The inhibitory effect of recombinant human soluble thrombomodulin on initiation and extension of coagulation. A comparison with other anticoagulants. Thromb Haemost. 1999;82:1687–93. 10613656

[376] Yamakawa K, Levy JH, Iba T. Recombinant human soluble thrombomodulin in patients with sepsis-associated coagulopathy (SCARLET): an updated meta-analysis. Crit Care. 2019;23:302. 31488189 10.1186/s13054-019-2587-2 PMC6729086

[377] Zhang C, Wang H, Yang H. Recombinant human soluble thrombomodulin and short-term mortality of infection patients with DIC: a meta-analysis. Am J Emerg Med. 2016;34:1876–82. 27452884 10.1016/j.ajem.2016.06.001

[378] Valeriani E, Squizzato A, Gallo A. Efficacy and safety of recombinant human soluble thrombomodulin in patients with sepsis-associated coagulopathy: a systematic review and meta-analysis. J Thromb Haemost. 2020;18:1618–25. 32237269 10.1111/jth.14812

[379] Vincent JL, Francois B, Zabolotskikh I. Effect of a recombinant human soluble thrombomodulin on mortality in patients with sepsis-associated coagulopathy: the SCARLET randomized clinical trial. JAMA. 2019;321:1993–2002. 31104069 10.1001/jama.2019.5358 PMC6547077

[380] Hagiwara A, Tanaka N, Uemura T. Can recombinant human thrombomodulin increase survival among patients with severe septic-induced disseminated intravascular coagulation: a single-centre, open-label, randomised controlled trial. BMJ Open. 2016;6: e012850. 10.1136/bmjopen-2016-012850 PMC5223629 28039291

[381] Mori S, Ai T, Sera T. Human soluble recombinant thrombomodulin, ART-123, resolved early phase coagulopathies, but did not significantly alter the 28 day outcome in the treatment of DIC associated with infectious systemic inflammatory response syndromes. J Clin Med. 2019;8:1553. 31569648 10.3390/jcm8101553 PMC6832475

[382] Vincent JL, Ramesh MK, Ernest D. A randomized, double-blind, placebo-controlled, phase 2b study to evaluate the safety and efficacy of recombinant human soluble thrombomodulin, ART-123, in patients with sepsis and suspected disseminated intravascular coagulation. Crit Care Med. 2013;41:2069–79. 23979365 10.1097/CCM.0b013e31828e9b03

[383] Sawano H, Shigemitsu K, Yoshinaga Y, Tsuruoka A, Natsukawa T, Hayashi Y, et al. Combined therapy with antithrombin and recombinant human soluble thrombomodulin in patients with severe sepsis and disseminated intravascular coagulation. JJAAM. 2013;24:119–31.

[384] Iba T, Gando S, Saitoh D, Ikeda T, Anan H, Oda S, et al. Efficacy and bleeding risk of antithrombin supplementation in patients with septic disseminated intravascular coagulation: a third survey. Clin Appl Thromb Hemost. 2017;23:422–8. 27161759 10.1177/1076029616648405

[385] Iba T, Gando S, Saitoh D, Wada H, Di Nisio M, Thachil J. Antithrombin supplementation and risk of bleeding in patients with sepsis-associated disseminated intravascular coagulation. Thromb Res. 2016;145:46–50. 27479532 10.1016/j.thromres.2016.07.016

[386] Iba T, Hagiwara A, Saitoh D, Anan H, Ueki Y, Sato K, et al. Effects of combination therapy using antithrombin and thrombomodulin for sepsis-associated disseminated intravascular coagulation. Ann Intensive Care. 2017;7:110. 29098447 10.1186/s13613-017-0332-z PMC5668219

[387] Suzuki J, Sasabuchi Y, Hatakeyama S, Matsui H, Sasahara T, Morisawa Y, et al. The effect of antithrombin added to recombinant human-soluble thrombomodulin for severe community-acquired pneumonia-associated disseminated intravascular coagulation: a retrospective cohort study using a nationwide inpatient database. J Intensive Care. 2020;8:8. 31956416 10.1186/s40560-019-0419-8 PMC6958595

[388] Umegaki T, Kunisawa S, Nishimoto K, Kamibayashi T, Imanaka Y. Effectiveness of combined antithrombin and thrombomodulin therapy on in-hospital mortality in mechanically ventilated septic patients with disseminated intravascular coagulation. Sci Rep. 2020;10:4874. 32184456 10.1038/s41598-020-61809-2 PMC7078266

[389] Umemura Y, Yamakawa K, Hayakawa M, Kudo D, Fujimi S. Concomitant versus individual administration of antithrombin and thrombomodulin for sepsis-induced disseminated intravascular coagulation: a nationwide Japanese registry study. Clin Appl Thromb Hemost. 2018;24:734–40. 29471674 10.1177/1076029618755948 PMC6714866

[390] Totoki T, Makino Y, Yamakawa K, Koami H, Wada T, Ito T, et al. Effects of combination therapy of antithrombin and thrombomodulin for sepsis-associated disseminated intravascular coagulation: a systematic review and meta-analysis. Thromb J. 2024;22:10. 38225597 10.1186/s12959-023-00579-z PMC10788990

[391] Spyropoulos AC, Goldin M, Giannis D, Diab W, Wang J, Khanijo S, et al. Efficacy and safety of therapeutic-dose heparin vs standard prophylactic or intermediate-dose heparins for thromboprophylaxis in high-risk hospitalized patients with COVID-19: the HEP-COVID randomized clinical trial. JAMA Intern Med. 2021;181:1612–20. 34617959 10.1001/jamainternmed.2021.6203 PMC8498934

[392] Billett HH, Reyes-Gil M, Szymanski J, Ikemura K, Stahl LR, Lo Y, et al. Anticoagulation in COVID-19: effect of enoxaparin, heparin, and apixaban on mortality. Thromb Haemost. 2020;120:1691–9. 33186991 10.1055/s-0040-1720978 PMC7869055

[393] Zhang Z, Yan T, Ren D, Zhou J, Liu L, Li J, et al. Low-molecular-weight heparin therapy reduces 28-day mortality in patients with sepsis-3 by improving inflammation and coagulopathy. Front Med. 2023;10:1157775. 10.3389/fmed.2023.1157775 PMC10289000 37359014

[394] Fu S, Yu S, Wang L, Ma X, Li X. Unfractionated heparin improves the clinical efficacy in adult sepsis patients: a systematic review and meta-analysis. BMC Anesthesiol. 2022;22:28. 35062871 10.1186/s12871-021-01545-w PMC8777179

[395] Huang JJ, Zou ZY, Zhou ZP, Liu Y, Yang ZJ, Zhang JJ, et al. Effectiveness of early heparin therapy on outcomes in critically ill patients with sepsis-induced coagulopathy. Front Pharmacol. 2023;14:1173893. 37256226 10.3389/fphar.2023.1173893 PMC10225678

[396] Li X, Liu Z, Luo M, Xi Y, Li C, Wang S, et al. Therapeutic effect of low-molecular-weight heparin on adult sepsis: a meta-analysis. Ann Palliat Med. 2021;10:3115–27. 33849098 10.21037/apm-21-169

[397] Taccone FS, Stordeur P, De Backer D. Gamma-globulin levels in patients with community-acquired septic shock. Shock. 2009;32:379–85. 19295479 10.1097/SHK.0b013e3181a2c0b2

[398] Akatsuka M, Tatsumi H, Sonoda T. Low immunoglobulin G level is associated with poor outcomes in patients with sepsis and septic shock. J Microbiol Immunol Infect. 2021;54:728–32. 32859530 10.1016/j.jmii.2020.08.013

[399] Darenberg J, Ihendyane N, Sjölin J. Intravenous immunoglobulin G therapy in streptococcal toxic shock syndrome: a European randomized, double-blind, placebo-controlled trial. Clin Infect Dis. 2003;37:333–40. 12884156 10.1086/376630

[400] Werdan K, Pilz G, Bujdoso O. Score-based immunoglobulin G therapy of patients with sepsis: the SBITS study. Crit Care Med. 2007;35:2693–701. 18074471

[401] Madsen MB, Hjortrup PB, Hansen MB. Immunoglobulin G for patients with necrotising soft tissue infection (INSTINCT): a randomised, blinded, placebo-controlled trial. Intensive Care Med. 2017;43:1585–93. 28421246 10.1007/s00134-017-4786-0

[402] Dominioni D, Bianchi V, Imperatori A. High-dose intravenous IgG for treatment of severe surgical infections. Dig Surg. 2016;13:430–4.

[403] Burns ER, Lee V, Rubinstein A. Treatment of septic thrombocytopenia with immune globulin. J Clin Immunol. 1991;11:363–8. 1761642 10.1007/BF00918802

[404] De Simone C, Delogu G, Corbetta G. Intravenous immunoglobulins in association with antibiotics: a therapeutic trial in septic intensive care unit patients. Crit Care Med. 1988;16:23–6. 3276446 10.1097/00003246-198801000-00005

[405] Grundmann R, Hornung M. Immunoglobulin therapy in patients with endotoxemia and postoperative sepsis—a prospective randomized study. Prog Clin Biol Res. 1988;272:339–49. 3293080

[406] Marenović T, Filipović D, Lukić Z. High doses of immunoglobulins decrease mortality rate of surgical patients with severe intraabdominal infections and sepsis. Vojnosanit Pregl. 1998;55:71–4. 9623362

[407] Masaoka T, Hasegawa H, Takaku F. The efficacy of intravenous immunoglobulin in combination therapy with antibiotics for severe infections. Jpn J Chemother. 2000;48:199–217.

[408] Parks T, Wilson C, Curtis N. Polyspecific intravenous immunoglobulin in clindamycin-treated patients with streptococcal toxic shock syndrome: a systematic review and meta- analysis. Clin Infect Dis. 2018;67:1434–6. 29788397 10.1093/cid/ciy401 PMC6186853

[409] Bartoszko JJ, Elias Z, Rudziak P, Lo CKL. Prognostic factors for streptococcal toxic shock syndrome: systematic review and meta-analysis. BMJ Open. 2022;12: e063023. 10.1136/bmjopen-2022-063023 PMC9716873 36456018

[410] Marik PE, Khangoora V, Rivera R. Hydrocortisone, vitamin C, and thiamine for the treatment of severe sepsis and septic shock: a retrospective before-after study. Chest. 2017;151:1229–38. 27940189 10.1016/j.chest.2016.11.036

[411] Fujii T, Luethi N, Young PJ. Effect of vitamin C, hydrocortisone, and thiamine vs hydrocortisone alone on time alive and free of vasopressor support among patients with septic shock: the VITAMINS randomized clinical trial. JAMA. 2020;323:423–31. 31950979 10.1001/jama.2019.22176 PMC7029761

[412] Hwang SY, Ryoo SM, Park JE. Combination therapy of vitamin C and thiamine for septic shock: a multi-centre, double-blinded randomized, controlled study. Intensive Care Med. 2020;46:2015–25. 32780166 10.1007/s00134-020-06191-3 PMC7417779

[413] Lamontagne F, Masse MH, Menard J. Intravenous vitamin C in adults with sepsis in the intensive care unit. N Engl J Med. 2022;386:2387–98. 35704292 10.1056/NEJMoa2200644

[414] Lyu QQ, Zheng RQ, Chen QH. Early administration of hydrocortisone, vitamin C, and thiamine in adult patients with septic shock: a randomized controlled clinical trial. Crit Care. 2022;26:295. 36171582 10.1186/s13054-022-04175-x PMC9520942

[415] Rosengrave P, Spencer E, Williman J. Intravenous vitamin C administration to patients with septic shock: a pilot randomised controlled trial. Crit Care. 2022;26:26. 35073968 10.1186/s13054-022-03900-w PMC8786621

[416] Sevransky JE, Rothman RE, Hager DN. Effect of vitamin C, thiamine, and hydrocortisone on ventilator- and vasopressor-free days in patients with sepsis: the VICTAS randomized clinical trial. JAMA. 2021;325:742–50. 33620405 10.1001/jama.2020.24505 PMC7903252

[417] Chang P, Liao Y, Guan J. Combined treatment with hydrocortisone, vitamin C, and thiamine for sepsis and septic shock: a randomized controlled trial. Chest. 2020;158:174–82. 32243943 10.1016/j.chest.2020.02.065

[418] Moskowitz A, Huang DT, Hou PC. Effect of ascorbic acid, corticosteroids, and thiamine on organ injury in septic shock: the ACTS randomized clinical trial. JAMA. 2020;324:642–50. 32809003 10.1001/jama.2020.11946 PMC7435341

[419] Wacker DA, Burton SL, Berger JP. Evaluating vitamin C in septic shock: a randomized controlled trial of vitamin C monotherapy. Crit Care Med. 2022;50:e458–67. 34982738 10.1097/CCM.0000000000005427 PMC9005102

[420] Fowler AA 3rd, Syed AA, Knowlson S. Phase I safety trial of intravenous ascorbic acid in patients with severe sepsis. J Transl Med. 2014;12:32. 24484547 10.1186/1479-5876-12-32 PMC3937164

[421] Fowler AA 3rd, Truwit JD, Hite RD. Effect of vitamin C infusion on organ failure and biomarkers of inflammation and vascular injury in patients with sepsis and severe acute respiratory failure: the CITRIS-ALI randomized clinical trial. JAMA. 2019;322:1261–70. 31573637 10.1001/jama.2019.11825 PMC6777268

[422] Hussein AA, Sabry NA, Abdalla MS. A prospective, randomised clinical study comparing triple therapy regimen to hydrocortisone monotherapy in reducing mortality in septic shock patients. Int J Clin Pract. 2021;75: e14376. 34003568 10.1111/ijcp.14376

[423] Iglesias J, Vassallo AV, Patel VV. Outcomes of metabolic resuscitation using ascorbic acid, thiamine, and glucocorticoids in the early treatment of sepsis: The ORANGES trial. Chest. 2020;158:164–73. 32194058 10.1016/j.chest.2020.02.049

[424] Jamshidi MR, Zeraati MR, Forouzanfar B. Effects of triple combination of hydrocortisone, thiamine, and vitamin C on clinical outcome in patients with septic shock: a single-center randomized controlled trial. J Res Med Sci. 2021;26:47. 34484379 10.4103/jrms.JRMS_593_19 PMC8383994

[425] Mahmoodpoor A, Shadvar K, Sanaie S. Effect of vitamin C on mortality of critically ill patients with severe pneumonia in intensive care unit: a preliminary study. BMC Infect Dis. 2021;21:616. 34187382 10.1186/s12879-021-06288-0 PMC8240083

[426] Mohamed ZU, Prasannan P, Moni M. Vitamin C therapy for routine Care in Septic Shock (ViCTOR) trial: effect of intravenous vitamin C, thiamine, and hydrocortisone administration on inpatient mortality among patients with septic shock. Indian J Crit Care Med. 2020;24:653–61. 33024370 10.5005/jp-journals-10071-23517 PMC7519616

[427] Wani SJ, Mufti SA, Jan RA. Combination of vitamin C, thiamine and hydrocortisone added to standard treatment in the management of sepsis: results from an open label randomised controlled clinical trial and a review of the literature. Infect Dis. 2020;52:271–8. 10.1080/23744235.2020.1718200 31990246

[428] Zabet MH, Mohammadi M, Ramezani M. Effect of high-dose ascorbic acid on vasopressor’s requirement in septic shock. J Res Pharm Pract. 2016;5:94–100. 27162802 10.4103/2279-042X.179569 PMC4843590

[429] van den Berghe G, Wouters P, Weekers F, Verwaest C, Bruyninckx F, Schetz M, et al. Intensive insulin therapy in critically ill patients. N Engl J Med. 2001;345:1359–67. 11794168 10.1056/NEJMoa011300

[430] Finfer S, Chittock DR, Su SY, Blair D, Foster D, Dhingra V, et al. Intensive versus conventional glucose control in critically ill patients. N Engl J Med. 2009;360:1283–97. 19318384 10.1056/NEJMoa0810625

[431] Annane D, Cariou A, Maxime V, Azoulay E, D’Honneur G, Timsit JF, et al. Corticosteroid treatment and intensive insulin therapy for septic shock in adults: a randomized controlled trial. JAMA. 2010;303:341–8. 20103758 10.1001/jama.2010.2

[432] Arabi YM, Dabbagh OC, Tamim HM, Al-Shimemeri AA, Memish ZA, Haddad SH, et al. Intensive versus conventional insulin therapy: a randomized controlled trial in medical and surgical critically ill patients. Crit Care Med. 2008;36:3190–7. 18936702 10.1097/CCM.0b013e31818f21aa

[433] Arabi YM, Tamim HM, Dhar GS, Al-Dawood A, Al-Sultan M, Sakkijha MH, et al. Permissive underfeeding and intensive insulin therapy in critically ill patients: a randomized controlled trial. Am J Clin Nutr. 2011;93:569–77. 21270385 10.3945/ajcn.110.005074

[434] Bilotta F, Caramia R, Cernak I, Paoloni FP, Doronzio A, Cuzzone V, et al. Intensive insulin therapy after severe traumatic brain injury: a randomized clinical trial. Neurocrit Care. 2008;9:159–66. 18373223 10.1007/s12028-008-9084-9

[435] Bilotta F, Caramia R, Paoloni FP, Delfini R, Rosa G. Safety and efficacy of intensive insulin therapy in critical neurosurgical patients. Anesthesiology. 2009;110:611–9. 19237874 10.1097/ALN.0b013e318198004b

[436] Bilotta F, Spinelli A, Giovannini F, Doronzio A, Delfini R, Rosa G. The effect of intensive insulin therapy on infection rate, vasospasm, neurologic outcome, and mortality in neurointensive care unit after intracranial aneurysm clipping in patients with acute subarachnoid hemorrhage: a randomized prospective pilot trial. J Neurosurg Anesthesiol. 2007;19:156–60. 17592345 10.1097/ANA.0b013e3180338e69

[437] Bland DK, Fankhanel Y, Langford E, Lee M, Lee SW, Maloney C, et al. Intensive versus modified conventional control of blood glucose level in medical intensive care patients: a pilot study. Am J Crit Care. 2005;14:370–6. 16120888

[438] Brunkhorst FM, Engel C, Bloos F, Meier-Hellmann A, Ragaller M, Weiler N, et al. Intensive insulin therapy and pentastarch resuscitation in severe sepsis. N Engl J Med. 2008;358:125–39. 18184958 10.1056/NEJMoa070716

[439] Bruno A, Kent TA, Coull BM, Shankar RR, Saha C, Becker KJ, et al. Treatment of hyperglycemia in ischemic stroke (THIS): a randomized pilot trial. Stroke. 2008;39:384–9. 18096840 10.1161/STROKEAHA.107.493544

[440] Cappi SB, Noritomi DT, Velasco IT, Curi R, Loureiro TC, Soriano FG. Dyslipidemia: a prospective controlled randomized trial of intensive glycemic control in sepsis. Intensive Care Med. 2012;38:634–41. 22297666 10.1007/s00134-011-2458-z

[441] Chan RP, Galas FR, Hajjar LA, Bello CN, Piccioni MA, Auler JO Jr. Intensive perioperative glucose control does not improve outcomes of patients submitted to open-heart surgery: a randomized controlled trial. Clinics. 2009;64:51–60. 19142552 10.1590/S1807-59322009000100010 PMC2671976

[442] Coester A, Neumann CR, Schmidt MI. Intensive insulin therapy in severe traumatic brain injury: a randomized trial. J Trauma. 2010;68:904–11. 20032790 10.1097/TA.0b013e3181c9afc2

[443] Davies RR, Newton RW, McNeill GP, Fisher BM, Kesson CM, Pearson D. Metabolic control in diabetic subjects following myocardial infarction: difficulties in improving blood glucose levels by intravenous insulin infusion. Scott Med J. 1991;36:74–6. 1925506 10.1177/003693309103600303

[444] de Azevedo JR, de Araujo LO, da Silva WS, de Azevedo RP. A carbohydrate-restrictive strategy is safer and as efficient as intensive insulin therapy in critically ill patients. J Crit Care. 2010;25:84–9. 19327317 10.1016/j.jcrc.2008.10.011

[445] De La Rosa GC, Donado JH, Restrepo AH, Quintero AM, González LG, Saldarriaga NE, et al. Strict glycaemic control in patients hospitalised in a mixed medical and surgical intensive care unit: a randomised clinical trial. Crit Care. 2008;12:R120. 18799004 10.1186/cc7017 PMC2592751

[446] Farah R, Samokhvalov A, Zviebel F, Makhoul N. Insulin therapy of hyperglycemia in intensive care. Isr Med Assoc J. 2007;9:140–2. 17402320

[447] Giakoumidakis K, Eltheni R, Patelarou E, Theologou S, Patris V, Michopanou N, et al. Effects of intensive glycemic control on outcomes of cardiac surgery. Heart Lung. 2013;42:146–51. 23453011 10.1016/j.hrtlng.2012.12.007

[448] Green DM, O’Phelan KH, Bassin SL, Chang CW, Stern TS, Asai SM. Intensive versus conventional insulin therapy in critically ill neurologic patients. Neurocrit Care. 2010;13:299–306. 20697836 10.1007/s12028-010-9417-3

[449] Grey NJ, Perdrizet GA. Reduction of nosocomial infections in the surgical intensive-care unit by strict glycemic control. Endocr Pract. 2004;10:46–52. 15251640 10.4158/EP.10.S2.46

[450] Hsu CW, Sun SF, Lin SL, Huang HH, Wong KF. Moderate glucose control results in less negative nitrogen balances in medical intensive care unit patients: a randomized, controlled study. Crit Care. 2012;16:R56. 22480187 10.1186/cc11299 PMC3681385

[451] Iapichino G, Albicini M, Umbrello M, Sacconi F, Fermo I, Pavlovich R, et al. Tight glycemic control does not affect asymmetric-dimethylarginine in septic patients. Intensive Care Med. 2008;34:1843–50. 18504551 10.1007/s00134-008-1158-9

[452] Kalfon P, Giraudeau B, Ichai C, Guerrini A, Brechot N, Cinotti R, et al. Tight computerized versus conventional glucose control in the ICU: a randomized controlled trial. Intensive Care Med. 2014;40:171–81. 24420499 10.1007/s00134-013-3189-0

[453] McMullin J, Brozek J, McDonald E, Clarke F, Jaeschke R, Heels-Ansdell D, et al. Lowering of glucose in critical care: a randomized pilot trial. J Crit Care. 2007;22:112. 17548021 10.1016/j.jcrc.2006.08.002

[454] Mitchell I, Knight E, Gissane J, Tamhane R, Kolli R, Leditschke IA, et al. A phase II randomised controlled trial of intensive insulin therapy in general intensive care patients. Crit Care Resusc. 2006;8:289–93. 17227263

[455] Mohod V, Ganeriwal V, Bhange J. Comparison of intensive insulin therapy and conventional glucose management in patients undergoing coronary artery bypass grafting. J Anaesthesiol Clin Pharmacol. 2019;35:493–7. 31920233 10.4103/joacp.JOACP_61_17 PMC6939551

[456] Oksanen T, Skrifvars MB, Varpula T, Kuitunen A, Pettilä V, Nurmi J, et al. Strict versus moderate glucose control after resuscitation from ventricular fibrillation. Intensive Care Med. 2007;33:2093–100. 17928994 10.1007/s00134-007-0876-8

[457] Poole AP, Finnis ME, Anstey J, Bellomo R, Bihari S, Biradar V, et al. The effect of a liberal approach to glucose control in critically ill patients with type 2 diabetes: a multicenter, parallel-group, open-label randomized clinical trial. Am J Respir Crit Care Med. 2022;206:874–82. 35608484 10.1164/rccm.202202-0329OC

[458] Preiser JC, Devos P, Ruiz-Santana S, Mélot C, Annane D, Groeneveld J, et al. A prospective randomised multi-centre controlled trial on tight glucose control by intensive insulin therapy in adult intensive care units: the Glucontrol study. Intensive Care Med. 2009;35:1738–48. 19636533 10.1007/s00134-009-1585-2

[459] Santana-Santos EKP, Vieira RC, Oliveira LB, Ferretti-Rebustini RE, Menezes AF, et al. Impact of intensive glycemic control on acute renal injury: a randomized clinical trial. Acta Paul Enferm. 2019;32:592–9.

[460] Savioli M, Cugno M, Polli F, Taccone P, Bellani G, Spanu P, et al. Tight glycemic control may favor fibrinolysis in patients with sepsis. Crit Care Med. 2009;37:424–31. 19114908 10.1097/CCM.0b013e31819542da

[461] Umpierrez G, Cardona S, Pasquel F, Jacobs S, Peng L, Unigwe M, et al. Randomized controlled trial of intensive versus conservative glucose control in patients undergoing coronary artery bypass graft surgery: GLUCO-CABG trial. Diabetes Care. 2015;38:1665–72. 26180108 10.2337/dc15-0303 PMC4542267

[462] Van den Berghe G, Wilmer A, Hermans G, Meersseman W, Wouters PJ, Milants I, et al. Intensive insulin therapy in the medical ICU. N Engl J Med. 2006;354:449–61. 16452557 10.1056/NEJMoa052521

[463] Walters MR, Weir CJ, Lees KR. A randomised, controlled pilot study to investigate the potential benefit of intervention with insulin in hyperglycaemic acute ischaemic stroke patients. Cerebrovasc Dis. 2006;22:116–22. 16685123 10.1159/000093239

[464] Wang Y, Li JP, Song YL, Zhao QH. Intensive insulin therapy for preventing postoperative infection in patients with traumatic brain injury: a randomized controlled trial. Medicine. 2017;96: e6458. 28353579 10.1097/MD.0000000000006458 PMC5380263

[465] Singer P, Blaser AR, Berger MM, Alhazzani W, Calder PC, Casaer MP, et al. ESPEN guideline on clinical nutrition in the intensive care unit. Clin Nutr. 2019;38:48–79. 30348463 10.1016/j.clnu.2018.08.037

[466] Bernard GR, Wheeler AP, Russell JA, Schein R, Summer WR, Steinberg KP, et al. The effects of ibuprofen on the physiology and survival of patients with sepsis. The ibuprofen in sepsis study group. N Engl J Med. 1997;336:912–8. 9070471 10.1056/NEJM199703273361303

[467] Niven DJ, Stelfox HT, Leger C, Kubes P, Laupland KB. Assessment of the safety and feasibility of administering antipyretic therapy in critically ill adults: a pilot randomized clinical trial. J Crit Care. 2013;28:296–302. 23102531 10.1016/j.jcrc.2012.08.015

[468] Yang YL, Liu DW, Wang XT, Long Y, Zhou X, Chai WZ. Body temperature control in patients with refractory septic shock: too much may be harmful. Chin Med J. 2013;126:1809–13. 23673091

[469] Young P, Saxena M, Bellomo R, Freebairn R, Hammond N, van Haren F, et al. Acetaminophen for fever in critically ill patients with suspected infection. N Engl J Med. 2015;373:2215–24. 26436473 10.1056/NEJMoa1508375

[470] Janz DR, Bastarache JA, Rice TW, Bernard GR, Warren MA, Wickersham N, et al. Randomized, placebo-controlled trial of acetaminophen for the reduction of oxidative injury in severe sepsis: the acetaminophen for the reduction of oxidative injury in severe sepsis trial. Crit Care Med. 2015;43:534–41. 25474535 10.1097/CCM.0000000000000718 PMC4336619

[471] Vasikasin V, Rojdumrongrattana T, Chuerboonchai W, Siriwiwattana T, Thongtaeparak W, Niyasom S, et al. Effect of standard dose paracetamol versus placebo as antipyretic therapy on liver injury in adult dengue infection: a multicentre randomised controlled trial. Lancet Glob Health. 2019;7:e664–70. 31000133 10.1016/S2214-109X(19)30032-4

[472] Haupt MT, Jastremski MS, Clemmer TP, Metz CA, Goris GB. Effect of ibuprofen in patients with severe sepsis: a randomized, double-blind, multicenter study. The ibuprofen study group. Crit Care Med. 1991;19:1339–47. 1935150 10.1097/00003246-199111000-00006

[473] Apte NM, Karnad DR, Medhekar TP, Tilve GH, Morye S, Bhave GG. Gastric colonization and pneumonia in intubated critically ill patients receiving stress ulcer prophylaxis: a randomized, controlled trial. Crit Care Med. 1992;20:590–3. 1572183 10.1097/00003246-199205000-00008

[474] Basso N, Bagarani M, Materia A, Fiorani S, Lunardi P, Speranza V. Cimetidine and antacid prophylaxis of acute upper gastrointestinal bleeding in high risk patients: Controlled, randomized trial. Am J Surg. 1981;141:339–41. 7011078 10.1016/0002-9610(81)90191-4

[475] Ben-Menachem T. Prophylaxis for stress-related gastric Hemorrhage in the medical intensive care unit: a randomized, controlled. Single-Blind Study, Ann Intern Med. 1994;121:568. 8085688 10.7326/0003-4819-121-8-199410150-00003

[476] Burgess P, Larson GM, Davidson P, Brown J, Metz CA. Effect of ranitidine on intragastric pH and stress-related upper gastrointestinal bleeding in patients with severe head injury. Dig Dis Sci. 1995;40:645–50. 7895560 10.1007/BF02064385

[477] Chan KH, Lai ECS, Tuen H. Prospective double-blind placebo-controlled randomized trial on the use of ranitidine in preventing postoperative gastroduodenal complications in high-risk neurosurgical patients. J Neurosurg. 1995;82:413–7. 7861219 10.3171/jns.1995.82.3.0413

[478] Darlong V, Jayalakhsmi TS, Kaul HL, Tandon R. Stress ulcer prophylaxis in patients on ventilator. Trop Gastroenterol. 2003;24:124–8. 14978984

[479] Eddleston JM, Pearson RC, Holland J, Tooth JA, Vohra A, Doran BH. Prospective endoscopic study of stress erosions and ulcers in critically ill adult patients treated with either sucralfate or placebo. Crit Care Med. 1994;22:1949–54. 7988131

[480] El-Kersh K, Jalil B, McClave SA. Enteral nutrition as stress ulcer prophylaxis in critically ill patients: a randomized controlled exploratory study. J Crit Care. 2018;43:108–13. 28865339 10.1016/j.jcrc.2017.08.036

[481] Granholm A, Krag M, Marker S, Alhazzani W, Perner A, Møller MH. Predictors of gastrointestinal bleeding in adult ICU patients in the SUP-ICU trial. Acta Anaesthesiol Scand. 2021;65:792–800. 33635540 10.1111/aas.13805

[482] Groll A, Simon JB, Wigle RD, Taguchi K, Todd RJ, Depew WT. Cimetidine prophylaxis for gastrointestinal bleeding in an intensive care unit. Gut. 1986;27:135–40. 3485068 10.1136/gut.27.2.135 PMC1433215

[483] Gündoğan K, Karakoç E, Teke T. Effects of oral/enteral nutrition alone versus plus pantoprazole on gastrointestinal bleeding in critically ill patients with low risk factor: a multicenter, randomized controlled trial. Turk J Med Sci. 2020;50:776–83. 32151119 10.3906/sag-1911-42 PMC7379460

[484] Halloran LG, Zfass AM, Gayle WE, Wheeler CB, Miller JD. Prevention of acute gastrointestinal complications after severe head injury: a controlled trial of cimetidine prophylaxis. Am J Surg. 1980;139:44–8. 6985776 10.1016/0002-9610(80)90228-7

[485] Hanisch EW, Encke A, Naujoks F, Windolf J. A randomized, double-blind trial for stress ulcer prophylaxis shows no evidence of increased pneumonia. Am J Surg. 1998;176:453–7. 9874432 10.1016/s0002-9610(98)00239-6

[486] Hastings PR, Skillman JJ, Bushnell LS, Silen W. Antacid titration in the prevention of acute gastrointestinal bleeding: a controlled, randomized trial in 100 critically ill patients. N Engl J Med. 1978;298:1041–5. 25384 10.1056/NEJM197805112981901

[487] Kantorova I, Svoboda P, Scheer P. Stress ulcer prophylaxis in critically ill patients: a randomized controlled trial. Hepatogastroenterology. 2004;51:757–61. 15143910

[488] Karlstadt RG, Iberti TJ, Silverstein J. Comparison of cimetidine and placebo for the prophylaxis of upper gastrointestinal bleeding due to stress-related gastric mucosal damage in the intensive care unit. J Intensive Care Med. 1990;5:26–32.

[489] Kaushal S, Midha V, Sood A, Chopra SC, Gupta C. A comparative study of the effects of famotidine and sucralfate in prevention of upper gastro-intestinal bleeding in patients of head injury. Ind J Pharmacol. 2000;32:246.

[490] Lin CC, Hsu YL, Chung CS, Lee TH. Stress ulcer prophylaxis in patients being weaned from the ventilator in a respiratory care center: a randomized control trial. J Formos Med Assoc. 2016;115:19–24. 25676674 10.1016/j.jfma.2014.10.006

[491] Martin LF, Booth FV, Karlstadt RG. Continuous intravenous cimetidine decreases stress-related upper gastrointestinal hemorrhage without promoting pneumonia. Crit Care Med. 1993;21:19–30. 8420726 10.1097/00003246-199301000-00009

[492] Metz CA, Livingston DH, Smith JS, Larson GM, Wilson TH. Impact of multiple risk factors and ranitidine prophylaxis on the development of stress-related upper gastrointestinal bleeding: a prospective, multicenter, double-blind, randomized trial. The ranitidine head injury study group. Crit Care Med. 1993;21:1844–9. 8252888 10.1097/00003246-199312000-00010

[493] Muzlovič I, Štubljar D. Stress ulcer prophylaxis as a risk factor for tracheal colonization and hospital-acquired pneumonia in intensive care patients: impact on latency time for pneumonia. Acta Clin Croat. 2019;58:72–86. 31363328 10.20471/acc.2019.58.01.10 PMC6629202

[494] Nourian A, Mohammadi M, Beigmohammadi MT. Comparing efficacy of enteral nutrition plus ranitidine and enteral nutrition alone as stress ulcer prophylaxis. J Comp Eff Res. 2018;7:493–501. 29775083 10.2217/cer-2017-0098

[495] Otsuka T, Yagi Y, Shimazaki S, Yamamoto Y, Suzuki T, Mitsui KNM. Examination of the inhibitory effect of famotidine injection on increased gastric acid secretion due to cerebrovascular injury and head injury - a placebo-controlled, double-blind comparative trial. Med Cons New Remed. 1991;28:1–12.

[496] Pinilla JC, Oleniuk FH, Reed D, Malik B, Laverty WH. Does antacid prophylaxis prevent upper gastrointestinal bleeding in critically ill patients? Crit Care Med. 1985;13:646–50. 3874752 10.1097/00003246-198508000-00007

[497] Powell H, Morgan M, Li S, Baron J. Inhibition of gastric acid secretion in the intensive care unit after coronary artery bypass graft. A pilot control study of intravenous omeprazole by bolus and infusion, ranitidine and placebo. Theoret Surg. 1993;8:125–30.

[498] Reusser P, Gyr K, Scheidegger D, Buchmann B, Buser M, Zimmerli W. Prospective endoscopic study of stress erosions and ulcers in critically ill neurosurgical patients: current incidence and effect of acid-reducing prophylaxis. Crit Care Med. 1990;18:270–4. 2302950 10.1097/00003246-199003000-00004

[499] Ruiz-Santana S, Ortiz E, Gonzalez B, Bolaños J, Ruiz-Santana AJ, Manzano JL. Stress-induced gastroduodenal lesions and total parenteral nutrition in critically ill patients: frequency, complications, and the value of prophylactic treatment. A prospective, randomized study. Crit Care Med. 1991;19:887–91. 1905214 10.1097/00003246-199107000-00011

[500] Selvanderan SP, Summers MJ, Finnis ME. Pantoprazole or placebo for stress ulcer prophylaxis (POP-UP): randomized double-blind exploratory study. Crit Care Med. 2016;44:1842–50. 27635481 10.1097/CCM.0000000000001819

[501] Van Den Berg B, Van Blankenstein M. Prevention of stress-induced upper gastrointestinal bleeding by cimetidine in patients on assisted ventilation. Digestion. 1985;31:1–8. 3979676 10.1159/000199170

[502] Zinner MJ, Zuidema GD, Smith PL, Mignosa M. The prevention of upper gastrointestinal tract bleeding in patients in an intensive care unit. Surg Gynecol Obstet. 1981;153:214–20. 7017982

[503] Krag M, Marker S, Perner A. Pantoprazole in patients at risk for gastrointestinal bleeding in the ICU. N Engl J Med. 2018;379:2199–208. 30354950 10.1056/NEJMoa1714919

[504] Rixen D, Livingston DH, Loder P, Denny TN. Ranitidine improves lymphocyte function after severe head injury: results of a randomized, double-blind study. Crit Care Med. 1996;24:1787–92. 8917026 10.1097/00003246-199611000-00005

[505] Cloud ML, Offen W. Continuous infusions of nizatidine are safe and effective in the treatment of intensive care unit patients at risk for stress gastritis. Scand J Gastroenterol. 1994;29:29–34. 7863249 10.3109/00365529409091418

[506] Rumbus Z, Matics R, Hegyi P, Zsiboras C, Szabo I, Illes A, et al. Fever is associated with reduced, hypothermia with increased mortality in septic patients: a meta-analysis of clinical trials. PLoS ONE. 2017;12: e0170152. 28081244 10.1371/journal.pone.0170152 PMC5230786

[507] Kushimoto S, Abe T, Ogura H, Shiraishi A, Saitoh D, Fujishima S, et al. Impact of body temperature abnormalities on the implementation of sepsis bundles and outcomes in patients with severe sepsis: a retrospective sub-analysis of the focused outcome research on emergency care for acute respiratory distress syndrome, sepsis and trauma study. Crit Care Med. 2019;47:691–9. 30789402 10.1097/CCM.0000000000003688

[508] Drewry AM, Fuller BM, Skrupky LP, Hotchkiss RS. The presence of hypothermia within 24 hours of sepsis diagnosis predicts persistent lymphopenia. Crit Care Med. 2015;43:1165–9. 25793436 10.1097/CCM.0000000000000940 PMC4700928

[509] Brown DJA, Brugger H, Boyd J, Peter P. Accidental hypothermia. N Engl J Med. 2012;367:1930–8. 23150960 10.1056/NEJMra1114208

[510] Epstein E, Anna K. Accidental hypothermia. BMJ. 2006;332:706–9. 16565126 10.1136/bmj.332.7543.706 PMC1410860

[511] Wolberg AS, Meng ZH, Monroe DM, Hoffman M. A systematic evaluation of the effect of temperature on coagulation enzyme activity and platelet function. J Trauma. 2004;56:1221–8. 15211129 10.1097/01.ta.0000064328.97941.fc

[512] Young PJ, Bellomo R. Fever in sepsis: is it cool to be hot? Crit Care. 2014;18:109. 24521542 10.1186/cc13726 PMC4056432

[513] Matthew H, Ineke P, Alexandre AS, Maryse W, Marcus JS, Janneke H, et al. Opinions and management of hypothermic sepsis: results from an online survey. Ther Hypothermia Temp Manag. 2020;10:102–5. 31233381 10.1089/ther.2019.0002

[514] Paal P, Gordon L, Strapazzon G, Maeder MB, Putzer G, Walpoth B, et al. Accidental hypothermia–an update: the content of this review is endorsed by the International Commission for Mountain Emergency Medicine (ICAR MEDCOM). Scand J Trauma Resusc Emerg Med. 2016;24:111. 27633781 10.1186/s13049-016-0303-7 PMC5025630

[515] Watanabe M, Matsuyama T, Morita S, Ehara N, Miyamae N, Okada Y, et al. Impact of rewarming rate on the mortality of patients with accidental hypothermia: analysis of data from the J-point registry. Scand J Trauma Resusc Emerg Med. 2019;27:105. 31771645 10.1186/s13049-019-0684-5 PMC6880476

[516] Delbove A, Darreau C, Hamel JF, Asfar P, Lerolle N. Impact of endotracheal intubation on septic shock outcome: a post hoc analysis of the SEPSISPAM trial. J Crit Care. 2015;30:1174–8. 26410680 10.1016/j.jcrc.2015.08.018

[517] Karamchandani K, Wheelwright J, Yang AL, Westphal ND, Khanna AK, Myatra SN. Emergency airway management outside the operating room: current evidence and management strategies. Anesth Analg. 2021;133:648–62. 34153007 10.1213/ANE.0000000000005644

[518] Manthous CA, Hall JB, Kushner R, Schmidt GA, Russo G, Wood LD. The effect of mechanical ventilation on oxygen consumption in critically ill patients. Am J Respir Crit Care Med. 1995;151:210–4. 7812556 10.1164/ajrccm.151.1.7812556

[519] Mosier JM, Joshi R, Hypes C, Pacheco G, Valenzuela T, Sakles JC. The physiologically difficult airway. West J Emerg Med. 2015;16:1109–17. 26759664 10.5811/westjem.2015.8.27467 PMC4703154

[520] Sklar MC, Detsky ME. Emergent airway management of the critically ill patient: current opinion in critical care. Curr Opin Crit Care. 2019;25:597–604. 31490206 10.1097/MCC.0000000000000659

[521] De Jong A, Myatra SN, Roca O, Jaber S. How to improve intubation in the intensive care unit. Update on knowledge and devices. Intensive Care Med. 2022;48:1287–98. 35986748 10.1007/s00134-022-06849-0 PMC9391631

[522] Jaber S, Rollé A, Godet T, Terzi N, Riu B, Asfar P, et al. Effect of the use of an endotracheal tube and stylet versus an endotracheal tube alone on first-attempt intubation success: a multicentre, randomised clinical trial in 999 patients. Intensive Care Med. 2021;47:653–64. 34032882 10.1007/s00134-021-06417-y PMC8144872

[523] Schmitz M, Roux X, Huttner B. Streptococcal toxic shock syndrome in the intensive care unit. Ann Intensive Care. 2018;8:88. 30225523 10.1186/s13613-018-0438-y PMC6141408

[524] Nelson GE, Pondo T, Toews KA. Epidemiology of invasive group a streptococcal infections in the United States, 2005–2012. Clin Infect Dis. 2016;63:478–86. 27105747 10.1093/cid/ciw248 PMC5776658

[525] Lamagni TL, Neal S, Keshishian C. Predictors of death after severe streptococcus pyogenes infection. Emerg Infect Dis. 2009;15:1304–7. 19751599 10.3201/eid1508.090264

[526] Sriskandan S, Ferguson M, Elliot V. Human intravenous immunoglobulin for experimental streptococcal toxic shock: bacterial clearance and modulation of inflammation. J Antimicrob Chemother. 2006;58:117–24. 16670109 10.1093/jac/dkl173

[527] Bergsten H, Madsen MB, Bergey F. Correlation between immunoglobulin dose administered and plasma neutralization of streptococcal superantigens in patients with necrotizing soft tissue infections. Clin Infect Dis. 2020;71:1772–5. 31916575 10.1093/cid/ciaa022

[528] Morris PE, Berry MJ, Files DC. Standardized rehabilitation and hospital length of stay among patients with acute respiratory failure: a randomized clinical trial. JAMA. 2016;315:2694–702. 27367766 10.1001/jama.2016.7201 PMC6657499

[529] Schweickert WD, Pohlman MC, Pohlman AS. Early physical and occupational therapy in mechanically ventilated, critically ill patients: a randomized controlled trial. Lancet. 2009;373:1874–82. 19446324 10.1016/S0140-6736(09)60658-9 PMC9906655

[530] Hodgson CL, Bailey M, Bellomo R. A binational multicenter pilot feasibility randomized controlled trial of early goal-directed mobilization in the ICU. Crit Care Med. 2016;44:1145–52. 26968024 10.1097/CCM.0000000000001643

[531] Schaller SJ, Anstey M, Blobner M. Early, goal-directed mobilization in the surgical intensive care unit: a randomized controlled trial. Lancet. 2016;388:1377–88. 27707496 10.1016/S0140-6736(16)31637-3

[532] Brummel NE, Girard TD, Ely EW. Feasibility and safety of early combined cognitive and physical therapy for critically ill medical and surgical patients: the activity and cognitive therapy in ICU (ACT-ICU) trial. Intensive Care Med. 2014;40:370–9. 24257969 10.1007/s00134-013-3136-0 PMC3943568

[533] Segers J, Hermans G, Bruyninckx F. Feasibility of neuromuscular electrical stimulation in critically ill patients. J Crit Care. 2014;29:1082–8. 25108833 10.1016/j.jcrc.2014.06.024

[534] Campos DR, Bueno TBC, Anjos JSGG. Early neuromuscular electrical stimulation in addition to early mobilization improves functional status and decreases hospitalization days of critically ill patients. Crit Care Med. 2022;50:1116–26. 35412472 10.1097/CCM.0000000000005557

[535] Kho ME, Truong AD, Zanni JM. Neuromuscular electrical stimulation in mechanically ventilated patients: a randomized, sham-controlled pilot trial with blinded outcome assessment. J Crit Care. 2015;30:32–9. 25307979 10.1016/j.jcrc.2014.09.014 PMC4268169

[536] Koutsioumpa E, Makris D, Theochari A. Effect of transcutaneous electrical neuromuscular stimulation on myopathy in intensive care patients. Am J Crit Care. 2018;27:495–503. 30385541 10.4037/ajcc2018311

[537] Nakanishi N, Oto J, Tsutsumi R. Effect of electrical muscle stimulation on upper and lower limb muscles in critically ill patients: a two-center randomized controlled trial. Crit Care Med. 2020;48:e997-1003. 32897665 10.1097/CCM.0000000000004522

[538] Routsi C, Gerovasili V, Vasileiadis I. Electrical muscle stimulation prevents critical illness polyneuromyopathy: a randomized parallel intervention trial. Crit Care. 2010;14:R74. 20426834 10.1186/cc8987 PMC2887197

[539] Silva PE, de Cássia MR, Livino-de-Carvalho K. Neuromuscular electrical stimulation in critically ill traumatic brain injury patients attenuates muscle atrophy, neurophysiological disorders, and weakness: a randomized controlled trial. J Intensive Care. 2019;7:59. 31890221 10.1186/s40560-019-0417-x PMC6909464

[540] Abu-Khaber HA, Abouelela AMZ, Abdelkarim EM. Effect of electrical muscle stimulation on prevention of ICU acquired muscle weakness and facilitating weaning from mechanical ventilation. Alex J Med. 2013;39:309–15.

[541] Baron MV, Silva PE, Koepp J. Efficacy and safety of neuromuscular electrical stimulation in the prevention of pressure injuries in critically ill patients: a randomized controlled trial. Ann Intensive Care. 2022;12:53. 35695996 10.1186/s13613-022-01029-1 PMC9188909

[542] Cebeci GC, Cebeci H, Kucuk MP. Neuromuscular electrical stimulator as a protective treatment against intensive care unit muscle wasting in sepsis/septic shock patients. J Coll Physicians Surg Pak. 2022;32:1300–7. 36205275 10.29271/jcpsp.2022.10.1300

[543] Chen YH, Hsiao HF, Li LF. Effects of electrical muscle stimulation in subjects undergoing prolonged mechanical ventilation. Respir Care. 2019;64:262–71. 30723168 10.4187/respcare.05921

[544] Nakamura K, Kihata A, Naraba H. Efficacy of belt electrode skeletal muscle electrical stimulation on reducing the rate of muscle volume loss in critically ill patients: a randomized controlled trial. J Rehabil Med. 2019;51:705–11. 31544949 10.2340/16501977-2594

[545] Ojima M, Takegawa R, Hirose T. Hemodynamic effects of electrical muscle stimulation in the prophylaxis of deep vein thrombosis for intensive care unit patients: a randomized trial. J Intensive Care. 2017;5:9. 28101364 10.1186/s40560-016-0206-8 PMC5237178

[546] Dos Santos FV, Cipriano G Jr, Vieira L. Neuromuscular electrical stimulation combined with exercise decreases duration of mechanical ventilation in ICU patients: a randomized controlled trial. Physiother Theory Pract. 2020;36:580–8. 30321084 10.1080/09593985.2018.1490363

[547] Shen SY, Lee CH, Lin RL. Electric muscle stimulation for weaning from mechanical ventilation in elder patients with severe sepsis and acute respiratory failure - a pilot study. Int J Gerontol. 2017;11:41–5.

[548] Bao W, Yang J, Li M. Prevention of muscle atrophy in ICU patients without nerve injury by neuromuscular electrical stimulation: a randomized controlled study. BMC Musculoskelet Disord. 2022;23:780. 35974369 10.1186/s12891-022-05739-2 PMC9380284

[549] Schmidt K, Worrack S, Von Korff M. Effect of a primary care management intervention on mental health-related quality of life among survivors of sepsis: a randomized clinical trial. JAMA. 2016;315:2703–11. 27367877 10.1001/jama.2016.7207 PMC5122319

[550] Cuthbertson BH, Rattray J, Campbell MK. The PRaCTICaL study of nurse led, intensive care follow-up programmes for improving long term outcomes from critical illness: a pragmatic randomised controlled trial. BMJ. 2009;339: b3723. 19837741 10.1136/bmj.b3723 PMC2763078

[551] Valsø Å, Rustøen T, Småstuen M. Effect of nurse-led consultations on post-traumatic stress and sense of coherence in discharged ICU patients with clinically relevant post-traumatic stress symptoms-a randomized controlled trial. Crit Care Med. 2020;48:e1218–25. 33048906 10.1097/CCM.0000000000004628

[552] Jackson JC, Ely EW, Morey MC. Cognitive and physical rehabilitation of intensive care unit survivors: results of the RETURN randomized controlled pilot investigation. Crit Care Med. 2012;40:1088–97. 22080631 10.1097/CCM.0b013e3182373115 PMC3755871

[553] Connolly B, Thompson A, Douiri A. Exercise-based rehabilitation after hospital discharge for survivors of critical illness with intensive care unit-acquired weakness: a pilot feasibility trial. J Crit Care. 2015;30:589–98. 25703957 10.1016/j.jcrc.2015.02.002 PMC4416081

[554] McWilliams DJ, Benington S, Atkinson D. Outpatient-based physical rehabilitation for survivors of prolonged critical illness: a randomized controlled trial. Physiother Theory Pract. 2016;32:179–90. 27043264 10.3109/09593985.2015.1137663

[555] Battle C, James K, Temblett P. Supervised exercise rehabilitation in survivors of critical illness: a randomised controlled trial. J Intensive Care Soc. 2019;20:18–26. 30792758 10.1177/1751143718767061 PMC6376574

[556] Elliott D, McKinley S, Alison J. Health-related quality of life and physical recovery after a critical illness: a multi-centre randomised controlled trial of a home-based physical rehabilitation program. Crit Care. 2011;15:R142. 21658221 10.1186/cc10265 PMC3219014

[557] Batterham AM, Bonner S, Wright J. Effect of supervised aerobic exercise rehabilitation on physical fitness and quality-of-life in survivors of critical illness: an exploratory minimized controlled trial (PIX study). Br J Anaesth. 2014;113:130–7. 24607602 10.1093/bja/aeu051 PMC4062299

[558] Vitacca M, Barbano L, Vanoglio F. Does 6-month home caregiver-supervised physiotherapy improve post-critical care outcomes? A randomized controlled trial. Am J Phys Med Rehabil. 2016;95:571–9. 26829083 10.1097/PHM.0000000000000441

[559] McDowell K, O’Neill B, Blackwood B. Effectiveness of an exercise programme on physical function in patients discharged from hospital following critical illness: a randomised controlled trial (the REVIVE trial). Thorax. 2017;72:600–9. 27852953 10.1136/thoraxjnl-2016-208723

[560] Shelly AG, Prabhu NS, Jirange P. Quality of life improves with individualized home-based exercises in critical care survivors. Indian J Crit Care Med. 2017;21:89–93. 28250604 10.4103/ijccm.IJCCM_433_16 PMC5330060

[561] Vasilevskis EE, Ely EW, Speroff T, Pun BT, Boehm L, Dittus RS. Reducing iatrogenic risks: ICU-acquired delirium and weakness—crossing the quality chasm. Chest. 2010;138:1224–33. 21051398 10.1378/chest.10-0466 PMC4694109

[562] Davidson JE, Harvey MA, Schuller J. Post-intensive care syndrome: what to do and how to prevent it. Am Nurse Today. 2013;8:32–8.

[563] Harvey MA, Davidson JE. Postintensive care syndrome: right care, right now and later. Crit Care Med. 2016;44:381–5. 26771784 10.1097/CCM.0000000000001531

[564] Barnes-Daly MA, Phillips G, Ely EW. Improving hospital survival and reducing brain dysfunction at seven California community hospitals: implementing PAD guidelines via the ABCDEF bundle in 6,064 patients. Crit Care Med. 2017;45:171–8. 27861180 10.1097/CCM.0000000000002149

[565] Pun BT, Balas MC, Barnes-Daly MA, Thompson JL, Aldrich JM, Barr J, et al. Caring for critically ill patients with the ABCDEF bundle: results of the ICU liberation collaborative in over 15,000 adults. Crit Care Med. 2019;47:3–14. 30339549 10.1097/CCM.0000000000003482 PMC6298815

[566] https://www.sccm.org/clinical-resources/iculiberation-home/abcdef-bundles. Accessed 12 Nov 2024.

[567] Lautrette A, Darmon M, Megarbane B. A communication strategy and brochure for relatives of patients dying in the ICU. N Engl J Med. 2007;356:469–78. 17267907 10.1056/NEJMoa063446

[568] Azoulay E, Pochard F, Chevret S. Impact of a family information leaflet on effectiveness of information provided to family members of intensive care unit patients: a multicenter, prospective, randomized, controlled trial. Am J Respir Crit Care Med. 2002;165:438–42. 11850333 10.1164/ajrccm.165.4.200108-006oc

[569] Robin S, Labarriere C, Sechaud G. Information pamphlet given to relatives during the end-of-life decision in the ICU: an assessor-blinded, randomized controlled trial. Chest. 2021;159:2301–8. 33549600 10.1016/j.chest.2021.01.072

[570] Greenberg JA, Basapur S, Quinn TV. Daily written care summaries for families of critically ill patients: a randomized controlled trial. Crit Care Med. 2022;50:1296–305. 35607975 10.1097/CCM.0000000000005583

[571] Chiang VCL, Lee RLP, Ho MF. Fulfilling the psychological and information need of the family members of critically ill patients using interactive mobile technology: a randomised controlled trial. Intensive Crit Care Nurs. 2017;41:77–83. 28438476 10.1016/j.iccn.2017.03.006

[572] Rodríguez-Huerta MD, Álvarez-Pol M, Fernández-Catalán ML. An informative nursing intervention for families of patients admitted to the intensive care unit regarding the satisfaction of their needs: the INFOUCI study. Intensive Crit Care Nurs. 2019;55:102755. 31515006 10.1016/j.iccn.2019.102755

[573] Berwick DM, Kotagal M. Restricted visiting hours in ICUs: time to change. JAMA. 2004;292:736–7. 15304472 10.1001/jama.292.6.736

[574] Cappellini E, Bambi S, Lucchini A. Open intensive care units: a global challenge for patients, relatives, and critical care teams. Dimens Crit Care Nurs. 2014;33:181–93. 24895947 10.1097/DCC.0000000000000052

[575] Rosa RG, Falavigna M, Silva DB, ICU visits study group investigators and the Brazilian research in intensive care network (BRICNet). Effect of flexible family visitation on delirium among patients in the intensive care unit: the ICU visits randomized clinical trial. JAMA. 2019;322:216–28. 31310297 10.1001/jama.2019.8766 PMC6635909

[576] Rosa RG, Pellegrini JAS, Moraes RB. Mechanism of a flexible ICU visiting policy for anxiety symptoms among family members in Brazil: a path mediation analysis in a cluster-randomized clinical trial. Crit Care Med. 2021;49:1504–12. 33870915 10.1097/CCM.0000000000005037

[577] Wu Y, Wang G, Zhang Z. Efficacy and safety of unrestricted visiting policy for critically ill patients: a meta-analysis. Crit Care. 2022;26:267. 36064613 10.1186/s13054-022-04129-3 PMC9446669

[578] Moss SJ, Rosgen BK, Lucini F. Psychiatric outcomes in ICU patients with family visitation: a population-based retrospective cohort study. Chest. 2022;162:578–87. 35271840 10.1016/j.chest.2022.02.051

[579] Ministry of Health, Labour and Welfare. The practice guidelines for process of decision making regarding treatment in the end of life care (in Japanese). https://www.mhlw.go.jp/file/06-Seisakujouhou-10800000-Iseikyoku/0000197721.pdf. Accessed 26 May 2023.

[580] Detering KM, Hancock AD, Reade MC. The impact of advance care planning on end of life care in elderly patients: randomised controlled trial. BMJ. 2010;340:1345. 10.1136/bmj.c1345 PMC2844949 20332506

[581] Kredentser MS, Blouw M, Marten N. Preventing posttraumatic stress in ICU survivors: a single-center pilot randomized controlled trial of ICU diaries and psychoeducation. Crit Care Med. 2018;46:1914–22. 30119073 10.1097/CCM.0000000000003367

[582] Nielsen AH, Angel S, Egerod I. The effect of family-authored diaries on posttraumatic stress disorder in intensive care unit patients and their relatives: a randomised controlled trial (DRIP-study). Aust Crit Care. 2020;33:123–9. 30795978 10.1016/j.aucc.2019.01.004

[583] Garrouste-Orgeas M, Flahault C, Vinatier I. Effect of an ICU diary on posttraumatic stress disorder symptoms among patients receiving mechanical ventilation: a randomized clinical trial. JAMA. 2019;322:229–39. 31310299 10.1001/jama.2019.9058 PMC6635906

[584] Sayde GE, Stefanescu A, Conrad E. Implementing an intensive care unit (ICU) diary program at a large academic medical center: results from a randomized control trial evaluating psychological morbidity associated with critical illness. Gen Hosp Psychiatry. 2020;66:96–102. 32763640 10.1016/j.genhosppsych.2020.06.017 PMC7329691

[585] Wang S, Xin HN. Chung Lim Vico C, Effect of an ICU diary on psychiatric disorders, quality of life, and sleep quality among adult cardiac surgical ICU survivors: a randomized controlled trial. Crit Care. 2020;24:81. 32143655 10.1186/s13054-020-2797-7 PMC7060606

[586] Rice RN, Qualls BW, Carey MG. Use of diaries for family members of intensive care unit patients to reduce long-term PTSD: a pilot study. J Patient Exp. 2022;9:5681. 10.1177/23743735221105681 PMC9168849 35677228

[587] Jones C, Skirrow P, Griffiths RD. Post-traumatic stress disorder-related symptoms in relatives of patients following intensive care. Intensive Care Med. 2004;30:456–60. 14767589 10.1007/s00134-003-2149-5

[588] Torke AM, Wocial LD, Johns SA. The family navigator: a pilot intervention to support intensive care unit family surrogates. Am J Crit Care. 2016;25:498–507. 27802950 10.4037/ajcc2016730 PMC5117831

[589] Bohart S, Egerod I, Bestle MH. Recovery programme for ICU survivors has no effect on relatives’ quality of life: secondary analysis of the RAPIT-study. Intensive Crit Care Nurs. 2018;47:39–45. 29606480 10.1016/j.iccn.2018.03.002

[590] Cox CE, Hough CL, Carson SS. Effects of a telephone- and web-based coping skills training program compared with an education program for survivors of critical illness and their family members. A randomized clinical trial. Am J Respir Crit Care Med. 2018;197:66–78. 28872898 10.1164/rccm.201704-0720OC

[591] White DB, Angus DC, Shields AM. A randomized trial of a family-support intervention in intensive care units. N Engl J Med. 2018;378:2365–75. 29791247 10.1056/NEJMoa1802637

[592] Ågren S, Eriksson A, Fredrikson M. The health promoting conversations intervention for families with a critically ill relative: a pilot study. Intensive Crit Care Nurs. 2019;50:103–10. 29731406 10.1016/j.iccn.2018.04.007

[593] Lester EG, Mace RA, Bannon SM. Can a dyadic resiliency program improve quality of life in cognitively intact dyads of neuro-ICU survivors and informal caregivers? Results from a pilot RCT. Neurocrit Care. 2021;35:756–66. 33880701 10.1007/s12028-021-01222-3 PMC10947170

[594] Gawlytta R, Kesselmeier M, Scherag A. Internet-based cognitive-behavioural writing therapy for reducing post-traumatic stress after severe sepsis in patients and their spouses (REPAIR): results of a randomised-controlled trial. BMJ Open. 2022;12: e050305. 10.1136/bmjopen-2021-050305 PMC8915321 35264337

[595] Schlapbach LJ, Watson RS, Sorce LR, Argent AC, Menon K, Hall MW, Society of critical care medicine pediatric sepsis definition task force, et al. International consensus criteria for pediatric sepsis and septic shock. JAMA. 2024;331:665–74. 38245889 10.1001/jama.2024.0179 PMC10900966

[596] Sanchez-Pinto LN, Bennett TD, DeWitt PE, Russell S, Rebull MN. Martin B development and validation of the Phoenix criteria for pediatric sepsis and septic shock. JAMA. 2024;331:675–86. 38245897 10.1001/jama.2024.0196 PMC10900964

[597] Evans IVR, Phillips GS, Alpern ER, Angus DC, Friedrich ME, Kissoon N, et al. Association between the New York sepsis care mandate and in-hospital mortality for pediatric sepsis. JAMA. 2018;320:358–67. 30043064 10.1001/jama.2018.9071 PMC6500448

[598] Weiss SL, Fitzgerald JC, Pappachan J, Wheeler D, Jaramillo-Bustamante JC, Salloo A, et al. Global epidemiology of pediatric severe sepsis: the sepsis prevalence, outcomes, and therapies study. Am J Respir Crit Care Med. 2015;191:1147–57. 25734408 10.1164/rccm.201412-2323OC PMC4451622

[599] Ruth A, McCracken CE, Fortenberry JD, Hall M, Simon HK, Hebbar KB. Pediatric severe sepsis: current trends and outcomes from the pediatric health information systems database. Pediatr Crit Care Med. 2014;15:828–38. 25226500 10.1097/PCC.0000000000000254

[600] Watson RS, Carcillo JA, Linde-Zwirble WT, Clermont G, Lidicker J, Angus DC. The epidemiology of severe sepsis in children in the United States. Am J Respir Crit Care Med. 2003;167:695–701. 12433670 10.1164/rccm.200207-682OC

[601] Niedner MF, Huskins WC, Colantuoni E, Muschelli J, Harris JM 2nd, Rice TB, et al. Epidemiology of central line-associated bloodstream infections in the pediatric intensive care unit. Infect Control Hosp Epidemiol. 2011;32:1200–8. 22080659 10.1086/662621

[602] Zervou FN, Zacharioudakis IM, Ziakas PD, Mylonakis E. MRSA colonization and risk of infection in the neonatal and pediatric ICU: a meta-analysis. Pediatrics. 2014;133:e1015–23. 24616358 10.1542/peds.2013-3413

[603] Prout AJ, Talisa VB, Carcillo JA, Decker BK, Yende S. Bacterial and fungal etiology of sepsis in children in the United States: reconsidering empiric therapy. Crit Care Med. 2020;48:e192–9. 31789702 10.1097/CCM.0000000000004140 PMC7875440

[604] Weiss SL, Peters MJ, Alhazzani W, Agus MSD, Flori HR, Inwald DP, et al. Surviving sepsis campaign international guidelines for the management of septic shock and sepsis-associated organ dysfunction in children. Pediatr Crit Care Med. 2020;1:e52-106. 10.1097/PCC.0000000000002198 32032273

[605] Ventura AM, Shieh HH, Bousso A, Góes PF, de Cássia FO, Fernandes I, de Souza DC, et al. Double-blind prospective randomized controlled trial of dopamine versus epinephrine as first-line vasoactive drugs in pediatric septic shock. Crit Care Med. 2015;43:2292–302. 26323041 10.1097/CCM.0000000000001260

[606] Ramaswamy KN, Singhi S, Jayashree M, Bansal A, Nallasamy K. Double-blind randomized clinical trial comparing dopamine and epinephrine in pediatric fluid-refractory hypotensive septic shock. Pediatr Crit Care Med. 2016;17:e502–12. 27673385 10.1097/PCC.0000000000000954

[607] Choong K, Bohn D, Fraser DD, Gaboury I, Hutchison JS, Joffe AR, et al. Vasopressin in pediatric vasodilatory shock: a multicenter randomized controlled trial. Am J Respir Crit Care Med. 2009;180:632–9. 19608718 10.1164/rccm.200902-0221OC

[608] Yildizdas D, Yapicioglu H, Celik U, Sertdemir Y, Alhan E. Terlipressin as a rescue therapy for catecholamine-resistant septic shock in children. Intensive Care Med. 2008;34:511–7. 18092150 10.1007/s00134-007-0971-x

[609] Patregnani JT, Sochet AA, Klugman D. Short-term peripheral vasoactive infusions in pediatrics: where is the harm? Pediatr Crit Care Med. 2017;18:e378–81. 28617763 10.1097/PCC.0000000000001230

[610] Taylor RW, Palagiri AV. Central venous catheterization. Crit Care Med. 2007;35:1390–6. 17414086 10.1097/01.CCM.0000260241.80346.1B

[611] Lampin ME, Rousseaux J, Botte A, Sadik A, Cremer R, Leclerc F. Noradrenaline use for septic shock in children: doses routes of administration and complications. Acta Paediatr. 2012;101:e426–30. 22568565 10.1111/j.1651-2227.2012.02725.x

[612] Owen VS, Rosgen BK, Cherak SJ, Ferland A, Stelfox HT, Fiest KM, et al. Adverse events associated with administration of vasopressor medications through a peripheral intravenous catheter: a systematic review and meta-analysis. Crit Care. 2021;25:146. 33863361 10.1186/s13054-021-03553-1 PMC8050944

[613] El-Nawawy A, Khater D, Omar H, Wali Y. Evaluation of early corticosteroid therapy in management of pediatric septic shock in pediatric intensive care patients: a randomized clinical study. Pediatr Infect Dis J. 2017;36:155–9. 27798546 10.1097/INF.0000000000001380

[614] Valoor HT, Singhi S, Jayashree M. Low-dose hydrocortisone in pediatric septic shock: an exploratory study in a third world setting. Pediatr Crit Care Med. 2009;10:121–5. 19057445 10.1097/PCC.0b013e3181936ab3

[615] Menon K, McNally D, O’Hearn K, Acharya A, Wong HR, Lawson M, Canadian critical care trials group, et al. A randomized controlled trial of corticosteroids in pediatric septic shock: a pilot feasibility study. Pediatr Crit Care Med. 2017;18:505–12. 28406862 10.1097/PCC.0000000000001121 PMC5457353

[616] Lacroix J, Hébert PC, Hutchison JS, Hume HA, Tucci M, Ducruet T, TRIPICU investigators, Canadian critical care trials group, Pediatric acute lung injury and sepsis investigators network, et al. Transfusion strategies for patients in pediatric intensive care units. N Engl J Med. 2007;356:1609–19. 17442904 10.1056/NEJMoa066240

[617] Akyildiz B, Ulgen Tekerek N, Pamukcu O, Dursun A, Karakukcu M, et al. Comprehensive analysis of Liberal and restrictive transfusion strategies in pediatric intensive care unit. J Trop Pediatr. 2018;64:118–25. 28575484 10.1093/tropej/fmx037

[618] Elshinawy M, Kamal M, Nazir H, Khater D, Hassan R, Elkinany H, et al. Sepsis-related anemia in a pediatric intensive care unit: transfusion-associated outcomes. Transfusion. 2020;60:S4–9. 32134129 10.1111/trf.15688

[619] Agus MS, Steil GM, Wypij D, Costello JM, Laussen PC, Langer M, SPECS Study Investigators, et al. Tight glycemic control versus standard care after pediatric cardiac surgery. N Engl J Med. 2012;367:1208–19. 22957521 10.1056/NEJMoa1206044 PMC3501680

[620] Agus MS, Wypij D, Hirshberg EL, Srinivasan V, Faustino EV, Luckett PM. Tight glycemic control in critically ill children. N Engl J Med. 2017;376:729–41. 28118549 10.1056/NEJMoa1612348 PMC5444653

[621] Jeschke MG, Kulp GA, Kraft R, Finnerty CC, Mlcak R, et al. Intensive insulin therapy in severely burned pediatric patients: a prospective randomized trial. Am J Respir Crit Care Med. 2010;182:351–9. 20395554 10.1164/rccm.201002-0190OC PMC2921599

[622] Macrae D, Grieve R, Allen E, Sadique Z, Morris K, Pappachan J, CHiP investigators, et al. A randomized trial of hyperglycemic control in pediatric intensive care. N Engl J Med. 2014;370:107–18. 24401049 10.1056/NEJMoa1302564

[623] Vlasselaers D, Milants I, Desmet L, Wouters PJ, Vanhorebeek I, van den Heuvel I, et al. Intensive insulin therapy for patients in paediatric intensive care: a prospective, randomised controlled study. Lancet. 2009;373:547–56. 19176240 10.1016/S0140-6736(09)60044-1

[624] Davidson JE, Jones C, Bienvenu OJ. Family response to critical illness: postintensive care syndrome-family. Crit Care Med. 2012;40:618–24. 22080636 10.1097/CCM.0b013e318236ebf9

[625] Alejandria MM, Lansang MA, Dans LF, Mantaring JB 3rd. Intravenous immunoglobulin for treating sepsis, severe sepsis and septic shock. Cochrane Database Syst Rev. 2013;2013:CD001090. 24043371 10.1002/14651858.CD001090.pub2 PMC6516813

[626] Busani S, Damiani E, Cavazzuti I, Donati A, Girardis M. Intravenous immunoglobulin in septic shock: review of the mechanisms of action and meta-analysis of the clinical effectiveness. Minerva Anestesiol. 2016;82:559–72. 26474267

[627] Aubron C, Berteau F, Sparrow RL. Intravenous immunoglobulin for adjunctive treatment of severe infections in ICUs. Curr Opin Crit Care. 2019;25:417–22. 31335381 10.1097/MCC.0000000000000639

[628] van der Poll T, Shankar-Hari M, Wiersinga WJ. The immunology of sepsis. Immunity. 2021;54:2450–64. 34758337 10.1016/j.immuni.2021.10.012

[629] El-Nawawy A, El-Kinany H, Hamdy El-Sayed M, Boshra N. Intravenous polyclonal immunoglobulin administration to sepsis syndrome patients: a prospective stud in a pediatric intensive acre unit. J Trop Pediatr. 2005;51:271–8. 15917261 10.1093/tropej/fmi011

[630] INIS Collaborative Group, Brocklehurst P, Farrell B, King A, Juszczak E, Darlow B, Haque K, et al. Treatment of neonatal sepsis with intravenous immune globulin. N Engl J Med. 2011;365:1201–11. 21962214 10.1056/NEJMoa1100441

[631] Ohlsson A, Lacy JB. Intravenous immunoglobulin for suspected or proven infection in neonates. Cochrane Database Syst Rev. 2020;1:CD001239. 31995649 10.1002/14651858.CD001239.pub6 PMC6988993

[632] Kadri SS, Swihart BJ, Bonne SL, Hohmann SF, Hennessy LV, Louras P, et al. Impact of intravenous immunoglobulin on survival in necrotizing fasciitis with vasopressor-dependent shock: a propensity score-matched analysis from 130 US hospitals. Clin Infect Dis. 2017;64:877–85. 28034881 10.1093/cid/ciw871 PMC5850528

