# Supplementary material for: The Japanese Clinical Practice Guidelines for Management of Sepsis and Septic Shock 2024
Source: J Intensive Care. 2025 Mar 14;13:15. doi: 10.1186/s40560-025-00776-0 (PMC11907869; doi:10.1186/s40560-025-00776-0)
Supplement: Supplementary file 1 — Additional file 1 [file 40560_2025_776_MOESM1_ESM.pdf]

Additional file. Selection of empiric antimicrobials for each infectious disease

Source of infection	Patient background / Condition		Causative pathogens	Choice of antimicrobials	Remarks
Pneumonia[a]	Community-acquired	Other than the reasons listed below	*Streptococcus pneumoniae*, *Haemophilus influenzae, Klebsiella spp*., *Mycoplasma pneumoniae, Legionella pneumophila*	CTRX 2g, every 24 h [1,2] ± AZM 500mg, every 24 h[1,2]	See CQ2–5 for Legionella risk.
		Secondary pneumonia with influenza infection, necrotizing pneumonia	Above + *Staphylococcus aureus* (including community-acquired MRSA)	CTRX 2g, every 24 h [1,3] ± VCM [1,3] [h]	See CQ2–5 for MRSA risk.
	Healthcare-associated/ ventilator-associated		*S. pneumoniae, E. coli, Pseudomonas aeruginosa, S. aureus*	CFPM 2g, every 8 h or TAZ/PIPC 4.5g, every 8 h ± VCM[1] [h]	Antimicrobial selection identical to community-acquired pneumonia is applicable at an early stage or when there is no risk of resistant bacteria. See CQ2–5 for MRSA risk.
	Impared cell-mediated immunity + no prevention of *Pneumocystis jirovecii* + bilateral shadows		*Pneumocystis jirovecii*	ST trimethoprim 240–320 mg, every 8 h or pentamidine 4 mg/kg, every 24 h[1]	ST: trimethoprim 15mg/kg/day≒Japanese ST mixture (1 tablet or 1g of trimethoprim is 80mg) 3–4 tablets or 3–4g, every 8 h.
Urinary tract infection	Community-acquired (low risk of ESBL-producing bacteria)		*E. coli*	CTRX 1-2g, every 24 h [1]	See CQ2–4 for ESBL-producing bacteria risk.
	Community-acquired (high risk of ESBL-producing bacteria)			CMZ 1-2g, every 8 h [4,5] or TAZ/PIPC 4.5g, every 8 h [6] or MEPM 1g, every 8 h [1]	
	Healthcare-associated		*E. coli, Klebsiella* spp., *Enterobacter* spp., *P. aeruginosa*, *Enterococcus* spp.	TAZ/PIPC 4.5g, every 8 h or MEPM 1g, every 8 h ± VCM [1] [h]	VCM is added when Gram staining shows Staphylococcus-like Gram-positive cocci.

Biliary tract / intra-abdominal infection	Community-acquired (low risk of ESBL-producing bacteria)	*E. coli*, anaerobic bacteria such as *Bacteroides* spp.	SBT/ABPC 3g, every 6 h [7] or CTRX 2g, every 24 h ＋ MNZ 500mg, every 8 h [7]	See CQ2–4 for ESBL-producing bacteria risk. Check antibiogram to see if SBT/ABPC can be selected.
	Community-acquired (high risk of ESBL-producing bacteria)		CMZ 1-2g, every 8 h [7] or TAZ/PIPC 4.5g, every 8 h [1]	
	Healthcare-associated	*E. coli,* anaerobic bacteria such as *Bacteroides spp., Enterobacter spp., P. aeruginosa, Enterococcus spp*.± *Candida spp*.	TAZ/PIPC 4.5g, every 8 h or CFPM 2g, every 8 h＋ MNZ 500mg, every 8 h or MEPM 1g, every 8 h [1, 7] ± MCFG 100mg, every 24 h [1]	See CQ2–5 for Candida risk.
Necrotic soft tissue infection [b]	Monomicrobial infection suspected (Gram-positive cocci or Gram-positive rods)	β-hemolytic Streptococci, *Clostridium* spp., rarely *S. aureus* (including community-acquired MRSA)	CTRX 2g, every 24 h or SBT/ABPC 3g, every 6 h± VCM [h] ± CLDM 600mg, every 8 h [1,8]	See CQ2–5 for MRSA risk. CLDM is intended for suppressing toxin production in toxic shock syndrome.
	Polymicrobial infection suspected (diabetic, Fournier's gangrene)	*S. aureus, E. coli*, anerobic bacteria	MEPM 1g, every 8 h or TAZ/PIPC 4.5g, every 8 h± VCM [1,8] [h]	
	Exposure to seawater / freshwater	*Aeromonas spp., Vibrio vulnificus*	CTRX 2g, every 24 h＋ MINO 100mg, every 12 h [1,8]	
Vertebral osteomyelitis [c]	Community-acquired	MSSA, *Streptococcus* spp., rarely *S. pneumoniae*, Gram-negative bacilli	CEZ 2g, every 8 h [1] or CTRX 2g, every 24 h [1]	See CQ2–5 for MRSA risk.
	Healthcare-associated	*S. aureus*, Gram-negative bacilli	CFPM 2g, every 12 h + VCM [1] [h]	
Endocarditis [d]	Native valve: without MRSA risk	MSSA, *Streptococcus spp*., *Enterococcus* spp.	SBT/ABPC 3g, every 6 h [1] or CTRX 2g, every 24 h ＋ ABPC 2g, every 4 h [1,9]	Select "CTRX+ABPC" when there is a high possibility of enterococcal infection. Select CTRX 2g every 12 h if there is an intracranial disseminated lesion.
	Native valve: with MRSA risk	Above + MRSA	CTRX 2g, every 24 h＋ VCM [1,9] [h]	Select CTRX 2 g every 12 h if there is an intracranial disseminated lesion. See CQ2–5 for MRSA risk.

	Prosthetic valve or pacemaker infection	Above + *Staphylococcus epidermidis*, Gram-negative bacilli	CTRX 2g, every 24 h or CFPM 2g, every 12 h＋ VCM[1, 9) h)] or VCM＋GM 2-3mg/kg, every 24 h ± RFP 600mg, every 24 h	
Mycotic aneurysm[e)]	Community-acquired	*S. aureus, Salmonella spp*., Gram-negative bacilli	CTRX 2g, every 12 h ± VCM [h)]	See CQ2–5 for MRSA risk.
	Prosthetic vascular graft infections	*S. aureus, S. epidermidis, P. aeruginosa*	TAZ/PIPC 4.5g, every 8 h＋ VCM [h)]	
Catheter-related bloodstream infections	Intravascular catheter	*S. epidermidis, S. aureus* (including MRSA), *E. coli, P. aeruginosa, ± Candida spp.*	VCM [h)]＋ CFPM 2g, every 8-12 h ± MCFG 100mg, every 24 h [1)]	See CQ2–5 for Candida risk
Meningitis[f)]	Community-acquired (patient younger than 50 years)	*S. pneumoniae, Neisseria meningitidis*	CTRX 2g, every 12 h＋ VCM[1,10) h)]	
	Community-acquired (patient older than 50 years, cell-mediated immunodeficiency)	*S. pneumoniae, N. meningitidis, Listeria monocytogenes*	ABPC 2g, every 4 h＋ CTRX 2g, every 12 h＋ VCM[1,10) h)]	
	Post-neurosurgery or shunt-related meningitis	MRSA, *P. aeruginosa*	CAZ or CFPM or MEPM (2g, every 8 h)＋ VCM[1,10) h)]	
Unknown or systemic source[g)]	Community-acquired	*S. pneumoniae, N. meningitidis,β-hemolytic streptococcus, E. coli*	CTRX 2g, every 24 h [1)]	See section on meningitis if there is a possibility of meningitis
	Healthcare-associated	*P. aeruginosa*, MRSA	CFPM 2g, every 8 h or TAZ/PIPC 4.5g, every 8 h or MEPM 1g, every 8 h＋ VCM [h)]	
	Toxic shock syndrome	*S. aureus, β-hemolytic streptococcus, Clostridium spp*.	CTRX 2g, every 24 h or SBT/ABPC 3g, every 6 h＋ CLDM 600 mg, every 8 h± VCM [h)]	See CQ2–5 for MRSA risk
	Rickettsia endemic areas	Japanese spotted fever, scrub typhus	MINO 100mg, every 12 h [11)]	
	Febrile neutropenia	*Pseudomonas aeruginosa*, MRSA	CFPM 2g, every 12 h＋ VCM[1) h)]	See CQ2–4 for anti-Pseudomonal drugs.
	Postsplenectomy	*S. pneumoniae*, *N. meningitidis, H. influenzae, Capnocytophaga spp.*	When there is no possibility of meningitis: CTRX 2 g, every 24 h[1)]	See section on meningitis if there is a possibility of [illegible]

	Shock + rash	Purpura fulminans (*N. meningitidis, S. pneumoniae*), *Rickettsia* spp.	CTRX 2g, every 12 h + VCM[1) h)] + MINO 100 mg, every 12 h [11,12)]	See section on endocarditis if there is a possibility of endocarditis

This table is based on guides published by the Japanese Association for Infectious Diseases and the Japanese Society of Chemotherapy (JAID/JSC). This table is to be used as a reference for decision-making, taking into account information on individual situations and local epidemiology.

ABPC, ampicillin; AZM, azithromycin; CAZ, ceftazidime; CFPM, cefepime; CLDM, clindamycin; CMZ, cefmetazole; CTRX, ceftriaxone; GM, gentamycin; MCFG, micafungin; MEPM, meropenem; MINO, minocycline; MNZ, metronidazole; RFP, rifampicin; SBT/ABPC, sulbactam/ampicillin; ST, sulfamethoxazole/trimethoprim; TAZ/PIPC, tazobactam/piperacillin; VCM, vancomycin; ESBL, Eextended-spectrum β-lactamase; MRSA, methicillin-resistant *Staphylococcus aureus*; MSSA, methicillin-sensitve *Staphylococcus aureus;*

a) Pneumonia: *S. aureus* (including MRSA) can be a causative bacterium in addition to the usual causes of community-acquired pneumonia following influenza virus infection or necrotizing pneumonia.
b) Necrotic soft tissue infection: Causative bacteria can be estimated from the patient background (exposure history, underlying disease) and clinical course.
c) Vertebral osteomyelitis: Empiric treatment is indicated when complications of sepsis are present [13]. The regimen of empiric treatment is not established, but options were described based on the JAID/JSC infectious disease treatment guides [1]
d) Endocarditis: Concomitant use of GM in native valve endocarditis was previously recommended for *Staphylococcus aureus* [1], but this is no longer recommended in recently [9]. We presented an option for endocarditis of the prosthetic valve that does not include GM as an empiric treatment when the causative organism is uncertain, considering the nephrotoxicity of GM.
e) Mycotic aneurysm: There is no description in the JAID/JSC infectious disease treatment guidelines and no established recommendation exists [1, 14].
h)Catheter-related bloodstream infections: options were presented based on the JAID/JSC infectious disease treatment guidelines [5]
f) Meningitis: Options were presented based on the JAID/JSC infectious disease treatment guidelines [1, 10]
g) Unknown or systemic sources: Options were presented based on the JAID/JSC infectious disease treatment guidelines

1) JAID/JSC infectious disease treatment guideline 2019. Japanese Association for Infectious Diseases / Japanese Society of Chemotherapy. Life Science Publishing, Tokyo. 2023.

2) Martin-Loeches I, Torres A, Nagavci B, et al. ERS/ESICM/ESCMID/ALAT guidelines for the management of severe community-acquired pneumonia Intensive Care Med. 2023; 49: 615-32.

3) Chertow DS, Memoli MJ. Bacterial coinfection in influenza: A grand rounds review. JAMA. 2013; 309: 275-82.

4) Matsumura Y, Yamamoto M, Nagao M, et al. Multicenter Retrospective Study of Cefmetazole and Flomoxef for Treatment of Extended-Spectrum-β-Lactamase-Producing Escherichia Coli Bacteremia. Antimicrob Agents Chemother. 2015; 59: 5107-13.

5) Fukuchi T, Iwata K, Kobayashi S, et al. Cefmetazole for bacteremia caused by ESBL-producing enterobacteriaceae comparing with carbapenems BMC Infect Dis. 2016; 16: 427.

6) Harris PNA, Tambyah PA, Lye DC, et al. Effect of piperacillintazobactam vs meropenem on 30-day mortality for patients with e coli or Klebsiella pneumoniae bloodstream infection and ceftriaxone resistance. JAMA. 2018; 320: 984-94.

7) Gomi H, Solomkin JS, Schlossberg D, et al. Tokyo Guidelines 2018: antimicrobial therapy for acute cholangitis and cholecystitis. J Hepatobiliary Pancreat Sci. 2018; 25: 3-16.

8) Hua C, Urbina T, Bosc R, et al. Necrotising soft-tissue infections. Lancet Infect Dis. 2023; 23: e81-e94.

9) Nakatani S, Ohara T, Ashihara K, et al. JCS 2017 Guideline on Prevention and Treatment of Infective Endocarditis. Circ J. 2019;83:1767-1809.

10) Practical Guideline for Bacterial Meningitis 2014. Japanese Society of Neurology. Nankodo, Tokyo. 2014.

11) Sando E. Rickettsial infection. Hospitalist. 2017; 5: 519-28.

12) IASR 31-5, Rickettsia japonica. https://idsc.nih.go.jp/iasr/31/363/dj363b.html Accessed August 20, 2023.

13) Berbari EF, Kanj SS, Kowalski T, et al. 2015 Infectious Diseases Society of America (IDSA) Clinical Practice Guidelines for the Diagnosis and Treatment of Native Vertebral Osteomyelitis in Adults. Clin Infect Dis. 2015; 61: e26-46.

14) Wilson WR, Bower TC, Creager MA, et al. Vascular Graft Infections, Mycotic Aneurysms, and Endovascular Infections: A Scientific Statement from the American Heart Association. Circulation. 2016; 134: e412-60.

15) Antibacterial TDM Guidelines 2022. Japanese Society of Chemotherapy. https://www.chemotherapy.or.jp/uploads/ffile/guideline/tdm2022.pdf
