# Supplementary material for: The Japanese Clinical Practice Guidelines for Management of Sepsis and Septic Shock 2024
Source: J Intensive Care. 2025 Mar 14;13:15. doi: 10.1186/s40560-025-00776-0 (PMC11907869; doi:10.1186/s40560-025-00776-0)
Supplement: Supplementary file 2 — Additional file 2 [file 40560_2025_776_MOESM2_ESM.pdf]

Additional file: Selection of targeted antimicrobials by causative microorganism

Causative microorganism	Source of infection	Susceptibility results	Options	Alternatives	Remarks
**Gram-positive cocci in clusters [GPC in clusters]**					
Staphylococcus aureus	Catheter-related bloodstream infection, vertebral osteomyelitis / septic arthritis / iliopsoas abscess, native valve endocarditis (without intracranial dissemination), pneumonia	MSSA (PCG: S & CEZ: S)<br>* When determining "PCG: S", non-producer of penicillinase must be confirmed.	PCG 4,000,000 units, every 4–6 h [1-3] or ABPC 2g, every 4-6 h [4]<br>(endocarditis: every 4 h; other: every 4–6 h)	CEZ	
		MSSA (PCG: R & CEZ: S)	CEZ 2g, every 8 h [4-6]		Concomitant use of GM is not recommended [5]
		MRSA (CEZ: R & VCM: S)	VCM initial dose 25–30 mg/kg and subsequent doses 15–20 mg/kg, every 12 h [4-9]	DAP (excluding pneumonia) or TEIC or LZD [4, 5, 8, 9]	Target AUC value for VCM is 400-600 μg・h/mL [i]
	Native valve endocarditis (with intracranial dissemination), post-operative meningitis (including cerebrospinal fluid shunt infection)	MSSA (PCG: S & CEZ: S)<br>*When determining "PCG: S", non-producer of penicillinase must be confirmed.	PCG 4,000,000 units, every 4–6 h [1-3] or ABPC 2g, every 4-6 h [4]<br>(endocarditis: every 4 h; other: every 4–6 h)	Avoid CEZ	
		MSSA (CEZ: S)	CTRX 2g, every 12 h or CFPM 2g , every 8h or MEPM 2g, every 8 h [5,10]	Avoid CEZ	CTX is suggested in ESC 2015 [6]
		MRSA (CEZ: R & VCM: S)	VCM initial dose 25–30 mg/kg and subsequent doses 15–20 mg/kg, every 12 h [4-9]	DAP or TEIC or LZD [5, 8, 9]	Target AUC value for VCM is 400-600 μg・h/mL [i].<br>VCM+RFP (optionally, in BSAC 2012 [11])
	Prosthetic valve endocarditis	GM: S & RFP: S	Each regimen of native valve endocarditis (mentioned above) + GM 2–3 mg/kg, every 24 h ± oral RFP 600 mg once a day (combination of three drugs) [5-7, 9]		
		GM: R, AMK or LVFX: S	Use AMK or LVFX instead of GM		Concomitant use of GM for 2 weeks. Target GM levels: 3–5 μg/mL at peak, less than 1 μg/mL at trough [5,8]. See section on "Coagulase Negative *Staphylococcus* (CNS)" (see next section) for RFP addition
	Toxic shock	CLDM: S	Consider adding CLDM 600 mg, every 8 h [12] for above anti-staphylococcal regimen		

	syndrome	CLDM: R & LZD: S	Consider adding CLDM 600 mg, every 8 h or LZD 600 mg, every 12 h [12] for above anti-staphylococcal regimen		CLDM is used for toxin production suppression even when susceptibility is "resistant" [13].
Coagulase-Negative Staphylococci (CNS)	Catheter-related bloodstream infections, prosthetic valve endocarditis, prosthetic joint infection	・Susceptibility-based selection is similar with that for *Staphylococcus aureus*. → see section on "*Staphylococcus aureus*" (above).<br>・RFP can be considered when prosthetic valve or joint is preserved. → RFP should not use solely due to rapid development of resistance. There is expert opinion on avoiding its use when there is a large quantity of bacteria. Susceptibility test results serve as a reference [4-7].			
**Gram-positive cocci in chains [GPC in chains]**					
Streptococcus pneumoniae *Note that PCG susceptibility criteria differ for meningitis and non-meningitis	Other than meningitis (e.g., pneumonia)	PCG: S (MIC ≦ 2μg/mL)	PCG 2,000,000 units, every 4 h or ABPC 2 g, every 6–8 h [4] (PCG 4,000,000 units, every 4 h or ABPC 2 g, every 4 h for endocarditis/invasive infection)	CTRX	
		PCG : I or R (MIC ≧ 4μg/mL)	CTRX 2g, every 24 h [4]	VCM or LVFX (if S)	
	Meningitis	PCG: S (MIC ≦ 0.06μg/mL)	PCG 4,000,000 units, every 4 h [4, 14] or ABPC 2g, every 4 h [4,15,16]	CTRX	
		PCG: R (MIC ≦ 0 .12 μg/mL) & CTRX: S (MIC ≦ 0.5μg/mL)	CTRX 2g , every 12 h [4,14]	CFPM [10]	
		PCG: R (MIC ≧ 0.12μg/mL) & CTRX: I or R (MIC ≧ 1.0μg/mL)	VCM initial dose 25–30 mg/kg and subsequent doses 15–20 mg/kg, every 12 h + CTRX 2 g, every 12 h [4,8,14] (consider RFP addition if CTRX MIC>2 μg/mL& RFP: S, and RFP addition)[4,15,16]		Target VCM AUC value is 400-600 μg・h/mL[i]
Group A, B, C, F, G *Streptococcus β*	Bacteremia, soft tissue infection	PCG: S	PCG 2,000,000–4,000,000 units, every 4 h [4] or ABPC 2g, every 4-6 h	CEZ or CTRX	CLDM is used for toxin production suppression purposes even when susceptibility is "resistant".
	Toxic shock syndrome	PCG: S	Each above-mentioned regimen + CLDM 600 mg, every 8 h [4,17]		
		PCG MIC ≦ 0.12μg/mL	PCG 4,000,000 units, every 4 h [4] or ABPC 2g, every 4-6 h [5]	CTRX [4]	PCG can be continuously infused for 24 h [4], or divided between 6-h intervals [6,7]. 2,000,000–3,000,000 units, every 4 h is also an option (native valve [6,7], prosthetic valve [6])

Viridans Streptococci, *S. gallolyticus* (*S. bovis*)	Endocarditis	PCG MIC = 0.25μg/mL	PCG 4,000,000 units, every 4 h or ABPC 2 g, every 4 h + GM 3 mg/kg, every 24 h (or 1 mg/kg, 2–3 times per day) [4-8]	CTRX (if MIC ≦ 0.5 μg/mL)＋GM	PCG can be continuously infused for 24 h [4]. Target GM levels: 3–5 μg/mL at peak, less than 1 μg/mL at trough [5,8]. Concomitant administration of GM for 2 weeks in case of native valve, 6 weeks in case of prosthetic valve.
		PCG MIC ≧ 0.5	Consult with infectious disease specialists [5-7].		
	Other than endocarditis (e.g., pneumonia, bacteremia, febrile neutropenia)	PCG: S	PCG 2,000,000–3,000,000 units, every 4–6 h or ABPC 2 g, every 6–8 h [4,18]	CTRX	Continuous infusion of PCG for 24 h can be selected[4].
		PCG: I/R & CTRX: S	CTRX 2g , every 24 h [18]		
		PCG: I / R & CTRX: R & VCM: S	VCM initial dose 25–30 mg/kg and subsequent doses 15–20 mg/kg, every 12 h [18]		
Enterococcus spp.	Endocarditis	PCG: S	(1) When MIC is 500 or more μg/mL in GM synergy screening tests: PCG 4,000,000 units, every 4 h or ABPC 2 g, every 4 h + GM 3 mg/kg, every 24 h (or 1 mg/kg, 2–3 times per day) [4-7]<br>(2) When MIC for GM is MIC > 500 μg/mL in GM synergy screening test, or when there is no combined use of GM: ABPC 2 g, every 4 h + CTRX 2 g, every 12 h [4-7]		Implement GM synergy screening test for endocarditis. Target GM levels: 3–5 μg/mL at peak, less than 1 μg/mL at trough [5, 8].
		PCG: R (MIC ≧ 16μg/mL) & VCM: S	When MIC for GM is 500 or more μg/mL in GM synergy screening test: VCM (initial dose, 25-30 mg/kg and subsequent doses, 15-20 mg/kg, every 12h [8]) + GM 3mg/kg, every 24 h (or 1 mg/kg, 2-3times per day)[4,5]	SBT / ABPC: if S SBT/ABPC+ GM [6,7]	Target GM levels: 3–5 μg/mL at peak, less than 1 μg/mL at trough [5,8].<br>Target VCM AUC value is 400-600 μg・h/mL[i].
		VCM: R (VRE)	LZD or DAP＋ABPC [6,7,19]		Consult with infectious disease specialists
	Other than endocarditis	PCG: S	PCG 3,000,000 units, every 4 h or ABPC 2 g, every 4–6 h [4]		
		PCG: R & VCM: S	VCM initial dose, 25–30 mg/kg and subsequent doses, 15–20 mg/kg, every 12 h [8]		
**Gram-positive rods [GPR]**					
Bacillus spp. (Other than *Bacillus anthracis*)	Catheter-related bloodstream infections, etc.	VCM: S	VCM initial dose, 25–30 mg/kg and subsequent doses, 15–20 mg/kg, every 12 h [8]	CLDM [4]	

Corynebacteri-um spp.	Catheter-related bloodstream infection, prosthetic infection, etc.	VCM: S	VCM initial dose, 25–30 mg/kg and subsequent doses, 15–20 mg/kg, every 12 h [4,8]	PCG (if S) or TEIC or LZD (if S)[4]	
Listeria monocytogenes	Meningitis	ABPC: S	ABPC 2g, every 4 h [4] ± GM 1.7mg/kg, every 8 h	ST or " ABPC + ST"	Consult with infectious disease specialists
Nocardia spp.	Severe pneumonia / brain abscess / disseminated infection	(Routine susceptibility tests are difficult)	ST trimethoprim 240–320 mg, every 8 h + IPM/CS 0.5 g, every 6 h or IPM/CS 0.5 g, every 6 h + AMK 15 mg/kg, every 24 h [4,20]	LZD, MEPM, CTRX, MINO	Consult with infectious disease specialist. LZD is usually "S". ST is rarely "R", but room for debate regarding correlation between susceptibility tests and clinical effects. ST: trimethoprim 15 mg/kg/day equals to Japanese ST mixture (1 tablet or 1 g of trimethoprim is 80 mg) 3–4 tablets or 3–4 g, every 8 h
**Gram-negative cocci [GNC]**					
Neisseria meningitidis	Meningitis, bacteremia	PCG: S (MIC < 0.1μg/mL)	PCG 4,000,000 units, every 4 h or ABPC 2 g, every 4 h [4,15,16]	CTRX	
		PCG: R	CTRX 2g , every 12 h [4,15,16]		
**Gram-negative rods (Enterobacteriaceae) [GNR]**					
Escherichia coli, Proteus mirabilis Note: See section on *Enterobacter spp*. for *Proteus vulgaris*	Urinary tract infection, bacteremia, etc. (excluding meningitis)	ABPC: S	ABPC 1～2g, every 6 h [21]	CPFX (if S) or ST (if S)	
		ABPC: R & CEZ: S	CEZ 2g, every 8 h [4,22,23]		
		ABPC: R & CEZ: R & CTRX (CTX): S	CTRX 1～2g, every 24 h [4,23,24]		
		ESBL-producing strain CTRX (CTX): R or CAZ: R & MEPM:S & TAZ/PIPC: S & CMZ: S	CMZ 1～2g, every 8 h [25,26]<br>TAZ/PIPC 4.5g, every 6～8 h [27,28]<br>MEPM 1g, every 8 h [4,23,24]		CMZ and TAZ/PIPC can be an option for pyelonephritis.
		Either MEPM or IPM/CS are not S	Consult for infectious disease specialists.		
	Meningitis	CTRX: S	CTRX 2g, every 12 h [4,29]		Avoid CEZ for meningitis.
		CTRX: R & MEPM: S	MEPM 2g, every 8 h [29]		
		Either MEPM or IPM/CS are not S	Consult with infectious disease specialists.		

Klebsiella spp.	Urinary tract infection, pneumonia, liver abscess, etc.	・ABPC is naturally resistant.<br>・See section on "*Escherichia coli, Proteus spp*".<br>・Observational studies indicates that CTRX is superior than CEZ for invasive liver abscess [30].			
Enterobacter spp., *Citrobacter spp.*, *Serratia marcescens*, *Proteus vulgaris*, *Morganella spp.*	Bacteremia, pneumonia, etc. (excluding meningitis)	CTRX (CTX): S & CAZ: S & CFPM: S	CFPM 1g, every 8 h or 2g, every 8-12 h [4,23,24,31]<br>TAZ/PIPC 4.5g, every 6-8 h [24] or CTRX 1-2g, every 24 h [4,23,24]	MEPM or CPFX (if S) or ST (if S)	ABPC is naturally resistant. CTRX, CAZ, and TAZ/PIPC potentially become resistant during treatment due to AmpC cephalosporinase production, specifically in cholangitis associated with biliary tract malignancies [32].
		CTRX (CTX): R or CAZ: R — CFPM: S & MEPM: S	CFPM (1g, every 8 h or 2g, every 8-12 h) [4,23,24]	CPFX (if S) or ST (if S)	
		CTRX (CTX): R or CAZ: R — CFPM: R & MEPM: S	MEPM 1g, every 8 h [4,23,24]		
		Either MEPM or IPM/CS are not S	Consult with infectious disease specialists		*Serratia spp.* is naturally resistant to colistin.
	Meningitis	CFPM: S	CFPM 2g, every 8 h [29]		Consult with infectious disease specialist. CTRX can be used for *C. koseri*.
		MEPM: S	MEPM 2g, every 8 h [29,33]		
		Either MEPM or IPM/CS are not S.	Consult with infectious disease specialists.		*Serratia spp.* is naturally resistant to colistin.
Salmonella spp. (extra-abdominal typhus)	Bacteremia, extra-abdominal infections (e.g., mycotic aneurysms)	ABPC: S	ABPC 2g, every 6 h [33]	CPFX (if S)	
		ABPC: R & CTRX: S	CTRX 2g, every 24 h [33]		2 g, every 12 h for meningitis
		ABPC: R & CTRX: R & MEPM: S	MEPM 1g, every 8 h [33]		2 g, every 8 h for meningitis
**Gram-negative rods (non-glucose fermenting bacteria) [GNR]**					
Pseudomonas aeruginosa	Pneumonia, urinary tract infection, bacteremia, febrile neutropenia, etc. (excluding meningitis)	CAZ: S	CAZ 2 g, every 8 h (or 1 g, every 6 h) [4,23]	MEPM (if S) or CPFX (if S)	
		CFPM: S	CFPM 2g, every 8-12 h (or 1g, every 8 h) [4,23]		
		PIPC: S	PIPC 4g, every 6 h [4]		PIPC susceptibility standard is set when at least 3 g is used every 6 h [23].
		All of the above and R & MEPM: S	MEPM 1g, every 8 h [4,23]	CPFX (if S)	
		Either MEPM or IPM/CS are not S	CTLZ/TAZ considered		Consult with infectious disease specialists.
	Meningitis	CAZ: S or CFPM: S	CAZ 2g, every 8 h or CFPM 2g, every 8 h [10]		
		MEPM: S	MEPM 2g, every 8 h [29]		
Acinetobacter baumannii	Hospital-acquired pneumonia / ventilator-associated pneumonia,	CFPM: S	CFPM 2g, every 8 h [4]	CPFX (if S) or MINO (if S)	
		SBT/ABPC: S	SBT/ABPC 3 g, every 6 h (consult with infectious disease specialist for severe cases) [4,34]		SBT exerts antibacterial effect.
		MEPM: S	MEPM 1g, every 8 h [23]		

	wound infection	Either MEPM or IPM/CS are not S	Consult with infectious disease specialist.		
Stenotrophom-onas maltophilia	Bacteremia, pneumonia	ST: S	240–320 mg, every 8 h as ST trimethoprim [4]	MINO [4] or CPFX (if S)	Naturally resistant to carbapenem. ST: trimethoprim 15 mg/kg/day equals to Japanese ST mixture (1 tablet or 1 g of trimethoprim is 80 mg) 3–4 tablets or 3–4 g, every 8 h
**Gram-negative rods (others) [GNR]**					
Haemophilus influenzae	Meningitis	ABPC: S	ABPC 2g, every 4 h [14-16]	CTRX [29]	
		ABPC: R & CTRX (CTX): S	CTRX 2g, every 12 h [4,15,16]	CFPM [29]	
	Pneumonia, epiglottitis	ABPC: S	ABPC 2g, every 6 h [4]		
		ABPC: R & SBT/ABPC: S	SBT/ABPC 3g, every 6 h [4]		
		ABPC: R & CTRX (CTX): S	CTRX 1-2g, every 24 h [4]		
Pasteurella multocida, Capnocytopha-ga canimorsus	Animal bite	PCG: S	SBT/ABPC 3g, every 6 h [17]	CTRX	PCG 4,000,000 units every 4 h for infections can be used for monobacterial infeciton.
		PCG: R & SBT/ABPC: S	SBT/ABPC 3g, every 6 h [17]	CTRX	
Aeromonas spp.	Soft tissue infection, bacteremia	CTRX: S or MINO: S	CTRX 2g, every 24 h＋MINO 100mg, every 12 h [17]	CPFX＋MINO, LVFX	
Vibrio vulnificus	Soft tissue infection, bacteremia	CTRX: S & MINO: S	CTRX 2g, every 24 h ＋MINO 100mg, every 12 h [17]	CTX＋CPFX, LVFX	Observational studies have indicated that β-lactam monotherapy had a higher mortality rate than combination therapy [35].
**Obligate anaerobic bacteria (other than *C. difficile*)**					
Obligate anaerobes	Polymicrobial infections	・Indication for antimicrobials against obligate anaerobes is considered depending on the efficacy of drainage.<br>・Indication for antimicrobials against obligate anaerobes for polymicrobial infections are determined by the susceptibility results.<br>・Obligate anaerobes have the three following characteristics depending on the susceptibility pattern.<br>(1) Most obligate anaerobes existing above the diaphragm (e.g., *Peptostreptococcus spp., Prevotella spp.*) are susceptibile to PCG and CLDM, while β-lactamase-producing strain exists.<br>(2) Obligate anaerobic bacteria existing below the diaphragm (e.g., *Bacteroides spp.*) include β-lactamase-producing strains. The resistance rates of non-fragilis *Bacteroides spp.* (other than *B. fragilis*) against CLDM and CMZ have been increasing.<br>(3) Most obligate anaerobes which include (1) and (2) are susceptibile to SBT/ABPC, TAZ/PIPC, MEPM, and MNZ.<br>・The two following points should be considered when selecting a target antimicrobials for polymicrobial infections where obligate anaerobes contribute: (1) Whether obligate anaerobic bacteria shuold truly be covered, and (2) Whether bacteria other than obligate anaerobic bacteria should be covered.			

Peptostreptococcus spp., Prevotella spp. (obligate anerobes above the diaphragm)	Lung abscess, deep cervical infection, etc.	Susceptibility results of bacteria other than obligate anaerobes should be referred.		SBT/ABPC 3g, every 6 h or CLDM 600mg, every 8 h or MNZ 500mg, every 8 h ＋PCG 2,000,000–3,000,000 units, every 4 h or CTRX 2g, every 24 h [36]	TAZ/PIPC	
	Brain abscess			PCG 4,000,000 units, every 4 h or CTRX 2g, every 12 h or CFPM 2g, every 8h ＋ MNZ 500mg, every 8 h[37]		
Bacteroides spp. (obligate anerobes below the diaphragm)	Polymicrobial intra- abdominal infection (secondary peritonitis, intraperitoneal abscess, cholangitis)	Insufficient drainage	Susceptibility results of bacteria other than obligate anaerobes should be referred.	SBT/ABPC 3g, every 8 h or TAZ/PIPC 4.5g, every 8 h or MNZ 500mg, every 8 h + CEZ 2g, every 8 h or CTRX 2g, every 24 h or CFPM 2g, every 12 h or CPFX 400mg, every 12h [4]	MEPM	CMZ: R and CLDM: "resistant" are increasing [4].
		Sufficient drainage		CMZ 1g, every 8 h or CLDM 600mg, every 8 h + CEZ 2g, every 8 h or CTRX 2g, every 24 h or CFPM 2g, every 12 h or CPFX 400mg, every 12 h or aforementioned “insufficient drainage” options. [4]		
Clostridium spp. (e.g., *C. perfringens*)	Gas gangrene	PCG: S		PCG 4,000,000 units, every 4 h +CLDM 600 mg, every 8 h [4,17]		CLDM is used for toxin production suppression purposes even when susceptibility is "resistant" [4].
Clostridioides (Clostridium) difficile						
Clostridioides (Clostridium) difficile	*Clostridioides difficile* infection (CDI)	Initial onset		VCM 125 mg, four times a day (orally or through nasogastric tube) or FDX 200mg, twice a day[4,38]	Non-severe: MNZ orally	Intravenous VCM is ineffective.
		Reccurrence		FDX 200mg, twice a day[38]	When initial treatment is MNZ: VCM	
		Shock, hypotension, megacolon, ileus, or VCM 125 mg regimen is ineffective		VCM 500 mg, every 6 h (orally or through nasogastric tube) (500 mg / saline 100 mL as stationary enema through anus for ileus) ± MNZ 500 mg, intravenously every 8 h[38]		
**Other bacteria**						
Legionella spp.	Pneumonia			LVFX 500～750mg, every 24 h [4]or AZM 500mg, every 24 h [4]	MINO [4]	
Mycoplasma pneumoniae	Pneumonia			MINO 100mg, every 12 h [4]	AZM or LVFX	
Rickettsia japonica	Japanese spotted fever			MINO 100mg, every 12 h [39]	CPFX	
Orientia tsutsugamushi	Scrub typhus			MINO 100mg, every 12 h [39]	AZM	CPFX is ineffective.

Leptospira interrogans	Leptospirosis		PCG 1,500,000 units, every 6 h [40]	CTRX or MINO	
**Fungi**					
Candida spp.	Candidemia, disseminated candidiasis (includes febrile neutropenia)	・Empirical treatment should be stepped down to oral FLCZ or VRCZ if blood culture is negative and clinically stable.<br>・Switch to FLCZ or VRCZ in endophthalmitis since MCFG has poor intraocular penetration (L-AMB ± 5-FC if there is resistance to FLCZ and VRCZ).<br>・Most of *C. albicans, C. parapsilosis,* and C. *tropicalis* are susceptible to FLCZ, *C. glabrata* is either susceptible or resistant, and *C. krusei* is naturally resistant. The difficult-to-identify *C. auris* that can be multi-drug resistant has been recently reported.<br>・Most cases of candiduria are not treated. However, candidemia and disseminated candidiasis may be diagnosed as a result of candiduria. Infectious disease specialists should also be consulted when candiduria requires treatment. MCFG and L-AMB have poor urinary tract penetration.			
Candida albicans, C. parapsilosis, C. tropicalis	After stabilization of candidemia	FLCZ: S	FLCZ initial dose 800 mg and subsequent doses 400 mg, every 24 h [41]		
C. glabrata		FLCZ: S	FLCZ initial dose 800 mg and subsequent doses 400 mg, every 24 h [41]		MCFG can be an alternative. Consult with infectious disease specialists
		FLCZ: R & VRCZ: S	VRCZ initial dose 6 mg/kg, every 12 h and subsequent doses 4 mg/kg, every 12 h[41]		
C. krusei		FLCZ: R & VRCZ: S	VRCZ initial dose 6 mg/kg, every 12 h and subsequent doses 4 mg/kg, every 12 h [41]		
Aspergillus spp.	Invasive pulmonary aspergillosis		VRCZ initial dose 6 mg/kg, every 12 h and subsequent doses 4 mg/kg, every 12 h[4,41]	L-AMB [4]	
Pneumocystis jirovecii	Pneumocystis		240–320 mg as ST trimethoprim, every 8 h [4]	Intravenous infusion of pentamidine [4]	ST: trimethoprim 15 mg/kg/day equals to Japanese ST mixture (1 tablet or 1 g of trimethoprim is 80 mg) 3–4 tablets or 3–4 g, every 8 h.
Cryptococcus spp.	Meningitis (non-HIV)		L-AMB, 3–4 mg/kg, every 24 h + 5-FC, 25 mg/kg orally, every 6 h [41]	FLCZ (high dose)	
Mucor spp .	Mucormycosis		L-AMB, 5～10mg/kg, every 24 h [41]		
**Virus**					
Influenza	Pneumonia, etc.		Oseltamivir 75 mg orally, twice a day [42]	Peramivir	

SFTS	Severe fever with thrombocytopenia syndrome		Not validated [43]		Consider tetracycline when rickettsial infection cannot be ruled out by medical history. [43]
CMV	Pneumonia, etc.		Ganciclovir 5 mg/kg, every 12 h [4]	Foscarnet	
HSV	Pneumonia, etc.		Acyclovir 10 mg/kg, every 8 h [44]		
SARS-CoV2	Pneumonia, etc.		See other guidelines for details [45,46]		

This table refers to guidelines relating to each infectious disease and the JAID/JSC infectious disease treatment guidelines and adds susceptibility test criteria [23] and information regarding proper use of antimicrobial agents [13].

ABPC, ampicillin; AMK, amikacin; AZM, azithromycin; CAZ, ceftazidime; CEZ, cefazolin; CFPM, cefepime; CLDM, clindamycin; CMV, cytomegalovirus; CMZ, cefmetazole; CPFX, ciprofloxacin; CTRX, ceftriaxone; CTX, cefotaxime; DAP, daptomycin; 5-FC, flucytosine; FDX, fidaxomicin; FLCZ, fluconazole; GM, gentamycin; HSV, herpes simplex virus; IPM/CS, imipenem/cilastatin; L-AMB, liposomal amphotericin B; LVFX, levofloxacin; LZD, linezolid; MCFG, micafungin; MEPM, meropenem; MINO, minocycline; MNZ, metronidazole; PCG, penicillin G; PIPC, piperacillin; RFP, rifampicin; SBT/ABPC, sulbactam/ampicillin; SFTS, severe fever with thrombocytopenia syndrome; ST, sulfamethoxazole/trimethoprim; TAZ/PIPC, tazobactam/piperacillin; TEIC, teicoplanin; VCM, vancomycin; VRCZ, voriconazole. (Abbreviations of antimicrobials are based on JAID/JSC infectious disease treatment guidelines)

S: susceptible, I: intermediarte, R: resistant

1) Fraimow HS. Systemic Antimicrobial Therapy in Osteomyelitis. Semin Plast Surg. 2009; 23: 90-9.

2) Bille J. Medical treatment of staphylococcal infective endocarditis. Eur Heart J. 1995; 16: 80-3.

3) Infectious Disease and Antimicrobial Agents: Penicillins. Available online at: www.antimicrobe.org/d24.asp Accessed August 20, 2023.

4) JAID/JSC infectious disease treatment guideline 2019. Japanese Association for Infectious Diseases / Japanese Society of Chemotherapy. 2023.

5) Nakatani S, Ohara T, Ashihara K, et al. JCS 2017 Guideline on Prevention and Treatment of Infective Endocarditis. Circ J. 2019;83:1767-809.

6) Habib G, Lancellotti P, Antunes MJ, et al. 2015 ESC Guidelines for the management of infective endocarditis: The task force for the management of infective endocarditis of the European society of cardiology (ESC): Endorsed by: European association for cardio-thoracic surgery (EACTS), the European association of nuclear medicine (EANM). Russ J Cardiol. 2016; 133: 65-116.

7) Oda S, Aibiki M, Ikeda T, et al. The Japanese guidelines for the management of sepsis. J Intensive Care. 2014;2:55.

8) Antibacterial TDM Guidelines 2022. Japanese Society of Chemotherapy. https://www.chemotherapy.or.jp/uploads/ffile/guideline/tdm2022.pdf

9) MRSA Infection Treatment Guideline - Revised Edition - 2019. Japanese Society of Chemotherapy / Japanese Association for Infectious Diseases.MRSA Infection Treatment Guideline Creation Committee. (2019).

10) Imaeda T, Nakada Ta, Takahashi N, et al. Trends in the incidence and outcome of sepsis using data from a Japanese nationwide medical claims database-the Japan Sepsis Alliance (JaSA) study group- Crit Care Med. 2021; 25: 338

11) Gould FK, Denning DW, Elliott TSJ, et al: Guidelines for the diagnosis and antibiotic treatment of endocarditis in adults: A report of the working party of the british society for antimicrobial chemotherapy. J Antimicrob Chemother. 2012; 67: 269-89.

12) Lappin E, Ferguson AJ. Gram-positive toxic shock syndromes.Lancet Infect Dis. 2009; 9: 281-90.

13) Guidance for implementing an antimicrobial stewardship programs in Japan. 2017. https://doi.org/10.11150/kansenshogakuzasshi.91.709

14) Practical Guideline for Bacterial Meningitis 2014. Japanese Society of Neurology. Nankodo, Tokyo. 2014.

15) Hasbun R. Progress and Challenges in Bacterial Meningitis: A Review JAMA. 2022; 328: 2147-2154.

16) Tunkel AR, Hartman BJ, Kaplan SL, et al. Practice Guidelines for the Management of Bacterial Meningitis. Clin Infect Dis. 2004; 39: 1267-84.

17) Stevens DL, Bisno AL, Chambers HF, et al. Practice Guidelines for the Diagnosis and Management of Skin and Soft Tissue Infections: 2014 Update by the Infectious Diseases Society of America. Clin Infect Dis. 2014; 59: e10-52.
18) Han SB, Bae EY, Lee JW, et al. Clinical characteristics and antimicrobial susceptibilities of viridans streptococcal bacteremia during febrile neutropenia in patients with hematologic malignancies: a comparison between adults and children. BMC InfectDis. 2013; 13: 273.
19) Nigo M, Munita JM, Arias CA, et al. What's new in the treatment of enterococcal endocarditis?. Curr Infect Dis Rep. 2014; 16: 431.
20) Restrepo A, Clark NM, Infectious Diseases Community of Practice of the American Society of Transplantation: Nocardia infections in solid organ transplantation: Guidelines from the Infectious Diseases Community of Practice of the American Society of Transplantation. Clin Transplant. 2019; 33: e13509.
21) NICE: Pyelonephritis (acute) antimicrobial prescribing guideline Evidence review. NICE guideline 111. 2018.
22) Wang KC, Liu MF, Lin CF, et al. The impact of revised CLSI cefazolin breakpoints on the clinical outcomes of Escherichia coli bacteremia. J Microbiol Immunol Infect. 2016; 49: 768-74.
23) Patel JB, Clinical and Laboratory Standards Institute: Performance standards for antimicrobial susceptibility testing. CLSI. M100; S27.
24) European Association of Urology. EAU Guidelines on Urological Infections. 2018.
25) Matsumura Y, Yamamoto M, Nagao M, et al. Multicenter Retrospective Study of Cefmetazole and Flomoxef for Treatment of Extended-Spectrum-β-Lactamase-Producing Escherichia Coli Bacteremia. Antimicrob Agents Chemother. 2015; 59: 5107-13.
26) Fukuchi T, Iwata K, Kobayashi S, et al. Cefmetazole for bacteremia caused by ESBL-producing enterobacteriaceae comparing with carbapenems BMC Infect Dis. 2016; 16: 427.
27) Harris PNA, Tambyah PA, Paterson DL. β-lactam and β-lactamase inhibitor combinations in the treatment of extended spectrum β-lactamase producing Enterobacteriaceae: Time for a reappraisal in the era of few antibiotic options? Lancet Infect Dis. 2015; 15: 475-85.
28) Harris PNA, Tambyah PA, Lye DC, et al. Effect of piperacillintazobactam vs meropenem on 30-day mortality for patients with e coli or Klebsiella pneumoniae bloodstream infection and ceftriaxone resistance. JAMA. 2018; 320: 984-94.
29) Tunkel AR, Hasbun R, Bhimraj A, et al. 2017 Infectious Diseases Society of America's Clinical Practice Guidelines for HealthcareAssociated Ventriculitis and Meningitis. Clin Infect Dis. 2017; 64: e34
30) Cheng HP, Siu LK, Chang FY. Extended-spectrum cephalosporin compared to cefazolin for treatment of Klebsiella pneumoniae caused liver abscess. Antimicrob Agents Chemother. 2003; 47: 2088-92.
31) Tamma PD, Girdwood SCT, Gopaul R, et al. The use of cefepime for treating AmpCβ-lactamase-producing enterobacteriaceae. Clin Infect Dis. 2013; 57: 781-8.
32) Choi SH, Lee JE, Park SJ, et al. Emergence of antibiotic resistance during therapy for infections caused by Enterobacteriaceae producing AmpCβ-lactamase: Implications for antibiotic use. Antimicrob Agents Chemother. 2008; 52: 995-1000.
33) Pegues DV, Miller SI. Salmonella Species. Mandell, Douglas, and Bennett's Principles and Practice of Infectious Diseases. Elsevier Inc., Amsterdam, 2019.
34) Chu H, Zhao L, Wang M, et al. Sulbactam-based therapy for Acinetobacter baumannii infection: A systematic review and meta-analysis. Braz J Infect Dis. 2013; 17: 389-94.
35) Wong KC, Brown AM, Luscombe GM, et al. Antibiotic use for Vibrio infections: important insights from surveillance data. BMC Infect Dis. 2015; 15: 226.
36) Desai H, Agrawal A. Pulmonary emergencies: Pneumonia, acute respiratory distress syndrome, lung abscess, and empyema. Med Clin North Am. 2012; 96: 1127-48.
37) Brouwer MC, Tunkel AR, McKhann GM, et al. N Engl J Med. 2014; 371: 447-56.
38) Japanese Chemotherapy Society / Japanese Association for Infectious Disease CDI Treatment Guideline Creation Committee. Clostridioides（Clostridium）difficile Infection Treatment Guideline. 20[illegible]
39) Sando E. Rickettsial infection. Hospitalist. 2017; 5: 519-28.
40) Rajapakse S. Leptospirosis: clinical aspects. Clin Med (Lond). 2022; 22: 14-17.
41) Japanese Society for Medical Mycology. Invasive Candidiasis Diagnostic and Treatment Guidelines. 2013.
42) IDSA. Clinical Practice Guidelines by the Infectious Diseases Society of America: 2018 Update on Diagnosis, Treatment, Chemoprophylaxis, and Institutional Outbreak Management of Seasonal Influenza. Clin Infect Dis. 2019; 30: 97-8.
43) Kato Y, et al. Severe Fever with Thrombocytopenia Syndrome (SFTS) Treatment Procedures Revised Edition (4th Ed.). National Center for Global Health and Medicine, Tokyo. 2019.
44) Practical Guideline for Herpes Simplex Encephalitis 2017.Japanese Society of Neurology. Nankodo, Tokyo. 2014.
45) Special Committee of the Japanese Clinical Practice Guidelines for the Management of Sepsis and Septic Shock 2020 (J-SSCG 2020), the COVID-19 Task Force. Japanese rapid/living recommendations on drug management for COVID-19: updated guidelines (July 2022). Acute Med Surg. 2022; 9(1): e789.

46) Japanese Association for Infectious Disease COVID-19 Treatment Guideline Creation Task Forse. Clinical manual for COVID-19 (version 10.1, in Japanese). https://www.mhlw.go.jp/content/001248424.pdf
