# Supplementary material for: The Japanese Clinical Practice Guidelines for Management of Sepsis and Septic Shock 2024
Source: J Intensive Care. 2025 Mar 14;13:15. doi: 10.1186/s40560-025-00776-0 (PMC11907869; doi:10.1186/s40560-025-00776-0)
Supplement: Supplementary file 3 — Additional file 3 [file 40560_2025_776_MOESM3_ESM.pdf]

Supplementary Online Content
The Japanese Clinical Practice Guidelines for Management of Sepsis and Septic Shock 2024

Evidence profile and summary of judgements

CQ2-1: Is Gram stain testing useful for selecting empiric antimicrobials for sepsis?
CQ2-2: Is the administration of empiric antimicrobials for sepsis started within 1 h after diagnosing sepsis?
CQ2-7: Is continuous or extended infusion of antimicrobials used for sepsis? (β-lactams)
CQ2-7: Is continuous or extended infusion of antimicrobials used for sepsis? (Glycopeptides)
CQ2-8: Is antimicrobial dosage adjusted using therapeutic drug monitoring (TDM) for sepsis?
CQ2-9: Is de-escalation based on culture and susceptibility results performed in antimicrobial therapy for sepsis?
CQ2-10: In patients with sepsis receiving empiric antifungal drugs, are antifungal drugs discontinued using β-D glucan as an indicator?
CQ2-11: Is procalcitonin (PCT) used as an indicator for discontinuing antimicrobial therapy for sepsis?
CQ2-12: Is short-term (≤ 7 days) antimicrobial therapy used for sepsis?
CQ3-3: What is the target mean arterial pressure (MAP) during initial resuscitation for sepsis?
CQ3-4: Which fluid is used for initial resuscitation of sepsis? (Balanced crystalloids)
CQ3-4: Which fluid is used for initial resuscitation of sepsis? (Isotonic albumin solutions 4–5%)
CQ3-4: Which fluid is used for initial resuscitation of sepsis? (Synthetic colloids)
CQ3-6: Is early administration of vasopressor performed during initial resuscitation for sepsis?
CQ3-7: Which vasopressor is used as the first-line drug in patients with septic shock? (Noradrenaline)
CQ3-7: Which vasopressor is used as the second-line drug in patients with septic shock? (Noradrenaline + vasopressin)
CQ3-8: Are steroids administered for septic shock?
CQ3-9: What is the threshold of hemoglobin level for transfusion in initial resuscitation for septic shock?
CQ3-10: Are β1-receptor blockers used for septic patients with persistent tachycardia after initial resuscitation?
CQ3-11: Is sodium bicarbonate intravenously administered for septic patients with severe metabolic acidosis (pH ≤ 7.2)?
CQ3-13: Is restrictive fluid management provided in septic patients with stable hemodynamics?
CQ4-1: Is polymyxin B-immobilized fiber column (PMX-DHP) used for patients with septic shock?
CQ4-2: Is early renal replacement therapy (RRT) performed for septic AKI?
CQ4-3: Is continuous treatment provided in RRT for septic AKI?
CQ4-4: Is treatment dose increased in RRT for septic AKI?
CQ5-3: Is antithrombin administered for sepsis-induced DIC?
CQ5-4: Is recombinant thrombomodulin administered for sepsis-induced DIC?
CQ6-1: Is intravenous immunoglobulin (IVIG) administered for sepsis?
CQ6-2: Is high-dose vitamin C therapy used for sepsis?
CQ6-3: What is the target blood glucose level for sepsis?
CQ6-4: Is antipyretic therapy provided to febrile patients with sepsis?
CQ6-5: Is stress ulcer prophylaxis performed for patients with sepsis to prevent gastrointestinal hemorrhage?

CQ7-1: Is early rehabilitation implemented to prevent PICS?
CQ7-2: Is neuromuscular electrical stimulation used to prevent ICU-acquired weakness (ICU-AW)?
CQ7-3: Is follow up after ICU discharge be implemented to improve physical, cognitive, and mental functions?
CQ7-4: Is rehabilitation after hospital discharge implemented to improve physical, cognitive, and mental functions?
CQ 8-1: Is written information provided to families of critically ill patients?
CQ 8-4: Is an ICU diary kept for critically ill patients?
CQ 8-5: Is follow-up after ICU discharge provided to families of critically ill patients to improve their mental health?
CQ9-5: Are steroids administered to pediatric patients with septic shock who are unresponsive to initial fluid therapy and vasopressors?
CQ9-6: What is the optimal hemoglobin level for blood transfusion in pediatric patients with sepsis who have stable hemodynamics?
CQ9-7: Is strict blood glucose control performed for pediatric sepsis?

CQ2-1: Is Gram stain testing useful for selecting empiric antimicrobials for sepsis?

P	Sepsis / septic shock or critically ill patients with infection
I	Gram staining-based antimicrobial therapy
C	Usual care
O	28-day mortality, clinical cure, appropriate antimicrobial selection, use of anti-MRSA agents, use of anti-pseudomonal agents

Evidence profile

Certainty assessment							Number of patients		Effect		Certainty	Importance
№ of studies	Study design	Risk of bias	Inconsistency	Indirectness	Imprecision	Other considerations	Intervention	Control	Relative (95% CI)	Absolute (95% CI)		
28-day mortality												
1	randomized trials	not serious	not serious	not serious	very serious	none	14/103 (13.6%)	18/103 (17.5%)	RR 0.78 (0.41 to 1.48)	38 fewer per 1000 (from 103 fewer to 84 more)	⊕⊕◯◯ Low	Critical
Clinical cure												
1	randomized trials	not serious	not serious	not serious	very serious	none	79/103 (76.7%)	74/103 (71.8%)	RR 1.07 (0.91 to 1.25)	50 more per 1000 (from 65 fewer to 180 more)	⊕⊕◯◯ Low	Critical
Appropriate antimicrobial selection												
1	randomized trials	not serious	not serious	not serious	serious	none	89/103 (86.4%)	95/103 (92.2%)	RR 0.94 (0.85 to 1.03)	55 fewer per 1000 (from 138 fewer to 28 more)	⊕⊕⊕◯ Moderate	Critical
Use of anti-MRSA agents												
1	randomized trials	not serious	not serious	not serious	serious	none	63/103 (61.2%)	103/103 (100%)	RR 0.61 (0.53 to 0.72)	390 more per 1000 (from 470 fewer to 280 fewer)	⊕⊕⊕◯ Moderate	Critical
Use of anti-pseudomonal agents												
1	randomized trials	not serious	not serious	not serious	serious	none	72/103 (69.9%)	103/103 (100%)	RR 0.70 (0.62 to 0.80)	300 fewer per 1000 (from 380 fewer to 200 fewer)	⊕⊕⊕◯ Moderate	Critical

CI: confidence interval; MRSA, Methicillin-resistant *Staphylococcus aureus*; RR: risk ratio

Summary of Judgements

	JUDGEMENT						
**Problem**	No	Probably no	Probably yes	**Yes**		Varies	Don't know
**Desirable Effects**	Trivial	Small	**Moderate**	Large		Varies	Don't know
**Undesirable Effects**	Large	Moderate	Small	**Trivial**		Varies	Don't know
**Certainty of evidence**	Very low	**Low**	Moderate	High			No included studies
**Values**	Important uncertainty or variability	**Possibly important uncertainty or variability**	Possibly no important uncertainty or variability	No important uncertainty or variability			
**Balance of effects**	Favors the comparison	Probably favors the comparison	Does not favor either the intervention or the comparison	Probably favors the intervention	**Favors the intervention**	Varies	Don't know
**Cost effectiveness**	Favors the comparison	Probably favors the comparison	Does not favor either the intervention or the comparison	Probably favors the intervention	Favors the intervention	Varies	**No included studies**
**Resources required**	Large costs	Moderate costs	**Negligible costs and savings**	Moderate savings	Large savings	Varies	Don't know
**Acceptability**	No	Probably no	**Probably yes**	Yes		Varies	Don't know
**Feasibility**	No	Probably no	Probably yes	Yes		**Varies**	Don't know

CQ2-2: Is the administration of empiric antimicrobials for sepsis started within 1 h after diagnosing sepsis?

P	Sepsis / septic shock
I	empiric antimicrobial administration within 1 h from diagnosing sepsis
C	empiric antimicrobial administration after 1 h from diagnosing sepsis
O	Hospital mortality, 28 or 30-day mortality

Evidence profile

Certainty assessment							Number of patients		Effect		Certainty	Importance
№ of studies	Study design	Risk of bias	Inconsistency	Indirectness	Imprecision	Other considerations	Intervention	Control	Relative (95% CI)	Absolute (95% CI)		
Hospital mortality												
11	non-randomized studies	very serious	serious	not serious	not serious	residual confounding*	2989/10226 (29.2%)	7065/22445 (31.5%)	RR 0.93 (0.82 to 1.05)	22 fewer per 1,000 (57 fewer to 16 more)	⊕⊕◯◯ Low	Critical
28 or 30-day mortality												
5	non-randomized studies	very serious	serious	not serious	serious	none	482/1816 (26.5%)	1396/5058 (27.6%)	RR 1.07 (0.82 to 1.40)	19 more per 1,000 (50 fewer to 110 more)	⊕◯◯◯ Very low	Critical

*All plausible residual confounding would reduce the demonstrated effect.

CI: confidence interval; RR: risk ratio

Summary of Judgements

	JUDGEMENT						
Problem	No	Probably no	Probably yes	**Yes**		Varies	Don't know
Desirable Effects	**Trivial**	Small	Moderate	Large		Varies	Don't know
Undesirable Effects	Large	Moderate	Small	Trivial		Varies	**Don't know**
Certainty of evidence	Very low	**Low**	Moderate	High			No included studies
Values	Important uncertainty or variability	Possibly important uncertainty or variability	**Possibly no important uncertainty or variability**	No important uncertainty or variability			
Balance of effects	Favors the comparison	Probably favors the comparison	**Does not favor either the intervention or the comparison**	Probably favors the intervention	Favors the intervention	Varies	Don't know
Cost effectiveness	Favors the comparison	Probably favors the comparison	Does not favor either the intervention or the comparison	Probably favors the intervention	Favors the intervention	Varies	**No included studies**
Resources required	Large costs	Moderate costs	**Negligible costs and savings**	Moderate savings	Large savings	Varies	Don't know
Acceptability	No	**Probably no**	Probably yes	Yes		Varies	Don't know
Feasibility	No	**Probably no**	Probably yes	Yes		Varies	Don't know

CQ2-7: Is continuous or extended infusion of antimicrobials used for sepsis? (β-lactams)

P	Sepsis
I	Continuous or extended infusion of antimicrobials
C	usual care
O	Mortality, clinical cure, adverse events, occurrence of antibiotic-resistant bacteria

Evidence profile

Certainty assessment							Number of patients		Effect		Certainty	Importance
№ of studies	Study design	Risk of bias	Inconsistency	Indirectness	Imprecision	Other considerations	Intervention	Control	Relative (95% CI)	Absolute (95% CI)		
Mortality												
14	randomized trials	not serious	not serious	not serious	serious	none	243/848 (28.7%)	1282/855 (33.0%)	RR 0.84 (0.71 to 1.00)	53 fewer per 1,000 (from 96 fewer to 0 fewer)	⊕⊕⊕◯ Moderate	Critical
Clinical cure												
11	randomized trials	serious	not serious	not serious	not serious	publication bias strongly suspected	293/539 (54.4%)	247/542 (45.6%)	RR 1.24 (1.04 to 1.47)	109 more per 1,000 (from 18 more to 214 more)	⊕⊕◯◯ Low	Critical
Adverse events												
5	randomized trials	serious	not serious	not serious	serious	none	48/685 (7.0%)	50/697 (7.2%)	RR 0.99 (0.68 to 1.43)	1 fewer per 1,000 (from 23 fewer to 31 more)	⊕⊕◯◯ Low	Critical
Occurrence of antibiotic-resistant bacteria												
2	randomized trials	not serious	not serious	not serious	serious	none	70/38 ((18.2%)	74/382 (19.4%)	RR 0.93 (0.70 to 1.24)	14 fewer per 1,000 (from 58 fewer to 46 more)	⊕⊕⊕◯ Moderate	Critical

CI: confidence interval; RR: risk ratio

Summary of Judgements

	JUDGEMENT						
**Problem**	No	Probably no	Probably yes	**Yes**		Varies	Don't know
**Desirable Effects**	Trivial	Small	Moderate	**Large**		Varies	Don't know
**Undesirable Effects**	Large	Moderate	Small	**Trivial**		Varies	Don't know
**Certainty of evidence**	Very low	Low	**Moderate**	High			No included studies
**Values**	Important uncertainty or variability	Possibly important uncertainty or variability	**Possibly no important uncertainty or variability**	No important uncertainty or variability			
**Balance of effects**	Favors the comparison	Probably favors the comparison	Does not favor either the intervention or the comparison	Probably favors the intervention	**Favors the intervention**	Varies	Don't know
**Cost effectiveness**	Favors the comparison	Probably favors the comparison	Does not favor either the intervention or the comparison	**Probably favors the intervention**	Favors the intervention	Varies	No included studies
**Resources required**	Large costs	Moderate costs	**Negligible costs and savings**	Moderate savings	Large savings	Varies	Don't know
**Acceptability**	No	Probably no	**Probably yes**	Yes		Varies	Don't know
**Feasibility**	No	Probably no	**Probably yes**	Yes		Varies	Don't know

CQ2-7: Is continuous or extended infusion of antimicrobials used for sepsis? (Glycopeptides)

P	Sepsis
I	Continuous or extended infusion of antimicrobials
C	usual care
O	Mortality, clinical cure, adverse events

Evidence profile

Certainty assessment							Number of patients		Effect		Certainty	Importance
№ of studies	Study design	Risk of bias	Inconsistency	Indirectness	Imprecision	Other considerations	Intervention	Control	Relative (95% CI)	Absolute (95% CI)		
Mortality												
1	randomized trials	very serious	not serious	not serious	very serious	none	21/61 (34.4%)	19/58 (32.8%)	RR 1.05 (0.63 to 1.74)	16 more per 1,000 (from 121 fewer to 242 more)	⊕◯◯◯ Very low	Critical
Clinical cure												
1	randomized trials	serious	not serious	not serious	serious	none	48/61 (78.7%)	47/58 (81.0%)	RR 0.97 (0.81 to 1.16)	24 fewer per 1,000 (from 154 fewer to 130 more)	⊕⊕◯◯ Low	Critical
Adverse events												
3	randomized trials	very serious	not serious	not serious	very serious	publication bias strongly suspected	11/109 (10.1%)	17/105 (16.2%)	RR 0.70 (0.34 to 1.42)	49 fewer per 1,000 (from 107 fewer to 68 more)	⊕◯◯◯ Very low	Critical

CI: confidence interval; RR: risk ratio

Summary of Judgements

	JUDGEMENT						
**Problem**	No	Probably no	Probably yes	**Yes**		Varies	Don't know
**Desirable Effects**	**Trivial**	Small	Moderate	Large		Varies	Don't know
**Undesirable Effects**	Large	Moderate	Small	Trivial		Varies	**Don't know**
**Certainty of evidence**	Very low	**Low**	Moderate	High			No included studies
**Values**	Important uncertainty or variability	Possibly important uncertainty or variability	**Possibly no important uncertainty or variability**	No important uncertainty or variability			
**Balance of effects**	Favors the comparison	**Probably favors the comparison**	Does not favor either the intervention or the comparison	Probably favors the intervention	Favors the intervention	Varies	Don't know
**Cost effectiveness**	Favors the comparison	Probably favors the comparison	**Does not favor either the intervention or the comparison**	Probably favors the intervention	Favors the intervention	Varies	No included studies
**Resources required**	Large costs	Moderate costs	**Negligible costs and savings**	Moderate savings	Large savings	Varies	Don't know
**Acceptability**	No	Probably no	**Probably yes**	Yes		Varies	Don't know
**Feasibility**	No	Probably no	**Probably yes**	Yes		Varies	Don't know

CQ2-8: Is antimicrobial dosage adjusted using therapeutic drug monitoring (TDM) for sepsis?

P	Sepsis / septic shock
I	Antimicrobial therapy using therapeutic drug monitoring
C	Usual care
O	28-day mortality, clinical cure

Evidence profile

Certainty assessment							Number of patients		Effect		Certainty	Importance
№ of studies	Study design	Risk of bias	Inconsistency	Indirectness	Imprecision	Other considerations	Intervention	Control	Relative (95% CI)	Absolute (95% CI)		
28-day mortality												
5	randomized trials	not serious	not serious	not serious	serious	none	137/510 (26.9%)	142/501 (28.3%)	RR 0.94 (0.77 to 1.14)	17 fewer per 1,000 (from 65 fewer to 42 more)	⊕⊕⊕◯ Moderate	Critical
Clinical cure												
3	randomized trials	not serious	serious	serious	very serious	none	72/123 (58.5%)	54/127(42.5%)	RR 1.23 (0.91 to 1.67)	98 more per 1,000 (from 38 fewer to 285 more	⊕◯◯◯ Very low	Critical

CI: confidence interval; RR: risk ratio

Summary of Judgements

	JUDGEMENT						
**Problem**	No	Probably no	Probably yes	**Yes**		Varies	Don't know
**Desirable Effects**	Trivial	Small	**Moderate**	Large		Varies	Don't know
**Undesirable Effects**	Large	Moderate	Small	**Trivial**		Varies	Don't know
**Certainty of evidence**	**Very low**	Low	Moderate	High			No included studies
**Values**	Important uncertainty or variability	Possibly important uncertainty or variability	**Possibly no important uncertainty or variability**	No important uncertainty or variability			
**Balance of effects**	Favors the comparison	Probably favors the comparison	Does not favor either the intervention or the comparison	**Probably favors the intervention**	Favors the intervention	Varies	Don't know
**Cost effectiveness**	Favors the comparison	Probably favors the comparison	Does not favor either the intervention or the comparison	Probably favors the intervention	Favors the intervention	Varies	**No included studies**
**Resources required**	Large costs	**Moderate costs**	Negligible costs and savings	Moderate savings	Large savings	Varies	Don't know
**Acceptability**	No	Probably no	**Probably yes**	Yes		Varies	Don't know
**Feasibility**	No	Probably no	Probably yes	Yes		**Varies**	Don't know

CQ2-9: Is de-escalation based on culture and susceptibility results performed in antimicrobial therapy for sepsis?

P	Sepsis / septic shock
I	De-escalation therapy based on culture and susceptibility
C	Usual care
O	Mortality, occurrence of superinfection

Evidence profile

Certainty assessment							Number of patients		Effect		Certainty	Importance
№ of studies	Study design	Risk of bias	Inconsistency	Indirectness	Imprecision	Other considerations	Intervention	Control	Relative (95% CI)	Absolute (95% CI)		
Mortality												
1	randomized trials	serious	not serious	not serious	very serious	none	18/59 (30.5%)	13/57 (22.8%)	RR 1.34 (0.72 to 2.47)	78 more per 1,000 (from 64 fewer to 335 more)	⊕◯◯◯ Very low	Critical
Mortality												
17	non-randomized studies	serious	not serious	not serious	not serious	none	288/1711 (16.8%)	656/2663 (24.6%)	RR 0.56 (0.44 to 0.71)	92 fewer per 1,000 (from 121 fewer to 58 fewer)	⊕⊕◯◯ Low	Critical
Occurrence of superinfection												
1	randomized trials	serious	not serious	not serious	serious	none	16/59 (27.1%)	6/57 (10.5%)	RR 2.58 (1.08 to 6.12)	166 more per 1,000 (from 8 more to 539 more)	⊕⊕◯◯ Low	Critical

CI: confidence interval; RR: risk ratio

Summary of Judgements

	JUDGEMENT						
**Problem**	No	Probably no	Probably yes	**Yes**		Varies	Don't know
**Desirable Effects**	Trivial	**Small**	Moderate	Large		Varies	Don't know
**Undesirable Effects**	Large	Moderate	Small	Trivial		Varies	**Don't know**
**Certainty of evidence**	Very low	**Low**	Moderate	High			No included studies
**Values**	Important uncertainty or variability	Possibly important uncertainty or variability	**Possibly no important uncertainty or variability**	No important uncertainty or variability			
**Balance of effects**	Favors the comparison	Probably favors the comparison	Does not favor either the intervention or the comparison	**Probably favors the intervention**	Favors the intervention	Varies	Don't know
**Cost effectiveness**	Favors the comparison	Probably favors the comparison	**Does not favor either the intervention or the comparison**	Probably favors the intervention	Favors the intervention	Varies	**No included studies**
**Resources required**	Large costs	Moderate costs	**Negligible costs and savings**	Moderate savings	Large savings	Varies	Don't know
**Acceptability**	No	Probably no	**Probably yes**	Yes		Varies	Don't know
**Feasibility**	No	Probably no	Probably yes	**Yes**		Varies	Don't know

CQ2-10: In patients with sepsis receiving empiric antifungal drugs, are antifungal drugs discontinued using β-D glucan as an indicator?

P	Patients with sepsis who have been administered empiric antifungal drugs
I	Discontinuation using β-D glucan
C	Usual care
O	28 or 30-day mortality, ICU mortality, duration of antifungal administration, ventilator-free days, duration of mechanical ventilation, detection of antifungal resistant candida

Evidence profile

Certainty assessment							Number of patients		Effect		Certainty	Importance
№ of studies	Study design	Risk of bias	Inconsistency	Indirectness	Imprecision	Other considerations	Intervention	Control	Relative (95% CI)	Absolute (95% CI)		
28 or 30-day mortality												
2	randomized trials	not serious	not serious	not serious	serious	none	30/114 (26.3%)	30/115 (26.1%)	RR 1.01 (0.65 to 1.56)	3 more per 1,000 (from 91 fewer to 146 more)	⊕⊕⊕◯ Moderate	Critical
ICU mortality												
2	randomized trials	not serious	not serious	not serious	serious	none	34/114 (29.8%)	33/115 (28.7%)	RR 1.04 (0.70 to 1.56)	11 more per 1,000 (86 fewer to 161 more)	⊕⊕⊕◯ Moderate	Critical
Duration of antifungal administration												
2	randomized trials	serious	not serious	not serious	serious	none	114	115	-	MD 7.64 days shorter (8.74 shorter to 6.54 shorter)	⊕⊕◯◯ Low	Critical
Ventilator-free days												
1	randomized trials	not serious	not serious	not serious	serious	none	54	55	-	MD 2.50 days longer (0.13 longer to 4.87 longer)	⊕⊕⊕◯ Moderate	Critical
Duration of mechanical ventilation												
1	randomized trials	not serious	not serious	not serious	serious	none	60	60	-	MD 0.00 days (3.88 shorter to 3.88 longer)	⊕⊕⊕◯ Moderate	Critical
Detection of antifungal resistant candida												
1	randomized trials	not serious	not serious	not serious	serious	none	5/54 (9.3%)	4/55 (7.3%)	RR 1.27 (0.36 to 4.49)	20 more per 1,000 (47 fewer to 254 more)	⊕⊕⊕◯ Moderate	Important

CI: confidence interval; MD: mean difference; RR: risk ratio

Summary of Judgements

	JUDGEMENT						
**Problem**	No	Probably no	Probably yes	**Yes**		Varies	Don't know
**Desirable Effects**	Trivial	**Small**	Moderate	Large		Varies	Don't know
**Undesirable Effects**	Large	Moderate	Small	**Trivial**		Varies	Don't know
**Certainty of evidence**	Very low	**Low**	Moderate	High			No included studies
**Values**	Important uncertainty or variability	Possibly important uncertainty or variability	**Possibly no important uncertainty or variability**	No important uncertainty or variability			
**Balance of effects**	Favors the comparison	Probably favors the comparison	Does not favor either the intervention or the comparison	**Probably favors the intervention**	Favors the intervention	Varies	Don't know
**Cost effectiveness**	Favors the comparison	Probably favors the comparison	Does not favor either the intervention or the comparison	Probably favors the intervention	Favors the intervention	Varies	**No included studies**
**Resources required**	Large costs	Moderate costs	**Negligible costs and savings**	Moderate savings	Large savings	Varies	Don't know
**Acceptability**	No	Probably no	**Probably yes**	Yes		Varies	Don't know
**Feasibility**	No	Probably no	Probably yes	**Yes**		Varies	Don't know

CQ2-11: Is procalcitonin (PCT) used as an indicator for discontinuing antimicrobial therapy for sepsis?

P	Sepsis / septic shock
I	Procalcitonin-guided therapy, C-reactive protein-guided therapy
C	Usual care (not using indicators)
O	Mortality, recurrence, duration of antimicrobial therapy

Evidence profile (Network meta-analysis)

Outcome		Effects and confidence in the estimate of effects			
		Procalcitonin-guided therapy		C-reactive protein-guided therapy	
**Mortality**					
**Usual care**	234 per 1,000 (23.4%)	RR 0.86 (0.77 to 0.96)	32 fewer per 1,000 (from 53 fewer to 9 fewer)	RR 1.17 (0.81 to 1.68)	39 more per 1,000 (from 44 fewer to 158 more)
		⨁⨁⨁⨁ High (Confidence in estimate) Based on 4,501 patients (13 randomized trials)		⨁⨁⨁◯ Moderate (Confidence in estimate due to imprecision) Based on 130 patients (1 randomized trial)	
**Recurrence**					
**Usual care**	128 per 1,000 (12.8%)	RR 1.05 (0.89 to 1.25)	7 more per 1,000 (from 14 fewer to 32 more)	RR 1.05 (0.89 to 1.25)	7 more per 1,000 (from 14 fewer to 32 more)
		⨁⨁◯◯ Low (Confidence in estimate due to risk of bias and imprecision) Based on 2,924 patients (8 randomized trials)		⨁⨁◯◯ Low (Confidence in estimate due to risk of bias and imprecision) Based on 130 patients (1 randomized trial)	
**Duration of antimicrobial therapy**					
**Usual care**		MD -2.15 days (-2.80 to -1.50)		MD -2.69 days (-4.70 to -0.67)	
		⨁⨁⨁⨁ High (Confidence in estimate) Based on 4,501 patients (13 randomized trials)		⨁⨁⨁⨁ High (Confidence in estimate) Based on 4,501 patients (13 randomized trials)	

CI: confidence interval; MD: mean difference; RR: risk ratio.

Summary of Judgements (Procalcitonin-guided therapy vs. usual care)

	JUDGEMENT						
**Problem**	No	Probably no	Probably yes	**Yes**		Varies	Don't know
**Desirable Effects**	Trivial	Small	**Moderate**	Large		Varies	Don't know
**Undesirable Effects**	Large	Moderate	Small	**Trivial**		Varies	Don't know
**Certainty of evidence**	Very low	Low	Moderate	**High**			No included studies
**Values**	Important uncertainty or variability	Possibly important uncertainty or variability	**Possibly no important uncertainty or variability**	No important uncertainty or variability			
**Balance of effects**	Favors the comparison	Probably favors the comparison	Does not favor either the intervention or the comparison	Probably favors the intervention	**Favors the intervention**	Varies	Don't know
**Cost effectiveness**	Favors the comparison	Probably favors the comparison	Does not favor either the intervention or the comparison	Probably favors the intervention	Favors the intervention	**Varies**	No included studies
**Resources required**	Large costs	Moderate costs	**Negligible costs and savings**	Moderate savings	Large savings	Varies	Don't know
**Acceptability**	No	Probably no	**Probably yes**	Yes		Varies	Don't know
**Feasibility**	No	Probably no	**Probably yes**	Yes		Varies	Don't know

CQ2-12: Is short-term (≤ 7 days) antimicrobial therapy used for sepsis?

P	Sepsis / septic shock
I	Short-term antimicrobial therapy (≤ 7 days)
C	Long-term antimicrobial therapy (> 7 days)
O	Clinical cure, mortality, new infection events, detection of drug-resistant bacteria

Evidence profile

Certainty assessment							Number of patients		Effect		Certainty	Importance
№ of studies	Study design	Risk of bias	Inconsistency	Indirectness	Imprecision	Other considerations	Intervention	Control	Relative (95% CI)	Absolute (95% CI)		
Clinical cure												
3	randomized trials	not serious	not serious	not serious	serious	none	148/314 (47.1%)	157/326 (48.2%)	RR 0.95 (0.80 to 1.13)	24 fewer per 1,000 (from 96 fewer to 63 more)	⊕⊕⊕◯ Moderate	Critical
Mortality												
6	randomized trials	not serious	not serious	not serious	serious	none	116/937 (12.4%)	115/944 (12.2%)	RR 1.04 (0.81 to 1.32)	5 more per 1,000 (from 23 fewer to39 more)	⊕⊕⊕◯ Moderate	Critical
New infection events												
4	randomized trials	not serious	serious	not serious	serious	none	90/661 (13.6%)	70/652 (10.7%)	RR 1.24 (0.81 to 1.89)	26 more per 1,000 (from 20 fewer to 96 more)	⊕⊕◯◯ Low	Critical
Detection of drug-resistant bacteria												
2	randomized trials	not serious	not serious	not serious	very serious	none	49/127 (38.6%)	58/119 (48.7%)	RR 0.73 (0.40 to 1.34)	132 fewer per 1,000 (from 292 fewer to 166 more)	⊕⊕◯◯ Low	Critical

CI: confidence interval; MD: mean difference; RR: risk ratio

Summary of Judgements

	JUDGEMENT						
**Problem**	No	Probably no	Probably yes	**Yes**		Varies	Don't know
**Desirable Effects**	Trivial	Small	**Moderate**	Large		Varies	Don't know
**Undesirable Effects**	Large	Moderate	**Small**	Trivial		Varies	Don't know
**Certainty of evidence**	Very low	**Low**	Moderate	High			No included studies
**Values**	Important uncertainty or variability	Possibly important uncertainty or variability	**Possibly no important uncertainty or variability**	No important uncertainty or variability			
**Balance of effects**	Favors the comparison	Probably favors the comparison	Does not favor either the intervention or the comparison	**Probably favors the intervention**	Favors the intervention	Varies	Don't know
**Cost effectiveness**	Favors the comparison	Probably favors the comparison	Does not favor either the intervention or the comparison	Probably favors the intervention	Favors the intervention	Varies	**No included studies**
**Resources required**	Large costs	Moderate costs	**Negligible costs and savings**	Moderate savings	Large savings	Varies	Don't know
**Acceptability**	No	Probably no	**Probably yes**	Yes		Varies	Don't know
**Feasibility**	No	Probably no	Probably yes	**Yes**		Varies	Don't know

CQ3-3: What is the target mean arterial pressure (MAP) during initial resuscitation for sepsis?

P	Sepsis
I	Higher target mean arterial pressure
C	Lower target mean arterial pressure
O	Short-term mortality, renal replacement therapy, serious adverse events

Evidence profile

Certainty assessment							Number of patients		Effect		Certainty	Importance
№ of studies	Study design	Risk of bias	Inconsistency	Indirectness	Imprecision	Other considerations	Intervention	Control	Relative (95% CI)	Absolute (95% CI)		
Short-term mortality												
3	randomized trials	not serious	not serious	not serious	not serious	none	645/1678 (38.4%)	669/1698 (39.4%)	RR 0.97 (0.89 to 1.06)	12 fewer per 1,000 (from 43 fewer to 24 more)	⊕⊕⊕⊕ High	Critical
Serious adverse events												
3	randomized trials	serious	not serious	not serious	serious	none	151/1729 (8.7%)	121/1747 (6.9%)	RR 1.23 (0.92 to 1.63)	16 more per 1,000 (from 6 fewer to 44 more)	⊕⊕◯◯ Low	Critical
Renal replacement therapy												
2	randomized trials	not serious	not serious	not serious	not serious	none	432/1606 (26.9%)	445/1627 (27.4%)	RR 0.98 (0.88 to 1.10)	5 fewer per 1,000 (from 33 fewer to 27 more)	⊕⊕⊕⊕ High	Critical

CI: confidence interval; RR: risk ratio

Summary of Judgements

	JUDGEMENT						
**Problem**	No	Probably no	Probably yes	**Yes**		Varies	Don't know
**Desirable Effects**	**Trivial**	Small	Moderate	Large		Varies	Don't know
**Undesirable Effects**	Large	Moderate	**Small**	Trivial		Varies	Don't know
**Certainty of evidence**	Very low	**Low**	Moderate	High			No included studies
**Values**	Important uncertainty or variability	Possibly important uncertainty or variability	**Possibly no important uncertainty or variability**	No important uncertainty or variability			
**Balance of effects**	Favors the comparison	Probably favors the comparison	**Does not favor either the intervention or the comparison**	Probably favors the intervention	Favors the intervention	Varies	Don't know
**Cost effectiveness**	Favors the comparison	**Probably favors the comparison**	Does not favor either the intervention or the comparison	Probably favors the intervention	Favors the intervention	Varies	No included studies
**Resources required**	Large costs	Moderate costs	**Negligible costs and savings**	Moderate savings	Large savings	Varies	Don't know
**Acceptability**	No	Probably no	**Probably yes**	Yes		Varies	Don't know
**Feasibility**	No	Probably no	**Probably yes**	Yes		Varies	Don't know

CQ3-4: Which fluid is used for initial resuscitation of sepsis? (Balanced crystalloids)

P	Sepsis / septic shock
I	Balanced crystalloids
C	Normal saline
O	Short-term mortality, renal replacement therapy, hyperkalemia, mechanical ventilation, vasopressor use, acute kidney injury, length of ICU stay

Evidence profile

Certainty assessment							Number of patients		Effect		Certainty	Importance
№ of studies	Study design	Risk of bias	Inconsistency	Indirectness	Imprecision	Other considerations	Intervention	Control	Relative (95% CI)	Absolute (95% CI)		
Short-term mortality												
7	randomized trials	not serious	not serious	serious	not serious	none	1791/9550 (18.8%)	1875/9574 (19.6%)	RR 0.96 (0.91 to 1.02)	8 fewer per 1,000 (from 18 fewer to 4 more)	⊕⊕⊕◯ Moderate	Critical
Renal replacement therapy												
7	randomized trials	not serious	not serious	serious	not serious	none	767/9689 (7.9%)	812/9708 (8.4%)	RR 0.95 (0.86 to 1.04)	4 fewer per 1,000 (from 12 fewer to 3 more)	⊕⊕⊕◯ Moderate	Critical
Hyperkalemia												
3	randomized trials	not serious	not serious	serious	not serious	none	6/2035 (0.3%)	8/1996 (0.4%)	RR 0.73 (0.25 to 2.10)	1 fewer per 1,000 (from 3 fewer to 4 more)	⊕⊕⊕◯ Moderate	Critical
Mechanical ventilation												
1	randomized trials	not serious	not serious	serious	not serious	none	207/303 (68.3%)	197/292 (67.5%)	RR 1.01 (0.91 to 1.13)	7 more per 1,000 (from 61 fewer to 88 more)	⊕⊕⊕◯ Moderate	Critical
Vasopressor use												
2	randomized trials	not serious	not serious	serious	very serious	none	2121/2468 (85.9%)	2137/2467 (86.6%)	RR 1.09 (0.69 to 1.74)	78 more per 1,000 (from 269 fewer to 641 more)	⊕◯◯◯ Very low	Important
Acute kidney injury												
7	randomized trials	not serious	not serious	serious	not serious	none	1339/7576 (17.7%)	1368/7483 (18.3%)	RR 0.97 (0.90 to 1.03)	5 fewer per 1,000 (from 18 fewer to 5 more)	⊕⊕⊕◯ Moderate	Important
Length of ICU stay												
2	randomized trials	not serious	not serious	serious	not serious	none	5002	5085	-	MD 0.11 days longer (0.39 shorter to 0.62 longer)	⊕⊕⊕◯ Moderate	Important

CI: confidence interval; MD: mean difference; RR: risk ratio

Summary of Judgements

	JUDGEMENT						
**Problem**	No	Probably no	Probably yes	**Yes**		Varies	Don't know
**Desirable Effects**	**Trivial**	Small	Moderate	Large		Varies	Don't know
**Undesirable Effects**	Large	Moderate	Small	**Trivial**		Varies	Don't know
**Certainty of evidence**	Very low	**Low**	Moderate	High			No included studies
**Values**	Important uncertainty or variability	Possibly important uncertainty or variability	**Possibly no important uncertainty or variability**	No important uncertainty or variability			
**Balance of effects**	Favors the comparison	Probably favors the comparison	Does not favor either the intervention or the comparison	**Probably favors the intervention**	Favors the intervention	Varies	Don't know

**Cost effectiveness**	Favors the comparison	Probably favors the comparison	Does not favor either the intervention or the comparison	Probably favors the intervention	Favors the intervention	Varies	**No included studies**
**Resources required**	Large costs	Moderate costs	**Negligible costs and savings**	Moderate savings	Large savings	Varies	Don't know
**Acceptability**	No	Probably no	Probably yes	**Yes**		Varies	Don't know
**Feasibility**	No	Probably no	Probably yes	**Yes**		Varies	Don't know

CQ3-4: Which fluid is used for initial resuscitation of sepsis? (Isotonic albumin solutions 4–5%)

P	Sepsis / septic shock
I	Isotonic albumin solutions 4–5%
C	Normal saline, crystalloids
O	Short-term mortality, severe adverse events (pulmonary edema), severe adverse events (lung injury score), length of ICU stay, ventilator-free days, vasopressor-free days

Evidence profile

Certainty assessment							Number of patients		Effect		Certainty	Importance
№ of studies	Study design	Risk of bias	Inconsistency	Indirectness	Imprecision	Other considerations	Intervention	Control	Relative (95% CI)	Absolute (95% CI)		
Short-term mortality												
4	randomized trials	not serious	not serious	not serious	very serious	none	283/804 (35.2%)	305/815 (37.4%)	RR 0.97 (0.75 to 1.26)	11 fewer per 1,000 (from 94 fewer to 97 more)	⊕⊕◯◯ Low	Critical
Severe adverse events (pulmonary edema)												
1	randomized trials	serious	not serious	not serious	serious	none	2/9 (22.2%)	7/9 (77.8%)	RR 0.25 (0.07 to 0.89)	583 fewer per 1,000 (from 723 fewer to 86 fewer)	⊕⊕◯◯ Low	Critical
Severe adverse events (lung injury score)												
1	randomized trials	serious	not serious	not serious	serious	none	12	12	-	MD 0.17 lower (1.95 lower to 1.61 higher)	⊕⊕◯◯ Low	Important
Length of ICU stay												
2	randomized trials	not serious	serious	not serious	not serious	none	783	795	-	MD 0.07 days longer (1.3 shorter to 1.43 longer)	⊕⊕⊕◯ Moderate	Important
Ventilator-free days												
1	randomized trials	not serious	not serious	not serious	serious	none	180	180	-	MD 4.70 days shorter (7.12 shorter to 2.28 shorter)	⊕⊕⊕◯ Moderate	Important
Vasopressor-free days												
1	randomized trials	not serious	not serious	not serious	serious	none	180	180	-	MD 4.60 days shorter (6.87 shorter to 2.33 shorter)	⊕⊕⊕◯ Moderate	Important

CI: confidence interval; MD: mean difference; RR: risk ratio

Summary of Judgements

	JUDGEMENT						
**Problem**	No	Probably no	Probably yes	**Yes**		Varies	Don't know
**Desirable Effects**	Trivial	Small	Moderate	**Large**		Varies	Don't know
**Undesirable Effects**	Large	Moderate	Small	Trivial		Varies	**Don't know**
**Certainty of evidence**	Very low	Low	**Moderate**	High			No included studies
**Values**	Important uncertainty or variability	Possibly important uncertainty or variability	**Possibly no important uncertainty or variability**	No important uncertainty or variability			
**Balance of effects**	Favors the comparison	Probably favors the comparison	Does not favor either the intervention or the comparison	Probably favors the intervention	**Favors the intervention**	Varies	Don't know
**Cost effectiveness**	Favors the comparison	Probably favors the comparison	Does not favor either the intervention or the comparison	Probably favors the intervention	Favors the intervention	Varies	**No included studies**
**Resources required**	Large costs	Moderate costs	**Negligible costs and savings**	Moderate savings	Large savings	Varies	Don't know
**Acceptability**	No	Probably no	**Probably yes**	Yes		Varies	Don't know
**Feasibility**	No	Probably no	**Probably yes**	Yes		Varies	Don't know

CQ3-4: Which fluid is used for initial resuscitation of sepsis? (Synthetic colloids)

P	Sepsis / septic shock
I	Synthetic colloids
C	Normal saline, crystalloids
O	Short-term mortality, renal replacement therapy, serious hemorrhage

Evidence profile

Certainty assessment							Number of patients		Effect		Certainty	Importance
№ of studies	Study design	Risk of bias	Inconsistency	Indirectness	Imprecision	Other considerations	Intervention	Control	Relative (95% CI)	Absolute (95% CI)		
Short-term mortality												
4	randomized trials	not serious	not serious	not serious	not serious	none	409/1293 (31.6%)	400/129 (30.9%)	RR 1.03 (0.92 to 1.15)	9 more per 1,000 (from 25 fewer to 46 more)	⊕⊕⊕⊕ High	Critical
Renal replacement therapy												
2	randomized trials	not serious	not serious	serious	serious	none	112/519 (21.6%)	83/515 (16.1%)	RR 1.34 (1.03 to 1.73)	55 more per 1,000 (from 5 more to 118 more)	⊕⊕◯◯ Low	Critical
Length of ICU stay												
3	randomized trials	not serious	not serious	not serious	serious	none	109	105	-	MD 1.27 days longer (3.63 shorter to 6.18 longer)	⊕⊕⊕◯ Moderate	Important
Serious bleeding events												
3	randomized trials	not serious	not serious	serious	serious	none	77/519 (14.8%)	50/515 (9.7%)	RR 1.50 (1.09 to 2.07)	49 more per 1,000 (from 9 more to 104 more)	⊕⊕◯◯ Low	Critical

CI: confidence interval; MD: mean difference; RR: risk ratio

Summary of Judgements

	JUDGEMENT						
**Problem**	No	Probably no	Probably yes	**Yes**		Varies	Don't know
**Desirable Effects**	**Trivial**	Small	Moderate	Large		Varies	Don't know
**Undesirable Effects**	Large	**Moderate**	Small	Trivial		Varies	Don't know
**Certainty of evidence**	Very low	Low	**Moderate**	High			No included studies
**Values**	Important uncertainty or variability	Possibly important uncertainty or variability	**Possibly no important uncertainty or variability**	No important uncertainty or variability			
**Balance of effects**	Favors the comparison	**Probably favors the comparison**	Does not favor either the intervention or the comparison	Probably favors the intervention	Favors the intervention	Varies	Don't know
**Cost effectiveness**	Favors the comparison	Probably favors the comparison	Does not favor either the intervention or the comparison	Probably favors the intervention	Favors the intervention	Varies	**No included studies**
**Resources required**	Large costs	Moderate costs	**Negligible costs and savings**	Moderate savings	Large savings	Varies	Don't know
**Acceptability**	No	**Probably no**	Probably yes	Yes		Varies	Don't know
**Feasibility**	No	**Probably no**	Probably yes	Yes		Varies	Don't know

CQ3-6: Is early administration of vasopressor performed during initial resuscitation for sepsis?

P	Septic patients with hypotension
I	Initial resuscitation with early administration of vasopressor
C	Initial resuscitation without early administration of vasopressor
O	Short-term mortality, pulmonary edema, acute kidney injury, ischemic organ dysfunction

Evidence profile

Certainty assessment							Number of patients		Effect		Certainty	Importance
№ of studies	Study design	Risk of bias	Inconsistency	Indirectness	Imprecision	Other considerations	Intervention	Control	Relative (95% CI)	Absolute (95% CI)		
28-day mortality												
2	randomized trials	not serious	not serious	not serious	very serious	none	27/204 (13.2%)	35/204 (17.2%)	RR 0.95 (0.30 to 3.02)	9 fewer per 1000 (from 120 fewer to 347 more)	⨁⨁◯◯ Low	Critical
Mortality (2 RCTs 28-day mortality, 1 RCT 90-day mortality, 1 RCT hospital mortality)												
4	randomized trials	not serious	not serious	not serious	serious	none	152/1043 (14.6%)	175/1029 (17.0%)	RR 0.76 (0.53 to 1.10)	41 fewer per 1000 (from 80 fewer to 17 more)	⨁⨁⨁◯ Moderate	Critical
Pulmonary edema												
3	randomized trials	serious	not serious	not serious	serious	none	23/987 (2.3%)	47/985 (4.8%)	RR 0.51 (0.32 to 0.79)	23 fewer per 1000 (from 32 fewer to 10 fewer)	⨁⨁◯◯ Low	Critical
Acute kidney injury												
4	randomized trials	serious	not serious	not serious	serious [b]	none	83/1000 (8.3%)	86/986 (8.7%)	RR 0.88 (0.68 to 1.14)	10 fewer per 1000 (from 28 fewer to 12 more)	⨁⨁◯◯ Low	Critical
Ischemic organ dysfunction												
3	randomized trials	serious	not serious	not serious	very serious	none	10/987 (1.0%)	6/985 (0.6%)	RR 1.65 (0.61 to 4.50)	4 fewer per 1000 (from 2 fewer to 21 more)	⨁◯◯◯ Very low	Critical

CI: confidence interval; RCT, randomized controlled trial; RR: risk ratio

Summary of Judgements

	JUDGEMENT						
**Problem**	No	Probably no	Probably yes	**Yes**		Varies	Don't know
**Desirable Effects**	Trivial	**Small**	Moderate	Large		Varies	Don't know
**Undesirable Effects**	Large	Moderate	Small	**Trivial**		Varies	Don't know
**Certainty of evidence**	Very low	**Low**	Moderate	High			No included studies
**Values**	Important uncertainty or variability	Possibly important uncertainty or variability	**Possibly no important uncertainty or variability**	No important uncertainty or variability			
**Balance of effects**	Favors the comparison	Probably favors the comparison	Does not favor either the intervention or the comparison	**Probably favors the intervention**	Favors the intervention	Varies	Don't know
**Cost effectiveness**	Favors the comparison	Probably favors the comparison	Does not favor either the intervention or the comparison	Probably favors the intervention	Favors the intervention	Varies	**No included studies**
**Resources required**	Large costs	Moderate costs	**Negligible costs and savings**	Moderate savings	Large savings	Varies	Don't know
**Acceptability**	No	Probably no	Probably yes	**Yes**		Varies	Don't know
**Feasibility**	No	Probably no	**Probably yes**	Yes		Varies	Don't know

CQ3-7: Which vasopressor is used as the first-line drug in patients with septic shock? (Noradrenaline)

P	Patients with sepsis who required vasopressors
I	Noradrenaline
C	Dopamine
O	Short-term mortality, arrhythmia, renal replacement therapy, organ ischemia (limb and intestinal tract)

Evidence profile

Certainty assessment							Number of patients		Effect		Certainty	Importance
№ of studies	Study design	Risk of bias	Inconsistency	Indirectness	Imprecision	Other considerations	Intervention	Control	Relative (95% CI)	Absolute (95% CI)		
Short-term mortality												
4	randomized trials	not serious	not serious	not serious	not serious	none	352/706 (49.9%)	403/760 (53.0%)	RR 0.96 (0.81 to 1.13)	21 fewer per 1,000 (from 101 fewer to 69 more)	⊕⊕⊕⊕ High	Critical
Arrhythmia												
3	randomized trials	not serious	serious	not serious	serious	none	112/1009 (11.1%)	239/1060 (22.5%)	RR 0.45 (0.22 to 0.95)	124 fewer per 1,000 (from 176 fewer to 11 fewer)	⊕⊕◯◯ Low	Critical
Renal replacement therapy												
1	randomized trials	not serious	not serious	not serious	very serious	none	61/821 (7.4%)	63/858 (7.3%)	RR 1.01 (0.72 to 1.42)	1 more per 1,000 (from 21 fewer to 31 more)	⊕⊕◯◯ Low	Critical
Ischemic organ injury												
1	randomized trials	not serious	not serious	not serious	very serious	none	20/821 (2.4%)	23/858 (2.7%)	RR 0.91 (0.50 to 1.64)	2 fewer per 1,000 (from 13 fewer to 17 more)	⊕⊕◯◯ Low	Critical
Time to achieve target blood pressure												
1	randomized trials	not serious	not serious	not serious	not serious	none	821	858	-	MD 1.50 days longer (0.34 longer to 2.66 longer)	⊕⊕⊕⊕ High	Important
Length of ICU stay												
2	randomized trials	not serious	not serious	not serious	serious	none	939	992	-	MD 0.50 days longer (0.35 longer to 0.65 longer)	⊕⊕⊕⊕ High	Important

CI: confidence interval; MD: mean difference; RR: risk ratio

Summary of Judgements

	JUDGEMENT						
**Problem**	No	Probably no	Probably yes	**Yes**		Varies	Don't know
**Desirable Effects**	Trivial	Small	Moderate	**Large**		Varies	Don't know
**Undesirable Effects**	Large	Moderate	Small	**Trivial**		Varies	Don't know
**Certainty of evidence**	**Very low**	Low	Moderate	High			No included studies
**Values**	Important uncertainty or variability	Possibly important uncertainty or variability	**Possibly no important uncertainty or variability**	No important uncertainty or variability			
**Balance of effects**	Favors the comparison	Probably favors the comparison	Does not favor either the intervention or the comparison	Probably favors the intervention	**Favors the intervention**	Varies	Don't know
**Cost effectiveness**	Favors the comparison	Probably favors the comparison	Does not favor either the intervention or the comparison	Probably favors the intervention	Favors the intervention	Varies	**No included studies**
**Resources required**	Large costs	Moderate costs	**Negligible costs and savings**	Moderate savings	Large savings	Varies	Don't know
**Acceptability**	No	Probably no	Probably yes	**Yes**		Varies	Don't know
**Feasibility**	No	Probably no	Probably yes	**Yes**		Varies	Don't know

CQ3-7: Which vasopressor is used as the second-line drug in patients with septic shock? (Noradrenaline + vasopressin)

P	Patients whose blood pressure cannot be maintained even with the use of noradrenaline
I	Vasopressin for second-line vasopressor
C	Noradrenaline alone
O	Short-term mortality, acute coronary syndrome, mesenteric Ischemia, arrhythmia, renal replacement therapy, acute kidney injury, shock-free days

Evidence profile

Certainty assessment							Number of patients		Effect		Certainty	Importance
№ of studies	Study design	Risk of bias	Inconsistency	Indirectness	Imprecision	Other considerations	Intervention	Control	Relative (95% CI)	Absolute (95% CI)		
Short-term mortality												
5	randomized trials	not serious	not serious	not serious	not serious	none	218/673 (32.4%)	225/656 (34.3%)	RR 0.94 (0.81 to 1.09)	21 fewer per 1,000 (from 65 fewer to 31 more)	⊕⊕⊕⊕ High	Critical
Acute coronary syndrome												
3	randomized trials	not serious	not serious	not serious	not serious	none	16/614 (2.6%)	10/596 (1.7%)	RR 1.45 (0.65 to 3.24)	583 fewer per 1,000 (from 723 fewer to 86 fewer)	⊕⊕⊕⊕ High	Critical
Mesenteric ischemia												
2	randomized trials	not serious	not serious	not serious	not serious	none	14/601 (2.3%)	18/586 (3.1%)	RR 0.76 (0.38 to 1.51)	7 fewer per 1,000 (from 19 fewer to 16 more)	⊕⊕⊕⊕ High	Critical
Arrhythmia												
3	randomized trials	not serious	not serious	not serious	not serious	none	11/616 (1.8%)	14/601 (2.3%)	RR 0.77 (0.33 to 1.81)	5 fewer per 1,000 (from 16 fewer to 19 more)	⊕⊕⊕⊕ High	Critical
Renal replacement therapy												
3	randomized trials	not serious	not serious	not serious	serious	none	58/265 (21.9%)	87/264 (33.0%)	RR 0.65 (0.42 to 1.00)	115 fewer per 1,000 (from 191 fewer to 0 fewer)	⊕⊕⊕◯ Moderate	Important
Acute kidney injury												
1	randomized trials	not serious	not serious	not serious	serious	none	87/205 (42.4%)	97/204 (47.5%)	RR 0.89 (0.72 to 1.11)	52 fewer per 1,000 (from 133 fewer to 52 more)	⊕⊕⊕◯ Moderate	Important
Shock-free days												
1	randomized trials	not serious	not serious	serious	very serious	none	396	382	-	MD 4.60 days shorter (6.87 shorter to 2.33 shorter)	⊕◯◯◯ Very low	Important

CI: confidence interval; RR: risk ratio

Summary of Judgements

	JUDGEMENT						
**Problem**	No	Probably no	Probably yes	**Yes**		Varies	Don't know
**Desirable Effects**	Trivial	Small	**Moderate**	Large		Varies	Don't know
**Undesirable Effects**	Large	Moderate	Small	**Trivial**		Varies	Don't know
**Certainty of evidence**	Very low	Low	Moderate	**High**			No included studies
**Values**	Important uncertainty or variability	Possibly important uncertainty or variability	**Possibly no important uncertainty or variability**	No important uncertainty or variability			
**Balance of effects**	Favors the comparison	Probably favors the comparison	Does not favor either the intervention or the comparison	Probably favors the intervention	**Favors the intervention**	Varies	Don't know
**Cost effectiveness**	Favors the comparison	Probably favors the comparison	Does not favor either the intervention or the comparison	Probably favors the intervention	Favors the intervention	Varies	**No included studies**

**Resources required**	Large costs	Moderate costs	**Negligible costs and savings**	Moderate savings	Large savings	Varies	Don't know
**Acceptability**	No	Probably no	Probably yes	**Yes**		Varies	Don't know
**Feasibility**	No	Probably no	Probably yes	**Yes**		Varies	Don't know

CQ3-8: Are steroids administered for septic shock?

P	Septic shock patients
I	Low-dose hydrocortisone (200–300 mg/day)
C	Placebo or control
O	Short-term mortality, recovery from shock, duration of recovery from shock, serious adverse events, secondary infections, gastrointestinal hemorrhage

Evidence profile

Certainty assessment							Number of patients		Effect		Certainty	Importance
№ of studies	Study design	Risk of bias	Inconsistency	Indirectness	Imprecision	Other considerations	Intervention	Control	Relative (95% CI)	Absolute (95% CI)		
Short-term mortality												
9	randomized trials	not serious	not serious	not serious	not serious	none	917/3207 (28.6%)	982/3216 (30.5%)	RR 0.96 (0.87 to 1.06)	12 fewer per 1000 (from 40 fewer to 18 more)	⊕⊕⊕⊕ High	Critical
Recovery from shock												
6	randomized trials	not serious	not serious	not serious	not serious	none	474/592 (80.1%)	439/590 (74.4%)	RR 1.08 (0.96 to 1.22)	60 more per 1000 (from 30 fewer to 164 more)	⊕⊕⊕⊕ High	Critical
Duration of recovery from shock												
4	randomized trials	serious	not serious	not serious	not serious	none	2313	2332	-	MD 1.6 days shorter (2.79 shorter to 0.41 shorter)	⊕⊕⊕◯ Moderate	Critical
Serious adverse events												
5	randomized trials	serious	not serious	not serious	serious	none	407/2840 (14.3%)	431/2847 (15.1%)	RR 1.06 (0.83 to 1.36)	9 more per 1000 (from 26 fewer to 54 more)	⊕⊕◯◯ Low	Critical
Secondary infections												
7	randomized trials	serious	not serious	not serious	not serious	none	584/2914 (20.0%)	559/2911 (19.2%)	RR 1.05 (0.95 to 1.16)	10 more per 1000 (from 10 fewer to 31 more)	⊕⊕⊕◯ Moderate	Critical
Gastrointestinal hemorrhage												
6	randomized trials	not serious	not serious	not serious	very serious	none	80/1079 (7.4%)	73/1082 (6.7%)	RR 1.18 (0.76 to 1.81)	12 more per 1000 (from 16 fewer to 55 more)	⊕⊕◯◯ Low	Critical

CI: confidence interval; MD: mean difference; RR: risk ratio

Summary of Judgements

	JUDGEMENT						
**Problem**	No	Probably no	Probably yes	**Yes**		Varies	Don't know
**Desirable Effects**	Trivial	Small	**Moderate**	Large		Varies	Don't know
**Undesirable Effects**	Large	Moderate	Small	**Trivial**		Varies	Don't know
**Certainty of evidence**	Very low	**Low**	Moderate	High			No included studies
**Values**	Important uncertainty or variability	Possibly important uncertainty or variability	**Possibly no important uncertainty or variability**	No important uncertainty or variability			
**Balance of effects**	Favors the comparison	Probably favors the comparison	Does not favor either the intervention or the comparison	**Probably favors the intervention**	Favors the intervention	Varies	Don't know
**Cost effectiveness**	Favors the comparison	Probably favors the comparison	Does not favor either the intervention or the comparison	Probably favors the intervention	Favors the intervention	Varies	**No included studies**
**Resources required**	Large costs	Moderate costs	**Negligible costs and savings**	Moderate savings	Large savings	Varies	Don't know
**Acceptability**	No	Probably no	Probably yes	**Yes**		Varies	Don't know
**Feasibility**	No	Probably no	Probably yes	**Yes**		Varies	Don't know

CQ3-9: What is the threshold of hemoglobin level for transfusion in initial resuscitation for septic shock?

P	Septic shock patients
I	Higher threshold: hemoglobin 8-10 g/dL
C	Lower threshold: hemoglobin 7g/dL
O	Mortality, ischemic organ dysfunction, serious adverse events

Evidence profile

Certainty assessment							Number of patients		Effect		Certainty	Importance
№ of studies	Study design	Risk of bias	Inconsistency	Indirectness	Imprecision	Other considerations	Intervention	Control	Relative (95% CI)	Absolute (95% CI)		
Mortality												
3	randomized trials	serious	serious	not serious	not serious	none	324/669 (48.4%)	333/675 (49.3%)	RR 0.96 (0.80 to 1.14)	20 fewer per 1000 (from 99 fewer to 69 more)	⊕⊕◯◯ Low	Critical
Ischemic organ dysfunction												
2	randomized trials	serious	not serious	not serious	very serious	none	48/638 (7.5%)	47/639 (7.4%)	RR 1.02 (0.69 to 1.51)	1 more per 1000 (from 23 fewer to 38 more)	⊕⊕◯◯ Low	Critical
Serious adverse events												
2	randomized trials	serious	not serious	not serious	very serious	none	1/638 (0.2%)	0/639 (0.0%)	RR 2.99 (0.12 to 73.31)	0 per 1000* (from 0 to 0)	⊕◯◯◯ Very low	Critical

CI: confidence interval; RR: risk ratio

* Assume that one patient in the control group had an event, the RD for serious adverse events increased by 3 per 1,000 (95% CI: 1 fewer to 113 more).

Summary of Judgements

	JUDGEMENT						
**Problem**	No	Probably no	Probably yes	**Yes**		Varies	Don't know
**Desirable Effects**	Trivial	**Small**	Moderate	Large		Varies	Don't know
**Undesirable Effects**	Large	Moderate	Small	**Trivial**		Varies	Don't know
**Certainty of evidence**	Very low	**Low**	Moderate	High			No included studies
**Values**	Important uncertainty or variability	Possibly important uncertainty or variability	**Possibly no important uncertainty or variability**	No important uncertainty or variability			
**Balance of effects**	Favors the comparison	Probably favors the comparison	Does not favor either the intervention or the comparison	**Probably favors the intervention**	Favors the intervention	Varies	Don't know
**Cost effectiveness**	Favors the comparison	Probably favors the comparison	Does not favor either the intervention or the comparison	Probably favors the intervention	Favors the intervention	Varies	**No included studies**
**Resources required**	Large costs	**Moderate costs**	Negligible costs and savings	Moderate savings	Large savings	Varies	Don't know
**Acceptability**	No	**Probably no**	Probably yes	Yes		Varies	Don't know
**Feasibility**	No	Probably no	Probably yes	**Yes**		Varies	Don't know

CQ3-10: Are β_1-receptor blockers used for septic patients with persistent tachycardia after initial resuscitation?

P	septic patients with persistent tachycardia after initial resuscitation
I	β 1-adrenoceptor antagonists to manage persistent tachycardia
C	Usual care
O	Short-term mortality, arrhythmia, serious adverse event

Evidence profile

Certainty assessment							Number of patients		Effect		Certainty	Importance
№ of studies	Study design	Risk of bias	Inconsistency	Indirectness	Imprecision	Other considerations	Intervention	Control	Relative (95% CI)	Absolute (95% CI)		
Short-term mortality												
4	randomized trials	not serious	not serious	not serious	serious	none	65/200 (32.5%)	127/234 (54.3%)	RR 0.62 (0.50 to 0.76)	206 fewer per 1000 (from 271 fewer to 130 fewer)	⊕⊕⊕◯ Moderate	Critical
Arrhythmia												
1	randomized trials	not serious	not serious	not serious	serious	none	7/75 (9.3%)	19/75 (25.3%)	RR 0.37 (0.16 to 0.82)	160 fewer per 1000 (from 213 fewer to 46 fewer)	⊕⊕⊕◯ Moderate	Critical
Serious adverse event												
3	randomized trials	serious	not serious	serious	very serious	none	14/125 (11.2%)	15/156 (9.6%)	RR 1.03 (0.36 to 2.91)	3 more per 1000 (from 62 fewer to 184 more)	⊕◯◯◯ Very low	Critical

CI: confidence interval; RR: risk ratio

Summary of Judgements

	JUDGEMENT						
**Problem**	No	Probably no	Probably yes	**Yes**		Varies	Don't know
**Desirable Effects**	Trivial	Small	Moderate	**Large**		Varies	Don't know
**Undesirable Effects**	Large	Moderate	Small	**Trivial**		Varies	Don't know
**Certainty of evidence**	Very low	**Low**	Moderate	High			No included studies
**Values**	Important uncertainty or variability	Possibly important uncertainty or variability	**Possibly no important uncertainty or variability**	No important uncertainty or variability			
**Balance of effects**	Favors the comparison	Probably favors the comparison	Does not favor either the intervention or the comparison	Probably favors the intervention	**Favors the intervention**	Varies	Don't know
**Cost effectiveness**	Favors the comparison	Probably favors the comparison	Does not favor either the intervention or the comparison	Probably favors the intervention	Favors the intervention	Varies	**No included studies**
**Resources required**	Large costs	Moderate costs	**Negligible costs and savings**	Moderate savings	Large savings	Varies	Don't know
**Acceptability**	No	Probably no	Probably yes	**Yes**		Varies	Don't know
**Feasibility**	No	Probably no	Probably yes	**Yes**		Varies	Don't know

CQ3-11: Is sodium bicarbonate intravenously administered for septic patients with severe metabolic acidosis (pH ≤ 7.2)?

P	Sepsis / septic shock
I	Intravenous administration of sodium bicarbonate
C	Usual care
O	Short-term mortality, new organ failure, renal replacement therapy, duration of vasopressor use, vasopressor-free days, length of ICU stay, severe metabolic adverse events

Evidence profile

Certainty assessment							Number of patients		Effect		Certainty	Importance
№ of studies	Study design	Risk of bias	Inconsistency	Indirectness	Imprecision	Other considerations	Intervention	Control	Relative (95% CI)	Absolute (95% CI)		
Short-term mortality												
1	randomized trials	serious	not serious	serious	serious	none	87/195 (44.6%)	104/194 (53.6%)	RR 0.83 (0.68 to 1.02)	91 fewer per 1,000 (from 172 fewer to 11 more)	⊕◯◯◯ Very low	Critical
New-onset organ failure												
1	randomized trials	serious	not serious	serious	serious	none	121/195 (62.1%)	134/194 (69.1%)	RR 0.90 (0.78 to 1.04)	69 fewer per 1,000 (from 152 fewer to 28 more)	⊕◯◯◯ Very low	Critical
Renal replacement therapy												
1	randomized trials	serious	not serious	serious	serious	none	68/195 (34.9%)	100/194 (51.5%)	RR 0.68 (0.53 to 0.86)	165 fewer per 1,000 (from 242 fewer to 72 fewer)	⊕◯◯◯ Very low	Critical
Duration of vasopressor use												
1	randomized trials	serious	not serious	serious	serious	none	195	194	-	MD 0.3 days longer (0.07 shorter to 0.67 longer)	⊕◯◯◯ Very low	Important
Vasopressor-free days												
1	randomized trials	serious	not serious	serious	serious	none	195	194	-	MD 3.3 days longer (0.56 shorter to 7.16 longer)	⊕◯◯◯ Very low	Important
Length of ICU stay												
1	randomized trials	serious	not serious	serious	serious	none	195	194	-	MD 1.7 days longer (0.24 shorter to 3.64 longer)	⊕◯◯◯ Very low	Important
Severe metabolic adverse events												
1	randomized trials	serious	not serious	serious	very serious	none	44/195 (22.6%)	41/194 (21.1%)	RR 1.07 (0.73 to 1.56)	15 more per 1,000 (from 57 fewer to 118 more)	⊕◯◯◯ Very low	Critical

CI: confidence interval; ICU, intensive care unit; MD: mean difference; RR: risk ratio

Summary of Judgements

	JUDGEMENT						
**Problem**	No	Probably no	Probably yes	**Yes**		Varies	Don't know
**Desirable Effects**	Trivial	Small	Moderate	**Large**		Varies	Don't know
**Undesirable Effects**	Large	Moderate	Small	**Trivial**		Varies	Don't know
**Certainty of evidence**	Very low	**Low**	Moderate	High			No included studies
**Values**	Important uncertainty or variability	Possibly important uncertainty or variability	**Possibly no important uncertainty or variability**	No important uncertainty or variability			
**Balance of effects**	Favors the comparison	Probably favors the comparison	Does not favor either the intervention or the comparison	Probably favors the intervention	**Favors the intervention**	Varies	Don't know

**Cost effectiveness**	Favors the comparison	Probably favors the comparison	Does not favor either the intervention or the comparison	Probably favors the intervention	Favors the intervention	Varies	**No included studies**
**Resources required**	Large costs	Moderate costs	**Negligible costs and savings**	Moderate savings	Large savings	Varies	Don't know
**Acceptability**	No	Probably no	Probably yes	**Yes**		Varies	Don't know
**Feasibility**	No	Probably no	Probably yes	**Yes**		Varies	Don't know

CQ3-13: Is restrictive fluid management provided in septic patients with stable hemodynamics?

P	Sepsis / septic shock
I	Restrictive fluid management
C	Usual care
O	90-day mortality, acute kidney injury, ventilator-free days, serious adverse events

Evidence profile

Certainty assessment							Number of patients		Effect		Certainty	Importance
№ of studies	Study design	Risk of bias	Inconsistency	Indirectness	Imprecision	Other considerations	Intervention	Control	Relative (95% CI)	Absolute (95% CI)		
90-day mortality												
5	randomized trials	not serious	not serious	not serious	not serious	none	473/1730 (27.3%)	494/1747 (28.3%)	RR 0.98 (0.88 to 1.08)	6 fewer per 1000 (from 34 fewer to 23 more)	⊕⊕⊕⊕ High	Critical
Acute kidney injury												
8	randomized trials	not serious	not serious	not serious	not serious	none	273/1776 (15.4%)	306/1802 (17.0%)	RR 0.89 (0.78 to 1.03)	19 fewer per 1000 (from 37 fewer to 5 more)	⊕⊕⊕⊕ High	Critical
Ventilator-free days												
5	randomized trials	not serious	not serious	not serious	serious	none	932	930	-	MD 0.47 days longer (0.54 shorter to 1.48 longer)	⊕⊕⊕◯ Moderate	Critical
Serious adverse events												
5	randomized trials	not serious	not serious	not serious	not serious	none	254/1712 (14.8%)	274/1732 (15.8%)	RR 0.95 (0.82 to 1.10)	8 fewer per 1000 (from 28 fewer to 16 more)	⊕⊕⊕⊕ High	Critical

CI: confidence interval; MD: mean difference; RR: risk ratio

Summary of Judgements

	JUDGEMENT						
**Problem**	No	Probably no	Probably yes	**Yes**		Varies	Don't know
**Desirable Effects**	Trivial	**Small**	Moderate	Large		Varies	Don't know
**Undesirable Effects**	Large	Moderate	Small	**Trivial**		Varies	Don't know
**Certainty of evidence**	Very low	**Low**	Moderate	High			No included studies
**Values**	Important uncertainty or variability	Possibly important uncertainty or variability	**Possibly no important uncertainty or variability**	No important uncertainty or variability			
**Balance of effects**	Favors the comparison	Probably favors the comparison	Does not favor either the intervention or the comparison	**Probably favors the intervention**	Favors the intervention	Varies	Don't know
**Cost effectiveness**	Favors the comparison	Probably favors the comparison	Does not favor either the intervention or the comparison	Probably favors the intervention	Favors the intervention	Varies	**No included studies**
**Resources required**	Large costs	Moderate costs	**Negligible costs and savings**	Moderate savings	Large savings	Varies	Don't know
**Acceptability**	No	Probably no	**Probably yes**	Yes		Varies	Don't know
**Feasibility**	No	Probably no	**Probably yes**	Yes		Varies	Don't know

CQ4-1: Is polymyxin B-immobilized fiber column (PMX-DHP) used for patients with septic shock?

P	Septic shock
I	Polymyxin B-immobilized fiber column
C	Usual care
O	Mortality, organ dysfunction, vasopressor-free days, serious adverse events

Evidence profile

Certainty assessment							Number of patients		Effect		Certainty	Importance
№ of studies	Study design	Risk of bias	Inconsistency	Indirectness	Imprecision	Other considerations	Intervention	Control	Relative (95% CI)	Absolute (95% CI)		
Mortality												
4	randomized trials	not serious	serious	not serious	serious	none	140/406 (34.5%)	130/389 (33.4%)	RR 0.8 (0.60 to 1.33)	37 fewer per 1000 (from 134 fewer to 110 more)	⊕⊕◯◯ Low	Critical
Organ dysfunction												
2	randomized trials	serious	serious	not serious	not serious	none	258	256	-	SMD 0.49 lower (from 1.2 lower to 0.21 higher)	⊕⊕◯◯ Low	Critical
Ventilator-free days												
1	randomized trials	serious	not serious	not serious	serious	none	119	113	-	MD 1.8 days shorter (from 4.14 shorter to 0.54 longer)	⊕◯◯◯ Very low	Critical
Serious adverse events												
3	randomized trials	serious	serious	not serious	very serious	none	110/365 (30.1%)	87/363 (24.0%)	RR 1.90 (0.62 to 5.75)	216 more per 1000 (from 91 fewer to 1000 more)	⊕◯◯◯ Very low	Critical

CI: confidence interval; MD: mean difference; RR: risk ratio; SMD: standardized mean difference.

Summary of Judgements

	JUDGEMENT						
**Problem**	No	Probably no	Probably yes	**Yes**		Varies	Don't know
**Desirable Effects**	**Trivial**	Small	Moderate	Large		Varies	Don't know
**Undesirable Effects**	**Large**	Moderate	Small	Trivial		Varies	Don't know
**Certainty of evidence**	**Very low**	Low	Moderate	High			No included studies
**Values**	Important uncertainty or variability	Possibly important uncertainty or variability	**Possibly no important uncertainty or variability**	No important uncertainty or variability			
**Balance of effects**	**Favors the comparison**	Probably favors the comparison	Does not favor either the intervention or the comparison	Probably favors the intervention	Favors the intervention	Varies	Don't know
**Cost effectiveness**	Favors the comparison	Probably favors the comparison	Does not favor either the intervention or the comparison	Probably favors the intervention	Favors the intervention	Varies	**No included studies**
**Resources required**	Large costs	**Moderate costs**	Negligible costs and savings	Moderate savings	Large savings	Varies	Don't know
**Acceptability**	No	**Probably no**	Probably yes	Yes		Varies	Don't know
**Feasibility**	No	Probably no	**Probably yes**	Yes		Varies	Don't know

CQ4-2: Is early renal replacement therapy (RRT) performed for septic AKI?

P	Septic acute kidney injury
I	Early initiation of renal replacement therapy (AKI stage 2/3 or within 12 h fulfilling inclusion criteria)
C	Delayed initiation of renal replacement therapy
O	Short-term mortality, dialysis dependence (longest observation period), short-term mortality or dialysis dependence (longest observation period), adverse events (hemorrhage)

Evidence profile

Certainty assessment							Number of patients		Effect		Certainty	Importance
№ of studies	Study design	Risk of bias	Inconsistency	Indirectness	Imprecision	Other considerations	Intervention	Control	Relative (95% CI)	Absolute (95% CI)		
Short-term mortality												
4	randomized trials	not serious	not serious	not serious	not serious	none	794/2070 (38.4%)	778/2064 (37.7%)	RR 1.02 (0.94 to 1.10)	8 more per 1000 (from 23 fewer to 38 more)	⊕⊕⊕⊕ High	Critical
Dialysis dependence (longest observation period)												
4	randomized trials	not serious	serious	not serious	very serious	none	90/1102 (8.2%)	62/1113 (5.6%)	RR 0.79 (0.28 to 2.25)	12 fewer per 1000 (from 40 fewer to 70 more)	⊕◯◯◯ Very low	Critical
Short-term mortality or dialysis dependence (longest observation period)												
4	randomized trials	not serious	not serious	not serious	not serious	none	1039/2055 (50.6%)	1001/2052 (48.8%)	RR 1.04 (0.97 to 1.10)	20 more per 1000 (from 15 fewer to 49 more)	⊕⊕⊕⊕ High	Critical
Adverse events (hemorrhage)												
4	randomized trials	serious	not serious	not serious	very serious	none	50/2108 (2.4%)	59/2091 (2.8%)	RR 0.84 (0.56 to 1.27)	5 fewer per 1000 (from 12 fewer to 8 more)	⊕◯◯◯ Very low	Critical

CI: confidence interval; RR: risk ratio.

Summary of Judgements

	JUDGEMENT						
**Problem**	No	Probably no	Probably yes	**Yes**		Varies	Don't know
**Desirable Effects**	**Trivial**	Small	Moderate	Large		Varies	Don't know
**Undesirable Effects**	Large	Moderate	Small	**Trivial**		Varies	Don't know
**Certainty of evidence**	Very low	**Low**	Moderate	High			No included studies
**Values**	Important uncertainty or variability	Possibly important uncertainty or variability	**Possibly no important uncertainty or variability**	No important uncertainty or variability			
**Balance of effects**	Favors the comparison	Probably favors the comparison	Does not favor either the intervention or the comparison	Probably favors the intervention	Favors the intervention	Varies	**Don't know**
**Cost effectiveness**	Favors the comparison	Probably favors the comparison	Does not favor either the intervention or the comparison	Probably favors the intervention	Favors the intervention	Varies	**No included studies**
**Resources required**	Large costs	**Moderate costs**	Negligible costs and savings	Moderate savings	Large savings	Varies	Don't know
**Acceptability**	No	Probably no	**Probably yes**	Yes		Varies	Don't know
**Feasibility**	No	Probably no	**Probably yes**	Yes		Varies	Don't know

CQ4-3: Is continuous treatment provided in RRT for septic AKI?

P	Septic acute kidney injury
I	Continuous renal replacement therapy
C	Intermittent renal replacement therapy
O	Mortality, dialysis dependence (longest observation period), mortality or dialysis dependence (longest observation period), adverse events (hemorrhage)

Evidence profile

Certainty assessment							Number of patients		Effect		Certainty	Importance
№ of studies	Study design	Risk of bias	Inconsistency	Indirectness	Imprecision	Other considerations	Intervention	Control	Relative (95% CI)	Absolute (95% CI)		
Mortality												
5	randomized trials	serious	not serious	not serious	serious	none	287/503 (57.1%)	272/501 (54.3%)	RR 1.07 (0.91 to 1.25)	38 more per 1,000 (from 49 fewer to 136 more)	⊕⊕◯◯ Low	Critical
Dialysis dependence (longest observation period)												
3	randomized trials	very serious	not serious	serious	very serious	none	9/127 (7.1%)	10/146 (6.8%)	RR 1.06 (0.44 to 2.55)	4 more per 1,000 (from 38 fewer to 106 more)	⊕◯◯◯ Very low	Critical
Mortality or dialysis dependence (longest observation period)												
2	randomized trials	serious	serious	serious	serious	none	93/154 (60.4%)	71/137 (51.8%)	RR 1.14 (0.78 to 1.69)	73 more per 1,000 (from 14 fewer to 358 more)	⊕◯◯◯ Very low	Critical
Adverse events (hemorrhage)												
2	randomized trials	very serious	not serious	not serious	very serious	none	16/297 (5.4%)	18/312 (5.8%)	RR 0.94 (0.49 to 1.80)	3 fewer per 1,000 (from 29 fewer to 46 more)	⊕◯◯◯ Very low	Critical

CI: confidence interval; RR: risk ratio.

Summary of Judgements

	JUDGEMENT						
**Problem**	No	Probably no	Probably yes	**Yes**		Varies	Don't know
**Desirable Effects**	**Trivial**	Small	Moderate	Large		Varies	Don't know
**Undesirable Effects**	Large	**Moderate**	Small	Trivial		Varies	Don't know
**Certainty of evidence**	**Very low**	Low	Moderate	High			No included studies
**Values**	Important uncertainty or variability	Possibly important uncertainty or variability	**Possibly no important uncertainty or variability**	No important uncertainty or variability			
**Balance of effects**	Favors the comparison	**Probably favors the comparison**	Does not favor either the intervention or the comparison	Probably favors the intervention	Favors the intervention	Varies	Don't know
**Cost effectiveness**	Favors the comparison	Probably favors the comparison	Does not favor either the intervention or the comparison	Probably favors the intervention	Favors the intervention	Varies	**No included studies**
**Resources required**	Large costs	**Moderate costs**	Negligible costs and savings	Moderate savings	Large savings	Varies	Don't know
**Acceptability**	No	Probably no	Probably yes	**Yes**		Varies	Don't know
**Feasibility**	No	Probably no	Probably yes	**Yes**		Varies	Don't know

CQ4-4: Is treatment dose increased in RRT for septic AKI?

P	Septic acute kidney injury
I	Increasing RRT dose
C	International standard dose (20–25 mL/kg/h)
O	Mortality, dialysis dependence (longest observation period), mortality or dialysis dependence (longest observation period), adverse events (hypophosphatemia)

Evidence profile

Certainty assessment							Number of patients		Effect		Certainty	Importance
№ of studies	Study design	Risk of bias	Inconsistency	Indirectness	Imprecision	Other considerations	Intervention	Control	Relative (95% CI)	Absolute (95% CI)		
Mortality												
3	randomized trials	not serious	not serious	not serious	not serious	none	630/1385 (45.5%)	603/1404 (42.9%)	RR 1.06 (0.98 to 1.15)	26 more per 1,000 (from 9 fewer to 64 more)	⊕⊕⊕⊕ High	Critical
Dialysis dependence (longest observation period)												
3	randomized trials	not serious	not serious	not serious	not serious	none	896/1381 (64.9%)	887/1404 (63.2%)	RR 1.02 (0.97 to1 .06)	13 more per 1,000 (from 19 fewer to 38 more)	⊕⊕⊕⊕ High	Critical
Mortality or dialysis dependence (longest observation period)												
2	randomized trials	not serious	not serious	not serious	serious	none	457/988 (46.3%)	429/1006 (42.6%)	RR 1.16 (0.88 to 1.53)	68 more per 1,000 (from 51 fewer to 226 more)	⊕⊕⊕◯ Moderate	Critical
Adverse events (hypophosphatemia)												
2	randomized trials	not serious	not serious	not serious	not serious	none	560/1271 (44.1%)	457/1294 (35.3%)	RR 1.35 (1.01 to 1.81)	124 more per 1,000 (from 4 more to 286 more)	⊕⊕⊕⊕ High	Critical

CI: confidence interval; RR: risk ratio.

Summary of Judgements

	JUDGEMENT						
Problem	No	Probably no	Probably yes	**Yes**		Varies	Don't know
Desirable Effects	Trivial	Small	Moderate	Large		Varies	**Don't know**
Undesirable Effects	**Large**	Moderate	Small	Trivial		Varies	Don't know
Certainty of evidence	Very low	Low	Moderate	**High**			No included studies
Values	Important uncertainty or variability	Possibly important uncertainty or variability	**Possibly no important uncertainty or variability**	No important uncertainty or variability			
Balance of effects	**Favors the comparison**	Probably favors the comparison	Does not favor either the intervention or the comparison	Probably favors the intervention	Favors the intervention	Varies	Don't know
Cost effectiveness	**Favors the comparison**	Probably favors the comparison	Does not favor either the intervention or the comparison	Probably favors the intervention	Favors the intervention	Varies	No included studies
Resources required	Large costs	**Moderate costs**	Negligible costs and savings	Moderate savings	Large savings	Varies	Don't know
Acceptability	No	Probably no	**Probably yes**	Yes		Varies	Don't know
Feasibility	No	Probably no	**Probably yes**	Yes		Varies	Don't know

CQ5-3: Is antithrombin administered for sepsis-induced DIC?

P	Sepsis-induced DIC
I	Antithrombin
C	Usual care
O	Mortality, bleeding complications, recovery from DIC

Evidence profile

Certainty assessment							Number of patients		Effect		Certainty	Importance
№ of studies	Study design	Risk of bias	Inconsistency	Indirectness	Imprecision	Other considerations	Intervention	Control	Relative (95% CI)	Absolute (95% CI)		
Mortality												
5	randomized trials	not serious	not serious	serious	serious	none	62/198 (31.3%)	86/193 (44.6%)	RR 0.67 (0.52 to 0.85)	147 fewer per 1,000 (from 214 fewer to 67 fewer)	⊕⊕◯◯ Low	Critical
Bleeding complications												
3	randomized trials	not serious	not serious	not serious	very serious	none	8/158 (5.1%	7/163 (4.3%)	RR 1.18 (0.45 to 3.08)	8 more per 1,000 (from 24 fewer to 89 more)	⊕⊕◯◯ Low	Critical
Recovery from disseminated intravascular coagulation												
2	randomized trials	serious	not serious	not serious	serious	none	32/51 (62.7%)	8/55 (14.5%)	RR 4.08 (2.11 to 7.87)	448 more per 1,000 (from 161 more to 999 more)	⊕⊕◯◯ Low	Important

CI: confidence interval; RR: risk ratio.

Summary of Judgements

	JUDGEMENT						
**Problem**	No	Probably no	Probably yes	**Yes**		Varies	Don't know
**Desirable Effects**	Trivial	Small	Moderate	**Large**		Varies	Don't know
**Undesirable Effects**	Large	Moderate	Small	**Trivial**		Varies	Don't know
**Certainty of evidence**	Very low	Low	**Moderate**	High			No included studies
**Values**	Important uncertainty or variability	Possibly important uncertainty or variability	**Possibly no important uncertainty or variability**	No important uncertainty or variability			
**Balance of effects**	Favors the comparison	Probably favors the comparison	Does not favor either the intervention or the comparison	Probably favors the intervention	**Favors the intervention**	Varies	Don't know
**Cost effectiveness**	Favors the comparison	Probably favors the comparison	Does not favor either the intervention or the comparison	Probably favors the intervention	Favors the intervention	Varies	**No included studies**
**Resources required**	Large costs	Moderate costs	**Negligible costs and savings**	Moderate savings	Large savings	Varies	Don't know
**Acceptability**	No	Probably no	**Probably yes**	Yes		Varies	Don't know
**Feasibility**	No	Probably no	**Probably yes**	Yes		Varies	Don't know

CQ5-4: Is recombinant thrombomodulin administered for sepsis-induced DIC?

P	Sepsis-induced DIC
I	Recombinant thrombomodulin
C	Usual care
O	Mortality, bleeding complications, recovery from DIC

Evidence profile

Certainty assessment							Number of patients		Effect		Certainty	Importance
№ of studies	Study design	Risk of bias	Inconsistency	Indirectness	Imprecision	Other considerations	Intervention	Control	Relative (95% CI)	Absolute (95% CI)		
Mortality												
4	randomized trials	not serious	not serious	not serious	serious	none	163/753 (21.6%)	199/774 (25.7%)	RR 0.85 (0.71 to 1.01)	39 fewer per 1,000 (from 75 fewer to 3 more)	⊕⊕⊕◯ Moderate	Critical
Bleeding complications												
4	randomized trials	not serious	not serious	not serious	serious	none	44/842 (5.2%)	34/851 (4.0%)	RR 1.31 (0.84 to 2.02)	12 more per 1,000 (from 6 fewer to 41 more)	⊕⊕⊕◯ Moderate	Critical
Recovery from disseminated intravascular coagulation												
3	randomized trials	not serious	not serious	not serious	serious	none	61/122 (50.0%)	46/126 (36.5%)	RR 1.33 (1.01 to 1.75)	120 more per 1,000 (from 4 more to 274 more)	⊕⊕⊕◯ Moderate	Important

CI: confidence interval; RR: risk ratio.

Summary of Judgements

	JUDGEMENT						
**Problem**	No	Probably no	Probably yes	**Yes**		Varies	Don't know
**Desirable Effects**	Trivial	Small	Moderate	**Large**		Varies	Don't know
**Undesirable Effects**	Large	Moderate	Small	**Trivial**		Varies	Don't know
**Certainty of evidence**	Very low	Low	**Moderate**	High			No included studies
**Values**	Important uncertainty or variability	Possibly important uncertainty or variability	**Possibly no important uncertainty or variability**	No important uncertainty or variability			
**Balance of effects**	Favors the comparison	Probably favors the comparison	Does not favor either the intervention or the comparison	Probably favors the intervention	**Favors the intervention**	Varies	Don't know
**Cost effectiveness**	Favors the comparison	Probably favors the comparison	Does not favor either the intervention or the comparison	Probably favors the intervention	Favors the intervention	Varies	**No included studies**
**Resources required**	Large costs	Moderate costs	**Negligible costs and savings**	Moderate savings	Large savings	Varies	Don't know
**Acceptability**	No	Probably no	**Probably yes**	Yes		Varies	Don't know
**Feasibility**	No	Probably no	**Probably yes**	Yes		Varies	Don't know

CQ6-1: Is intravenous immunoglobulin (IVIG) administered for sepsis?

P	Sepsis / septic shock
I	Intravenous immunoglobulin
C	Usual care
O	Short-term mortality, long-term mortality, serious adverse events

Evidence profile

Certainty assessment							Number of patients		Effect		Certainty	Importance
№ of studies	Study design	Risk of bias	Inconsistency	Indirectness	Imprecision	Other considerations	Intervention	Control	Relative (95% CI)	Absolute (95% CI)		
Short-term mortality												
3	randomized trials	not serious	not serious	serious	serious	none	133/381 (34.9%)	123/364 (33.8%)	RR 1.04 (0.85 to 1.26)	14 more per 1,000 (from 51 fewer to 88 more)	⊕⊕◯◯ Low	Critical
Short-term mortality (low risk of bias)												
2	randomized trials	not serious	not serious	serious	very serious	none	7/60 (11.7%)	10/61 (16.4%)	RR 0.70 (0.23 to 2.18)	49 more per 1,000 (from 126 fewer to 193 more)	⊕◯◯◯ Very low	Critical
Long-term mortality												
2	randomized trials	not serious	not serious	serious	very serious	none	13/60 (21.7%)	18/61 (29.5%)	RR 0.74 (0.40 to1.37)	77 fewer per 1,000 (from 177 fewer to 109 more)	⊕◯◯◯ Very low	Critical
Long-term mortality (low risk of bias)												
2	randomized trials	not serious	not serious	serious	very serious	none	13/60 (21.7%)	18/61 (29.5%)	RR 0.74 (0.40 to 1.37)	77 fewer per 1,000 (from 177 fewer to 109 more)	⊕◯◯◯ Very low	Critical
Serious adverse events												
2	randomized trials	not serious	not serious	serious	very serious	none	15/371 (4.0%)	15/353 (4.2%)	RR 0.97 (0.45 to 2.08)	1 fewer per 1,000 (from 23 fewer to 46 more)	⊕◯◯◯ Very low	Critical
Serious adverse events (low risk of bias)												
1	randomized trials	not serious	not serious	serious	very serious	none	8/50 (16.0%)	11/50 (22.0%)	RR 0.73 (0.32 to 1.65)	59 fewer per 1,000 (from 150 fewer to 143 more)	⊕◯◯◯ Very low	Critical

CI: confidence interval; RR: risk ratio.

Summary of Judgements

	JUDGEMENT						
Problem	No	Probably no	Probably yes	**Yes**		Varies	Don't know
Desirable Effects	**Trivial**	Small	Moderate	Large		Varies	Don't know
Undesirable Effects	Large	Moderate	**Small**	Trivial		Varies	Don't know
Certainty of evidence	Very low	**Low**	Moderate	High			No included studies
Values	Important uncertainty or variability	Possibly important uncertainty or variability	**Possibly no important uncertainty or variability**	No important uncertainty or variability			
Balance of effects	Favors the comparison	**Probably favors the comparison**	Does not favor either the intervention or the comparison	Probably favors the intervention	Favors the intervention	Varies	Don't know
Cost effectiveness	Favors the comparison	Probably favors the comparison	Does not favor either the intervention or the comparison	Probably favors the intervention	Favors the intervention	Varies	**No included studies**
Resources required	Large costs	Moderate costs	**Negligible costs and savings**	Moderate savings	Large savings	Varies	Don't know
Acceptability	No	Probably no	**Probably yes**	Yes		Varies	Don't know
Feasibility	No	Probably no	Probably yes	**Yes**		Varies	Don't know

CQ6-2: Is high-dose vitamin C therapy used for sepsis?

P	Sepsis
I	High-dose vitamin C therapy
C	Usual care
O	Short-term mortality, long-term mortality, serious adverse events

Evidence profile

Certainty assessment							Number of patients		Effect		Certainty	Importance
№ of studies	Study design	Risk of bias	Inconsistency	Indirectness	Imprecision	Other considerations	Intervention	Control	Relative (95% CI)	Absolute (95% CI)		
Short-term mortality												
15	randomized trials	serious	not serious	not serious	not serious	none	468/1526 (30.7%)	486/1523 (31.9%)	RR 0.92 (0.81 to 1.06)	26 fewer per 1,000 (from 61 fewer to 19 more)	⨁⨁⨁◯ Moderate	Critical
Long-term mortality												
6	randomized trials	not serious	not serious	not serious	not serious	none	434/1072 (40.5%)	410/1076 (38.1%)	RR 1.06 (0.96 to 1.18)	23 more per 1,000 (from 15 fewer to 69 more)	⨁⨁⨁⨁ High	Critical
Hospital mortality												
9	randomized trials	serious	not serious	not serious	not serious	none	202/686 (29.4%)	200/686 (29.2%)	RR 1.02 (0.87 to 1.20)	6 more per 1,000 (from 38 fewer to 58 more)	⨁⨁⨁◯ Moderate	Critical
Acute kidney injury												
6	randomized trials	not serious	not serious	serious	serious	none	209/919 (22.7%)	197/927 (21.3%)	RR 1.12 (0.84 to 1.49)	26 more per 1,000 (from 34 fewer to 104 more)	⨁⨁◯◯ Low	Critical

CI: confidence interval; RR: risk ratio.

Summary of Judgements

	JUDGEMENT						
**Problem**	No	Probably no	Probably yes	**Yes**		Varies	Don't know
**Desirable Effects**	Trivial	Small	Moderate	Large		Varies	**Don't know**
**Undesirable Effects**	Large	**Moderate**	Small	Trivial		Varies	Don't know
**Certainty of evidence**	Very low	Low	**Moderate**	High			No included studies
**Values**	Important uncertainty or variability	Possibly important uncertainty or variability	**Possibly no important uncertainty or variability**	No important uncertainty or variability			
**Balance of effects**	Favors the comparison	**Probably favors the comparison**	Does not favor either the intervention or the comparison	Probably favors the intervention	Favors the intervention	Varies	Don't know
**Cost effectiveness**	Favors the comparison	Probably favors the comparison	Does not favor either the intervention or the comparison	Probably favors the intervention	Favors the intervention	Varies	**No included studies**
**Resources required**	Large costs	Moderate costs	**Negligible costs and savings**	Moderate savings	Large savings	Varies	Don't know
**Acceptability**	No	Probably no	**Probably yes**	Yes		Varies	Don't know
**Feasibility**	No	Probably no	**Probably yes**	Yes		Varies	Don't know

CQ6-3: What is the target blood glucose level for sepsis?

P	Sepsis
I	Target blood glucose level < 110, 110–144, 144–180, > 180 mg/dL
C	
O	28 or 30-day mortality, hospital mortality, long-term mortality, infection, hypoglycemia

Evidence profile (Network meta-analysis)
28 or 30-day mortality

№ of studies	Relative effect (95% CI)	Effects and confidence in the estimate of effects			Certainty	Ranking (SUCRA)
		Control	Intervention	Absolute effect (95% CI)		
> 180 mg/dL<br>6 RCTs, 2,999 patients	RR 1.02<br>(0.85–1.25)	312/1000	316/1000	4 more per 1,000<br>(from 34 fewer to 50 more)	⊕⊕◯◯<br>Low	3<br>(43.1)
144–180 mg/dL<br>4 RCTs, 9,811 patients	RR 0.94<br>(0.78–1.11)	224/1000	213/1000	11 fewer per 1,000<br>(from 40 fewer to 19 more)	⊕⊕◯◯<br>Low	1<br>(78.3)
110–144 mg/dL<br>1 RCT, 90 patients	RR 1.19<br>(0.65–2.27)	333/1000	373/1000	40 more per 1,000<br>(from 88 fewer to 198 more)	⊕⊕◯◯<br>Low	4<br>(28.9)
< 110 mg/dL	(Reference)					2<br>(49.7)
> 180 mg/dL<br>2 RCTs, 198 patients	RR 0.86<br>(0.46–1.59)	263/1000	235/1000	28 fewer per 1,000<br>(from 122 fewer to 99 more)	⊕⊕◯◯<br>Low	-
144–180 mg/dL<br>no RCTs, indirect evidence	RR 0.79<br>(0.41–1.48)	263/1000	220/1000	43 fewer per 1,000<br>(from 135 fewer to 83 more)	⊕◯◯◯<br>Very low	-
110–144 mg/dL	(Reference)					-
> 180 mg/dL<br>1 RCT, 212 patients	RR 1.09<br>(0.86–1.43)	10/1000	11/1000	1 more per 1,000<br>(from 1 fewer to 4 more)	⊕◯◯◯<br>Very low	-
144–180 mg/dL	(Reference)					-

CI: confidence interval; RR: risk ratio; SUCRA: surface under the cumulative ranking.

Hospital mortality

№ of studies	Relative effect (95% CI)	Effects and confidence in the estimate of effects			Certainty	Ranking (SUCRA)
		Control	Intervention	Absolute effect (95% CI)		
> 180 mg/dL<br>9 RCTs, 4,722 patients	RR 1.14<br>(0.90–1.42)	269/1000	296/1000	27 more per 1,000<br>(from 20 fewer to 74 more)	⊕◯◯◯<br>Very low	4<br>(13.7)
144–180 mg/dL<br>5 RCTs, 3,963 patients	RR 0.92<br>(0.63–1.21)	259/1000	243/1000	16 fewer per 1,000<br>(from 79 fewer to 38 more)	⊕◯◯◯<br>Very low	1<br>(71.7)
110–144 mg/dL	RR 0.93	265/1000	251/1000	14 fewer per 1,000	⊕◯◯◯	2

no RCTs, indirect evidence	(0.54–1.57)			(from 102 fewer to 96 more)	Very low	(63.4)
< 110 mg/dL	(Reference)					3<br>(51.3)
> 180 mg/dL<br>5 RCTs, 384 patients	RR 1.23<br>(0.74–2.02)	257/1000	298/1000	41 more per 1,000<br>(from 53 fewer to 154 more)	⊕⊕◯◯<br>Low	-
144–180 mg/dL<br>2 RCTs, 89 patients	RR 0.99<br>(0.53–1.71)	261/1000	259/1000	2 fewer per 1,000<br>(from 103 fewer to 116 more)	⊕⊕◯◯<br>Low	-
110–144 mg/dL	(Reference)					-
>180 mg/dL<br>1 RCT, 212 patients	RR 1.23<br>(0.88–1.90)	10/1000	12/1000	2 more per 1,000<br>(from 1 fewer to 9 more)	⊕◯◯◯<br>Very low	-
144–180 mg/dL	(Reference)					-

CI: confidence interval; RR: risk ratio; SUCRA: surface under the cumulative ranking.

Long-term mortality

№ of studies	Relative effect (95% CI)	Effects and confidence in the estimate of effects			Certainty	Ranking (SUCRA)
		Control	Intervention	Absolute effect (95% CI)		
> 180 mg/dL<br>8 RCTs, 3,210 patients	RR 1.02<br>(0.86–1.21)	383/1000	388/1000	5 more per 1,000<br>(from 35 fewer to 46 more)	⊕⊕⊕◯<br>Moderate	4<br>(34.5)
144–180 mg/dL<br>4 RCTs, 8,830 patients	RR 0.93<br>(0.76–1.10)	291/1000	276/1000	15 fewer per 1,000<br>(from 53 fewer to 20 more)	⊕⊕⊕◯<br>Moderate	1<br>(72.1)
110–144 mg/dL<br>no RCTs, indirect evidence	RR 0.96<br>(0.40–2.31)	315/1000	306/1000	9 fewer per 1,000<br>(from 160 fewer to 200 more)	⊕⊕◯◯<br>Low	2<br>(52.1)
< 110 mg/dL	(Reference)					3<br>(41.3)
> 180 mg/dL<br>3 RCTs, 221 patients	RR 1.06<br>(0.45–2.52)	109/1000	115/1000	6 more per 1,000<br>(from 57 fewer to 127 more)	⊕⊕◯◯<br>Low	-
144–180 mg/dL<br>no RCTs, indirect evidence	RR 0.97<br>(0.40–2.45)	109/1000	106/1000	3 fewer per 1,000<br>(from 135 fewer to 83 more)	⊕⊕◯◯<br>Low	-
110–144 mg/dL	(Reference)					-
>180 mg/dL<br>1 RCT, 419 patients	RR 1.10<br>(0.88–1.40)	249/1000	267/1000	18 more per 1,000<br>(from 23 fewer to 68 more)	⊕⊕⊕◯<br>Moderate	-
144–180 mg/dL	(Reference)					-

CI: confidence interval; RR: risk ratio; SUCRA: surface under the cumulative ranking.

Infection

№ of studies	Relative effect (95% CI)	Effects and confidence in the estimate of effects			Certainty	Ranking (SUCRA)
		Control	Intervention	Absolute effect (95% CI)		
> 180 mg/dL<br>8 RCTs, 3,935 patients	RR 1.35<br>(1.05–1.72)	162/1000	207/1000	45 more per 1,000<br>(from 7 more to 88 more)	⊕⊕⊕◯<br>Moderate	4<br>(0.9)
144–180 mg/dL<br>4 RCTs, 8,831 patients	RR 0.91<br>(0.66–1.17)	139/1000	128/1000	11 fewer per 1,000<br>(from 43 fewer to 20 more)	⊕⊕◯◯<br>Low	2<br>(69.5)
110–144 mg/dL<br>no RCTs, indirect evidence	RR 0.82<br>(0.51–1.23)	146/1000	123/1000	23 fewer per 1,000<br>(from 66 fewer to 28 more)	⊕◯◯◯<br>Very low	1<br>(85.0)
< 110 mg/dL	(Reference)					3<br>(44.6)
> 180 mg/dL<br>5 RCTs, 485 patients	RR 1.66<br>(1.12–2.56)	269/1000	379/1000	110 more per 1,000<br>(from 23 more to 216 more)	⊕⊕⊕◯<br>Moderate	-
144–180 mg/dL<br>2 RCTs, 357 patients	RR 1.11<br>(0.72–1.75)	274/1000	295/1000	21 more per 1,000<br>(from 60 fewer to 124 more)	⊕⊕◯◯<br>Low	-
110–144 mg/dL	(Reference)					-
>180 mg/dL<br>2 RCTs, 631 patients	RR 1.49<br>(1.09–2.11)	67/1000	97/1000	30 more per 1,000<br>(from 6 more to 65 more)	⊕⊕⊕◯<br>Moderate	-
144–180 mg/dL	(Reference)					-

CI: confidence interval; RR: risk ratio; SUCRA: surface under the cumulative ranking.

Hypoglycemia

№ of studies	Relative effect (95% CI)	Effects and confidence in the estimate of effects			Certainty	Ranking (SUCRA)
		Control	Intervention	Absolute effect (95% CI)		
> 180 mg/dL<br>10 RCTs, 5,213 patients	RR 0.16<br>(0.09–0.28)	149/1000	27/1000	122 fewer per 1,000<br>(from 133 fewer to 102 fewer)	⊕⊕◯◯<br>Low	1<br>(87.1)
144–180 mg/dL<br>6 RCTs, 9,932 patients	RR 0.19<br>(0.08–0.39)	87/1000	18/1000	69 fewer per 1,000<br>(from 79 fewer to 51 fewer)	⊕⊕◯◯<br>Low	2<br>(77.6)
110–144 mg/dL<br>1 RCT, 90 patients	RR 0.82<br>(0.15–4.26)	108/1000	90/1000	18 fewer per 1,000<br>(from 90 fewer to 232 more)	⊕⊕◯◯<br>Low	3<br>(21.9)
< 110 mg/dL	(Reference)					4<br>(13.5)
> 180 mg/dL<br>1 RCT, 112 patients	RR 0.20<br>(0.04–1.07)	36/1000	7/1000	29 fewer per 1,000<br>(from 35 fewer to 2 more)	⊕⊕⊕◯<br>Moderate	-
144–180 mg/dL<br>2 RCTs, 639 patients	RR 0.23<br>(0.05–1.07)	85/1000	21/1000	64 fewer per 1,000<br>(from 80 fewer to 5 more)	⊕⊕◯◯<br>Low	-
110–144 mg/dL	(Reference)					-

>180 mg/dL<br>1 RCT, 419 patients	RR 0.86<br>(0.35–2.12)	10/1000	9/1000	1 fewer per 1,000<br>(from 6 fewer to 11 more)	⊕⊕⊕◯<br>Moderate	-
144–180 mg/dL	(Reference)					-

CI: confidence interval; RR: risk ratio; SUCRA: surface under the cumulative ranking.

Summary of Judgements

	JUDGEMENT						
**Problem**	No	Probably no	Probably yes	**Yes**		Varies	Don't know
**Desirable Effects**	Trivial	Small	Moderate	Large		**Varies**	Don't know
**Undesirable Effects**	Large	Moderate	Small	Trivial		**Varies**	Don't know
**Certainty of evidence**	Very low	**Low**	Moderate	High			No included studies
**Values**	Important uncertainty or variability	Possibly important uncertainty or variability	**Possibly no important uncertainty or variability**	No important uncertainty or variability			
**Balance of effects**	Favors the comparison	Probably favors the comparison	Does not favor either the intervention or the comparison	Probably favors the intervention	Favors the intervention	**Varies**	Don't know
**Cost effectiveness**	Favors the comparison	Probably favors the comparison	Does not favor either the intervention or the comparison	Probably favors the intervention	Favors the intervention	Varies	**No included studies**
**Resources required**	Large costs	Moderate costs	**Negligible costs and savings**	Moderate savings	Large savings	Varies	Don't know
**Acceptability**	No	Probably no	**Probably yes**	Yes		Varies	Don't know
**Feasibility**	No	Probably no	Probably yes	**Yes**		Varies	Don't know

CQ6-4: Is antipyretic therapy provided to febrile patients with sepsis?

P	Sepsis
I	Antipyretic therapy
C	Usual care
O	28 or 30-day mortality, long-term mortality, hospital mortality, serious adverse events, infectious complications

Evidence profile

Certainty assessment							Number of patients		Effect		Certainty	Importance
№ of studies	Study design	Risk of bias	Inconsistency	Indirectness	Imprecision	Other considerations	Intervention	Control	Relative (95% CI)	Absolute (95% CI)		
28 or 30-day mortality												
4	randomized trials	not serious	serious	not serious	serious	none	155/618 (25.1%)	149/618 (24.1%)	RR 1.18 (0.80 to 1.72)	43 more per 1,000 (from 48 fewer to 174 more)	⊕⊕◯◯ Low	Critical
Long-term mortality												
1	randomized trials	not serious	not serious	not serious	very serious	none	55/346 (15.9%)	57/344 (16.6%)	RR 0.96 (0.68 to 1.35)	7 fewer per 1,000 (from 53 fewer to 58 more)	⊕⊕◯◯ Low	Critical
Hospital mortality												
4	randomized trials	not serious	not serious	not serious	very serious	none	53/183 (29.0%)	56/174 (32.2%)	RR 0.99 (0.54 to 1.85)	3 fewer per 1,000 (from 148 fewer to 274 more)	⊕⊕◯◯ Low	Critical
Serious adverse events												
4	randomized trials	serious	serious	not serious	very serious	none	53/654 (8.1%)	57/658 (8.7%)	RR 1.01 (0.55 to 1.85)	1 more per 1,000 (from 39 fewer to 74 more)	⊕◯◯◯ Very low	Critical
Infectious complications												
3	randomized trials	not serious	not serious	not serious	very serious	none	21/254 (8.3%)	29/256 (11.3%)	RR 0.75 (0.38 to 1.48)	28 fewer per 1,000 (from 70 fewer to 54 more)	⊕⊕◯◯ Low	Critical

CI: confidence interval; RR: risk ratio.

Summary of Judgements

	JUDGEMENT						
**Problem**	No	Probably no	Probably yes	**Yes**		Varies	Don't know
**Desirable Effects**	**Trivial**	Small	Moderate	Large		Varies	Don't know
**Undesirable Effects**	Large	**Moderate**	Small	Trivial		Varies	Don't know
**Certainty of evidence**	Very low	**Low**	Moderate	High			No included studies
**Values**	Important uncertainty or variability	Possibly important uncertainty or variability	**Possibly no important uncertainty or variability**	No important uncertainty or variability			
**Balance of effects**	Favors the comparison	**Probably favors the comparison**	Does not favor either the intervention or the comparison	Probably favors the intervention	Favors the intervention	Varies	Don't know
**Cost effectiveness**	Favors the comparison	Probably favors the comparison	Does not favor either the intervention or the comparison	Probably favors the intervention	Favors the intervention	Varies	**No included studies**
**Resources required**	Large costs	Moderate costs	**Negligible costs and savings**	Moderate savings	Large savings	Varies	Don't know
**Acceptability**	No	Probably no	**Probably yes**	Yes		Varies	Don't know
**Feasibility**	No	Probably no	Probably yes	**Yes**		Varies	Don't know

CQ6-5: Is stress ulcer prophylaxis performed for patients with sepsis to prevent gastrointestinal hemorrhage?

P	Sepsis
I	Performing stress ulcer prophylaxis
C	Not performing stress ulcer prophylaxis
O	Gastrointestinal bleeding, mortality, pneumonia, serious adverse events, *Clostridioides* infection

Evidence profile

Certainty assessment							Number of patients		Effect		Certainty	Importance
№ of studies	Study design	Risk of bias	Inconsistency	Indirectness	Imprecision	Other considerations	Intervention	Control	Relative (95% CI)	Absolute (95% CI)		
Gastrointestinal bleeding												
30	randomized trials	serious	serious	not serious	not serious	none	292/3735 (7.8%)	433/3131 (13.8%)	RR 0.52 (0.39 to 0.69)	66 fewer per 1,000 (from 84 fewer to 43 fewer)	⊕⊕◯◯ Low	Critical
Mortality												
14	randomized trials	serious	not serious	not serious	not serious	none	684/2668 (25.6%)	614/2397 (25.6%)	RR 1.04 (0.95 to 1.14)	10 more per 1,000 (from 13 fewer to 36 more)	⊕⊕⊕◯ Moderate	Critical
Pneumonia												
15	randomized trials	serious	not serious	not serious	not serious	none	432/2755 (15.7%)	368/2391 (15.4%)	RR 1.05 (0.92 to 1.19)	8 more per 1,000 (from 12 fewer to 29 more)	⊕⊕⊕◯ Moderate	Critical
Serious adverse events												
7	randomized trials	serious	not serious	not serious	serious	none	84/2156 (3.9%)	72/1987 (3.6%)	RR 1.13 (0.83 to 1.54)	5 more per 1,000 (from 6 fewer to 20 more)	⊕⊕◯◯ Low	Critical
Clostridioides infection												
3	randomized trials	not serious	not serious	not serious	very serious	none	21/1807 (1.2%)	28/1800 (1.6%)	RR 0.75 (0.42 to 1.31)	4 fewer per 1,000 (from 9 fewer to 5 more)	⊕⊕◯◯ Low	Critical

CI: confidence interval; RR: risk ratio.

Summary of Judgements

	JUDGEMENT						
**Problem**	No	Probably no	**Probably yes**	Yes		Varies	Don't know
**Desirable Effects**	Trivial	Small	**Moderate**	Large		Varies	Don't know
**Undesirable Effects**	Large	Moderate	**Small**	Trivial		Varies	Don't know
**Certainty of evidence**	**Very low**	Low	Moderate	High			No included studies
**Values**	Important uncertainty or variability	Possibly important uncertainty or variability	**Possibly no important uncertainty or variability**	No important uncertainty or variability			
**Balance of effects**	Favors the comparison	Probably favors the comparison	Does not favor either the intervention or the comparison	Probably favors the intervention	**Favors the intervention**	Varies	Don't know
**Cost effectiveness**	Favors the comparison	Probably favors the comparison	Does not favor either the intervention or the comparison	Probably favors the intervention	Favors the intervention	Varies	**No included studies**
**Resources required**	Large costs	Moderate costs	**Negligible costs and savings**	Moderate savings	Large savings	Varies	Don't know
**Acceptability**	No	Probably no	Probably yes	**Yes**		Varies	Don't know
**Feasibility**	No	Probably no	Probably yes	**Yes**		Varies	Don't know

CQ7-1: Is early rehabilitation implemented to prevent PICS?

P	Critically ill patients
I	Early rehabilitation
C	Usual care
O	Muscle strength, cognitive function, mental health, activities of daily living, mortality, adverse events

Evidence profile

Certainty assessment							Number of patients		Effect		Certainty	Importance
№ of studies	Study design	Risk of bias	Inconsistency	Indirectness	Imprecision	Other considerations	Intervention	Control	Relative (95% CI)	Absolute (95% CI)		
Muscle strength												
2	randomized trials	very serious	not serious	not serious	serious	none	132	136	-	SMD 0.16 higher (from 0.08 lower to 0.40 higher)	⊕◯◯◯ Very ow	Critical
Cognitive function												
1	randomized trials	very serious	not serious	not serious	serious	none	84	81	-	MD 0.6 higher (from 0.25 lower to 1.45 higher)	⊕◯◯◯ Very ow	Critical
Mental health												
1	randomized trials	very serious	not serious	not serious	serious	none	21	16	-	MD 0.3 higher (from 4.92 lower to 5.52 higher)	⊕◯◯◯ Very ow	Critical
Activities of daily living												
2	randomized trials	not serious	not serious	not serious	serious	none	153	151	-	SMD 0.57 higher (from 0.1 higher to 1.05 higher)	⊕⊕⊕◯ Moderate	Critical
Mortality												
5	randomized trials	serious	not serious	not serious	serious	none	52/354 (14.7%)	47/344 (13.7%)	RR 1.08 (0.74 to 1.56)	11 more per 1,000 (from 36 fewer to 77 more)	⊕⊕◯◯ Low	Critical
Adverse events												
3	randomized trials	serious	not serious	not serious	very serious	none	26/283 (9.2%)	24/267 (9.0%)	RR 0.92 (0.36 to 2.38)	7 fewer per 1,000 (from 58 fewer to 124 more)	⊕◯◯◯ Very ow	Critical

CI: confidence interval; MD: mean difference; RR: risk ratio; SMD: standardized mean difference.

Summary of Judgements

	JUDGEMENT						
**Problem**	No	Probably no	**Probably yes**	Yes		Varies	Don’t know
**Desirable Effects**	Trivial	**Small**	Moderate	Large		Varies	Don’t know
**Undesirable Effects**	Large	Moderate	Small	**Trivial**		Varies	Don’t know
**Certainty of evidence**	**Very low**	Low	Moderate	High			No included studies
**Values**	Important uncertainty or variability	Possibly important uncertainty or variability	**Possibly no important uncertainty or variability**	No important uncertainty or variability			
**Balance of effects**	Favors the comparison	Probably favors the comparison	Does not favor either the intervention or the comparison	**Probably favors the intervention**	Favors the intervention	Varies	Don’t know
**Cost effectiveness**	Favors the comparison	Probably favors the comparison	Does not favor either the intervention or the comparison	Probably favors the intervention	Favors the intervention	Varies	**No included studies**
**Resources required**	Large costs	Moderate costs	**Negligible costs and savings**	Moderate savings	Large savings	Varies	Don’t know
**Acceptability**	No	Probably no	**Probably yes**	Yes		Varies	Don’t know
**Feasibility**	No	Probably no	**Probably yes**	Yes		Varies	Don’t know

CQ7-2: Is neuromuscular electrical stimulation used to prevent ICU-acquired weakness (ICU-AW)?

P	Critically ill patients
I	Neuromuscular electrical stimulation
C	Usual care
O	ICU-acquired weakness, health-related quality of life, mortality, adverse events

Evidence profile

Certainty assessment							Number of patients		Effect		Certainty	Importance
№ of studies	Study design	Risk of bias	Inconsistency	Indirectness	Imprecision	Other considerations	Intervention	Control	Relative (95% CI)	Absolute (95% CI)		
ICU-acquired weakness												
6	randomized trials	not serious	not serious	not serious	serious	none	25/131 (19.1%)	60/143 (42.0%)	RR 0.48 (0.32 to 0.72)	218 fewer per 1,000 (from 285 fewer to 117 more)	⨁⨁⨁◯ Moderate	Critical
Health-related quality of life												
1	randomized trials	serious	not serious	not serious	serious	none	21	26	-	MD 0.2 higher (from 0.03 lower to 0.43 higher)	⨁⨁◯◯ Low	Critical
Mortality												
14	randomized trials	serious	not serious	not serious	serious	none	126/475 (26.5%)	120/47 (25.5%)	RR 1.07 (0.87 to 1.31)	18 more per 1,000 (from 33 fewer to 79 more)	⨁⨁◯◯ Low	Critical
Adverse events												
8	randomized trials	serious	not serious	not serious	serious	none	7/280 (2.5%)	0/268 (0.0%)	RR 6.87 (0.84 to 56.50)	10 more per 1,000 (from 20 fewer to 40 more)	⨁⨁◯◯ Low	Critical

CI: confidence interval; MD: mean difference; RR: risk ratio.

Summary of Judgements

	JUDGEMENT						
**Problem**	No	Probably no	**Probably yes**	Yes		Varies	Don't know
**Desirable Effects**	Trivial	Small	**Moderate**	Large		Varies	Don't know
**Undesirable Effects**	Large	Moderate	Small	**Trivial**		Varies	Don't know
**Certainty of evidence**	Very low	**Low**	Moderate	High			No included studies
**Values**	Important uncertainty or variability	Possibly important uncertainty or variability	**Possibly no important uncertainty or variability**	No important uncertainty or variability			
**Balance of effects**	Favors the comparison	Probably favors the comparison	Does not favor either the intervention or the comparison	**Probably favors the intervention**	Favors the intervention	Varies	Don't know
**Cost effectiveness**	Favors the comparison	Probably favors the comparison	Does not favor either the intervention or the comparison	Probably favors the intervention	Favors the intervention	Varies	**No included studies**
**Resources required**	Large costs	**Moderate costs**	Negligible costs and savings	Moderate savings	Large savings	Varies	Don't know
**Acceptability**	No	Probably no	**Probably yes**	Yes		Varies	Don't know
**Feasibility**	No	Probably no	**Probably yes**	Yes		Varies	Don't know

CQ7-3: Is follow up after ICU discharge be implemented to improve physical, cognitive, and mental functions?

P	Critically ill patients
I	Follow up after ICU discharge
C	Usual care
O	Physical impairment, Cognitive impairment, Mental impairment (post-traumatic stress disorder), Mental impairment (depression)

Evidence profile

Certainty assessment							Number of patients		Effect		Certainty	Importance
№ of studies	Study design	Risk of bias	Inconsistency	Indirectness	Imprecision	Other considerations	Intervention	Control	Relative (95% CI)	Absolute (95% CI)		
Physical impairment												
1	randomized trials	serious	not serious	not serious	serious	none	112	107	-	MD 15.0 lower (from 25.41 lower to 4.59 lower)	⊕⊕◯◯ Low	Critical
Cognitive impairment												
1	randomized trials	serious	not serious	not serious	serious	none	111	106	-	MD 0.30 lower (from 1.35 lower to 0.75 higher)	⊕⊕◯◯ Low	Critical
Mental impairment (post-traumatic stress disorder)												
3	randomized trials	serious	very serious	not serious	serious	none	296	305	-	SMD 0.1 higher (from 0.42 lower to 0.62 higher)	⊕◯◯◯ Very low	Critical
Mental impairment (depression)												
2	randomized trials	serious	not serious	not serious	serious	none	217	222	-	SMD 0.00 (from 0.19 lower to 0.19 higher)	⊕⊕◯◯ Low	Critical

CI: confidence interval; MD: mean difference; RR: risk ratio; SMD: standardized mean difference.

Summary of Judgements

	JUDGEMENT						
**Problem**	No	Probably no	**Probably yes**	Yes		Varies	Don't know
**Desirable Effects**	Trivial	**Small**	Moderate	Large		Varies	Don't know
**Undesirable Effects**	Large	Moderate	Small	**Trivial**		Varies	Don't know
**Certainty of evidence**	**Very low**	Low	Moderate	High			No included studies
**Values**	Important uncertainty or variability	Possibly important uncertainty or variability	**Possibly no important uncertainty or variability**	No important uncertainty or variability			
**Balance of effects**	Favors the comparison	Probably favors the comparison	Does not favor either the intervention or the comparison	**Probably favors the intervention**	Favors the intervention	Varies	Don't know
**Cost effectiveness**	Favors the comparison	Probably favors the comparison	Does not favor either the intervention or the comparison	Probably favors the intervention	Favors the intervention	Varies	**No included studies**
**Resources required**	Large costs	**Moderate costs**	Negligible costs and savings	Moderate savings	Large savings	Varies	Don't know
**Acceptability**	No	Probably no	**Probably yes**	Yes		Varies	Don't know
**Feasibility**	No	Probably no	Probably yes	Yes		**Varies**	Don't know

CQ7-4: Is rehabilitation after hospital discharge implemented to improve physical, cognitive, and mental functions?

P	Critically ill patients
I	Rehabilitation after hospital discharge
C	Usual care
O	Physical function after hospital discharge, cognitive function after hospital discharge, mental impairment (depression) after hospital discharge, adverse events

Evidence profile

Certainty assessment							Number of patients		Effect		Certainty	Importance
№ of studies	Study design	Risk of bias	Inconsistency	Indirectness	Imprecision	Other considerations	Intervention	Control	Relative (95% CI)	Absolute (95% CI)		
Physical function after hospital discharge												
4	randomized trials	serious	not serious	not serious	serious	none	65	66	-	SMD 0.17 higher (from 0.17 lower to 0.52 higher)	⊕⊕◯◯ Low	Critical
Cognitive function after hospital discharge												
1	randomized trials	serious	not serious	not serious	serious	none	7	8	-	MD 3.5 higher (from 1.56 higher to 5.44 higher)	⊕⊕◯◯ Low	Critical
Mental impairment (depression) after hospital discharge												
2	randomized trials	not serious	serious	not serious	serious	none	30	27	-	MD 0.24 lower (from 3.53 lower to 3.05 higher)	⊕⊕◯◯ Low	Critical
Adverse events												
9	randomized trials	serious	not serious	not serious	serious	none	13/282 (4.6%)	3/276 (1.1%)	RR 3.66 (1.23 to 10.82)	29 more per 1,000 (from 2 more to 107 more)	⊕⊕◯◯ Low	Critical

CI: confidence interval; MD: mean difference; RR: risk ratio; SMD: standardized mean difference.

Summary of Judgements

	JUDGEMENT						
**Problem**	No	Probably no	**Probably yes**	Yes		Varies	Don't know
**Desirable Effects**	Trivial	**Small**	Moderate	Large		Varies	Don't know
**Undesirable Effects**	Large	Moderate	Small	**Trivial**		Varies	Don't know
**Certainty of evidence**	Very low	**Low**	Moderate	High			No included studies
**Values**	Important uncertainty or variability	Possibly important uncertainty or variability	**Possibly no important uncertainty or variability**	No important uncertainty or variability			
**Balance of effects**	Favors the comparison	Probably favors the comparison	Does not favor either the intervention or the comparison	**Probably favors the intervention**	Favors the intervention	Varies	Don't know
**Cost effectiveness**	Favors the comparison	Probably favors the comparison	Does not favor either the intervention or the comparison	Probably favors the intervention	Favors the intervention	Varies	**No included studies**
**Resources required**	Large costs	Moderate costs	Negligible costs and savings	Moderate savings	Large savings	**Varies**	Don't know
**Acceptability**	No	Probably no	Probably yes	Yes		**Varies**	Don't know
**Feasibility**	No	Probably no	**Probably yes**	Yes		Varies	Don't know

CQ 8-1: Is written information provided to families of critically ill patients?

P	Critically ill patients and their families
I	Written information provision related to intensive care
C	Usual care
O	Anxiety and Depression, anxiety subscale, depression subscale, post-traumatic stress disorder, satisfaction, comprehension

Evidence profile

Certainty assessment							Number of patients		Effect		Certainty	Importance
№ of studies	Study design	Risk of bias	Inconsistency	Indirectness	Imprecision	Other considerations	Intervention	Control	Relative (95% CI)	Absolute (95% CI)		
Anxiety and depression												
2	randomized trials	serious	not serious	not serious	serious	none	96	97	-	MD 4.52 lower (from 6.71 lower to 2.33 lower)	⊕⊕◯◯ Low	Critical
Cognitive function after hospital discharge												
2	randomized trials	serious	not serious	not serious	serious	none	79	80	-	SMD 0.27 lower (from 0.68 lower to 0.13 higher)	⊕⊕◯◯ Low	Critical
Depression subscale												
2	randomized trials	serious	not serious	not serious	serious	none	79	80	-	SMD 0.23 lower (from 0.54 lower to 0.08 higher)	⊕⊕◯◯ Low	Critical
Post-traumatic stress disorder												
2	randomized trials	serious	not serious	not serious	serious	none	96	97	-	MD 9.39 lower (from 13.47 lower to 5.3 lower)	⊕⊕◯◯ Low	Critical
Satisfaction												
3	randomized trials	serious	not serious	not serious	serious	none	227	237	-	MD 1.26 lower (from 2.35 lower to 0.17 lower)	⊕⊕◯◯ Low	Critical
Comprehension												
1	randomized trials	serious	not serious	not serious	serious	none	77/87 (88.5%)	52/88 (59.1%)	RR 1.50 (1.24 to 1.81)	295 more per 1,000 (from 142 more to 479 more)	⊕⊕◯◯ Low	Critical

CI: confidence interval; MD: mean difference; RR: risk ratio; SMD: standardized mean difference.

Summary of Judgements

	JUDGEMENT						
Problem	No	Probably no	**Probably yes**	Yes		Varies	Don't know
Desirable Effects	Trivial	Small	**Moderate**	Large		Varies	Don't know
Undesirable Effects	Large	Moderate	Small	Trivial		Varies	**Don't know**
Certainty of evidence	Very low	**Low**	Moderate	High			No included studies
Values	Important uncertainty or variability	Possibly important uncertainty or variability	**Possibly no important uncertainty or variability**	No important uncertainty or variability			
Balance of effects	Favors the comparison	Probably favors the comparison	Does not favor either the intervention or the comparison	Probably favors the intervention	**Favors the intervention**	Varies	Don't know
Cost effectiveness	Favors the comparison	Probably favors the comparison	Does not favor either the intervention or the comparison	Probably favors the intervention	Favors the intervention	Varies	**No included studies**
Resources required	Large costs	Moderate costs	**Negligible costs and savings**	Moderate savings	Large savings	Varies	Don't know
Acceptability	No	Probably no	Probably yes	**Yes**		Varies	Don't know
Feasibility	No	Probably no	Probably yes	**Yes**		Varies	Don't know

CQ 8-4: Is an ICU diary kept for critically ill patients?

P	Critically ill patients and their families
I	ICU diary
C	Usual care
O	Acute stress disorder / post-traumatic stress disorder, anxiety, depression

Evidence profile

Certainty assessment							Number of patients		Effect		Certainty	Importance
№ of studies	Study design	Risk of bias	Inconsistency	Indirectness	Imprecision	Other considerations	Intervention	Control	Relative (95% CI)	Absolute (95% CI)		
Acute stress disorder / post-traumatic stress disorder (patients)												
4	randomized trials	serious	not serious	not serious	serious	none	241	254	-	SMD 0.13 lower (from 0.32 lower to 0.06 higher)	⊕⊕◯◯ Low	Critical
Acute stress disorder / post-traumatic stress disorder (families)												
3	randomized trials	serious	not serious	not serious	serious	none	343	352	-	SMD 0.09 lower (from 0.29 lower to 0.11 higher)	⊕⊕◯◯ Low	Critical
Anxiety (patients)												
4	randomized trials	serious	not serious	not serious	serious	none	227	237	-	MD 1.15 lower (from 2.59 lower to 0.28 higher)	⊕⊕◯◯ Low	Critical
Anxiety (families)												
2	randomized trials	serious	not serious	not serious	serious	none	64/144 (44.4%)	45/125 (36.0%)	RR 1.16 (0.88 to 1.53)	58 more per 1,000 (from 43 fewer to 191 more)	⊕⊕◯◯ Low	Critical
Depression (patients)												
4	randomized trials	serious	not serious	not serious	serious	none	227	235	-	MD 0.39 lower (from 1.06 lower to 0.28 higher)	⊕⊕◯◯ Low	Critical
Depression (families)												
2	randomized trials	serious	not serious	not serious	serious	none	71/144 (49.3%)	59/125 (47.2%)	RR 0.96 (0.78 to 1.17)	19 fewer per 1,000 (from 104 fewer to 80 more)	⊕⊕◯◯ Low	Critical

CI: confidence interval; MD: mean difference; RR: risk ratio; SMD: standardized mean difference.

Summary of Judgements

	JUDGEMENT						
Problem	No	Probably no	**Probably yes**	Yes		Varies	Don't know
Desirable Effects	Trivial	**Small**	Moderate	Large		Varies	Don't know
Undesirable Effects	Large	Moderate	Small	**Trivial**		Varies	Don't know
Certainty of evidence	Very low	**Low**	Moderate	High			No included studies
Values	Important uncertainty or variability	Possibly important uncertainty or variability	**Possibly no important uncertainty or variability**	No important uncertainty or variability			
Balance of effects	Favors the comparison	Probably favors the comparison	Does not favor either the intervention or the comparison	**Probably favors the intervention**	Favors the intervention	Varies	Don't know
Cost effectiveness	Favors the comparison	Probably favors the comparison	Does not favor either the intervention or the comparison	Probably favors the intervention	Favors the intervention	Varies	**No included studies**
Resources required	Large costs	Moderate costs	**Negligible costs and savings**	Moderate savings	Large savings	Varies	Don't know
Acceptability	No	Probably no	Probably yes	**Yes**		Varies	Don't know
Feasibility	No	Probably no	Probably yes	**Yes**		Varies	Don't know

CQ 8-5: Is follow-up after ICU discharge provided to families of critically ill patients to improve their mental health?

P	Families of critically ill patients
I	Follow-up after ICU discharge
C	No follow-up
O	Anxiety, depression, post-traumatic stress disorder, psychological quality of life, health related quality of life

Evidence profile

Certainty assessment							Number of patients		Effect		Certainty	Importance
№ of studies	Study design	Risk of bias	Inconsistency	Indirectness	Imprecision	Other considerations	Intervention	Control	Relative (95% CI)	Absolute (95% CI)		
Anxiety												
5	randomized trials	serious	not serious	serious	not serious	none	447	653	-	SMD 0.03 lower (from 0.15 lower to 0.09 higher)	⊕⊕◯◯ Low	Critical
Depression												
5	randomized trials	serious	not serious	serious	not serious	none	447	653	-	SMD 0.03 lower (from 0.09 lower to 0.15 higher)	⊕⊕◯◯ Low	Critical
Post-traumatic stress disorder												
5	randomized trials	serious	not serious	serious	not serious	none	441	646	-	SMD 0.01 lower (from 0.14 lower to 0.11 higher)	⊕⊕◯◯ Low	Critical
Psychological quality of life												
5	randomized trials	not serious	not serious	serious	serious	None	126	143	-	SMD 0.06 lower (from 0.03 lower to 0.18 higher)	⊕⊕◯◯ Low	Critical
Health related quality of life												
5	randomized trials	not serious	not serious	serious	serious	None	126	143	-	SMD 0.11 lower (from 0.35 lower to 0.13 higher)	⊕⊕◯◯ Low	Critical

CI: confidence interval; MD: mean difference; RR: risk ratio; SMD: standardized mean difference.

Summary of Judgements

	JUDGEMENT						
**Problem**	No	Probably no	**Probably yes**	Yes		Varies	Don't know
**Desirable Effects**	**Trivial**	Small	Moderate	Large		Varies	Don't know
**Undesirable Effects**	Large	Moderate	Small	**Trivial**		Varies	Don't know
**Certainty of evidence**	Very low	**Low**	Moderate	High			No included studies
**Values**	Important uncertainty or variability	Possibly important uncertainty or variability	**Possibly no important uncertainty or variability**	No important uncertainty or variability			
**Balance of effects**	Favors the comparison	Probably favors the comparison	Does not favor either the intervention or the comparison	**Probably favors the intervention**	Favors the intervention	Varies	Don't know
**Cost effectiveness**	Favors the comparison	Probably favors the comparison	Does not favor either the intervention or the comparison	Probably favors the intervention	Favors the intervention	Varies	**No included studies**
**Resources required**	Large costs	**Moderate costs**	Negligible costs and savings	Moderate savings	Large savings	Varies	Don't know
**Acceptability**	No	Probably no	Probably yes	Yes		**Varies**	Don't know
**Feasibility**	No	Probably no	Probably yes	Yes		**Varies**	Don't know

CQ9-5: Are steroids administered to pediatric patients with septic shock who are unresponsive to initial fluid therapy and vasopressors?

P	Pediatric patients with septic shock who are unresponsive to initial fluid therapy and vasopressors
I	Routine systemic steroids
C	Usual care
O	Mortality, infectious complication, length of hospitalization, duration until recovery from shock

Evidence profile

Certainty assessment							Number of patients		Effect		Certainty	Importance
№ of studies	Study design	Risk of bias	Inconsistency	Indirectness	Imprecision	Other considerations	Intervention	Control	Relative (95% CI)	Absolute (95% CI)		
Mortality												
3	randomized trials	not serious	not serious	not serious	very serious	none	22/74 (29.7%)	29/81 (35.8%)	RR 0.84 (0.55 to 1.28)	57 fewer per 1,000 (from 161 fewer to 100 more)	⨁⨁◯◯ Low	Critical
Infectious complication												
2	randomized trials	not serious	not serious	not serious	very serious	none	7/42 (16.7%)	6/45 (13.3%)	RR 1.30 (0.49 to 3.46)	40 more per 1,000 (from 68 fewer to 328 more)	⨁⨁◯◯ Low	Critical
Length of hospitalization												
1	randomized trials	not serious	not serious	not serious	very serious	none	32	36	-	MD 3.2 days longer (from 0.13 shorter to 6.53 longer)	⨁⨁◯◯ Low	Critical
Duration until recovery from shock												
1	randomized trials	very serious	not serious	not serious	serious	None	32	36	-	MD 3.3 days shorter (from 3.97 shorter to 2.63 shorter)	⨁◯◯◯ Very low	Critical

CI: confidence interval; MD: mean difference; RR: risk ratio.

Summary of Judgements

	JUDGEMENT						
**Problem**	No	Probably no	Probably yes	**Yes**		Varies	Don’t know
**Desirable Effects**	Trivial	**Small**	Moderate	Large		Varies	Don’t know
**Undesirable Effects**	Large	Moderate	**Small**	Trivial		Varies	Don’t know
**Certainty of evidence**	**Very low**	Low	Moderate	High			No included studies
**Values**	Important uncertainty or variability	Possibly important uncertainty or variability	**Possibly no important uncertainty or variability**	No important uncertainty or variability			
**Balance of effects**	Favors the comparison	Probably favors the comparison	**Does not favor either the intervention or the comparison**	Probably favors the intervention	Favors the intervention	Varies	Don’t know
**Cost effectiveness**	Favors the comparison	Probably favors the comparison	Does not favor either the intervention or the comparison	Probably favors the intervention	Favors the intervention	Varies	**No included studies**
**Resources required**	Large costs	Moderate costs	**Negligible costs and savings**	Moderate savings	Large savings	Varies	Don’t know
**Acceptability**	No	Probably no	**Probably yes**	Yes		Varies	Don’t know
**Feasibility**	No	Probably no	Probably yes	**Yes**		Varies	Don’t know

CQ9-6: What is the optimal hemoglobin level for blood transfusion in pediatric patients with sepsis who have stable hemodynamics?

P	Pediatric critically ill patients
I	Lower hemoglobin threshold for transfusion
C	Higher hemoglobin threshold for transfusion
O	ICU mortality, hospital mortality, length of ICU stay, duration of mechanical ventilation, transfusion related complication, new or progressive multiple organ dysfunction

Evidence profile

Certainty assessment							Number of patients		Effect		Certainty	Importance
№ of studies	Study design	Risk of bias	Inconsistency	Indirectness	Imprecision	Other considerations	Intervention	Control	Relative (95% CI)	Absolute (95% CI)		
ICU mortality												
1	randomized trials	not serious	not serious	not serious	very serious	none	11/320 (3.4%)	8/317 (2.5%)	RR 1.37 (0.56 to 3.27)	9 more per 1,000 (from 11 fewer to 57 more)	⊕⊕◯◯ Low	Critical
Hospital mortality												
2	randomized trials	not serious	not serious	not serious	serious	none	12/10 (11.4%)	27/122 (22.1%)	RR 0.47 (0.23 to 0.90)	117 fewer per 1,000 (from 170 fewer to 22 more)	⊕⊕⊕◯ Moderate	Critical
Length of ICU stay												
3	randomized trials	not serious	serious	not serious	serious	none	425	439	-	MD 1.78 days shorter (from 2.7 shorter to 0.86 shorter)	⊕⊕◯◯ Low	Critical
Duration of mechanical ventilation												
3	randomized trials	not serious	serious	not serious	serious	none	425	439	-	MD 1.02 days shorter (from 1.77 shorter to 0.27 shorter)	⊕⊕◯◯ Low	Critical
Transfusion related complication												
1	randomized trials	not serious	not serious	not serious	very serious	none	97/320 (30.3%)	90/317 (28.4%)	RR 1.07 (0.83 to 1.34)	20 more per 1,000 (from 48 fewer to 97 more)	⊕⊕◯◯ Low	Critical
New or progressive multiple organ dysfunction												
1	randomized trials	not serious	not serious	not serious	very serious	none	38/320 (11.9%)	39/317 (12.3%)	RR 0.96 (0.63 to 1.45)	5 fewer per 1,000 (from 46 fewer to 55 more)	⊕⊕◯◯ Low	Critical

CI: confidence interval; MD: mean difference; RR: risk ratio.

Summary of Judgements

	JUDGEMENT						
**Problem**	No	Probably no	Probably yes	**Yes**		Varies	Don't know
**Desirable Effects**	Trivial	**Small**	Moderate	Large		Varies	Don't know
**Undesirable Effects**	Large	Moderate	Small	**Trivial**		Varies	Don't know
**Certainty of evidence**	Very low	**Low**	Moderate	High			No included studies
**Values**	**Important uncertainty or variability**	Possibly important uncertainty or variability	Possibly no important uncertainty or variability	No important uncertainty or variability			
**Balance of effects**	Favors the comparison	Probably favors the comparison	Does not favor either the intervention or the comparison	**Probably favors the intervention**	Favors the intervention	Varies	Don't know
**Cost effectiveness**	Favors the comparison	Probably favors the comparison	Does not favor either the intervention or the comparison	Probably favors the intervention	Favors the intervention	Varies	**No included studies**
**Resources required**	Large costs	Moderate costs	**Negligible costs and savings**	Moderate savings	Large savings	Varies	Don't know
**Acceptability**	No	Probably no	Probably yes	**Yes**		Varies	Don't know
**Feasibility**	No	Probably no	Probably yes	**Yes**		Varies	Don't know

CQ9-7: Is strict blood glucose control performed for pediatric sepsis?

P	Pediatric critically ill patients
I	Strict blood glucose control
C	Usual care
O	Short-term mortality, length of ICU stay, duration of mechanical ventilation, hypoglycemia, infectious complication

Evidence profile

Certainty assessment							Number of patients		Effect		Certainty	Importance
№ of studies	Study design	Risk of bias	Inconsistency	Indirectness	Imprecision	Other considerations	Intervention	Control	Relative (95% CI)	Absolute (95% CI)		
Short-term mortality												
4	randomized trials	not serious	serious	not serious	serious	none	95/1879 (5.1%)	90/1858 (4.8%)	OR 1.05 (0.78 to 1.41)	2 more per 1,000 (from 10 fewer to 19 more)	⊕⊕◯◯ Low	Critical
Length of ICU stay												
3	randomized trials	not serious	serious	not serious	not serious	none	1533	1516	-	MD 0.51 days shorter (from 0.53 shorter to 0.49 shorter)	⊕⊕⊕◯ Moderate	Critical
Duration of mechanical ventilation												
3	randomized trials	not serious	not serious	not serious	not serious	none	1533	1516	-	MD 0.30 days shorter (from 0.32 shorter to 0.28 shorter)	⊕⊕⊕⊕ High	Critical
Hypoglycemia												
5	randomized trials	not serious	serious	not serious	not serious	none	382/1931 (19.8%)	141/200 (7.0%)	RR 3.08 (2.54 to 3.72)	146 more per 1,000 (from 108 more to 192 more)	⊕⊕⊕◯ Moderate	Critical
Infectious complication												
5	randomized trials	not serious	serious	not serious	serious	none	180/1931 (9.3%)	231/2002 (11.5%)	RR 0.84 (0.70 to 1.00)	18 fewer per 1,000 (from 35 fewer to 0)	⊕⊕◯◯ Low	Critical

CI: confidence interval; MD: mean difference; OR: odds ratio; RR: risk ratio.

Summary of Judgements

	JUDGEMENT						
**Problem**	No	Probably no	Probably yes	**Yes**		Varies	Don't know
**Desirable Effects**	**Trivial**	Small	Moderate	Large		Varies	Don't know
**Undesirable Effects**	Large	**Moderate**	Small	Trivial		Varies	Don't know
**Certainty of evidence**	Very low	**Low**	Moderate	High			No included studies
**Values**	Important uncertainty or variability	Possibly important uncertainty or variability	**Possibly no important uncertainty or variability**	No important uncertainty or variability			
**Balance of effects**	Favors the comparison	**Probably favors the comparison**	Does not favor either the intervention or the comparison	Probably favors the intervention	Favors the intervention	Varies	Don't know
**Cost effectiveness**	Favors the comparison	Probably favors the comparison	Does not favor either the intervention or the comparison	Probably favors the intervention	Favors the intervention	Varies	**No included studies**
**Resources required**	Large costs	Moderate costs	**Negligible costs and savings**	Moderate savings	Large savings	Varies	Don't know
**Acceptability**	No	Probably no	**Probably yes**	Yes		Varies	Don't know
**Feasibility**	No	Probably no	**Probably yes**	Yes		Varies	Don't know
