# Supplementary material for: The Japanese Clinical Practice Guidelines for Management of Sepsis and Septic Shock 2024
Source: J Intensive Care. 2025 Mar 14;13:15. doi: 10.1186/s40560-025-00776-0 (PMC11907869; doi:10.1186/s40560-025-00776-0)
Supplement: Supplementary file 4 — Additional file 4 [file 40560_2025_776_MOESM4_ESM.pdf]

Committee for the Japanese Clinical Practice Guidelines for the Management of Sepsis and Septic Shock 2024
- Member's names, conflicts of interest (COI), and roles -

Panelists, Organizers

Name	Position	Area of GL	Academic COI		Financial COI													
			Position in academic society	Contribution to other GL	A-1	A-2	A-3	A-4	A-5	A-6	A-7	A-8	A-9	B-1	B-2	B-3	C-1	C-2
Nobuaki Shime	Chairparson		Japanese Society of Intensive Care Medicine（director）<br>Japanese Society of Respiratory Care Medicine（director）<br>Japanese Society of Pediatric Intensive and Critical Care (director)	JRS Guidelines for the Management of Pneumonia in Adults	No	No	No	Gilead（2021）/BD（2022）/Asahi-Kasei Pharma（2023）	No	No	Asahi-Kasei Pharma（2021/2022/2023）	No	No	No	No	No	No	No
Taka-aki Nakada	Chairparson				Smart119（2021/2022/2023）	Smart119（2021/2022/2023）	No	No	No	No	Nihon Kohden（2021）	No	No	No	No	No	No	No
Yoshitaka Aoki	Panelist	AC			No	No	No	No	No	No	No	No	No	No	No	No	No	No
Shigeaki Inoue	Panelist	WG7,WG8, PR			No	No	No	No	No	No	No	No	No	No	No	No	No	No
Toshiaki Iba	Panelist	WG6			No	No	No	Japan Blood Products Organization（2022/2023）/ Toray Medical（2022）	No	JIMRO（2023）	Asahi-Kasei Pharma（2021/2022/2023）	No	No	No	No	No	No	No
Moritoki Egi	Panelist, Director	WG2,WG6	Japanese Society of Intensive Care Medicine（director）	ARDS clinical practice guidline 2021	No	No	No	No	No	No	No	No	No	No	No	No	No	No
Hiroshi Ogura	Panelist	WG1, Scenario			No	No	No	No	No	No	No	No	No	No	No	No	No	No
Yusuke Kawai	Panelist	WG8	Japanese Society for Early Mobilization（director）	Japanese Clinical Practice Guidelines for Rehabilitation in Critically Ill Patients 2023<br>Clinical practice guidelines on tracheal suctioning 2023; for adults with artificial airway	No	No	No	No	No	No	No	No	No	No	No	No	No	No
Atsushi Kawaguchi	Panelist	WG9		Japanese Practice Guidelines for Inter-hospital Transport of Critically ill Patients	Konica Minolta（2023）	No	No	No	No	Fisher & Paykel Healthcare（2023）	No	No	No	No	No	No	No	No

Tatsuya Kawasaki	Panelist	WG9		ARDS clinical practice guidline2021<br>Japanese rapid/living recommendations on drug management for COVID-19<br>Consensus statement on Children with multisystem inflammatory syndrome associated with COVID-19(MIS-C/PIMS)<br>Japanese Clinical Practice Guidelines for Rehabilitation in Critically Ill Patients2023<br>Operational Guideline on Rapid Response System	No	No	No	No	No	No	No	No	No	No	No	No	No	No
Shigeki Kushimoto	Panelsit, Director		Japanese Association for Acute Medicine（director）<br>Japanese Society of Intensive Care Medicine（director）<br>Japanese Socierty for the Acute Care Surgery（director）<br>Japanese Society for Abdominal Emergency Midicine（director）<br>Japanese College of Surgeons（director）<br>Japan Trauma Care and Research（director）<br>Japanese Association for the Surgery of Trauma（director）<br>General social foundation Japan Skin Bank Network（supervisor）	Transfusion Guideline for patients with Massive Bleeding<br>Revised Guidelines for the Establishment and Operation of Remote ICUs<br>Japanese Practice Guidelines for Inter-hospital Transport of Critically ill Patients<br>Japan Advanced Trauma Evaluation and Care Guidelines<br>DIC clinical practice guideline<br>Japan Expert Trauma Evaluation and Care Guidelines	No	No	No	No	No	No	No	No	No	No	No	No	No	No
Yutaka Kondo	Panelist	WG2		Japanese Clinical Practice Guidelines for Rehabilitation in Critically Ill Patients2023<br>Heatstroke Guideline 2024<br>JCS 2022 Guideline on Perioperative Cardiovascular Assessment and Management for Non-cardiac Surgery<br>ARDS clinical practice guidline2021	No	No	No	No	No	No	No	Toppan（2021~）	No	No	No	No	No	No
Masaaki Sakuraya	Panelist	WG3		ARDS clinical practice guidline2021	No	No	No	No	No	No	No	No	No	No	No	No	No	No
Shunsuke Taito	Panelist	WG7		ARDS clinical practice guidline2021<br>Japanese Clinical Practice Guidelines for Rehabilitation in Critically Ill Patients 2023	No	No	No	No	No	No	No	No	No	No	No	No	No	No
Kent Doi	Panelist	WG4, Databank, Scenario	Japan Society for Blood Purification in Critical Care（chairperson）	Guidelines for treatment of renal injury during cancer chemotherapy 2022	No	No	No	Asahi-Kasei Pharma（2023）	No	Nipro（2022）	No	No	No	No	No	No	No	No

Hideki Hashimoto	Panelist	WG1		Japanese Practice Guidelines for Inter-hospital Transport of Critically ill Patients<br>Japanese rapid/living recommendations on drug management for COVID-19	No	No	No	No	No	No	No	No	No	No	No	No	No	No
Yoshitaka Hara	Panelist	WG4			No	No	No	No	No	No	No	No	No	No	No	No	No	No
Tatsuma Fukuda	Panelist	WG3		JRC Guideline 2025	No	No	No	No	No	No	No	No	No	No	No	No	No	No
Asako Matsushima	Panelist	WG3	Japanese Association for Acute Medicine（director）	Clinical Practice Guidelines for Management of Burn Care<br>Surviving Sepsis After Burn Campaign	No	No	No	No	No	No	No	No	No	No	No	No	No	No
Tomoaki Yatabe	Vice chairparson	WG6		ARDS clinical practice guidline2021	No	No	No	No	No	No	No	No	No	No	No	No	No	No
Kazuma Yamakawa	Vice chairparson	WG5		DIC clinical practice guideline	No	No	No	Japan Blood Products Organization（2022/2023）	No	Japan Blood Products Organization（2023）/JIMRO（2021）/Fujimori Kogyo（2023）	Asahi-Kasei Pharma（2021/2022/2023）	No	No	No	No	No	No	No

Academic Guideline Promotion Team members

Name	Position	Area of GL	Academic COI		Financial COI													
			Position in academic society	Contribution to other GL	A-1	A-2	A-3	A-4	A-5	A-6	A-7	A-8	A-9	B-1	B-2	B-3	C-1	C-2
Atsuhiro Ijiri	AC			Japanese Guidelines for Nutrition Support Therapy in the Critically Ill Patients	No	No	No	No	No	No	No	No	No	No	No	No	No	No
Kei Ugata	AC	WG3,WG4			No	No	No	No	No	No	No	No	No	No	No	No	No	No
Shuji Uda	AC				No	No	No	No	No	No	No	No	No	No	No	No	No	No
Yutaka Umemura	AC	WG1			No	No	No	No	No	No	No	No	No	No	No	No	No	No
Takehiko Oami	AC	LLM for SR			No	No	No	Japan Blood Products Organization（2023）	No	No	No	No	No	No	No	No	No	No
Kohei Ota	AC,WG	WG8		ARDS clinical practice guidline2021<br>Japanese Clinical Practice Guidelines for Rehabilitation in Critically Ill Patients2023<br>Clinical practice guidelines on tracheal suctioning 2023; for adults with artificial airway	No	No	No	No	No	No	No	No	No	No	No	No	No	No

Hiromu Okano	AC			ARDS clinical practice guidline2021<br>Clinical practice guidelines on tracheal suctioning 2023; for adults with artificial airway<br>Japanese rapid/living recommendations on drug management for COVID-19<br>Neurointensive Care Guideline<br>Japanese Practice Guidelines for Inter-hospital Transport of Critically ill Patients	No	No	No	No	No	No	No	No	No	No	No	No	No	No
Ryuta Onodera	AC			Heatstroke Guideline	No	No	No	No	No	No	No	No	No	No	No	No	No	No
Ken-ichi Kano	AC,WG	WG2,PR		ARDS clinical practice guidline2021<br>Heatstroke Guideline 2024<br>Practice Guidelines for Primary Care of Acute Abdomen 2024<br>Japanese Guidelines for Nutrition Support Therapy in the Critically Ill Patients	No	No	No	No	No	No	No	No	No	No	No	No	No	No
Sadatoshi Kawakami	AC,WG	WG3			No	No	No	No	No	No	No	No	No	No	No	No	No	No
Akira Kawakami	AC,WG,SR	WG2			No	No	No	No	No	No	No	No	No	No	No	No	No	No
Yusuke Kawamura	AC,WG,SR	WG7,PR			No	No	No	No	No	No	No	No	No	No	No	No	No	No
Kazuya Kikutani	AC			Japanese Practice Guidelines for Inter-hospital Transport of Critically ill Patients	No	No	No	No	No	No	No	No	No	No	No	No	No	No
Yuki Kotani	AC,WG	WG3			No	No	No	No	No	No	No	No	No	No	No	No	No	No
Masaki Takahashi	AC	WG9			No	No	No	No	No	No	No	No	No	No	No	No	No	No
Chikashi Takeda	AC				No	No	No	No	No	No	No	No	No	No	No	No	No	No
Takero Terayama	AC			Neurointensive Care Guideline<br>Japanese Practice Guidelines for Inter-hospital Transport of Critically ill Patients<br>ARDS clinical practice guidline2021<br>Japanese rapid/living recommendations on drug management for COVID-19	No	No	No	Gilead (2022)	Yodosha (2021)<br>Herusu Publishing (2021)	JA-Kyosai (2022)	No	No	No	No	No	No	No	No
Satoshi Nakajima	AC			Heatstroke Guideline 2024	No	No	No	No	No	No	No	No	No	No	No	No	No	No
Katsuhiko Hashimoto	AC,WG	WG6			No	No	No	No	No	No	No	Yes	No	No	No	No	No	No

Junta Honda	AC	WG6			No	No	No	No	No	No	No	No	No	No	No	No	No	No
Yuto Makino	AC,WG,SR	WG5			No	No	No	No	No	No	No	No	No	No	No	No	No	No
Tsuguhiro Matsumoto	AC				No	No	No	No	No	No	No	No	No	No	No	No	No	No
Akito Mizuno	AC	WG4			No	No	No	No	No	No	No	No	No	No	No	No	No	No
Sohma Miyamoto	AC	WG3			No	No	No	No	No	No	No	No	No	No	No	No	No	No
Shunsuke Yasuo	AC,SR	WG5		ARDS clinical practice guidline 2021<br>Heatstroke Guideline 2024	No	No	No	No	No	No	No	No	No	No	No	No	No	No
Yuki Wakabayashi	AC,WG,SR	WG3		Japanese Clinical Practice Guidelines for Rehabilitation in Critically Ill Patients 2023	No	No	No	No	No	No	No	No	No	No	No	No	No	No
Takeshi Wada	AC	WG5, Scenario			No	No	No	Asahi-Kasei Pharma (2022/2023)<br>Japan Blood Products Organization (2022)	No	Takeda Science Foundation (2021)	No	No	No	No	No	No	No	No

Working group members

Name	Position	Area of GL	Academic COI		Financial COI													
			Position in academic society	Contribution to other GL	A-1	A-2	A-3	A-4	A-5	A-6	A-7	A-8	A-9	B-1	B-2	B-3	C-1	C-2
Gen Aikawa	WG	WG7			No	No	No	No	No	Terumo Life Science Foundation (2023)	No	No	No	No	No	No	No	No
Masayuki Akatsuka	WG	WG6			No	No	No	No	No	No	No	No	No	No	No	No	No	No
Hideki Asai	WG	WG8			No	No	No	No	No	No	No	No	No	No	No	No	No	No
Tadashi Ishihara	WG	WG9		Heatstroke Guideline 2024<br>ARDS clinical practice guidline 2021	No	No	No	No	No	No	No	No	No	No	No	No	No	No
Tadayoshi Ishimaru	WG	WG3			No	No	No	No	No	No	No	No	No	No	No	No	No	No
Akira Endo	WG	WG3			No	No	No	No	No	No	No	No	No	No	No	No	No	No
Marina Oi	WG	WG3			No	No	No	No	No	No	No	No	No	No	No	No	No	No

Akira Ouchi	WG	WG8		Japanese Clinical Practice Guidelines for Rehabilitation in Critically Ill Patients<br>Japanese Guidelines for Nutrition Support Therapy in the Critically Ill Patients<br>Clinical practice guidelines on tracheal suctioning 2023; for adults with artificial airway	No	No	No	No	No	No	No	No	No	No	No	No	No	No
Itsuki Osawa	WG	WG4			No	No	No	No	No	No	No	No	No	No	No	No	No	No
Takanori Ohno	WG	WG1			No	No	No	No	No	No	No	No	No	No	No	No	No	No
Masahiro Kashiura	WG	WG3		ARDS clinical practice guidline 2021	No	No	No	No	No	No	No	No	No	No	No	No	No	No
Daisuke Kasugai	WG	WG2			No	No	No	Japan Blood Products Organization (2023)<br>TXP medical (2023)	No	No	No	No	No	No	No	No	No	No
Ryo Kamidani	WG	WG9			No	No	No	No	No	No	No	No	No	No	No	No	No	No
Yuki Kishihara	WG,SR	WG3		ARDS clinical practice guidline 2021<br>Heatstroke Guideline 2024	No	No	No	No	No	No	No	No	No	No	No	No	No	No
Sho Kimura	WG	WG9			No	No	No	No	No	No	No	No	No	No	No	No	No	No
Kenji Kubo	WG	WG2		JSTB Guideline for the Diagnosis and Treatment of Tuberculosis	No	No	No	No	No	No	No	No	No	No	No	No	No	No
Tomoki Kuribara	WG	WG3		Japanese Clinical Practice Guidelines for Rehabilitation in Critically Ill Patients	No	No	No	No	No	No	No	No	No	No	No	No	No	No
Shigeru Koba	WG	WG3			No	No	No	No	No	No	No	No	No	No	No	No	No	No
Takehito Sato	WG	WG3			No	No	No	No	No	No	No	No	No	No	No	No	No	No
Ren Sato	WG	WG6			No	No	No	No	No	No	No	No	No	No	No	No	No	No
Yusuke Sawada	WG	WG6		Heatstroke Guideline 2024	No	No	No	No	No	No	No	No	No	No	No	No	No	No
Haruka Shida	WG	WG7			No	No	No	No	No	No	No	No	No	No	No	No	No	No
Motohiro Shimizu	WG	WG7			No	No	No	No	No	No	No	No	No	No	No	No	No	No
Takuto Shiraishi	WG	WG1			No	No	No	No	No	No	No	No	No	No	No	No	No	No
Toru Shinkai	WG	WG1			No	No	No	No	No	No	No	No	No	No	No	No	No	No
Kenji Kandori	WG	WG8			No	No	No	No	No	No	No	No	No	No	No	No	No	No
Gaku Sugiura	WG,SR	WG4			No	No	No	No	No	No	No	No	No	No	No	No	No	No

Hiroshi Sugimoto	WG	WG2		Japanese Guidelines for Nutrition Support Therapy in the Critically Ill Patients<br>ARDS clinical practice guidline 2021	No	No	No	No	No	No	No	No	No	No	No	No	No	No
Motohiro Sekino	WG	WG6			No	No	No	No	No	No	No	No	No	No	No	No	No	No
Kenji Sonota	WG	WG9			No	No	No	No	No	No	No	No	No	No	No	No	No	No
Mahoko Taito	WG	WG8		Japanese Guidelines for Nutrition Support Therapy in the Critically Ill Patients	No	No	No	No	No	No	No	No	No	No	No	No	No	No
Nozomi Takahashi	WG	WG2			No	No	No	No	No	No	No	No	No	No	No	No	No	No
Jun Takeshita	WG,SR	WG9	Japanese Society of Cardiovascular Anesthesiologists（director）	ARDS clinical practice guidline 2021<br>Japanese Guidelines for Nutrition Support Therapy in the Critically Ill Patients	No	No	No	No	No	No	No	No	No	No	No	No	No	No
Atsushi Tanikawa	WG	WG6			No	No	No	No	No	No	No	No	No	No	No	No	No	No
Masanori Tani	WG	WG9			No	No	No	No	No	No	No	No	No	No	No	No	No	No
Akihito Tampo	WG	WG4		Japanese Guidelines for Nutrition Support Therapy in the Critically Ill Patients	No	No	No	No	No	No	No	No	No	No	No	No	No	No
Takumi Tsuchida	WG	WG5			No	No	No	No	No	No	No	No	No	No	No	No	No	No
Yusuke Tsutsumi	WG	WG6		Japanese Clinical Practice Guidelines for Rehabilitation in Critically Ill Patients	No	No	No	No	No	No	No	No	No	No	No	No	No	No
Ryo Deguchi	WG	WG2			No	No	No	No	No	No	No	No	No	No	No	No	No	No
Takaaki Totoki	WG	WG5		DIC clinical practice guideline	No	No	No	No	No	No	No	No	No	No	No	No	No	No
Shunichiro Nakao	WG	WG1			No	No	No	No	No	No	No	No	No	No	No	No	No	No
Hiroki Nagasawa	WG	WG3			No	No	No	No	No	No	No	No	No	No	No	No	No	No
Yasuhisa Nakatani	WG	WG9			No	No	No	No	No	No	No	No	No	No	No	No	No	No
Mitsuaki Nishikimi	WG	WG3			No	No	No	No	No	No	No	No	No	No	No	No	No	No
Satoko Noguchi	WG	WG9			No	No	No	No	No	No	No	No	No	No	No	No	No	No
Suguru Nonami	WG	WG3			No	No	No	No	No	No	No	No	No	No	No	No	No	No
Osamu Nomura	WG	WG9		JRC Guideline	No	No	No	No	No	Toyota Foundation（2021）	No	No	No	No	No	No	No	No
Junji Hatakeyama	WG	WG8		Japanese Guidelines for Nutrition Support Therapy in the Critically Ill Patients	No	No	No	No	No	No	No	No	No	No	No	No	No	No
Yasutaka Hamai	WG	WG3		Japanese Guidelines for Nutrition Support Therapy in the Critically Ill Patients	No	No	No	No	No	No	No	No	No	No	No	No	No	No
Ryo Hisamune	WG	WG3			No	No	No	No	No	No	No	No	No	No	No	No	No	No

Ryota Fuke	WG,SR	WG2			No	No	No	No	No	No	No	No	No	No	No	No	No	No
Naoki Fujie	WG	WG5			No	No	No	No	No	No	No	No	No	No	No	No	No	No
Jun Fujinaga	WG	WG3			No	No	No	No	No	No	No	No	No	No	No	No	No	No
Yoshihisa Fujinami	WG	WG7		Japanese Guidelines for Nutrition Support Therapy in the Critically Ill Patients	No	No	No	No	No	No	No	No	No	No	No	No	No	No
Sho Fujiwara	WG	WG1			No	No	No	No	No	No	No	No	No	No	No	No	No	No
Hiroshi Matsuura	WG,SR	WG3		Clinical Practice Guidelines for Management of Burn Care	No	No	No	No	No	No	No	No	No	No	No	No	No	No
Ayaka Matsuoka	WG,SR	WG7			No	No	No	No	No	No	No	No	No	No	No	No	No	No
Tadashi Matsuoka	WG	WG5			No	No	No	No	No	No	No	No	No	No	No	No	No	No
Yukari Miyoshi	WG	WG2			No	No	No	No	No	No	No	No	No	No	No	No	No	No
Satoshi Murata	WG	WG9			No	No	No	No	No	No	No	No	No	No	No	No	No	No
Kohei Yamada	WG	WG3		ARDS clinical practice guidline 2021<br>Japanese Guidelines for Nutrition Support Therapy in the Critically Ill Patients<br>Japanese rapid/living recommendations on drug management for COVID-19	No	No	No	No	No	No	No	No	No	No	No	No	No	No
Hiroyuki Yamada	WG	WG4			No	No	No	No	No	No	No	No	No	No	No	No	No	No
Ryo Yamamoto	WG	WG1		Japanese Guidelines for Nutrition Support Therapy in the Critically Ill Patients	No	No	No	No	No	No	No	No	No	No	No	No	No	No
Tetsuya Yumoto	WG,SR	WG3			No	No	No	No	No	No	No	No	No	No	No	No	No	No
Yuji Yoshida	WG	WG1			No	No	No	No	No	No	No	No	No	No	No	No	No	No
Shodai Yoshihiro	WG	WG7		ARDS clinical practice guidline 2021<br>Japanese Clinical Practice Guidelines for Rehabilitation in Critically Ill Patients 2023	No	No	No	No	No	No	No	No	No	No	No	No	No	No
Satoshi Yoshimura	WG	WG1		Heatstroke Guideline 2024<br>ARDS clinical practice guidline 2021	No	No	No	No	No	No	No	No	No	No	No	No	No	No
Jumpei Yoshimura	WG	WG2			No	No	No	MSD (2021/2022/2023)	No	No	No	No	No	No	No	No	No	No
Hiroshi Yonekura	WG	WG3		ARDS clinical practice guidline 2026	No	No	No	No	No	No	No	No	No	No	No	No	No	No

Systematic review members

Name	Position	Area of GL	Academic COI		Financial COI

Name	Position	Area of GL	Position in academic society	Contribution to other GL	A-1	A-2	A-3	A-4	A-5	A-6	A-7	A-8	A-9	B-1	B-2	B-3	C-1	C-2
Makoto Aoki	SR	WG3		Transfusion Guideline for patients with Massive Bleeding	No	No	No	No	No	No	No	No	No	No	No	No	No	No
Yu Amemiya	SR	WG3			No	No	No	No	No	No	No	No	No	No	No	No	No	No
Noritaka Ushio	SR	WG5			No	No	No	No	No	No	No	No	No	No	No	No	No	No
Hisashi Imahase	SR	WG8			No	No	No	No	No	No	No	No	No	No	No	No	No	No
Haruki Imura	SR	WG2		JSTB Guideline for the Diagnosis and Treatment of Tuberculosis	No	No	No	No	No	No	No	No	No	No	No	No	No	No
Naoya Iwasaki	SR	WG4			No	No	No	No	No	No	No	No	No	No	No	No	No	No
Michiko Uchi	SR	WG8			No	No	No	No	No	No	No	No	No	No	No	No	No	No
Takeshi Umegaki	SR	WG6			No	No	No	No	No	No	No	No	No	No	No	No	No	No
Yoshiyasu Oshima	SR	WG2		ARDS clinical practice guidline 2021	No	No	No	No	No	No	No	No	No	No	No	No	No	No
Hiroyuki Koami	SR	WG5			No	No	No	No	No	No	No	No	No	No	No	No	No	No
Kazushige Shimizu	SR	WG4		ARDS clinical practice guidline 2021<br>Clinical practice guidelines on tracheal suctioning 2023; for adults with artificial airway	No	No	No	No	No	No	No	No	No	No	No	No	No	No
Kensuke Sugimoto	SR	WG7			No	No	No	No	No	No	No	No	No	No	No	No	No	No
Tomohiro Suhara	SR	WG6			No	No	No	No	No	No	No	No	No	No	No	No	No	No
Aiko Tanaka	SR	WG6			No	No	No	No	No	No	No	No	No	No	No	No	No	No
Hao Chen	SR	WG2		JRS Guideline for the Management of Chronic Obstructive Pilmonary Disease<br>JRS Guideline for the Management of Pneumonia 2024<br>JRS Guideline for the Management of Cough and Sputum	No	No	No	No	No	No	No	No	No	No	No	No	No	No

Takefumi Tsunemitsu	SR	WG3		Japanese Guidelines for Nutrition Support Therapy in the Critically Ill Patients	No	No	No	No	No	No	No	No	No	No	No	No	Healthtech Laboratory（2021/2022/2023）	Ritsuan（2023）ZOLL Medical Corporation（2022）
Kenichi Tetsuhara	SR	WG3			No	No	No	No	No	No	No	No	No	No	No	No	No	No
Nobuto Nakanishi	SR	WG7		Japanese Clinical Practice Guidelines for Rehabilitation in Critically Ill Patients Japanese Guidelines for Nutrition Support Therapy in the Critically Ill Patients	No	No	No	No	No	No	No	No	No	No	No	No	No	No
Norihiro Nishioka	SR	WG4		Clinical Practice Guidelines for the Management of Rheumatoid Arthritis Clinical Practice Guidelines for Rapidly Progressive Glomerulonephritis	No	No	No	No	No	No	No	No	No	No	No	No	No	No
Mayu Hikone	SR	WG3			No	No	No	No	No	No	No	No	No	No	No	No	No	No
Hiraku Funakoshi	SR	WG3			No	No	No	No	No	No	No	No	No	No	No	No	No	No
Yosuke Matsumura	SR	WG3			No	No	No	No	No	No	No	No	No	No	No	No	No	No
Teppei Murata	SR	WG3		ARDS clinical practice guidline 2021	No	No	No	No	No	No	No	No	No	No	No	No	No	No
Ryohei Yamamoto	SR	WG3			No	No	No	No	No	No	No	No	No	No	No	No	No	No
Shinichi Watanabe	SR	WG7		Japanese Guidelines for Nutrition Support Therapy in the Critically Ill Patients Japanese Clinical Practice Guidelines for Rehabilitation in Critically Ill Patients 2023	No	No	No	No	No	No	No	No	No	No	No	No	No	No

Guideline related working group member

Name	Position	Area of GL	Academic COI		Financial COI													
			Position in academic society	Contribution to other GL	A-1	A-2	A-3	A-4	A-5	A-6	A-7	A-8	A-9	B-1	B-2	B-3	C-1	C-2
Toshikazu Abe	WG	Databank			No	No	No	No	No	No	No	No	No	No	No	No	No	No

Ryo Ishizawa	WG	Aap			No	No	No	No	No	No	No	No	No	No	No	No	No	No
Yusuke Itosu	WG	PR			No	No	No	No	No	No	No	No	No	No	No	No	No	No
Hiroyasu Inoue	WG	Aap		Clinical practice guidelines on physical therapy: revision 2021<br>Japanese Clinical Practice Guidelines for Rehabilitation in Critically Ill Patients 2023	No	No	No	No	No	No	No	No	No	No	No	No	No	No
Masatoshi Uchida	WG	Databank			No	No	No	No	No	No	No	No	No	No	No	No	No	No
Yohei Okada	WG	LLM for SR		ARDS clinical practice guidline	No	No	No	No	No	No	No	No	No	No	No	No	No	No
Yoshihito Ogawa	WG	Scenario			No	No	No	No	No	No	No	No	No	No	No	No	No	No
Daisuke Kawakami	WG	PR		ARDS clinical practice guidline 2021	No	No	No	No	No	No	No	No	No	No	No	No	No	No
Tadanaga Shimada	WG	Databank			No	No	No	No	No	No	No	No	No	No	No	No	No	No
Junko Tatsuno	WG	Aap	Japanese Association for Emergency Nursing（director）	Japanese Clinical Practice Guidelines for Rehabilitation in Critically Ill Patients 2023<br>Japanese Guidelines for Nutrition Support Therapy in the Critically Ill Patients	No	No	No	No	No	No	No	No	No	No	No	No	No	No
Yuki Togami	WG	Aap			No	No	No	No	No	No	No	No	No	No	No	No	No	No
Yoshinori Tomoda	WG	Aap			No	No	No	No	No	No	No	No	No	No	No	No	No	No
Tomoya Hirose	WG	Aap			No	No	No	No	No	No	No	No	No	No	No	No	No	No
Ryo Fujii	WG	Scenario		ARDS clinical practice guidline 2021	No	No	No	No	No	No	No	No	No	No	No	No	No	No
Koichiro Homma	WG	Scenario			No	No	No	No	No	No	No	No	No	No	No	No	No	No
Hiromasa Yakushiji	WG	Aap			No	No	No	CSL Behring (2021/2022/2023)<br>Takeda Pharmaceutical (2021/2022/2023)<br>Vexon International (2021/2022/2023)	No	No	No	No	No	No	No	No	No	No

【Policy of COI disclosure】
・The Japanese Society of Intensive Care Medicine and the Japanese Association for Acute Medicine submitted this COI disclosure jointly, based on the same policy issued by the Japanese Association of Midical Sciences.

・Both groups used the modified submission format provided by the Japanese Association of Medical Sciences.
・In accordance with these guidelines, organizations are only required to disclose COI that relate to associated companies or for-profit organizations as financial COI.
・We asked all members to submit their financial and academic COI for the past three years, in accordance with the current policy, shown in the last table.

Standard of conflict of interest disclosure		
Financial COI	A-1	Presence of officer or advisor of for-profit company or organization, or received any remuneration (list those where the remuneration amount from one company/ organization is 1 million yen or more per year)
	A-2	Holding of shares or profits obtained from those shares (profit from shares in the last year) (list those where the annual profit of one company is 1 million yen or more, or those who own more than 5% of shares)
	A-3	Remuneration paid as a patent royalty from for-profit company or organization (list those where one patent royalty was 1 million yen or more)
	A-4	Remuneration such as a daily allowance, lecture fee, etc. paid for the time and labor requested by the researcher by a for-profit company or organization for attending meeting (e.g., presentation, advising) (list the lecture fee from one company/organization based on the annual total amount)
	A-5	Manuscript fee paid for writing a pamphlet, roundtable article, etc. created by a for-profit company or organization
	A-6	Research funds provided by a for-profit company or organization (list those where 1 million yen or more were actually allocated with a research contract from a one company/organization for medical research [e.g., joint research, contract research, clinical trial] that allows the filer to substantially decide the purpose of use)
	A-7	Scholarship (incentive) donations provided by a for-profit company or organization (list donations over 1 million yen actually allocated to the individual filer or the course/field of laboratory with which the filer is affilated, where they can substantially decide the purpose of use)
	A-8	Donation courses provided by companies, etc.(list donations of over 1 million yen actually allocated and can be used for practical purposes)
	A-9	Other remuneration (travel, gifts, etc. that are not directly related to research)(list based on the annual remuneration received from one company/organization)
	B-1	Presence of officer or advisor of for-profit company or organization, or received any remuneration (list based on the annual remuneration from one company/ organization)
	B-2	Holding of shares or profits obtained from those shares (profit from shares in the last year) (list the total annual profits of one company, or those holding 5% or more of shares)
	B-3	Remuneration paid as a patent royalty from for-profit company or organization (list those where one patent royalty was 1 million yen or more)
	C-1	Research funds provided by a for-profit company or organization (list those where research contract money was actually allocated for medical research [e.g., joint research, contract research] based on one company/organization or contract, where the applicable head of the organization/department could substantially determine the purpose of use)
	C-2	Scholarship (incentive) donations provided by a for-profit company or organization (list those where the donation was actually allocated from one company/for-profit organization with filer's research, and where the head of the research institution, hospital, faculty, center, or course could substantially determine the purpose of use)
Academic COI		Position higher than academic society director or auditor
		Involvement in the development of other guidelines
